# Mechanistic insight into benzylidene-directed glycosylation reactions using cryogenic infrared spectroscopy

**DOI:** 10.1038/s44160-024-00619-0

**Published:** 2024-07-26

**Authors:** Chun-Wei Chang, Kim Greis, Gurpur Rakesh D. Prabhu, Dana Wehner, Carla Kirschbaum, Katja Ober, América Y. Torres-Boy, Sabrina Leichnitz, Gerard Meijer, Gert von Helden, Peter H. Seeberger, Kevin Pagel

**Affiliations:** 1 Freie Universität Berlin, Institute of Chemistry and Biochemistry, Berlin, Germany; 2 Fritz Haber Institute of the Max Planck Society, Berlin, Germany; 3 Max Planck Institute of Colloids and Interfaces, Potsdam, Germany; 4 Present Address: Department of Chemistry and Applied Biosciences, ETH Zurich, Zurich, Switzerland; 5 Present Address: Kavli Institute for Nanoscience Discovery, University of Oxford, Oxford, UK

**Keywords:** Mass spectrometry, Carbohydrate chemistry, Infrared spectroscopy, Reaction mechanisms

## Abstract

The stereoselective formation of 1,2-*cis* glycosidic linkages is challenging. The currently most widely used strategy for their installation uses 4,6-*O*-benzylidene-protected building blocks. The stereoselectivity of this reaction is thought to be driven by a covalent intermediate, which reacts via an S_N_2 mechanism. However, the role of cationic S_N_1-type intermediates in this reaction is unclear. Here we elucidate the structure of glycosyl cations carrying 4,6-*O*-benzylidene groups using cryogenic infrared ion spectroscopy and computational methods. The data reveal that the intermediates form anhydro cations, which correlates well with the stereoselective outcome of S_N_1-type glycosylations. The study highlights how cryogenic infrared spectroscopy can elucidate the role of intermediates in sugar chemistry and how these structural data can be linked to reactions in solution.

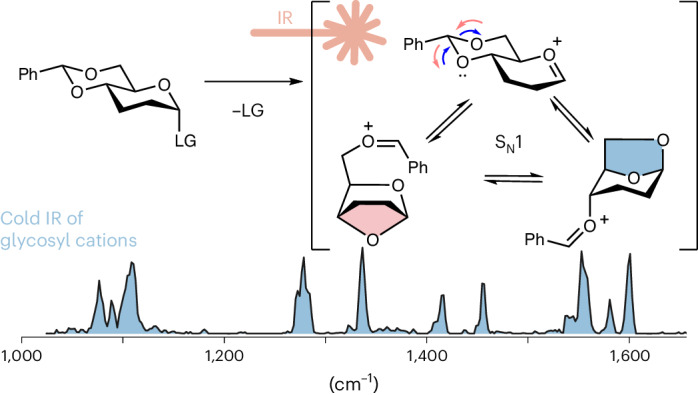

## Abstract (web summary)

The role of cationic intermediates in the benzylidene-directed synthesis of 1,2-*cis* glycosidic linkages is unclear. Now cryogenic infrared spectroscopy provides insight into the S_N_1 mechanism of benzylidene-directed glycosylation reactions. The analysis reveals that cationic intermediates form anhydro cations through a two-step process, which correlates with the observed stereochemical outcome.

## Main

Carbohydrates are ubiquitous in nature and are essential for many biological events. Specific 1,2-*cis* or 1,2-*trans* linkages are crucial for a variety of recognition processes and play a central role during viral infections^1,2^. Accessing pure oligosaccharides through chemical synthesis is challenging. The regiochemistry has to be controlled via an elaborate protecting group strategy; the emergence of a chiral centre during glycosidic bond formation complicates stereocontrol at the anomeric carbon. While 1,2-*trans* glycosides can be readily obtained using neighbouring group participation^3^, 1,2-*cis* selectivity is much more difficult to achieve because there is no universal method to reliably construct 1,2-*cis* glycosidic bonds^4,5^.

**Fig. 1:**
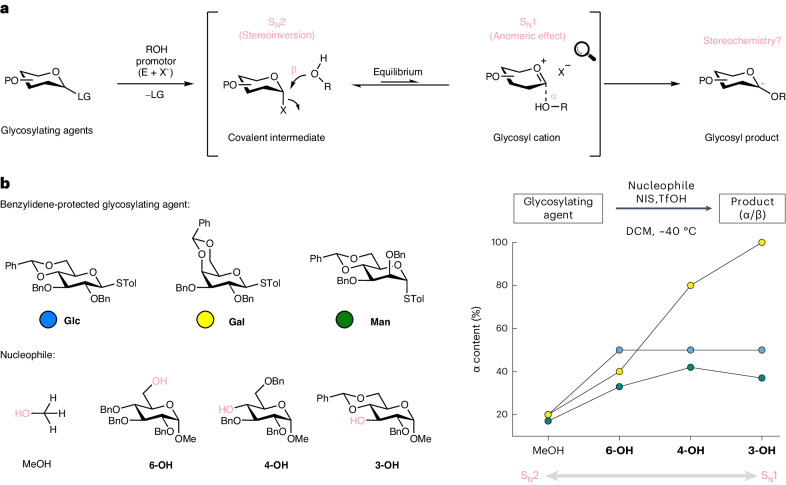
Stereoselectivity in glycosylation reactions. **a**, Schematic mechanisms of a glycosylation reaction. The stereochemistry of the products is determined by a S_N_1–S_N_2 continuum. **b**, Stereochemical outcome of condensed-phase benzylidene-directed glycosylation reactions. The experimental data were initially reported by Wang^31,32^. The examples shown here depend on the strength of the nucleophile; the reaction can be directed towards an S_N_2- or an S_N_1-like mechanism. High β-selectivity is obtained with strong nucleophiles, favouring an S_N_2 mechanism, whereas the α content increases for weak nucleophiles, indicating an S_N_1 mechanism. P, protecting group; LG, leaving group; Bn, benzyl; Tol, tolyl; NIS, N-iodosuccinimide; DCM, dichloromethane.

A better understanding of the reaction mechanism can help to optimize reaction conditions^3,6–10^. The mechanism of glycosylation reactions is a continuum between two extremes: an S_N_1-type mechanism involving a cationic intermediate on one hand, and an S_N_2-type reaction with a penta-coordinated transition state on the other (Fig. 1a)^10,11^. α-Glycosyl triflates in combination with strong nucleophiles almost exclusively lead to β-glycosides via an S_N_2 mechanism, as discovered by Crich^11–13^. Experiments using isotopically labelled glycosylating agents further provided evidence for the generation of α-selective β-triflates^14^. Although it is commonly agreed that α-covalent intermediate proceeds through S_N_2-like pathways with the majority of the data pointing towards an inversion mechanism, a S_N_1-like manifold could still be invoked in some instances^15–17^. For instance, it was also suggested that weak nucleophiles may not react via an S_N_2 mechanism^18^. Instead, the reaction mechanism shifts to a dissociative S_N_1 pathway, where transient cationic intermediates control stereoselectivity.

The glycosylation reaction is a complex organic transformation, and its mechanism remains not fully understood. Glycosylation could be kinetically controlled, and α-glycosylation is probably governed by conformational preference of the oxocarbenium ion^19^. Kerns and Ito have also discovered that an in situ anomerization of covalent intermediate drives an inversion mechanism^20,21^. However, many groups emphasize that temperature affects glycosylation reactions, suggesting a thermodynamically controlled process. The α-glycosyl product is observed at high temperatures and is preferred thermodynamically due to the anomeric effect^22^. Different temperature also influences the stability of the intermediate^23–25^, reaction pathways^26^ and stereoselective outcome^6,27^.

A promising method to preferentially obtain 1,2-*cis* glycosides is 4,6-*O*-benzylidene-directed glycosylations, which were initially introduced by Crich^16,28^ and further refined by Wang and Codée (Fig. 1b)^29–32^. With strong nucleophiles, such as methanol, the obtained α-selectivity is low (<20%). However, the α-selectivity gradually increases with weaker nucleophiles, such as isopropanol or monosaccharides. Strong nucleophiles therefore favour an S_N_2-type reaction, whereas weak nucleophiles more likely react via an S_N_1 mechanism. The increase in α-selectivity is significantly different for galactose (Gal), glucose (Glc) and mannose (Man). Galactose shows the highest α-selectivity with up to 100%, while glucose and mannose only reach between 40% and 50%, respectively. This distinct selectivity arises from a delicate interplay between different factors such as the anomeric effect, the structure of the oxocarbenium ion and the presence of other highly reactive species.

A detailed structural understanding of the reaction intermediates is essential to understand the glycosylation mechanism. Previously, the S_N_2 mechanism and its α-glycosyl triflate intermediate were studied using nuclear magnetic resonance (NMR) spectroscopy^14,16,23,33^. Typical counter ions, such as triflate or halides, are also known to undergo rapid equilibrium with glycosyl triflate through the anomerization of the leaving group, resulting in a Curtin–Hammett type scenario. This phenomenon has been further investigated by Taylor and Boltje using NMR exchange spectroscopy^34–36^. In contrast, the intermediate of S_N_1-type glycosylation reactions—the glycosyl cation—is inaccessible to traditional spectroscopic techniques because of its short lifetime (picoseconds)^33,37^. Nevertheless, valuable information on the S_N_1 trajectory has been obtained using density functional theory (DFT)^38,39^, kinetic isotope experiments^16,17^, cation clock reactions^15,40^ and NMR in super-acid media^41^. Despite these advances, no direct structural data of glycosyl cations in benzylidene-mediated glycosylations have been obtained so far.

Gas-phase spectroscopy is a powerful tool for the structural characterization of glycosyl cations^3,6–8,42–48^. Here we use a combination of cryogenic infrared (IR) spectroscopy in helium droplets and DFT to unravel the structure of glycosyl cations generated from 4,6-*O*-benzylidene-protected building blocks. Our data suggest that the intermediate forms an anhydro cation, which correlates with the stereoselective outcome observed for 4,6-*O*-benzylidene-protected glycosylating agents in combination with weak nucleophiles.

## Results and discussion

### Glycosyl cation characterization using cryogenic IR spectroscopy

The experimental setup has been described in detail previously (Supplementary Fig. 1)^49–51^. Briefly, glycosyl precursors are ionized and transferred into the gas phase by nano-electrospray ionization (nESI). The leaving group at the anomeric carbon (C1) of glycosyl precursors is subsequently cleaved through in-source fragmentation to generate glycosyl cations. Subsequently, mass-to-charge selected glycosyl cations are guided into a hexapole ion trap. A coaxial beam of superfluid helium nanodroplets (0.37 K) picks up the ions and transports them from the trap to an interaction region. Here the droplets overlap with an IR beam generated by the Fritz Haber Institute Free-Electron Laser^52^. Sequential absorption of multiple resonant photons leads to evaporation of the helium shell and ejection of the ions, which are detected by a time-of-flight mass analyser. The ion count as a function of the laser wavelength leads to a highly resolved IR spectrum. The experimental vibrational spectra can be linked to a glycosyl cation structure using DFT calculations. A conformational search using CREST^53^ with the semiempirical method GFN2-xTB^54^ yielded over 300 structures for each intermediate. Free energies and harmonic frequencies were computed at the PBE0 + D3/6-311 + G(d,p)^55,56^ level of theory in Gaussian 16 (ref. ^57^). Boltzmann analysis and four additional levels of DFT were used to validate the computed spectra (Supplementary Figs. 13–18 and 37–44).

**Fig. 2:**
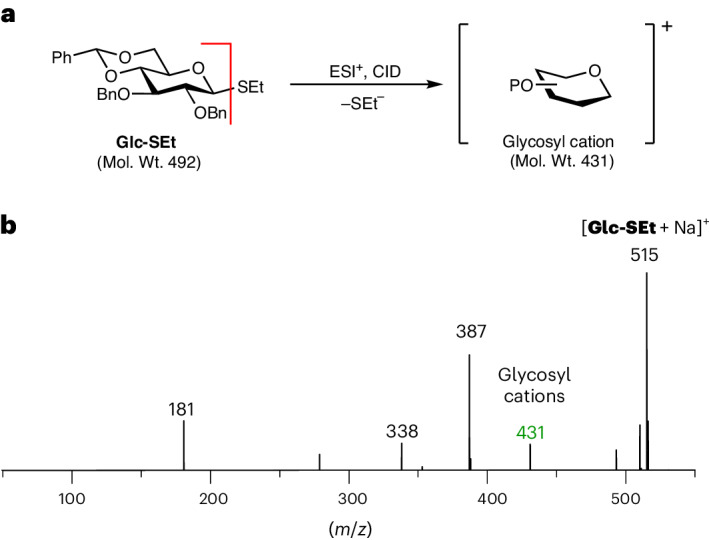
Formation of glycosyl cations using MS. **a**, Fragmentation of **Glc-SEt** precursors by in-source fragmentation after ESI leads to the formation of glycosyl cations. **b**, Fragmentation mass spectrum of **Glc-SEt** recorded on the helium droplet instrument. In-source fragmentation of precursor ions [M + Na]^+^ (*m*/*z* = 514) leads to the formation of glycosyl cations (*m*/*z* = 431). Bn, benzyl; Et, ethyl; CID, collision-induced dissociation; Mol. Wt., molecular weight.

Electrospray ionization (ESI) and in-source fragmentation of the thioethyl glucoside **Glc-SEt** leads to cleavage of the thioethyl (SEt) group and the formation of a glycosyl cation (Fig. 2a). The ESI–mass spectrometry (MS) spectra (Fig. 2b) reveal the presence of sodium adducts of glycosyl precursors [**Glc-SEt** + Na]^+^ at *m*/*z* of 514, and glycosyl cations at *m*/*z* 431 in positive ion mode. The glycosyl cations (*m*/*z* = 431) are accumulated in a hexapole ion trap, where they are pre-cooled to 90 K by collisions with helium buffer gas. Subsequently, the intermediate is trapped and rapidly cooled to 0.37 K within the superfluid helium environment, and its IR spectrum is recorded in the 1,000–1,800 cm^−1^ range.

**Fig. 3:**
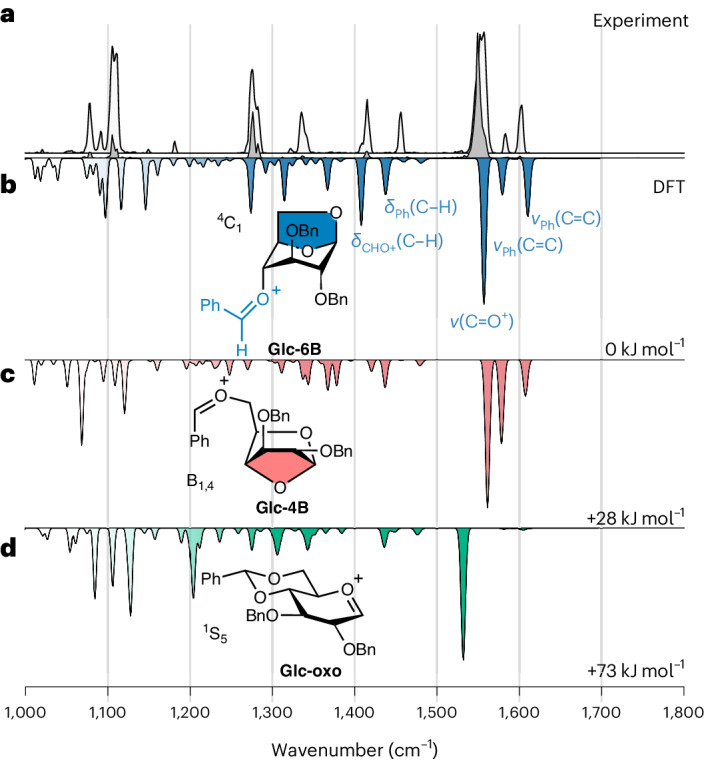
IR spectra of glucosyl cations with benzylidene groups. **a**, Experimental cryogenic IR spectrum of glucosyl cation carrying a benzylidene protecting group. Cryo-IR spectra were recorded at two different macropulse energies of the FEL. The low-energy FEL (20 mJ) produces a dark-grey spectrum, while the high-energy FEL (35 or 70 mJ) generates a white spectrum. **b**–**d**, The computed spectra of the lowest-energy structures 1,6-anhydro cation (**b**), 1,4-anhydro cation (**c**) and oxocarbenium ion (**d**) are represented in the inverted traces. Ring puckers and relative free energies at 90 K are indicated. *δ*, bending vibrations; *ν*, streching vibrations.

The cryo-IR spectra were recorded at three different macropulse energies of the free electron laser (FEL) (Fig. 3a and Supplementary Fig. 3). Lower FEL energies (dark grey, 20 mJ) prevent oversaturation and capture the most prominent absorption bands. Spectra with higher FEL energies (white, 35 and 70 mJ) are partially saturated but reveal additional absorption bands in the fingerprint regions. Initially, it was expected that the vibrational signature corresponds to an oxocarbenium-type ion **Glc-oxo** (Fig. 3d, green), which is a commonly suggested intermediate^10,11,33^. Surprisingly, oxocarbenium ions are not formed, because the characteristic C_1_=O_5_ stretching vibration in the experimental spectrum around 1,500 cm^−1^ is clearly missing^3,6^. Instead, the experimental spectrum is most consistent with the lowest-energy computed spectrum of an 1,6-anhydro cation **Glc-6B**. Here the benzylidene protecting group splits into a hydroxylate moiety at C6, which forms a covalent bond to the anomeric carbon, and a benzylium moiety (PhCHO^+^) at C4 (Fig. 3b, blue). Anhydro cations are more stable than oxocarbenium ions owing to delocalization of the positive charge in the PhCHO^+^ moiety. The characteristic IR pattern of anhydro cations falls into two main regions: the absorption of the benzylic carbocation in the higher-frequency region (1,400–1,650 cm^−1^) and the C–O and C–C stretching as well as C–H bending modes below 1,400 cm^−1^. Five major bands originating from the PhCHO^+^ moiety are diagnostic for anhydro cations: (1) the feature at 1,557 cm^−1^, which corresponds to the C=O^+^ stretch of the benzylium moiety, the bands at (2) 1,610 and (3) 1,579 cm^−1^, which are symmetric and antisymmetric C=C stretches within the phenyl ring, and the diagnostic vibrations at (4) 1,437 and (5) 1,408 cm^−1^, which are C–H bends of the CHO^+^ and the Ph moieties, respectively. Energetically, **Glc-6B** is found to be 73 kJ mol^−1^ more stable than the oxocarbenium ion **Glc-oxo**. The formation of an alternative 1,4-anhydro cation **Glc-4B**, where the generated hydroxylate group is at C4 (Fig. 3c, red), is conceivable as well. Its harmonic frequencies partially match the experimental IR spectrum; however, it overall matches less well than **Glc-6B** and is 28 kJ mol^−1^ less stable.

The rearrangement of a 4,6-*O*-benzylidene system into anhydro cations is particularly surprising because the 4,6-*O*-benzylidene acetal moiety is considered non-participating. That is, the 4,6-*O*-benzylidene acetal is not expected to interact with the anomeric carbon. The formation of anhydro cations not only results in a energetic stabilization of the intermediate but also twists the sugar into a more rigid [3.2.1]-bicyclic backbone^58^. As a result, all functional groups are reoriented into the rather unusual axial/pseudo-axial positions.

A clear correlation between the structure of the gas-phase intermediate and the stereoselectivity of the condensed-phase products is observed. In the solution-phase glycosylation reactions, the α content gradually increases when weak nucleophiles are used^31,32^. Under these conditions, an S_N_1 mechanism and the formation of a positively charged intermediate is likely. In the 1,6-anhydro cations experimentally detected here, the β-face is shielded, and a nucleophilic attack is favoured from the α-side. The 1,4-anhydro cations, on the other hand, have potential β-selectivity. However, the high relative energy level suggests that these intermediates are not particularly abundant. Taken together, the formation of anhydro cations in condensed-phase S_N_1-type glycosylations could explain the increased content of α-products. However, an involvement of β-triflates in these α-glycosylations cannot be ruled out on the basis of these data^14^.

**Fig. 4:**
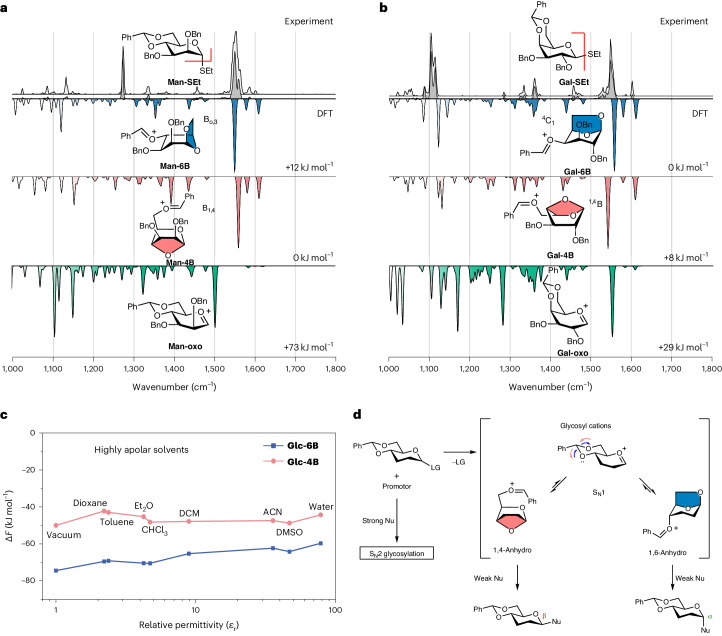
Structures and IR spectra of glycosyl cations with benzylidene group. **a**, IR spectra of mannosyl cations generated from **Man-SEt**. **b**, IR spectra of galactosyl cations generated from **Gal-SEt**. Three different FEL energies are used for recording IR spectra. The low-energy FEL (20 mJ) produces a dark-grey spectrum, while the high-energy FEL (35 or 70 mJ) generates a white spectrum. The computed spectra of the lowest-energy 1,6-anhydro cation (blue), 1,4-anhydro cation (red) and oxocarbenium ion (green) are represented in the inverted traces below. Ring puckers and relative free energies at 90 K are indicated. **c**, The relative free energies of **Glc-6B** (shown in blue) and **Glc-4B** (shown in red) calculated using the COSMO solvation model. The calculated free energies are referenced against the lowest-energy oxocarbenium structure **Glc-oxo** (Δ*F* = 0). The data suggest that the stability of anhydro intermediates is comparable in the gas phase and in various non-polar aprotic solvents. **d**, Postulated mechanism for benzylidene-mediated glycosylation on glucose and mannose. The predicted stereoselectivity for anhydro-type intermediates is correlated with the stereochemical outcome of benzylidene-mediated S_N_1-type glycosylation reactions. Bn, benzyl; Et, ethyl; Ph, phenyl; Et_2_O, diethyl ether; DCM, dichloromethane; ACN, acetonitrile; DMSO, dimethyl sulfoxide; LG, leaving group; Nu, nucleophile.

Similar to the glucose intermediate, no indication for the presence of oxocarbenium-type structures **Man-oxo** is observed for the corresponding 4,6-*O*-benzylidene mannosyl cation (Fig. 4). Instead, the comparison between experiment and theory suggests the formation of an anhydro cation as well (Fig. 4a). The lowest-energy oxocarbenium-type structure is 73 kJ mol^−1^ less stable than the lowest-energy **Man-4B** 1,4-anhydro cation (B_1,4_, 0 kJ mol^−1^). The harmonic frequencies of this cation as well as those of its 1,6-anhydro counterpart **Man-6B** (B_o,3_, +12 kJ mol^−1^) match the experiment comparably well. Two distinct peaks at 1,547 cm^−1^ and 1,560 cm^−1^ were observed in the low-energy spectrum (dark grey), which could originate from the C=O^+^ stretch of the PhCHO^+^ moiety of the two intermediates (**Man-4B** and **Man-6B**), respectively. Although **Man-4B** is lower in energy, it is possible that both ions are generated in the experiment. As for glucose, the vibrational modes associated with the PhCHO^+^ moiety in the 1,400–1,650 cm^−1^ region are the major diagnostic features for the formation of anhydro cations. The main difference to the glucosyl cation spectrum is found in the fingerprint region (1,000–1,200 cm^−1^).

In the condensed phase, the mannosyl building block is more β-selective than its glucose counterpart when weak nucleophiles are used (Fig. 1b). Interestingly, the formation of the anhydro ring in the lowest-energy structure **Man-4B** leads to shielding of the α-side and a preferential nucleophilic attack from the β-side. The selectivity of this structure aligns well to the β-selectivity observed in benzylidene-directed mannosylatios^16,28,29,31^. The less stable **Man-6B**, on the other hand, would rather induce the formation of α-mannosides. One could speculate that the higher α-selectivity observed in glucose compared with mannose can be attributed to the involvement of both intermediates. Specifically, the higher stability of **Glc-6B** compared with **Glc-4B** promotes a greater α content. Conversely, the prevalence of β-mannosylation could be influenced by the higher thermodynamic stability of **Man-4B** in comparison with **Man-6B**.

For galactose, the computed spectra (Fig. 4b) of two anhydro cations **Gal-6B** (^4^C_1_, 0 kJ mol^−1^) and **Gal-4B** (^1,4^B, +8 kJ mol^−1^) are close to the experimental IR spectrum, compared with **Gla-oxo**. As for the other sugars, anhydro galactose cations are energetically preferred over oxocarbenium ions (+29 kJ mol^−1^). Although the two anhydro cations are structurally different, their computed free energies are similar, with **Gal-4B** being 8 kJ mol^−1^ less stable than **Gal-6B**. In contrast to Glc and Man, however, the β-side of the anomeric carbon is blocked in both structures **Gal-6B** and **Gal-4B**. This implies a pronounced α-selectivity in galactosylations with weak nucleophiles. Surprisingly, this is exactly what is observed in condensed-phase reactions using 4,6-*O*-benzylidene galactosyl building blocks, which show almost 100% α-selectivity (Fig. 1b)^59^.

### Correlating gas-phase structures to solution-phase reactions

The correlation between gas-phase and condensed-phase structures is the subject of intense debate^60,61^ and has been studied by many synthetic^8,33,44–46,62–64^ and physical chemistry laboratories^3,6,7,43,47,48,51^. Here the structure of glycosyl cations is probed in the vacuum of a mass spectrometer (relative permittivity *ε*_r_ = 1), which is considerably different to an aqueous solution (*ε*_r_ = 80). However, typical glycosylation reactions are not performed in water. Instead, highly apolar solvents, such as toluene (*ε*_r_ = 2.4) or dichloromethane (*ε*_r_ = 8.9), with a relative permittivity close to that of vacuum are used. Therefore, the presented gas-phase structural data are potentially relevant for typical reactions in glycochemistry.

To test the impact of the solvent on the relative stability of the generated intermediates, the calculated structures were reoptimized using the COSMO solvation model^65,66^ for various solvents with distinct relative permittivities (Fig. 4c and Supplementary Figs. 19–23). The free energies of the most stable glycosyl cations with a solvent model are compared with those of the gas-phase structures. The results suggest that anhydro cations are not only the most stable structures in the gas phase but also retain their stability when a solvent model is introduced. In the conformational analysis across various solvents, the resulting data support this notion. Vacuum environment yields a considerable number of low-energy conformers, and higher-energy conformers are revealed with increasing electric permittivity (*ε*_r_). However, the conformer distributions in vacuum align better with those observed in highly apolar organic solvents than with that obtained for water (Supplementary Figs. 38–44). Further, the computational data show that upon solvation the ring puckers largely remain identical to those adopted in the gas phase, whereas the side chains (OBn) orient differently. These results are in line with previous studies^67,68^, where IR spectra of other intermediates were found to be nearly identical, regardless of whether they were probed in solution or in the gas phase.

Glycosyl cations in solution are stabilized by various conjugate bases, such as promoters or leaving groups. To probe the counterion effect on the behaviour of glycosyl cations, a series of experiments was conducted using cryogenic IR spectroscopy, ion mobility MS^69,70^ and DFT calculations. First, we examined the conformation of glycosyl cations under different promoters (TMOSTf, TfOH and AcOH) using ion mobility MS (Supplementary Fig. 30). The resulting mobilogram of glycosyl cations displayed striking similarity, suggesting minimal influence from various promoters to alter conformation of glycosyl cation in the gas phase. Second, the structure of glycosyl cations was investigated across a range of glycosyl donors bearing leaving groups (for example, trichloroimidate, acetate, chloride and thio-aglycon) via cryogenic IR experiments (Supplementary Fig. 31). There is a difference in the detected signal intensity. However, as supported by our previous work^48^, the obtained IR signatures of corresponding glycosyl cations exhibited identical stretching patterns, indicating that structure of the glycosyl cation does not retain anomeric memory. Third, DFT calculations were employed to explore the effect of triflate anions (OTf^−^) (Supplementary Figs. 32–35). The calculated data suggest that OTf^−^ coordinates to the benzylic carboncation of anhydro sugars with a bond length estimated to be around 3.0 Å. This ion-pair configuration is thermodynamically more stable than the oxocarbenium ion. Additionally, it maintains the same sugar backbone as observed in the gas phase, where there is no counterion present, but only alters the orientation of the OBn side chain. Overall, it is important to note that the multi-pronged approach of gas-phase IR spectroscopy, ion mobility spectrometry–mass spectrometry (IMS-MS) and DFT provide a snapshot of the mechanism of origin of glycosidic bond formation. The data indicate that mechanistic insights obtained in gas phase can be extended to solution-phase reactions.

From a structural point of view, the anhydro cations bear a benzylic carbocation (PhCHO^+^), which may potentially act as an electrophile during glycosylation reactions. However, based on the analysis of electrostatic potentials (Supplementary Figs. 36 and 37)^71,72^, we observed that the carbon of benzylic carbocation moiety carries a very low charge (+0.22), probably due to charge delocalization facilitated by the adjacent phenyl group. Instead, the anomeric carbon (C1) still exhibits a high positive charge (+0.43), suggesting that C1 serves as the preferred electrophilic site for nucleophilic attacks during glycosylation reactions.

## Conclusions

We present here the direct structural characterization of glycosyl cation intermediates generated from benzylidene-protected glycosylating agents. The presented structures were derived by matching cryogenic gas-phase IR spectra with computed harmonic frequencies obtained from DFT calculations. For glucose, galactose and mannose, the intermediates unexpectedly form anhydro cations. The anhydro cation formation probably involves two steps (Fig. 4d): (1) the oxocarbenium ion undergoes a ring-opening reaction of the benzylidene acetal at O4 or O6 to generate a zwitterionic species^73^; (2) O6 or O4 remote participation leads to the formation of an anhydro ring^6^. The obtained structures show a clear connection between the structure of the intermediates and the stereoselective outcome in glycosylation reactions. The inverse selectivities of the anhydro cations correlate well with the mixed anomer ratios that are observed when benzylidene-protected building blocks are used under conditions that favour an S_N_1-type glycosylation. Computations on glycosyl cations with a solvent model furthermore reveal a high degree of similarity between the gas- and condensed-phase structures. The structural insights reported here provide a plausible mechanistic explanation of the S_N_1-type reactivity in benzylidene-directed glycosylations, while we acknowledge that participation of oxocarbenium ion still cannot be ignored. This discovery can serve as a guideline to fine tune their reactivity on the road to 1,2-*cis* selective glycosylation reactions.

## Methods

### MS and IR spectroscopy

The precursors were dissolved in a 9:1 (v/v) mixture of acetonitrile and water to yield 0.1 mM solutions. Pd/Pt-coated glass capillaries (Sputter Coater HR 208, Cressington) for nESI are pulled to a tip with an inner diameter of 1–2 µm using a micropipette puller (Model P-1000, Sutter Instrument). Glycosyl cations were generated and probed using a custom-built helium droplet instrument. Glycosyl cations are formed after nESI (Z-spray) with a voltage of 1.1 kV to the tip of the capillary of the precursors, followed by in-source fragmentation of the generated ions. Commonly, nESI of the precursor leads to sodiated and protonated ions, whereas in-source fragmentation can lead to the cleavage of labile leaving groups, such as SEt.

After passing through two ring-electrode ion guides, the ions of interest are mass-to-charge selected by a quadrupole mass filter. Then, the ions enter a quadrupole bender. If no voltage is applied, the ions directly pass through the bender to get to a time-of-flight detector to record mass spectra and to monitor the ion signal. If ±33 V are applied to rods of the quadrupole bender, the ions are bent and enter a hexapole ion trap that is cooled to 90 K by liquid nitrogen in this experiment. The ions of interest are subsequently accumulated in the ion trap and thermalized by collisions with helium buffer gas.

Expansion of pressurized helium into the vacuum by a pulsed Even-Lavie valve leads to the formation of a beam of superfluid helium nanodroplets (0.4 K) that traverses the ion trap, picking up ions, rapidly cooling them to their equilibrium temperature and guiding them to the detection region. Here an IR beam generated by the Fritz Haber Institute Free-Electron Laser overlaps with the ion beam. Upon the absorption of resonant photons, vibrational modes of the molecular ions are excited. The ions dissipate the energy to the helium matrix to get back to their ground state. After the absorption of multiple photons, the probed ions are released from the helium nanodroplets and detected by a time-of-flight detector. The ion yield can be plotted as a function of the IR wavenumber, leading to an IR spectrum. Due to the multiphoton absorption process, the intensities in the obtained IR spectrum do not scale linearly. As a first-order correction, the ion signal is divided by the energy of the IR macropulse.

### Computational methods

Initial geometries of glycosyl cations candidates (**Glc-oxo**, **Glc-4B**, **Glc-6B**, **Man-oxo**, **Man-4B**, **Man-6B**, **Gal-oxo**, **Gal-4B** and **Gal-6B**) were constructed by chemical intuition using GaussView 6 (ref. ^57^). Conformational search was performed using CREST^53^ with the semiempirical method GFN2-xTB^54^ using default settings. The selected structures are reoptimized and their harmonic frequencies are computed at the PBE1PBE/6-311 + G(d,p) EmpiricalDispersion=GD3BJ^55,56^ level of theory using Gaussian 16 (ref. ^57^). The calculated harmonic vibrational frequencies have been scaled with standard scaling factors 0.965. Relative free energies at 90 K (approximate temperature of the ion trap) were extracted from the frequency calculation and are represented with the energy Δ*E* (including zero-point vibrational energy) in the tables and figures below.

To evaluate the significance of various structures, four additional computational methods are employed: method 1 (wB97XD/def2TZVP), method 2 (M062X/cc-pvtz), method 3 (M062X/def2TZVP) and method 4 (PBE1PBE/def2TZVP EmpiricalDispersion=GD3BJ). Our findings indicate that anhydro cations consistently exhibit thermodynamic stability, showing the lowest free energy at 90 K compared with oxocarbenium ions. The calculated harmonic vibrational frequencies have been scaled with standard scaling factors 0.965. Furthermore, computational IR spectra generated using each method closely resemble those obtained with the PBE1PBE/6-311 + G(d,p) EmpiricalDispersion=GD3BJ level. This strengthens the assertion that anhydro cations are formed and observable in cold-IR experiments.

To study the counterion effect on glycosyl cations, initial geometries of glycosyl cations candidates bearing triflate anion (OTf^−^) were constructed by chemical intuition using GaussView 6 (ref. ^57^). Conformational search was performed using CREST^53^ with the semiempirical method GFN2-xTB^54^ using default settings. The selected structures are reoptimized and their harmonic frequencies are computed at the PBE1PBE/6-311 + G(d,p) EmpiricalDispersion=GD3BJ^55,56^ level of theory using Gaussian 16 (ref. ^57^).

## Supplementary information

Supplementary Information Supplementary Figs. 1–44, Discussion and Tables 1–7.

Supplementary Data 1 SI_coordinated_gas_phase.pdf.

Supplementary Data 2 SI_coordinated_solvents.pdf.

Supplementary Data 3 SI coordinated_counterion.pdf.

## Data Availability

The data reported in this paper are available in the main text or Supplementary Information. *xyz* coordinates of all reoptimized geometries at the PBE0 + D3/6-311 + G(d,p) level of theory can be found in Supplementary Data 1–3.
