# Supplementary material for: Mechanistic insight into benzylidene-directed glycosylation reactions using cryogenic infrared spectroscopy
Source: Nat Synth. 2024 Jul 26;3(11):1377–84. doi: 10.1038/s44160-024-00619-0 (PMC11549046; doi:10.1038/s44160-024-00619-0)
Supplement: Supplementary file 1 — Supplementary Figs. 1–44, Discussion and Tables 1–7. [file 44160_2024_619_MOESM1_ESM.pdf]

nature synthesis

Supplementary information

https://doi.org/10.1038/s44160-024-00619-0

Mechanistic insight into benzylidene-directed glycosylation reactions using cryogenic infrared spectroscopy

In the format provided by the authors and unedited

1. Mass Spectrometry and Infrared Spectroscopy 1

1.1 Experimental Setup 1

1.2 Infrared Spectra 3

1.3 Mass Spectra 5

2. Computational Methods 7

2.1 Method Description 7

2.2 Energetics of Intermediates in Vacuum 8

2.3 Energy Hierarchies of Intermediates in Vacuum 11

2.4 3D Structures of Intermediates in Vacuum 13

2.5 Structure Validation: Comparative Analysis Using Four Additional Computational Methods 16

2.6 Energetics of Intermediates Using a Solvent Model 23

2.7 Energy Hierarchies of Intermediates Using a Solvent Model 24

2.8 3D Structures of Intermediates in Different Solvents 28

3. Exploring the Impact of Counterions on the Structure of Glycosyl Cations 34

3.1 Promoter Effect on the Structure of Glycosyl Cations 34

3.2 Leaving Group Effect on the Structure of Glycosyl Cations 35

3.3 Study the Counterion Effect on the Structure of Glycosyl Cations using Computational Method 36

3.4 Analysis of the Electrophilic Site in Anhydro Cations 41

4. Conformational Analysis of Glycosyl Cations 43

4.1 Boltzmann Analysis of the Anhydro Cations 43

4.2 Solvents Impact on the Conformational Population of Glycosyl Cations. 44

5. xyz-Coordinates of Reoptimized Structures 50

6. Experimental Details 51

6.1 Materials 51

6.2 Probing the Stereoselectivity of 4,6-*O*-Benzylidene-Directed Glycosylation Reaction 68

References 71

1. Mass Spectrometry and Infrared Spectroscopy

1.1 Experimental Setup

The precursors were dissolved in a 9:1 (V:V) mixture of acetonitrile and water to yield 0.1 mM solutions. Pd/Pt coated glass capillaries (Sputter Coater HR 208, *Cressington*) for nano electrospray ionization (nESI) are pulled to a tip with an inner diameter of 1–2 μm using a micropipette puller (Model P-1000, *Sutter Instrument*).

Glycosyl cations were generated and probed using a custom-built helium droplet instrument (Figure S1). Glycosyl cations are formed after nESI (Z-spray) with a voltage of 1.1 kV to the tip of the capillary of the precursors, followed by in-source fragmentation of the generated ions. Commonly, nESI of the precursor leads to sodiated and protonated ions, whereas in-source fragmentation can lead to the cleavage of labile leaving groups, such as SEt.

After passing through two ring-electrode ion guides, the ions of interest are mass-to-charge selected by a quadrupole mass filter. Then, the ions enter a quadrupole bender. If no voltage is applied, the ions directly pass through the bender to get to a time-of-flight detector to record mass spectra (Figures S4-S6) and to monitor the ion signal. If ±33 V are applied to rods of the quadrupole bender, the ions are bent and enter a hexapole ion trap that is cooled to 90 K by liquid nitrogen in this experiment. The ions of interest are subsequently accumulated in the ion trap and thermalized by collisions with helium buffer gas.

Expansion of pressurized helium into the vacuum by a pulsed Even-Lavie valve leads to the formation of a beam of superfluid helium nanodroplets (0.37 K) that is traversing the ion trap, picking up ions, rapidly cooling them to their equilibrium temperature, and guiding them to the interaction region. Here, an infrared (IR) beam generated by the Fritz Haber Institute free-electron laser (FHI FEL[1]) overlaps with the ion beam. Upon the absorption of resonant photons, vibrational modes of the molecular ions are excited. The ions dissipate the energy to the helium matrix to get back to their ground state. After the absorption of multiple photons, the probed ions are released from the helium nanodroplets and detected by another time-of-flight detector. The ion yield can be plotted as a function of the IR wavenumber, leading to an IR spectrum (Figures 3, 4, S2 and S3). Due to the multiphoton absorption process, the intensities in the obtained IR spectrum do not scale linearly. As a first-order correction, the ion signal is divided by the energy of the IR macropulse.

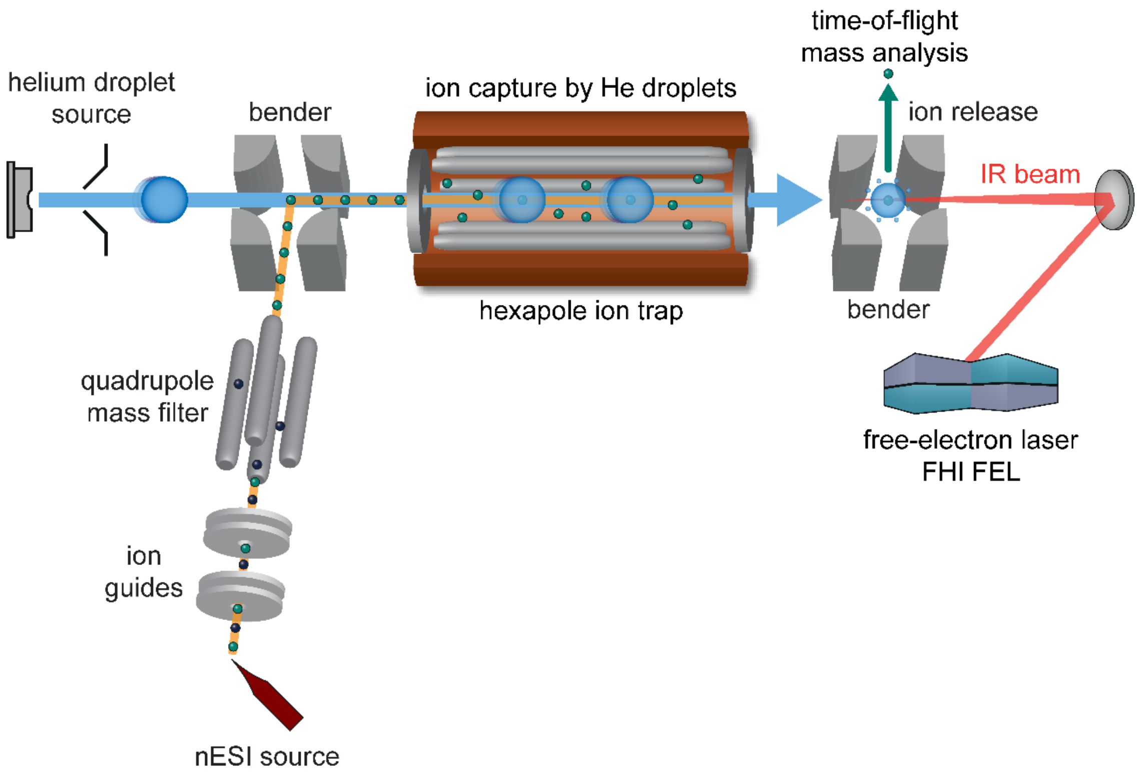

Figure S1. Schematic drawing of the custom-built helium droplet instrument combining mass spectrometry and infrared spectroscopy in helium droplets to probe mass-to-charge selected ions. Reproduced from https://doi.org/10.1021/jacs.2c05859,[2] under the terms of the CC BY 4.0 license, https://creativecommons.org/licenses/by/4.0/.

1.2 Infrared Spectra

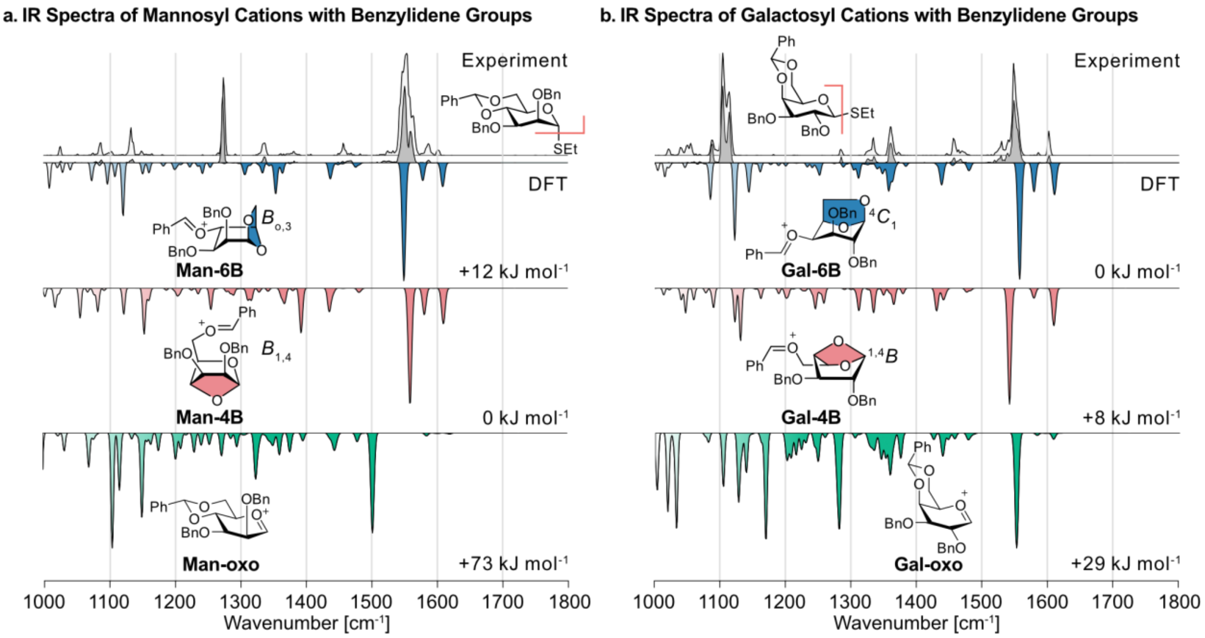

Figure S2. Cryogenic infrared spectra of glycosyl cations (m/z = 431) generated from **Man-SEt** and **Gal-SEt** precursors individually. The experimental cryogenic IR spectrum is shown as a gray trace. Dark gray represents the spectrum obtained using low-power free electron lasers (FEL), while the white represents the IR spectrum obtained using high-power levels of FEL. Besides, computed spectra of the lowest-energy 1,6-anhydro cation (blue), 1,4-anhydro cation (red) and oxocarbenium ion (green) for (a) mannosyl and (b) galactosyl cations are shown in the inverted traces below. The selected DFT structures are reoptimized and their harmonic frequencies are computed at the PBE1PBE/6-311+G(d,p) EmpiricalDispersion=GD3BJ level of theory using Gaussian 16. By employing a lower energy setting (20 mJ), we have mitigated oversaturation issues, capturing the most significant stretching bands while avoiding saturation. Subsequently, higher energy settings (35 and 70 mJ) were utilized, revealing additional stretching bands within the fingerprint regions, albeit with some saturation.

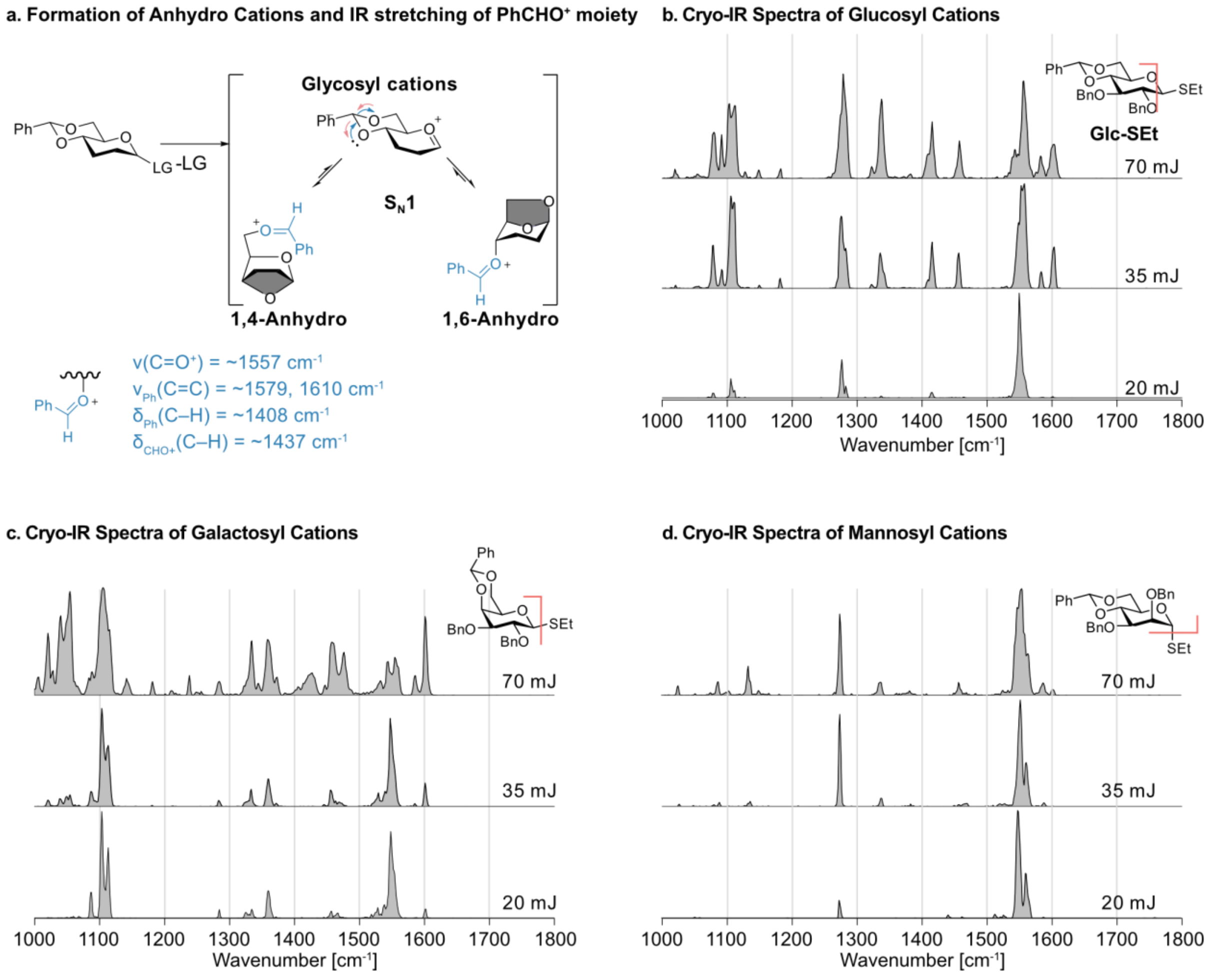

Figure S3. Cryogenic infrared spectra of glycosyl cations. a) Cryogenic infrared spectra of glycosyl cations (m/z = 431) generated from thioglycoside (Glc-SEt, Man-SEt, and Gal-SEt) individually. Different power levels of the free electron laser (FEL) are applied to obtain the cryo-IR spectra for b) glucosyl cation, c) galactosyl cation, and d) mannosyl cation. Applying low energy (20 mJ) of FEL results in a prominent stretching band at approximately 1557 cm^{-1}, originating from the $C=O^+$ stretch of the benzylium carbocation ($PhCHO^+$) of anhydro cations. The relative intensity of all stretching bands was clearly outlined. With increasing power of FEL (35 and 70 mJ), low-frequency peaks gradually appear, while the high-frequency peak at 1557 cm^{-1} becomes oversaturated. The IR stretching of the $PhCHO^+$ moiety provides the most diagnostic peaks for anhydro cations, with five major absorptions defined: The feature at 1557 cm^{-1} corresponds to the $C=O^+$ stretch. The bands at 1610 cm^{-1} and 1579 cm^{-1} represent symmetric and antisymmetric C=C stretches within the phenyl ring. Diagnostic vibrations at 1437 cm^{-1} and 1408 cm^{-1} correspond to C–H bends of the CHO^+ and Ph moieties, respectively.

1.3 Mass Spectra

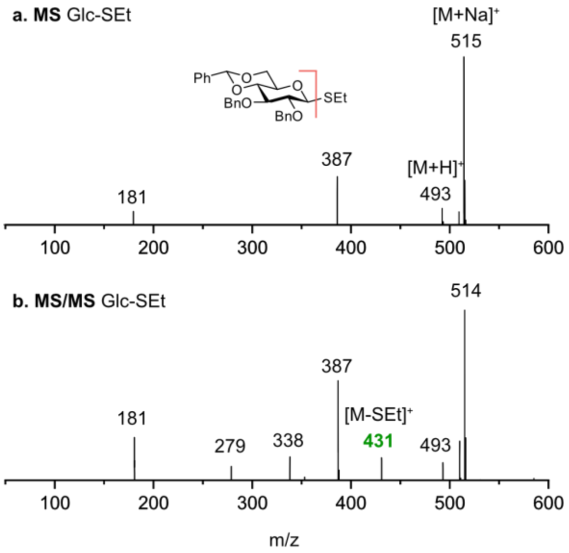

Figure S4. Mass spectra of precursors of (a) Ethyl 2,3-di-*O*-benzyl-4,6-*O*-benzylidene-D-thioglucopyramoside (**Glc-SEt**) recorded on the helium droplet instrument. (b) In-source fragmentation of precursor ions [M+H]$^+$ (*m*/*z* = 493) and [M+Na]$^+$ (*m*/*z* = 514) leads to galactosyl cations (*m*/*z* = 431).

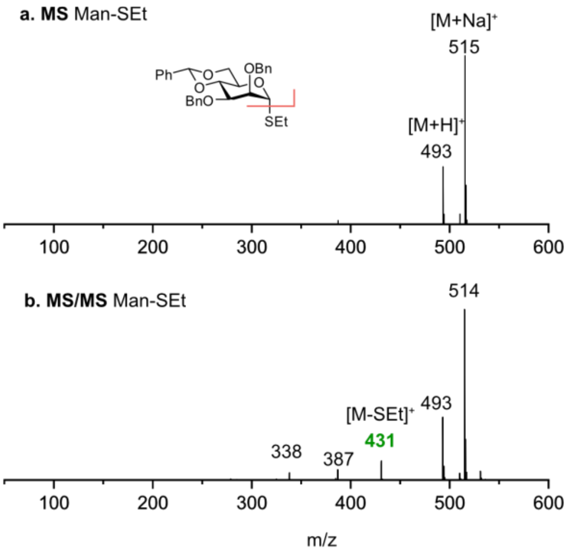

Figure S5. Mass spectra of precursors of (a) Ethyl 2,3-di-*O*-benzyl-4,6-*O*-benzylidene-D-thiomannopyramoside (**Man-SEt**) recorded on the helium droplet instrument. (b) In-source fragmentation of precursor ions [M+H]$^+$ (*m*/*z* = 493), and [M+Na]$^+$ (*m*/*z* = 514) leads to galactosyl cations (*m*/*z* = 431).

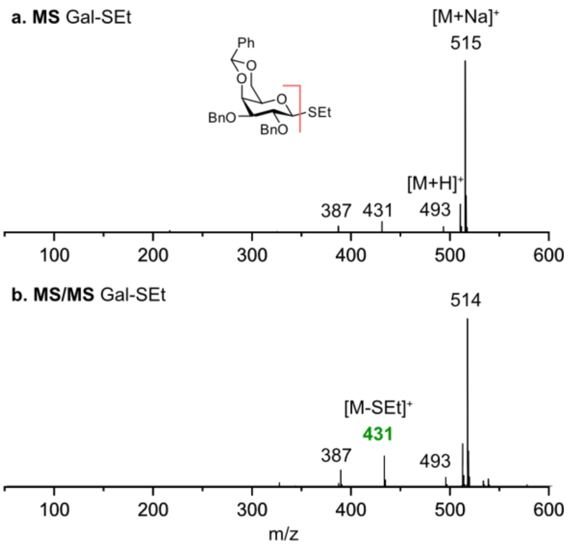

Figure S6. Mass spectra of precursors of (a) Ethyl 2,3-di-*O*-benzyl-4,6-*O*-benzylidene-D-thiogalactopyramoside (**Gal-SEt**) recorded on the helium droplet instrument. (b) In-source fragmentation of precursor ions $[M+H]^+$ (m/z = 493), and $[M+Na]^+$ (m/z = 514) leads to galactosyl cations (m/z = 431).

2. Computational Methods

2.1 Method Description

Initial geometries of glycosyl cations candidates (**Glc-oxo**, **Glc-4B**, **Glc-6B**, **Man-oxo**, **Man-4B**, **Man-6B**, **Gal-oxo**, **Gal-4B**, **Gal-6B**) were constructed by chemical intuition using GaussView 6.[3] Conformational search were performed using CREST[4] with the semiempirical method GFN2-xTB[5] using default settings. The selected structures are reoptimized and their harmonic frequencies are computed at the PBE1PBE/6-311+G(d,p) EmpiricalDispersion=GD3BJ[6,7] level of theory using Gaussian 16.[3] The calculated harmonic vibrational frequencies have been scaled with standard scaling factors 0.965. Relative free energies at 90 K (approximate temperature of the ion trap) were extracted from the frequency calculation and are represented with the energy ΔE (including zero-point vibrational energy) in the tables and figures below.

2.2 Energetics of Intermediates in Vacuum

Table S1. List of conformations of glucosyl (Glc) cations optimized at PBE1PBE/6-311+G(d,p) EmpiricalDispersion=GD3BJ level of theory. Relative energies (ΔE, including zero-point vibrational energy), and free energies (ΔF) at 90 K are assigned to each conformer. †The lowest-free energy conformer and its simulated IR spectra are showed highlighted in the manuscript or SI.

Glycosyl cations/conformer	ΔE (kJ mol^{-1})	ΔF (kJ mol^{-1})
Glc-oxo/conf_1†	72.0883535	72.80242962
Glc-oxo/conf_2	73.4116055	74.23188162
Glc-oxo/conf_3	78.4578165	77.58998814
Glc-oxo/conf_4	81.427257	82.21266148
Glc-oxo/conf_5	95.316152	93.92049379
Glc-oxo/conf_6	99.5983425	100.4582455
Glc-oxo/conf_7	102.5021455	103.2495082
Glc-oxo/conf_8	104.767952	105.0596057
Glc-6B/conf_1†	0	0
Glc-6B/conf_2	5.6894585	5.319343575
Glc-6B/conf_3	7.0809735	5.968251113
Glc-6B/conf_4	13.363795	12.04580545
Glc-6B/conf_5	17.06575	16.85572761
Glc-4B/conf_1†	28.9461375	27.58852108
Glc-4B/conf_2	35.9614735	35.44632425
Glc-4B/conf_3	38.726125	39.257125
Glc-4B/conf_4	41.79796	40.9887794
Glc-4B/conf_5	44.565237	43.41447282
Glc-4B/conf_6	50.840182	49.96046558
Glc-4B/conf_7	63.657873	63.00402972

Table S2. List of conformations of galactosyl (Gal) cations optimized at PBE1PBE/6-311+G(d,p) EmpiricalDispersion=GD3BJ level of theory. Relative energies (ΔE, including zero-point vibrational energy), and free energies (ΔF) at 90 K are assigned to each conformer. †The lowest-free energy conformer and its simulated IR spectra are showed highlighted in the manuscript or SI.

Glycosyl cations/conformer	ΔE (kJ mol^{-1})	ΔF (kJ mol^{-1})
Gal-oxo/conf_1†	29.5500025	28.78361892
Gal-oxo/conf_2	32.367164	32.04380878
Gal-oxo/conf_3	42.065761	41.62590279
Gal-oxo/conf_4	43.1605945	43.60837808
Gal-oxo/conf_5	57.7163665	58.55724859
Gal-6B/conf_1†	0	0
Gal-6B/conf_2	4.0353935	2.554933799
Gal-6B/conf_3	6.3248295	6.169492187
Gal-6B/conf_4	14.266967	12.05341028
Gal-6B/conf_5	19.554724	14.37152997
Gal-6B/conf_6	20.352876	17.75969391
Gal-6B/conf_7	21.098518	19.28440009
Gal-6B/conf_8	28.6783365	28.45563351
Gal-6B/conf_9	34.168257	33.52550924
Gal-4B/conf_1†	9.4911825	7.822098918
Gal-4B/conf_2	11.3762915	8.907537769
Gal-4B/conf_3	12.261085	10.4897641
Gal-4B/conf_4	15.936785	12.68975963
Gal-4B/conf_5	23.403707	22.19270998
Gal-4B/conf_6	26.3889005	24.36079751

Table S3. List of conformations of mannosyl (Man) cations optimized at PBE1PBE/6-311+G(d,p) EmpiricalDispersion=GD3BJ level of theory. Relative energies (ΔE, including zero-point vibrational energy), and free energies (ΔF) at 90 K are assigned to each conformer. †The lowest-free energy conformer and its simulated IR spectra are showed highlighted in the manuscript or SI.

Glycosyl cations/conformer	ΔE (kJ mol^{-1})	ΔF (kJ mol^{-1})
Man-oxo/conf_1†	72.353529	72.99548422
Man-oxo/conf_2	79.2087095	78.10311995
Man-oxo/conf_3	86.9434325	86.08352951
Man-oxo/conf_4	93.268262	94.11865454
Man-oxo/conf_5	100.7220565	100.5564162
Man-6B/conf_1†	13.153755	12.32238336
Man-6B/conf_2	24.264871	24.03820533
Man-4B/conf_1†	0	0
Man-4B/conf_2	1.3731365	2.821894709
Man-4B/conf_3	11.841005	13.50454082
Man-4B/conf_4	15.0572425	16.37126937
Man-4B/conf_5	19.0690065	20.81338113
Man-4B/conf_6	21.47659	22.33807806
Man-4B/conf_7	32.435427	32.75244193

2.3 Energy Hierarchies of Intermediates in Vacuum

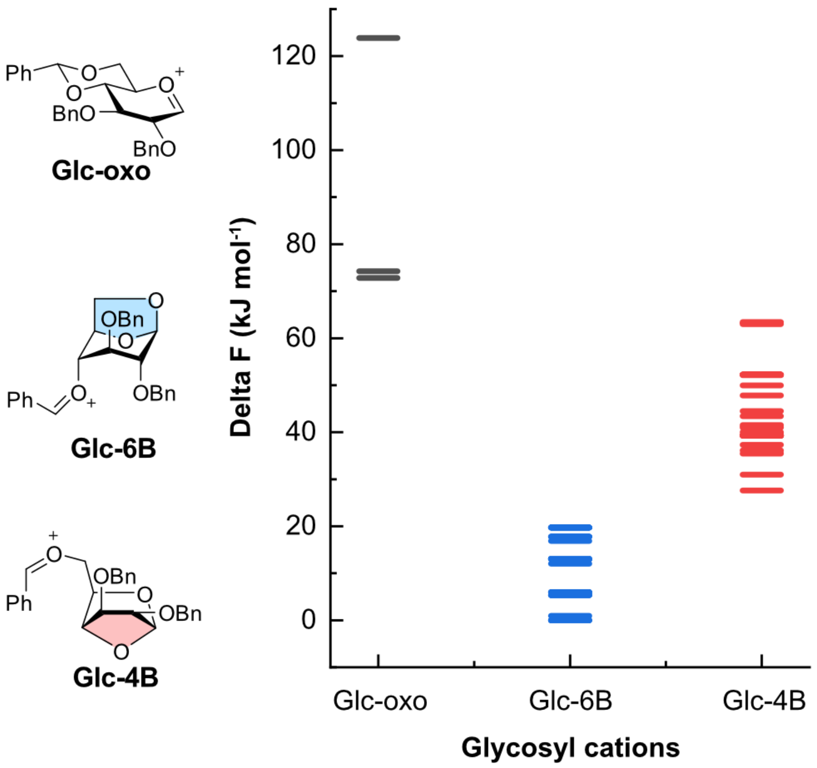

Figure S7. Energy hierarchies of the reoptimized structures for glucosyl cations.

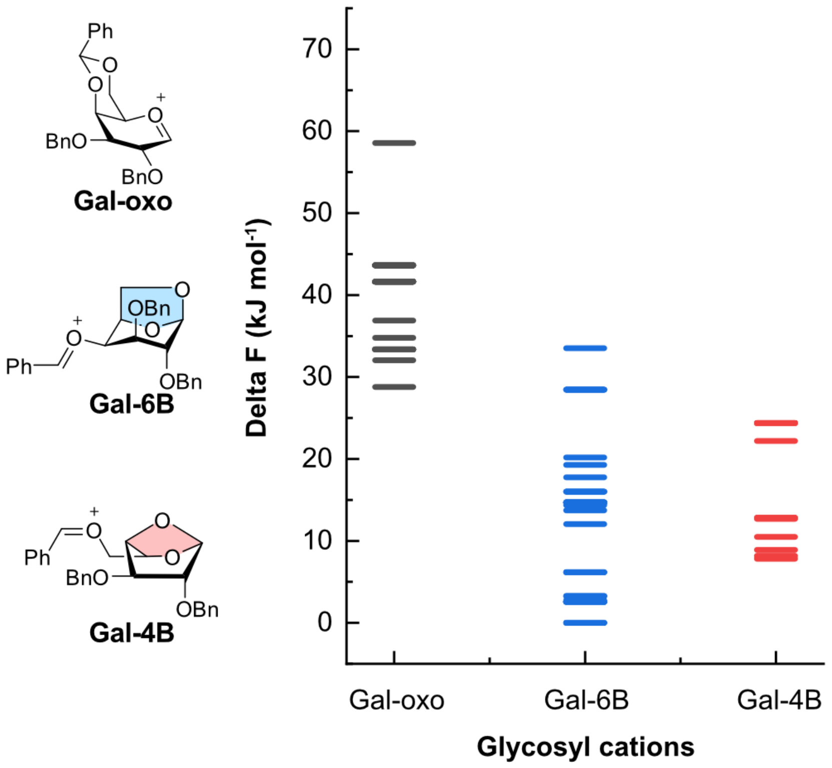

Figure S8. Energy hierarchies of the reoptimized structures for galactosyl cations.

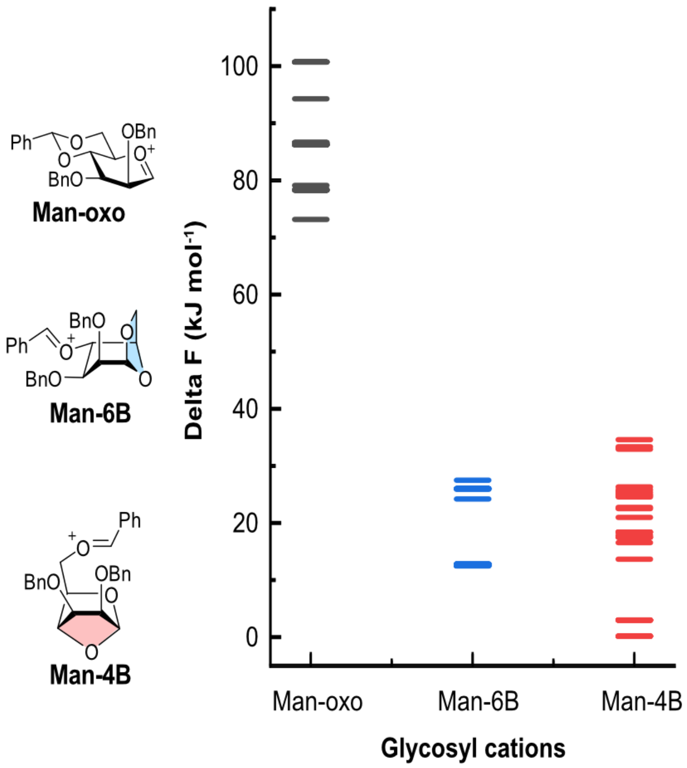

Figure S9. Energy hierarchies of the reoptimized structures for mannosyl cations.

2.4 3D Structures of Intermediates in Vacuum

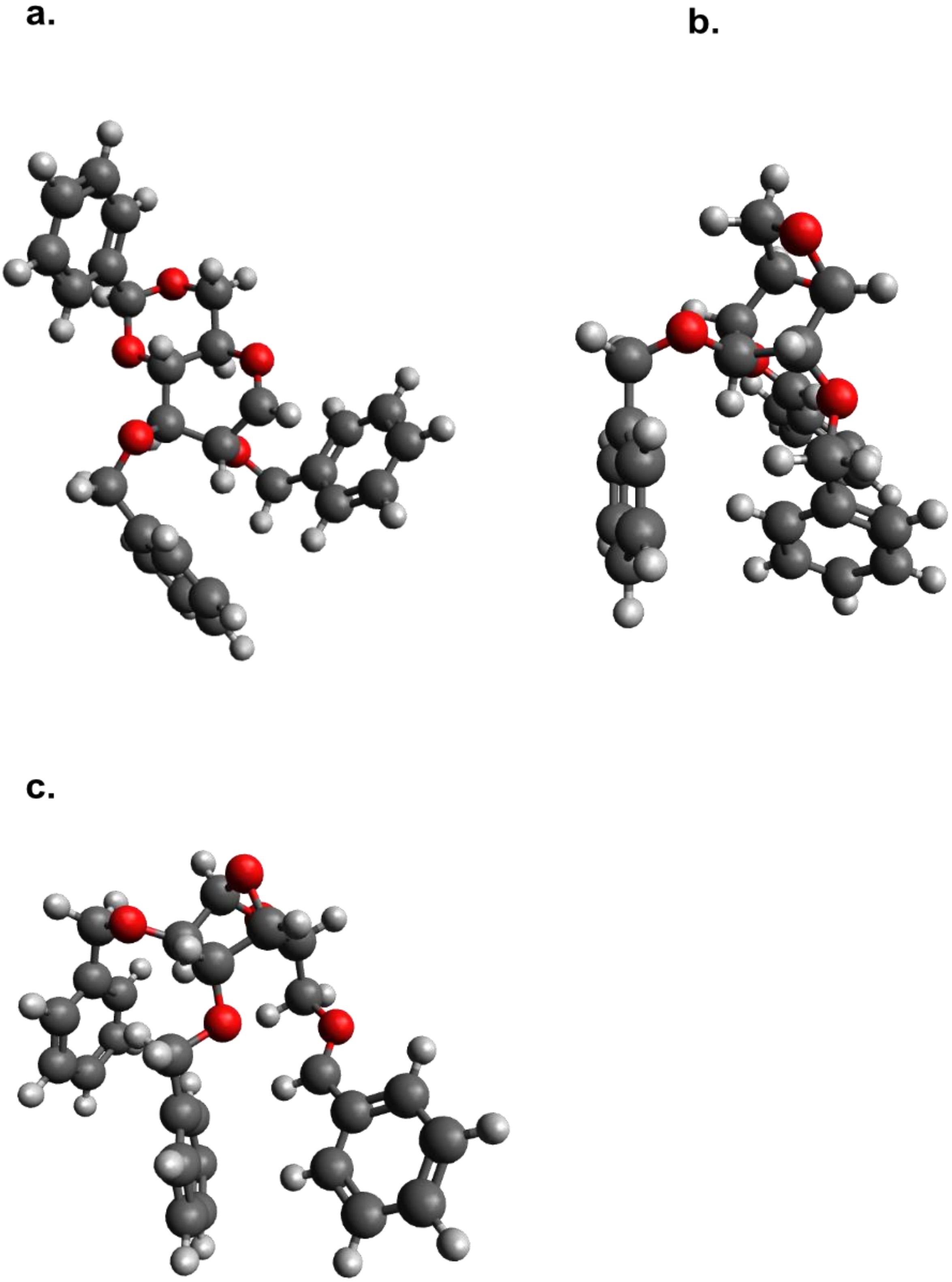

Figure S10. Reoptimized geometries of glucosyl (Glc) cations. The structures correspond to those that are highlighted with a dagger (†) in Tables S1. a) **Glc-oxo**, b) **Glc-6B**, and c) **Glc-4B**.

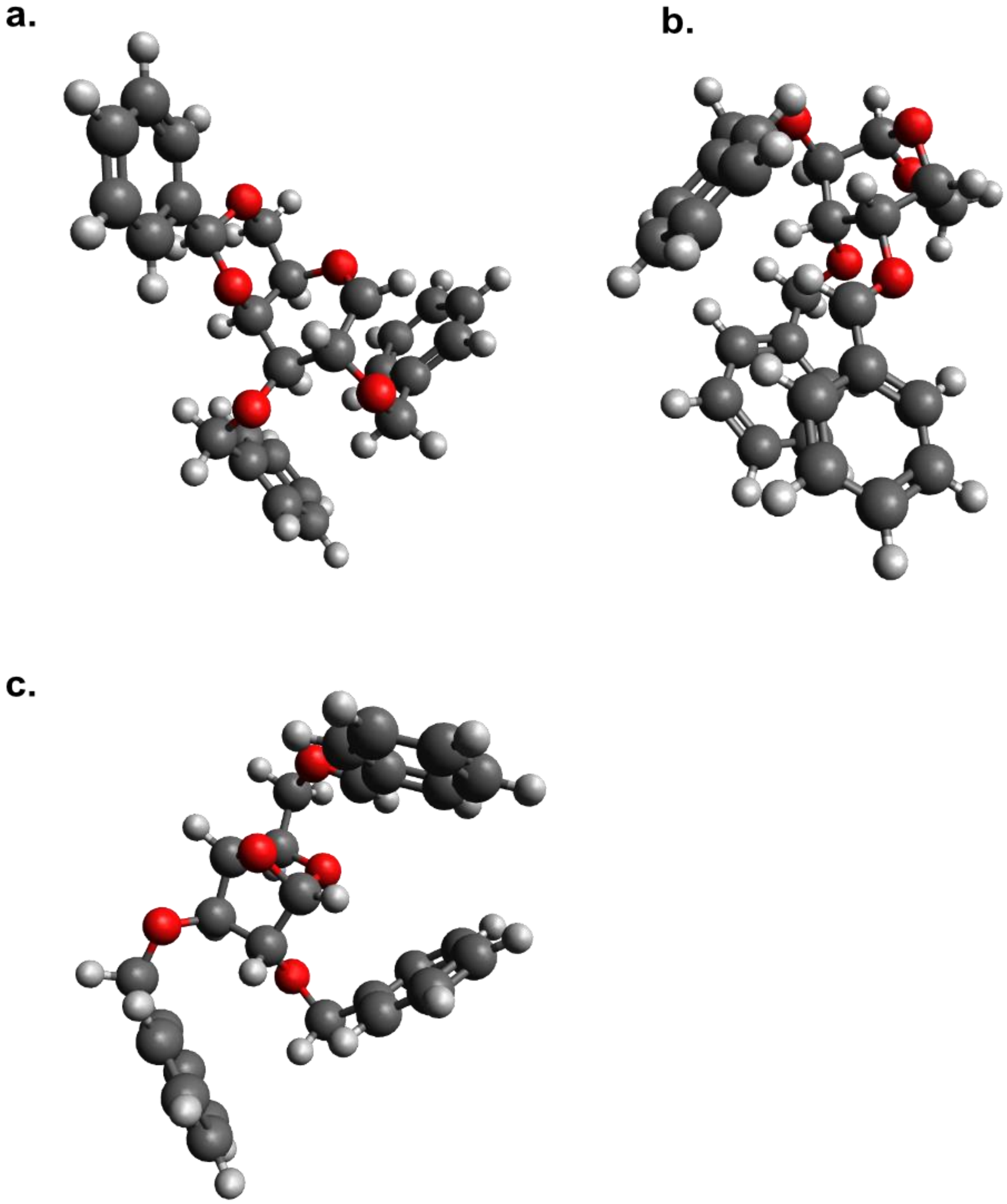

Figure S11. Reoptimized geometries of galactosyl (Gal) cations. The structures correspond to those that are highlighted with a dagger (†) in Tables S2. a) **Gal-oxo**, b) **Gal-6B**, and c) **Gal-4B**.

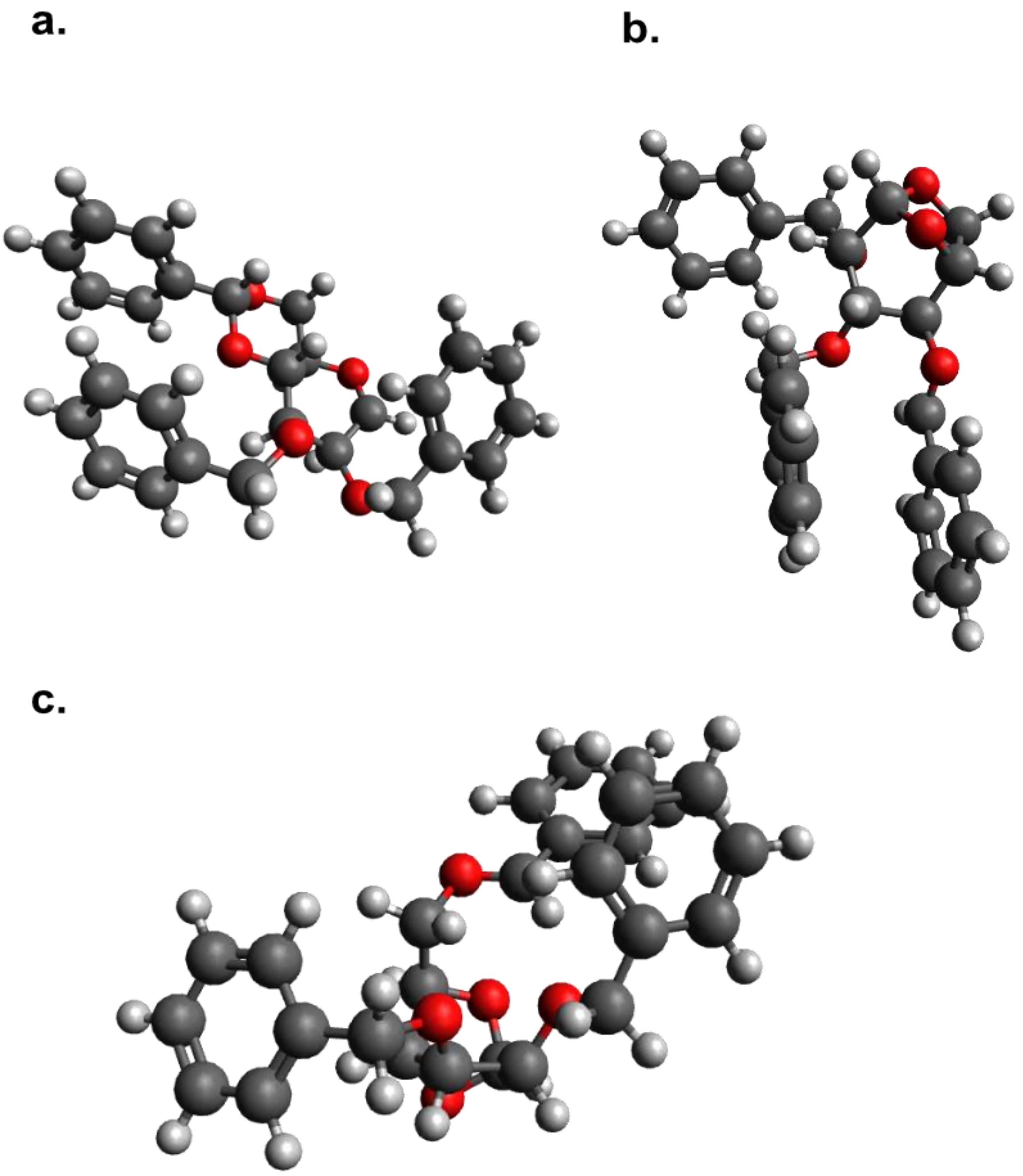

Figure S12. Reoptimized geometries of mannosyl (Man) cations. The structures correspond to those that are highlighted with a dagger (†) in Tables S3. a) **Man-oxo**, b) **Man-6B**, and c) **Man-4B**.

2.5 Structure Validation: Comparative Analysis Using Four Additional Computational Methods

To evaluate the significance of various structures, four additional computational methods are employed: Method 1 (wB97XD/def2TZVP), Method 2 (M062X/cc-pvtz), Method 3 (M062X/def2TZVP), and Method 4 (PBE1PBE/def2TZVP EmpiricalDispersion=GD3BJ). Our findings indicate that anhydro cations consistently exhibit thermodynamic stability, showing the lowest free energy at 90 K compared to oxocarbenium ions. The calculated harmonic vibrational frequencies have been scaled with standard scaling factors 0.965. Furthermore, computational infrared (IR) spectra generated using each method closely resemble those obtained with PBE1PBE/6-311+G(d,p) EmpiricalDispersion=GD3BJ level. This strengthens the assertion that anhydro cations are formed and observable in cold-IR experiments.

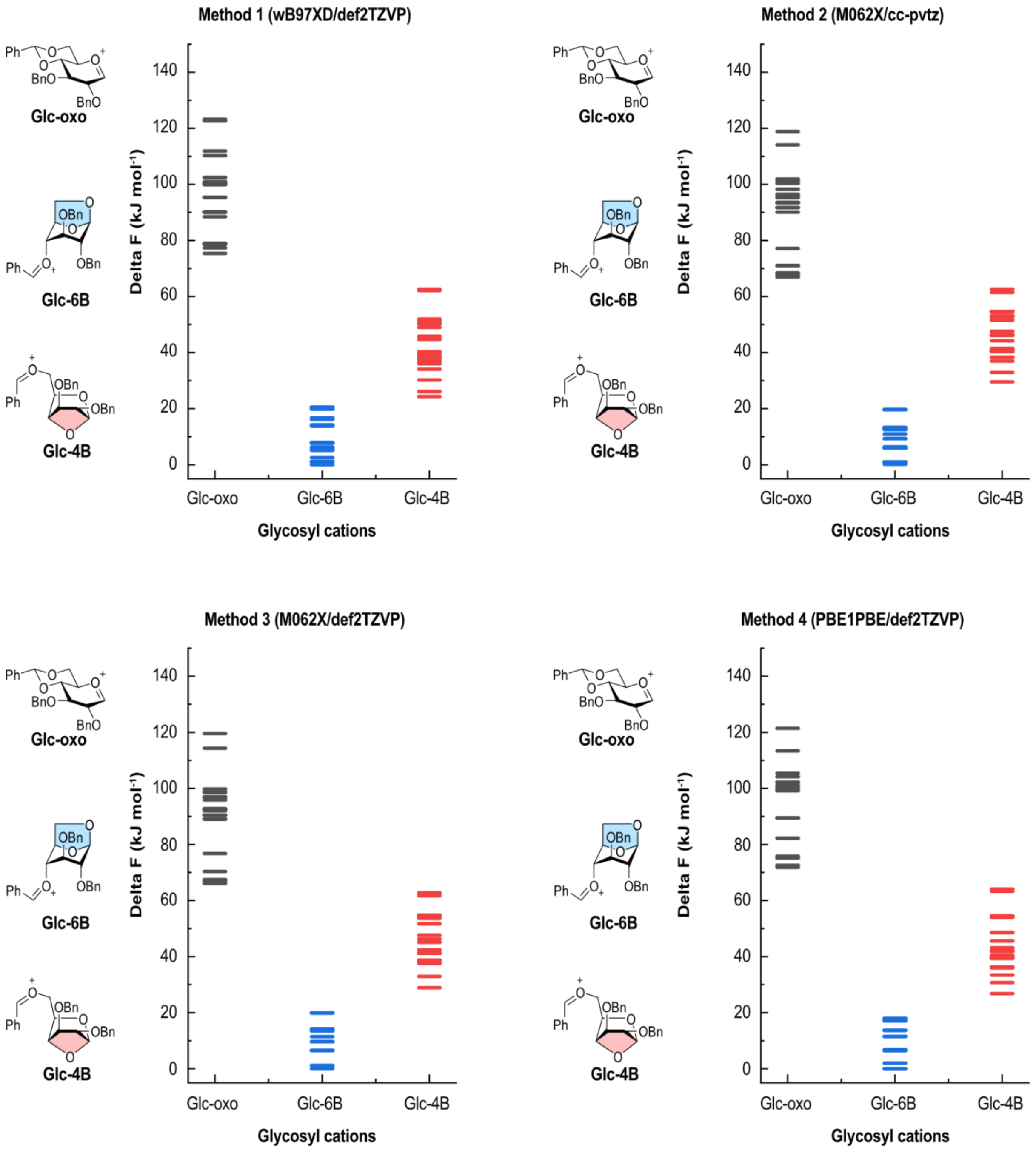

Figure S13. Energy hierarchies of the reoptimized structures for glucosyl cations. a) Method 1 (wB97XD/def2TZVP). b) Method 2 (M062X/cc-pvtz). C) Method 3 (M062X/def2TZVP). d) Method 4 (PBE1PBE/def2TZVP). The distribution of free energy is consistent across all these methods. The data suggests that **Glc-6B** is the most thermodynamically stable intermediate.

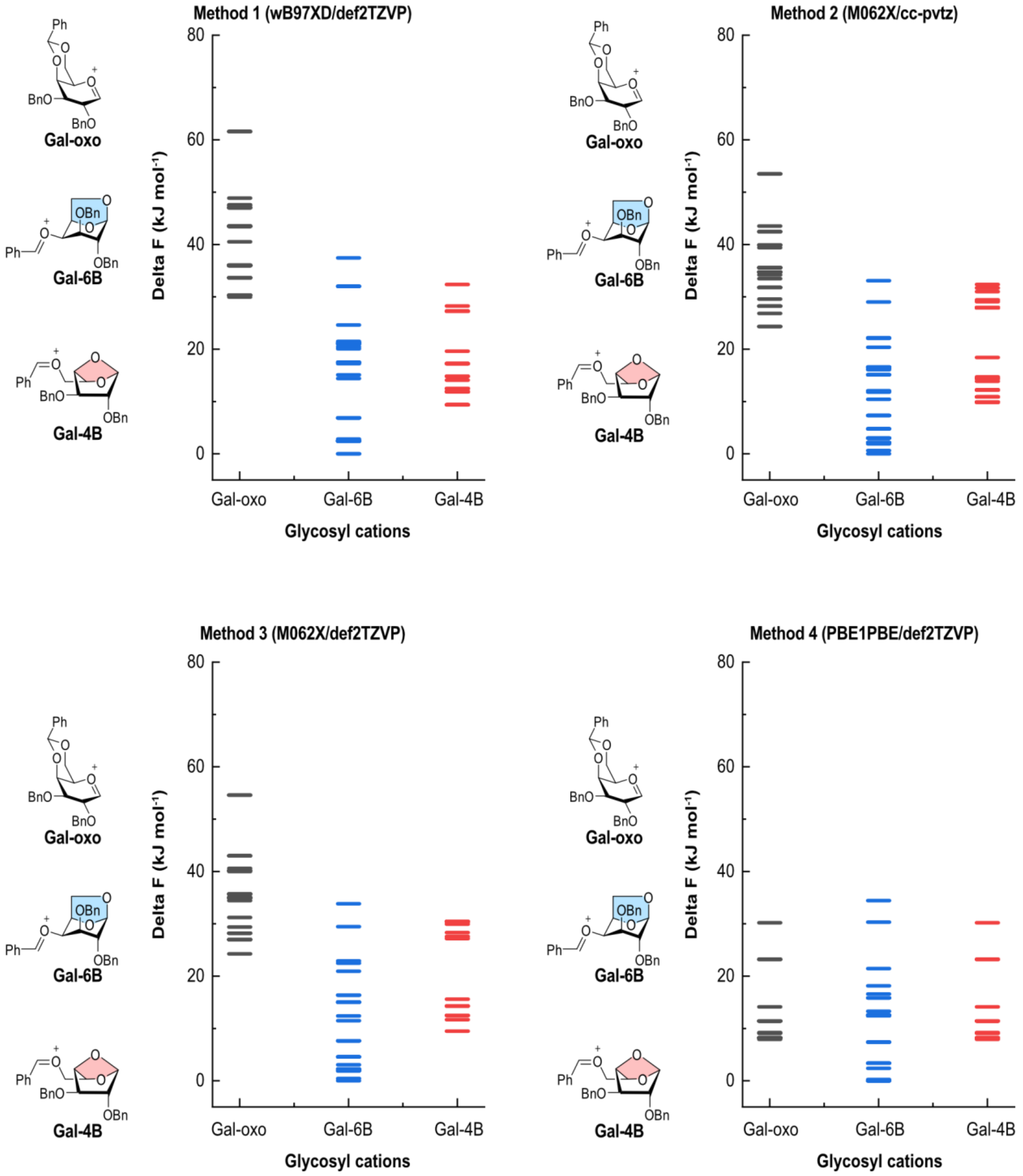

Figure S14. Energy hierarchies of the reoptimized structures for Galactosyl cations. a) Method 1 (wB97XD/def2TZVP). b) Method 2 (M062X/cc-pvtz). C) Method 3 (M062X/def2TZVP). d) Method 4 (PBE1PBE/def2TZVP). The distribution of free energy is consistent across all these methods. The data suggests that **Gal-6B** and **Gal-4B** are much more thermodynamic stable, compared to oxocarbenium ion (**Gal-oxo**).

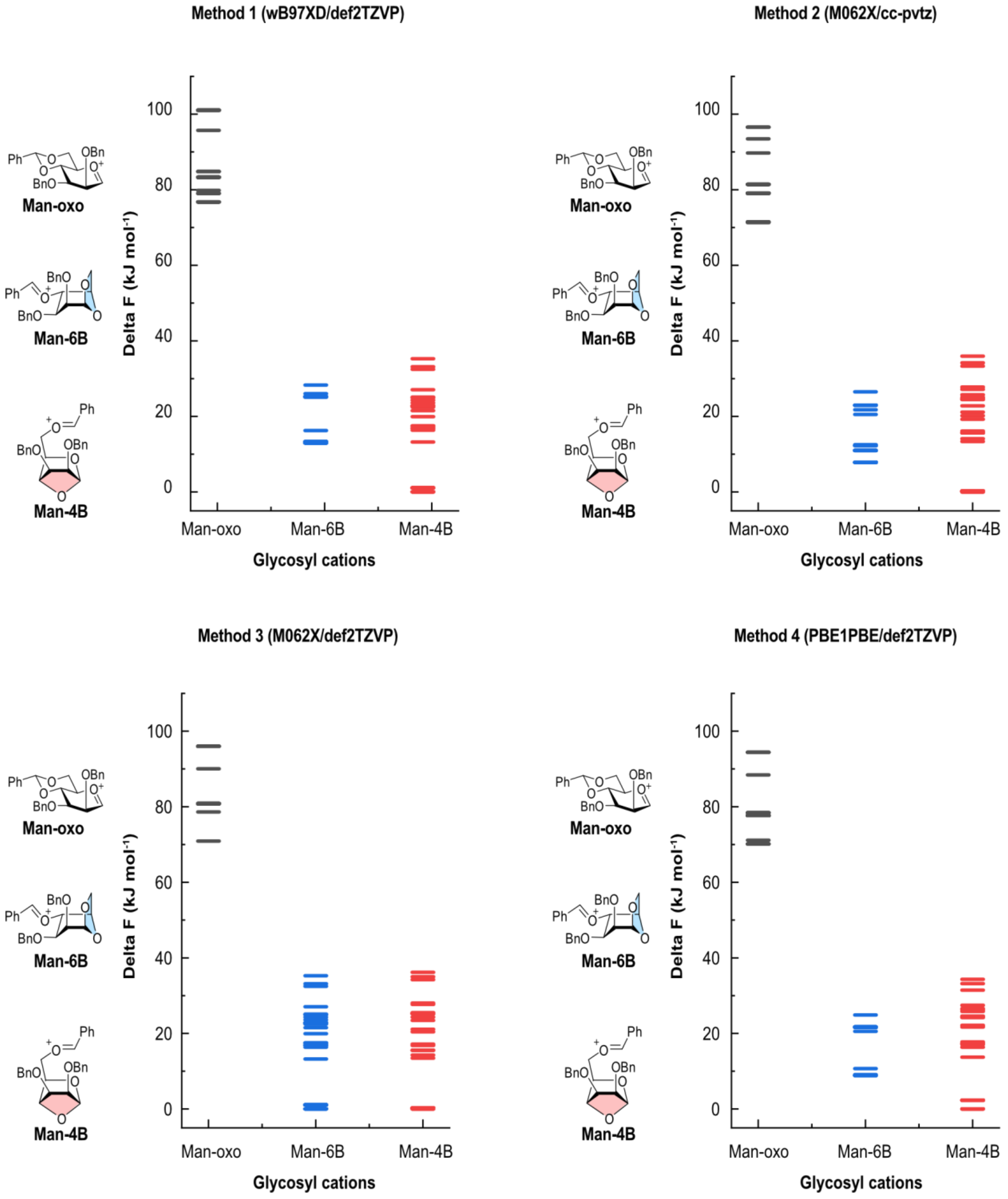

Figure S15. Energy hierarchies of the reoptimized structures for Mannosyl cations. a) Method 1 (wB97XD/def2TZVP). b) Method 2 (M062X/cc-pvtz). C) Method 3 (M062X/def2TZVP). d) Method 4 (PBE1PBE/def2TZVP). The distribution of free energy is consistent across all these methods. The data suggests that **Man-6B** and **Man-4B** are much thermodynamic stable, comparing to oxocarbenium ion (**Man-oxo**).

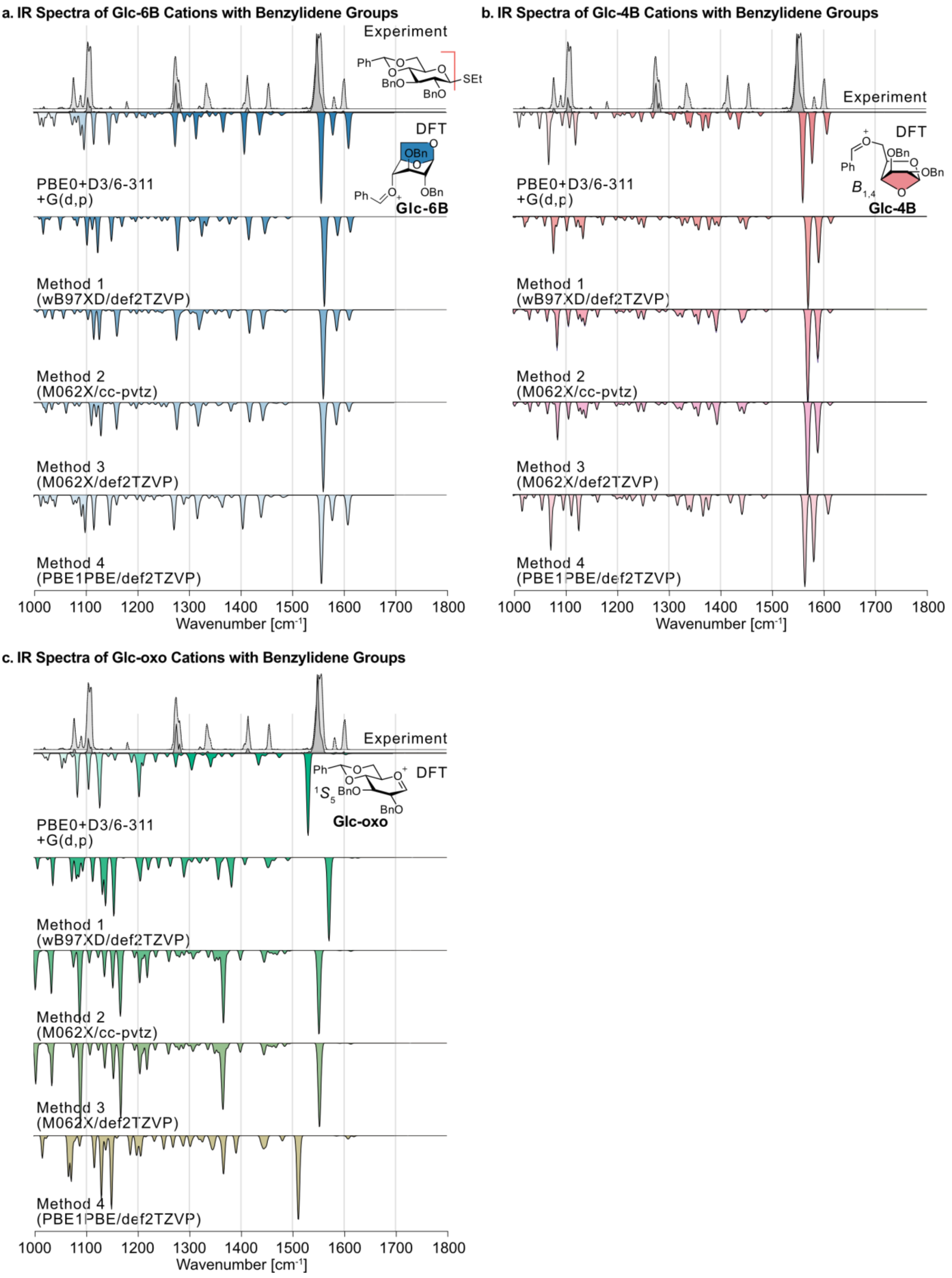

Figure S16. Comparison of frequencies and structures of a) Glc-6B, b) Glc-4B and c) Glc-oxo across various levels of theory. Experimental IR spectra are depicted as gray traces, while computed spectra of the most stable conformer are shown as inverted traces. The results suggest that Glc-6B is the most promising structure in the experimental IR spectra.

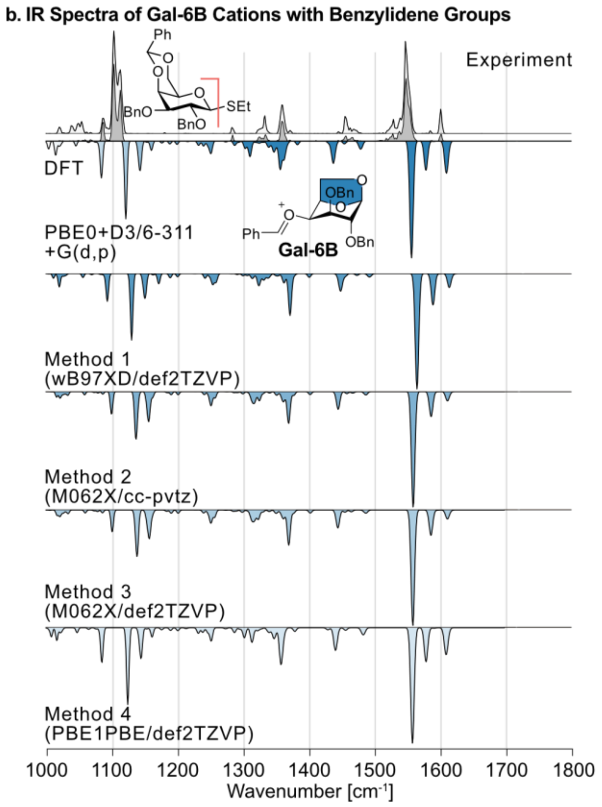

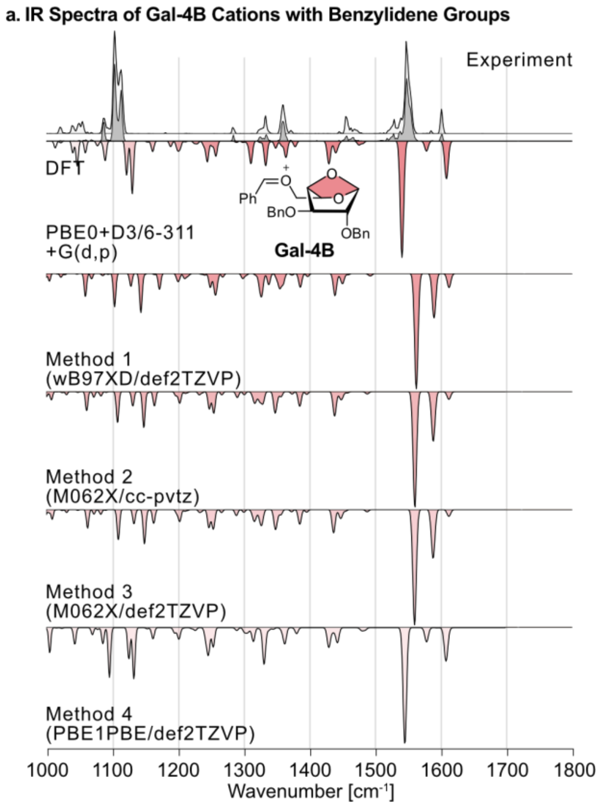

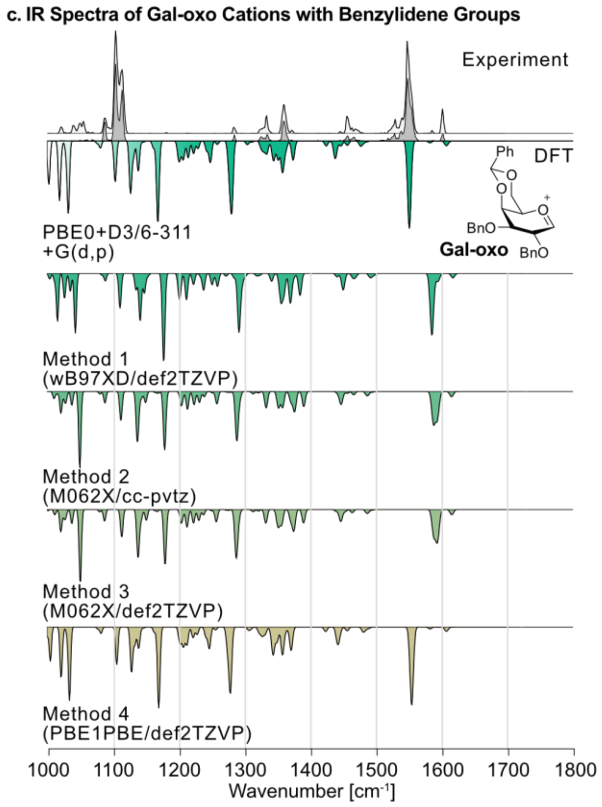

Figure S17. Comparison of frequencies and structures of a) Gal-6B, b) Gal-4B and c) Gal-oxo across various levels of theory. Experimental IR spectra are depicted as gray traces, while computed spectra of the most stable conformer are shown as inverted traces. The results suggest that Gal-6B and Gal-4B are the most promising structure in the experimental IR spectra.

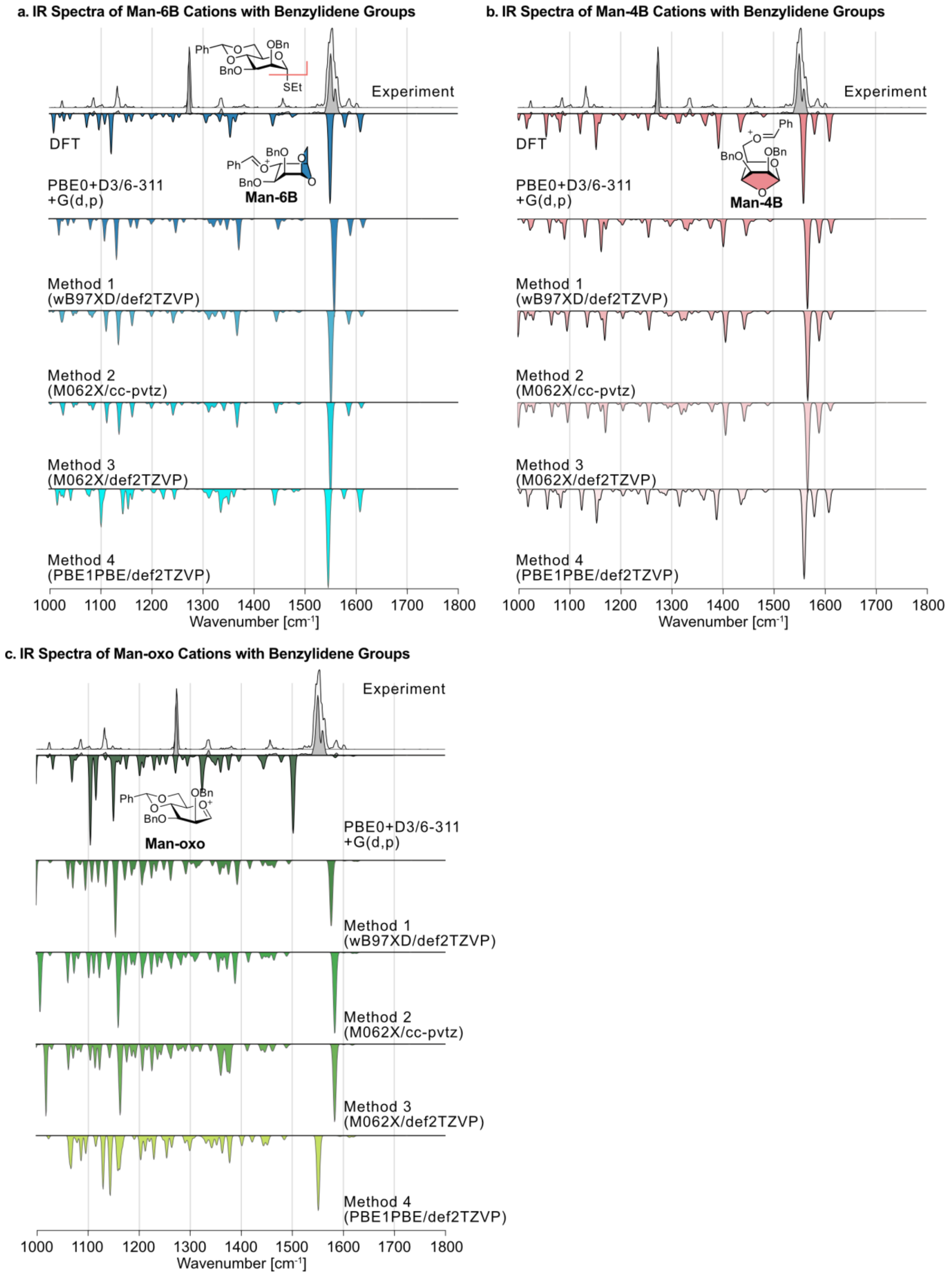

Figure S18. Comparison of frequencies and structures of a) Man-6B, b) Man-4B and c) Man-oxo across various levels of theory. Experimental IR spectra are depicted as gray traces, while computed spectra of the most stable conformer are shown as inverted traces. The results suggest that Man-6B and Man-4B is the most promising structure in the experimental IR spectra.

2.6 Energetics of Intermediates Using a Solvent Model

Table S4. List of the most stable conformer of glycosyl cations (**Glc-4B** and **Glc-6B**) optimized at the PBE0+D3/6-311+G(d,p) level of theory using the COSMO solvation model[8,9] for various solvents with distinct relative permittivities (εr). Relative free energies (ΔF) at 298 K are assigned to each conformer and compared to the most stable structure of **Glc-oxo** (ΔF = 0) in the corresponding solvent system. The corresponding structures of are shown in the SI.

**Structure**	**Solvent**								
	Vacuum	**Dioxane**	**Toluene**	**Et_2O**	**$CHCl_3$**	**DCM**	**ACN**	**DMSO**	**H_2O**
**Glc-4B**	-50.0053	-42.2390	-42.9427	-45.2662	-48.1989	-47.8366	-47.4113	-48.7057	-44.3027
**Glc-6B**	-74.4539	-69.4707	-69.1583	-70.4159	-70.4842	-65.2463	-62.3162	-64.1646	-59.6802

Table S5. List of the most stable conformer of mannosyl cations (**Man-4B** and **Man-6B**) optimized at the PBE0+D3/6-311+G(d,p) level of theory using the COSMO solvation model[8,9] for various solvents with distinct relative permittivities (εr). Relative free energies (ΔF) at 298 K are assigned to each conformer and compared to the most stable structure of **Man-oxo** (ΔF = 0) in the corresponding solvent system. The corresponding structures of are shown in the SI.

**Structure**	**Solvent**								
	Vacuum	**Dioxane**	**Toluene**	**Et_2O**	**$CHCl_3$**	**DCM**	**ACN**	**DMSO**	**H_2O**
**Man-4B**	-74.4802	-58.0052	-63.1932	-66.5853	-64.7816	-61.8568	-60.4285	-59.8640	-59.7721
**Man-6B**	-64.0806	-65.3776	-65.9342	-61.4813	-59.4492	-57.6770	-59.5647	-56.1069	-55.7026

Table S6. List of the most stable conformer of galactosyl cations (**Gal-4B** and **Gal-6B**) optimized at the PBE0+D3/6-311+G(d,p) level of theory using the COSMO solvation model[8,9] for various solvents with distinct relative permittivities (εr). Relative free energies (ΔF) at 298 K are assigned to each conformer and compared to the most stable structure of **Gal-oxo** (ΔF = 0) in the corresponding solvent system. The corresponding structures of are shown in the SI.

**Structure**	**Solvent**								
	Vacuum	**Dioxane**	**Toluene**	**Et_2O**	**$CHCl_3$**	**DCM**	**ACN**	**DMSO**	**H_2O**
**Gal-4B**	-23.8185	-19.0585	-23.3381	-22.1251	-20.9856	-24.3961	-19.0086	-22.6318	-21.9781
**Gal-6B**	-27.8802	-23.9131	-28.9488	-25.9452	-25.1943	-25.8953	-22.6974	-26.8169	-26.0476

2.7 Energy Hierarchies of Intermediates Using a Solvent Model

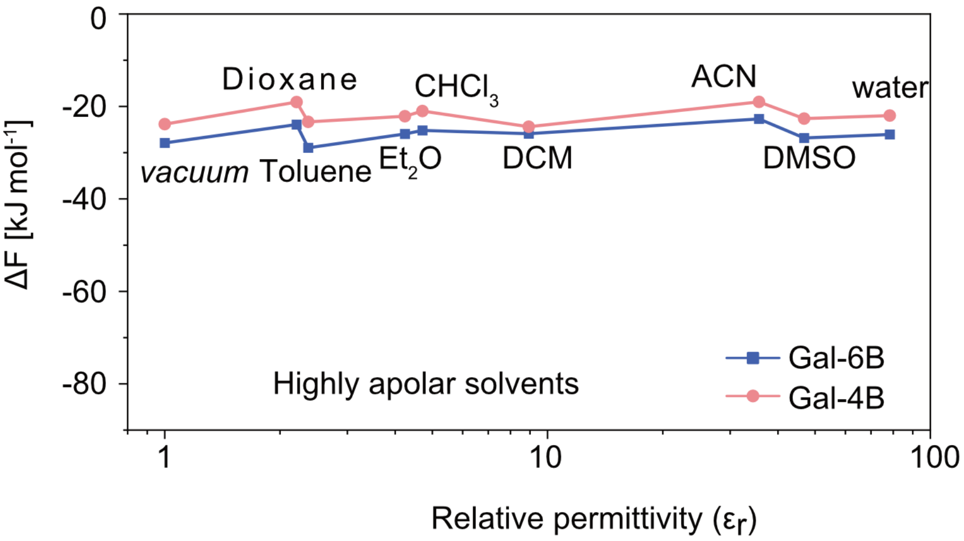

Figure S19. Relative stabilities of rearranged galactosyl cations using a solvent model. The free energies of the most stable conformers of **Gal-6B** (blue) and **Gal-4B** (red) were calculated with a solvent model for different solvents relative to **Gal-oxo** (ΔF = 0). **Gal-6B** and **Gal-4B** are similarly thermodynamically stabilized for different solvents.

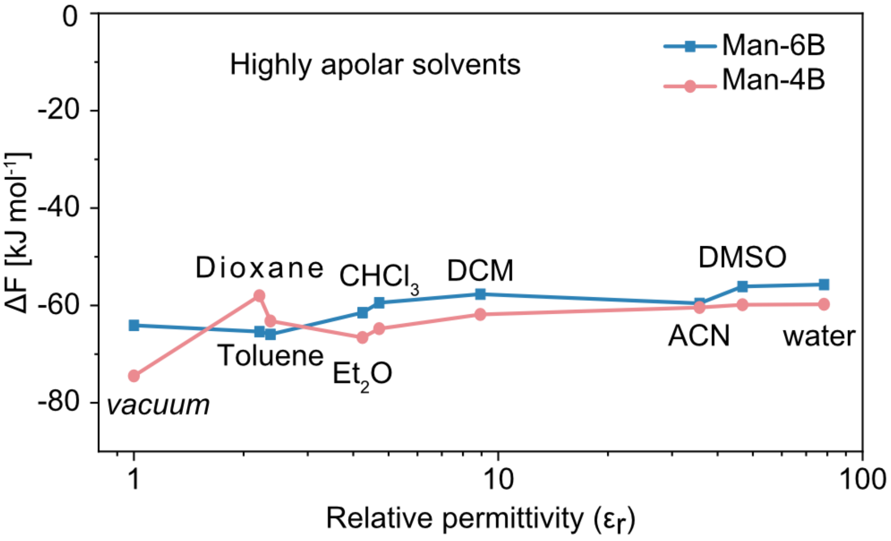

Figure S20. Relative stabilities of rearranged galactosyl cations using a solvent model. The free energies of the most stable conformers of **Man-6B** (blue) and **Man-4B** (red) were calculated with a solvent model for different solvents relative to **Man-oxo** (ΔF = 0). **Man-6B** and **Man-4B** are similarly thermodynamically stabilized for different solvents.

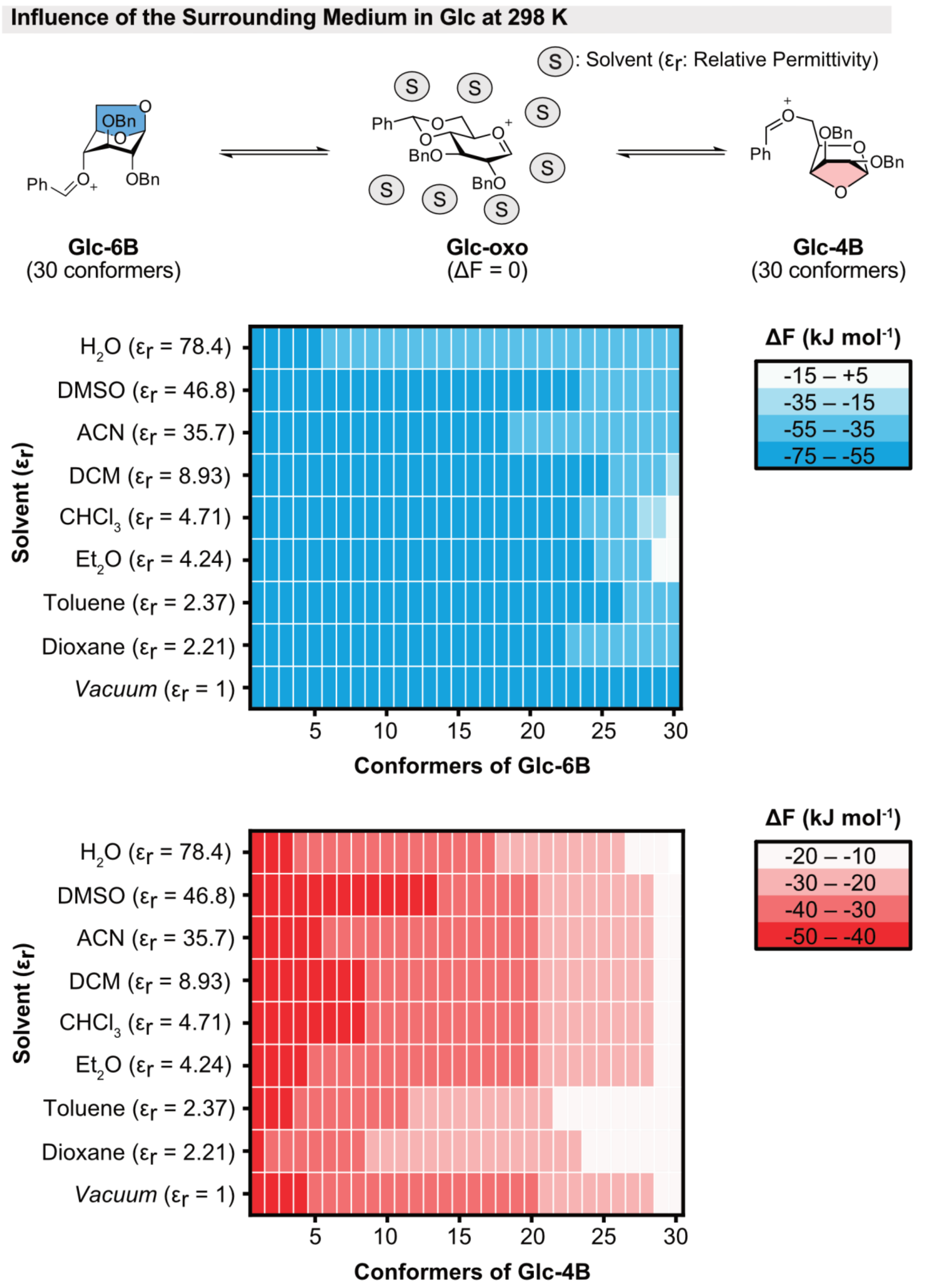

Figure S21. 30 conformers of **Glc-6B** (blue) and **Glc-4B** (red) were optimized with a solvent model for different solvents and their relative energies of each conformer relative to **Glc-oxo** are shown.

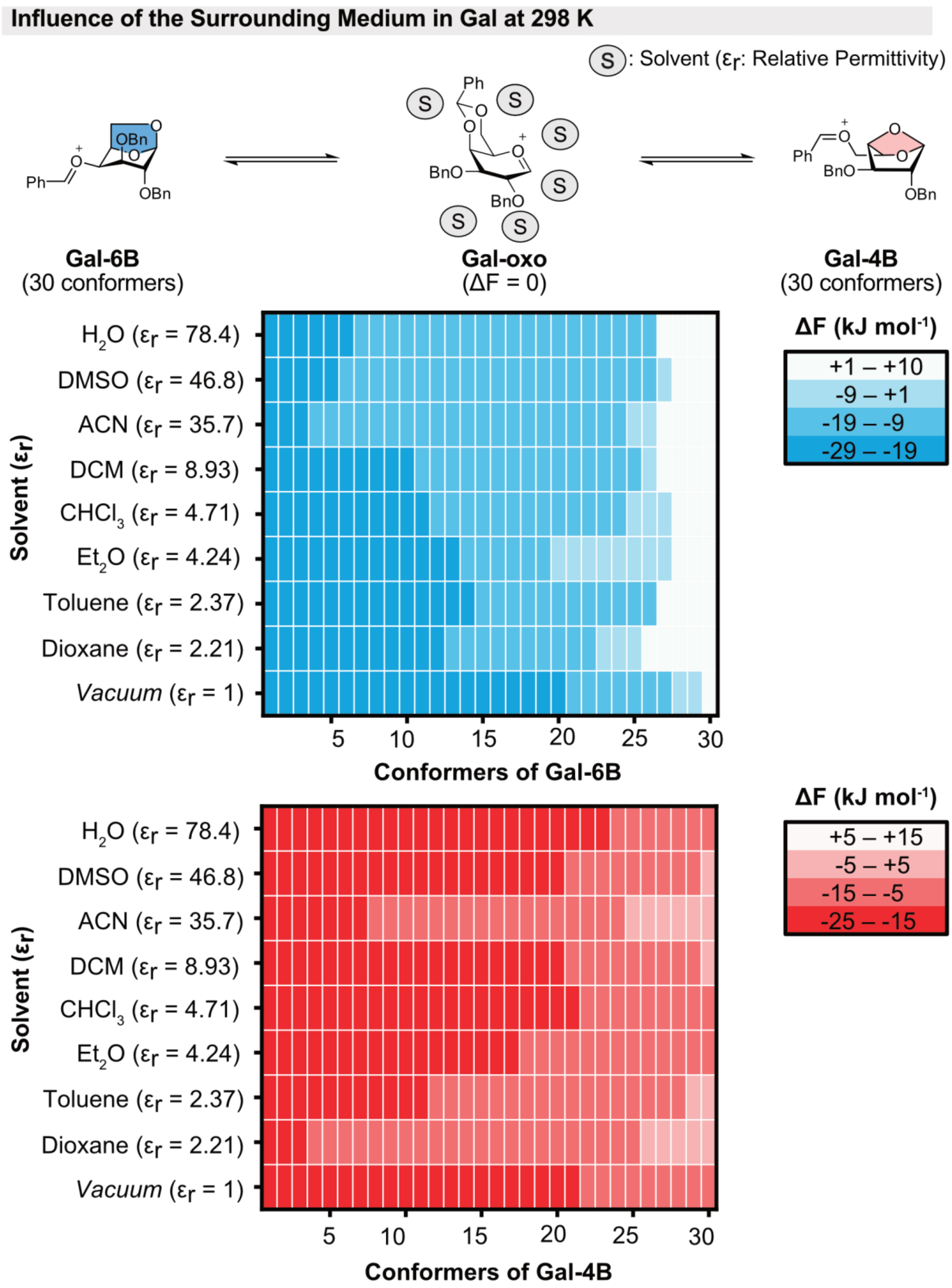

Figure S22. 30 conformers of **Gal-6B** (blue) and **Gal-4B** (red) were calculated with a solvent model for different solvents, and the relative energies of each conformer against **Gal-oxo** are shown.

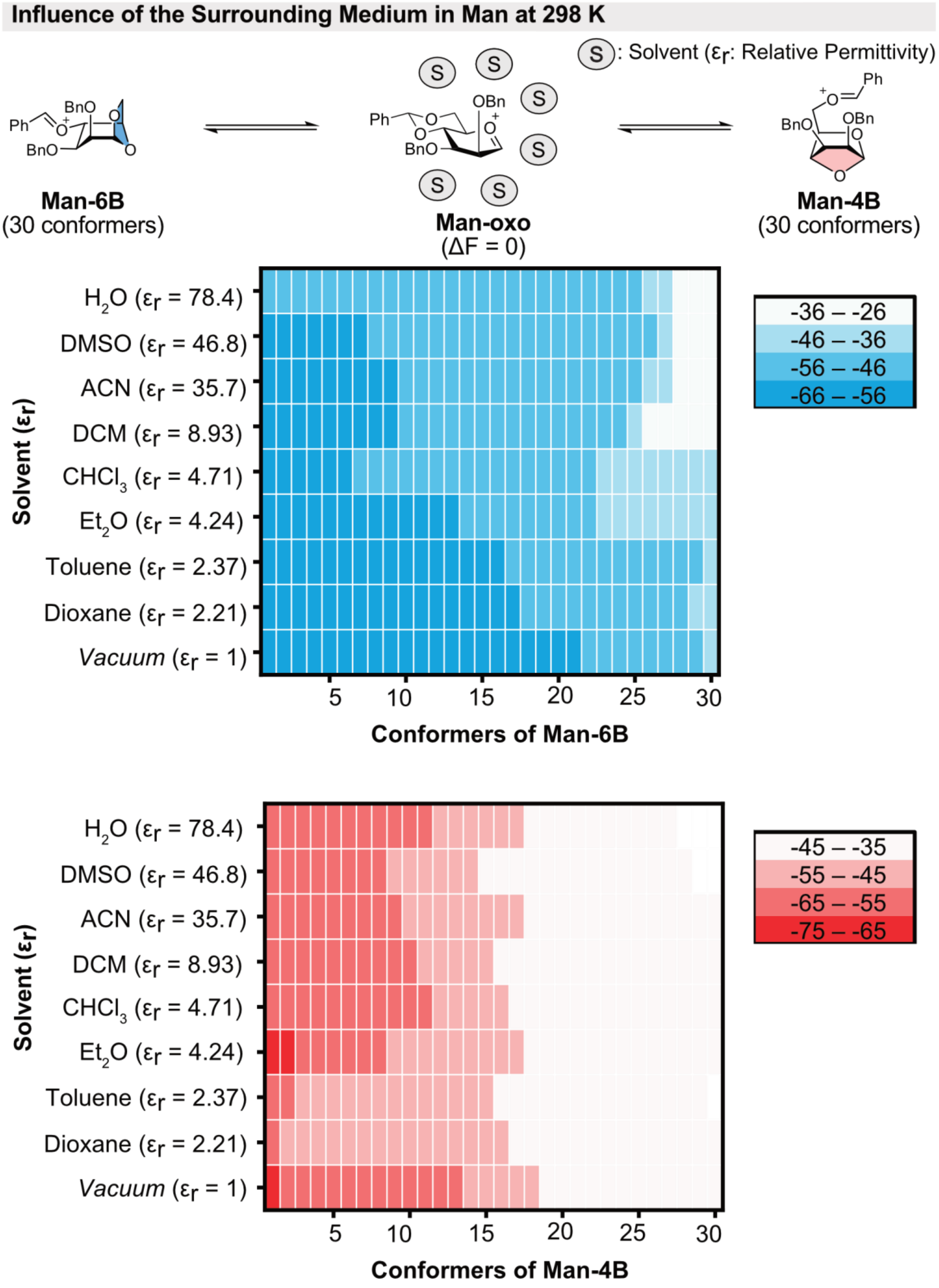

Figure S23. 30 conformers of **Man-6B** (blue) and **Man-4B** (red) were calculated with a solvent model for different solvents and their relative energies of each conformer against **Man-oxo** are shown.

2.8 3D Structures of Intermediates in Different Solvents

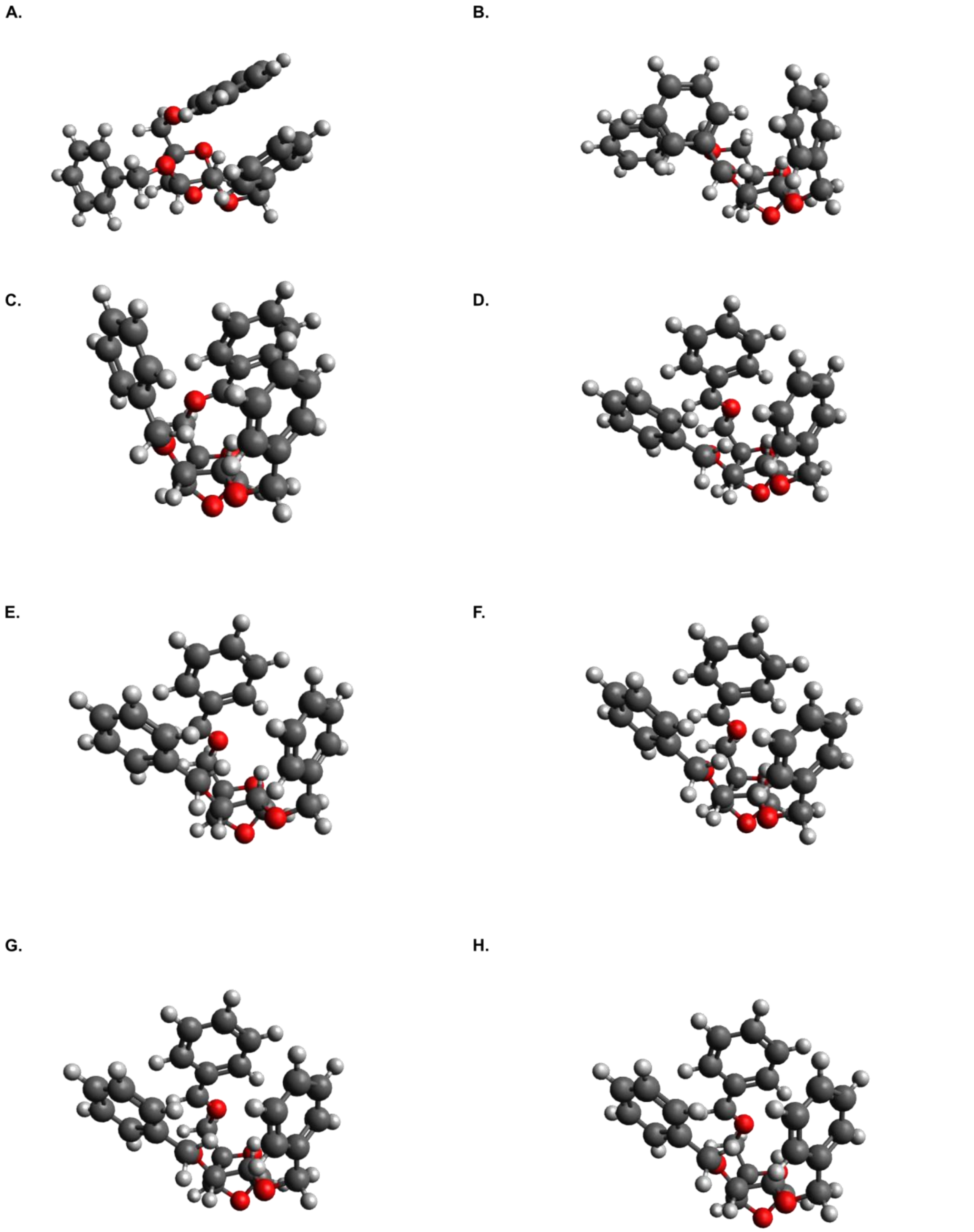

Figure S24. Reoptimized geometries of **Glc-4B** using a solvent model with different solvents. The structures correspond to those that are highlighted in entry 1, Table S4. A) 1,4-Dioxane. B) Toluene. C) Et_2O. D) $CHCl_3$. E) DCM. F) ACN. G) DMSO. H) Water.

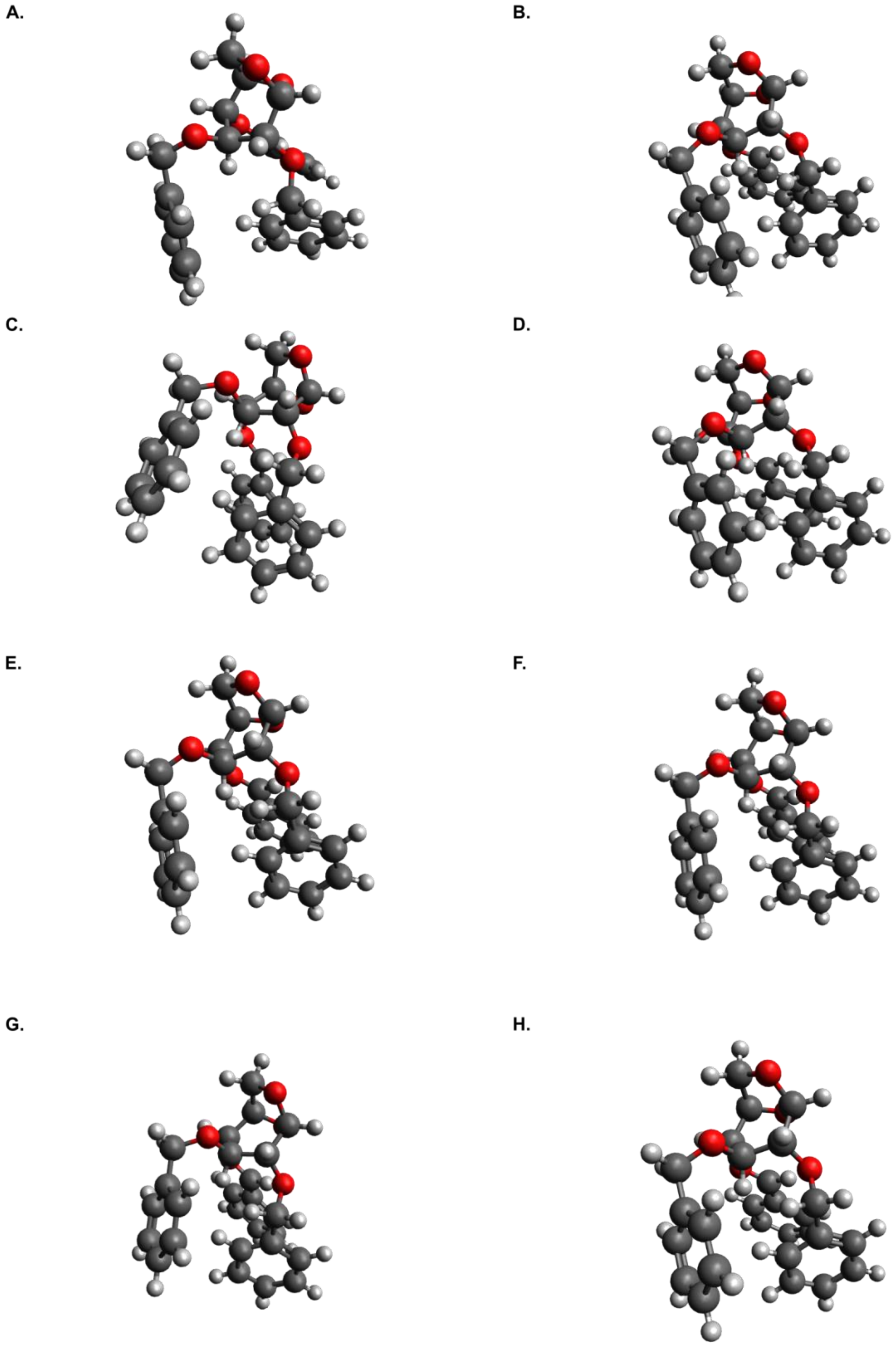

Figure S25. Reoptimized geometries of **Glc-6B** within different surrounding medias (solvents). The structures correspond to those that are highlighted in entry 1, Table S4. A) 1,4-Dioxane. B) Toluene. C) Et_2O. D) $CHCl_3$. E) DCM. F) ACN. G) DMSO. H) Water.

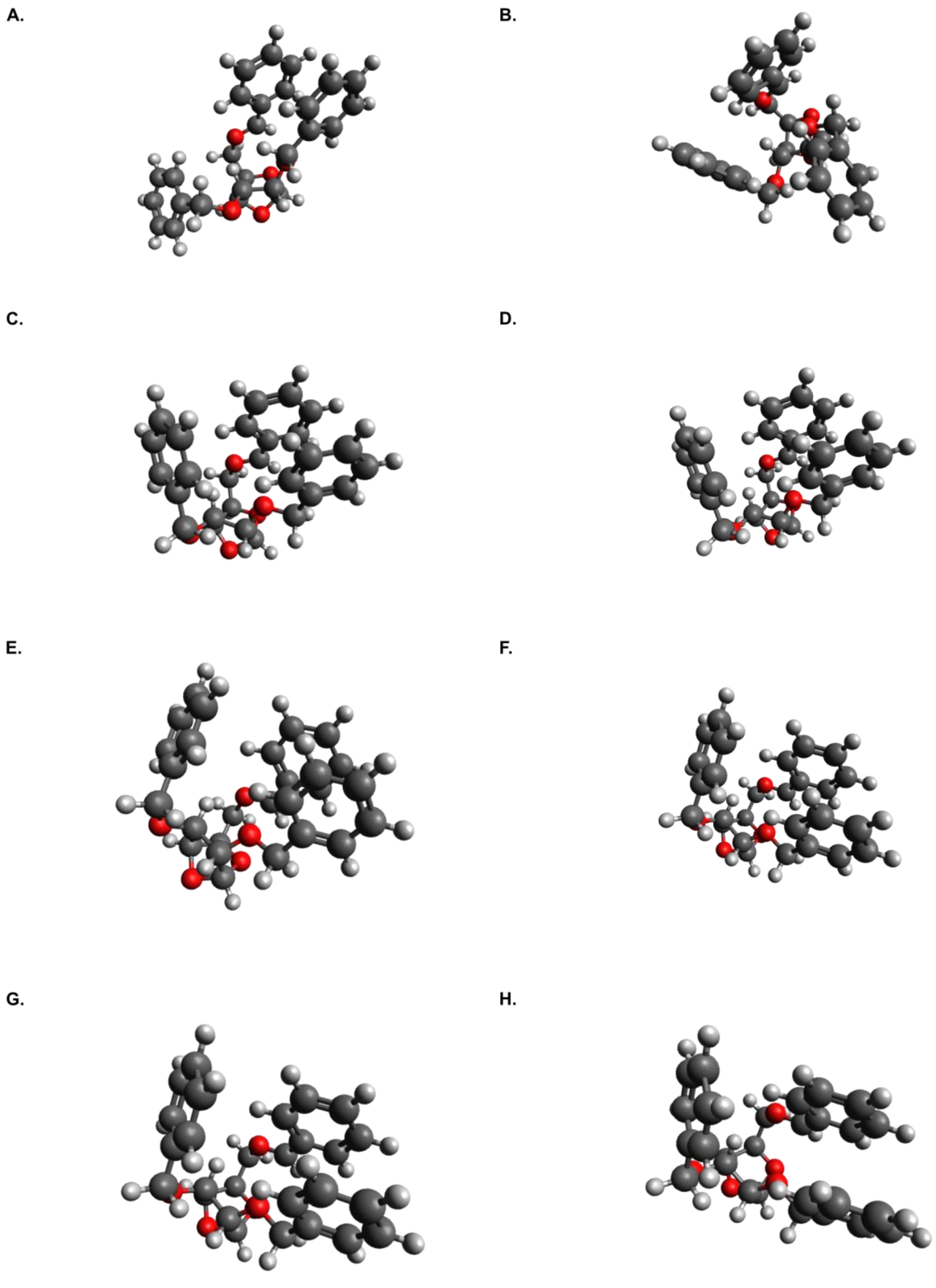

Figure S26. Reoptimized geometries of **Man-4B** within different surrounding medias (solvents). The structures correspond to those that are highlighted in entry 1, Table S5. A) 1,4-Dioxane. B) Toluene. C) Et_2O. D) $CHCl_3$. E) DCM. F) ACN. G) DMSO. H) Water.

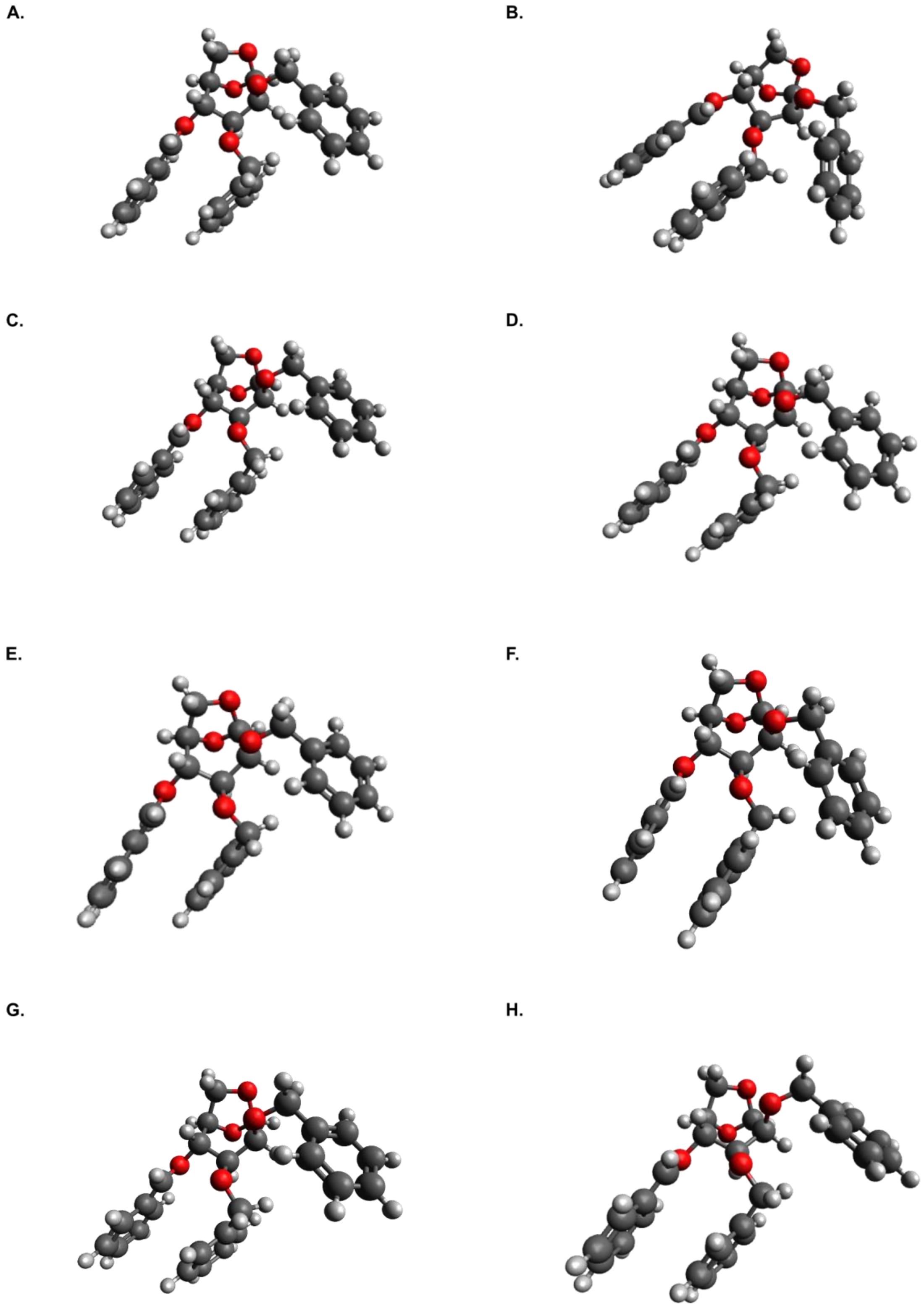

Figure S27. Reoptimized geometries of **Man-6B** within different surrounding medias (solvents). The structures correspond to those that are highlighted in entry 1, Table S5. A) 1,4-Dioxane. B) Toluene. C) Et_2O. D) $CHCl_3$. E) DCM. F) ACN. G) DMSO. H) Water.

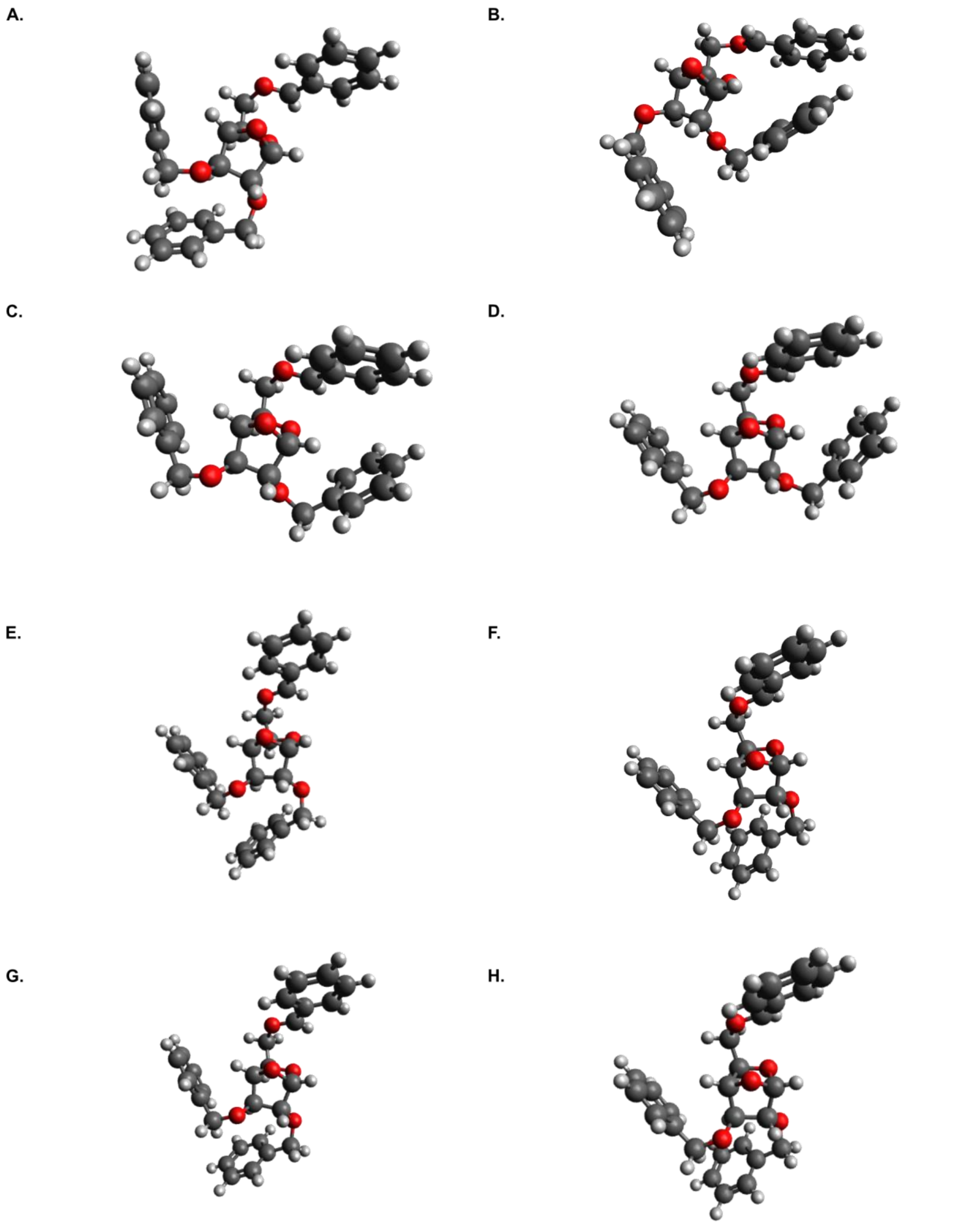

Figure S28. Reoptimized geometries of **Gal-4B** within different surrounding medias (solvents). The structures correspond to those that are highlighted in entry 1, Table S6. A) 1,4-Dioxane. B) Toluene. C) Et_2O. D) $CHCl_3$. E) DCM. F) ACN. G) DMSO. H) Water.

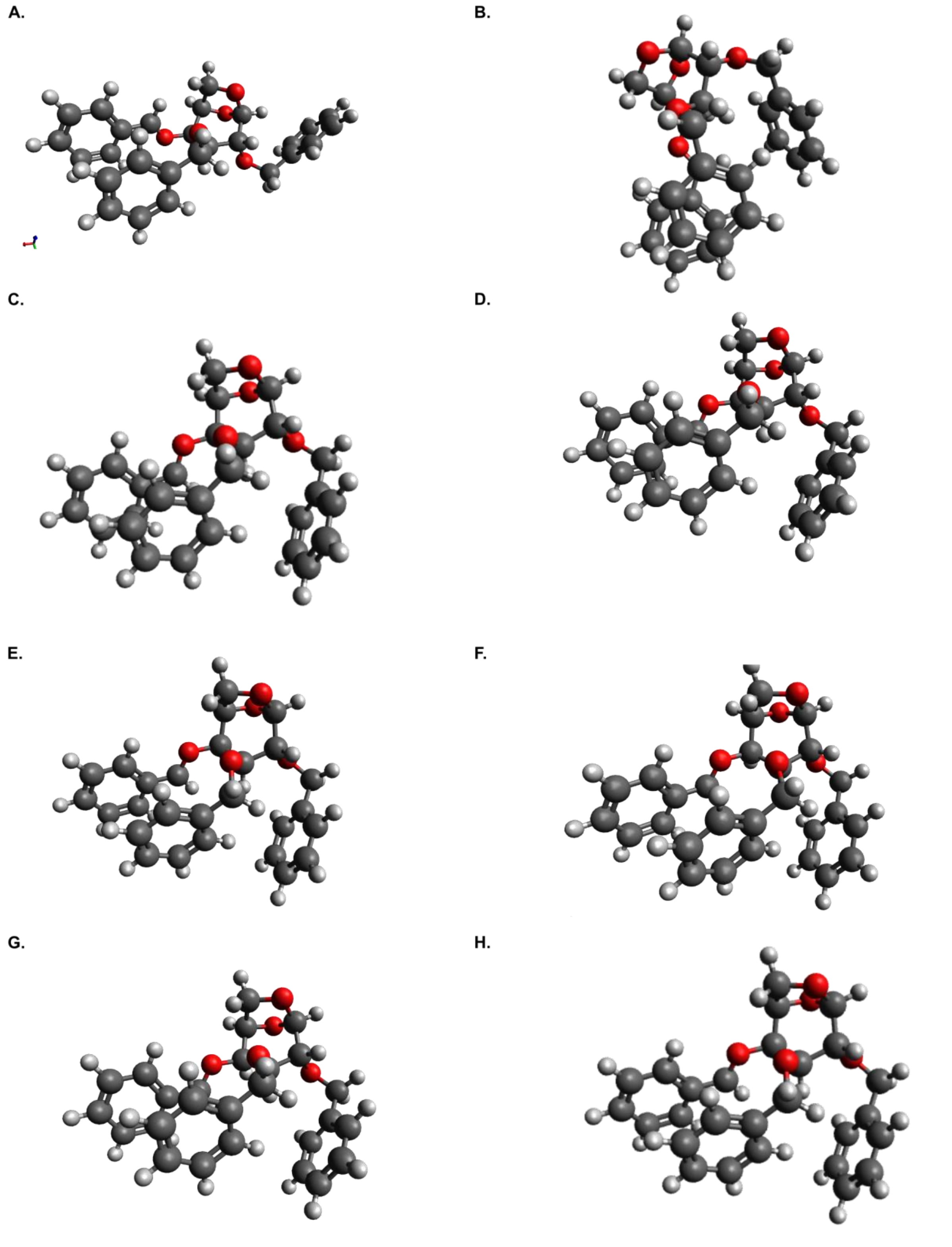

Figure S29. Reoptimized geometries of **Gal-6B** within different surrounding medias (solvents). The structures correspond to those that are highlighted in entry 1, Table S6. A) 1,4-Dioxane. B) Toluene. C) Et_2O. D) $CHCl_3$. E) DCM. F) ACN. G) DMSO. H) Water.

3. Exploring the Impact of Counterions on the Structure of Glycosyl Cations

3.1 Study Promoter Effect on the Structure of Glycosyl Cations using IMS

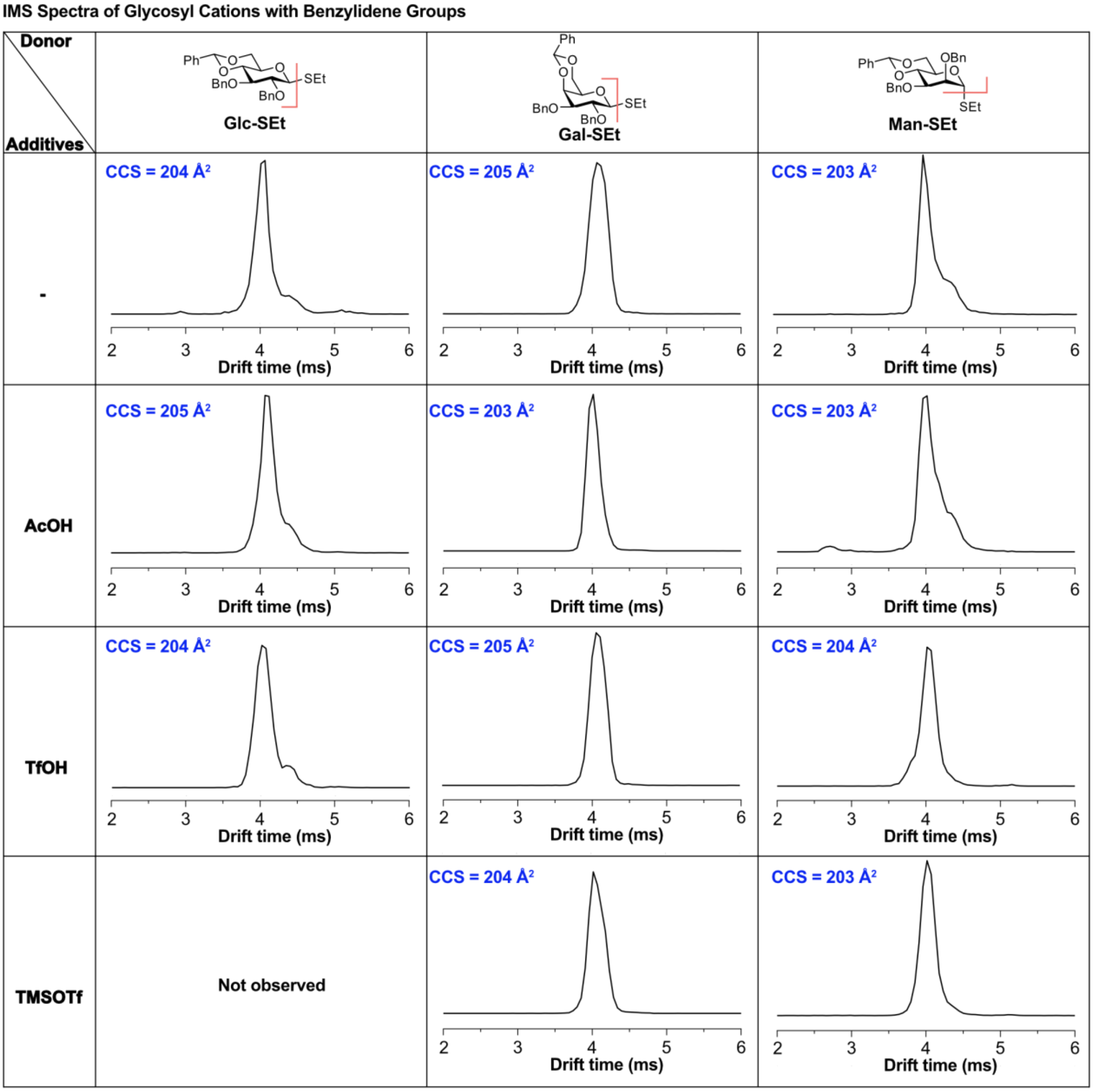

Figure S30. IMS-MS mobiligram of glycosyl cations. a) Fragmentation of thioglycoside occurs through in-source fragmentation following electrospray ionization, resulting in the formation of glycosyl cations. Various acids were introduced into the samples. The conformation of glycosyl cation is studied using traveling wave ion mobility spectrometry (TW-IMS). b) IM–MS drift-time distributions for glycosyl cations (m/z= 431) are presented. The values in Å^2 correspond to the estimated Collisional Cross Sections (CCSs) in the nitrogen drift gas. Despite the presence of different acids, the conformation of glycosyl cations is nearly identical based on their CCSs, indicating that the presence of different acids has minimal influence on altering the conformation of glycosyl cations.

3.2 Leaving Group Effect on the Structure of Glycosyl Cations

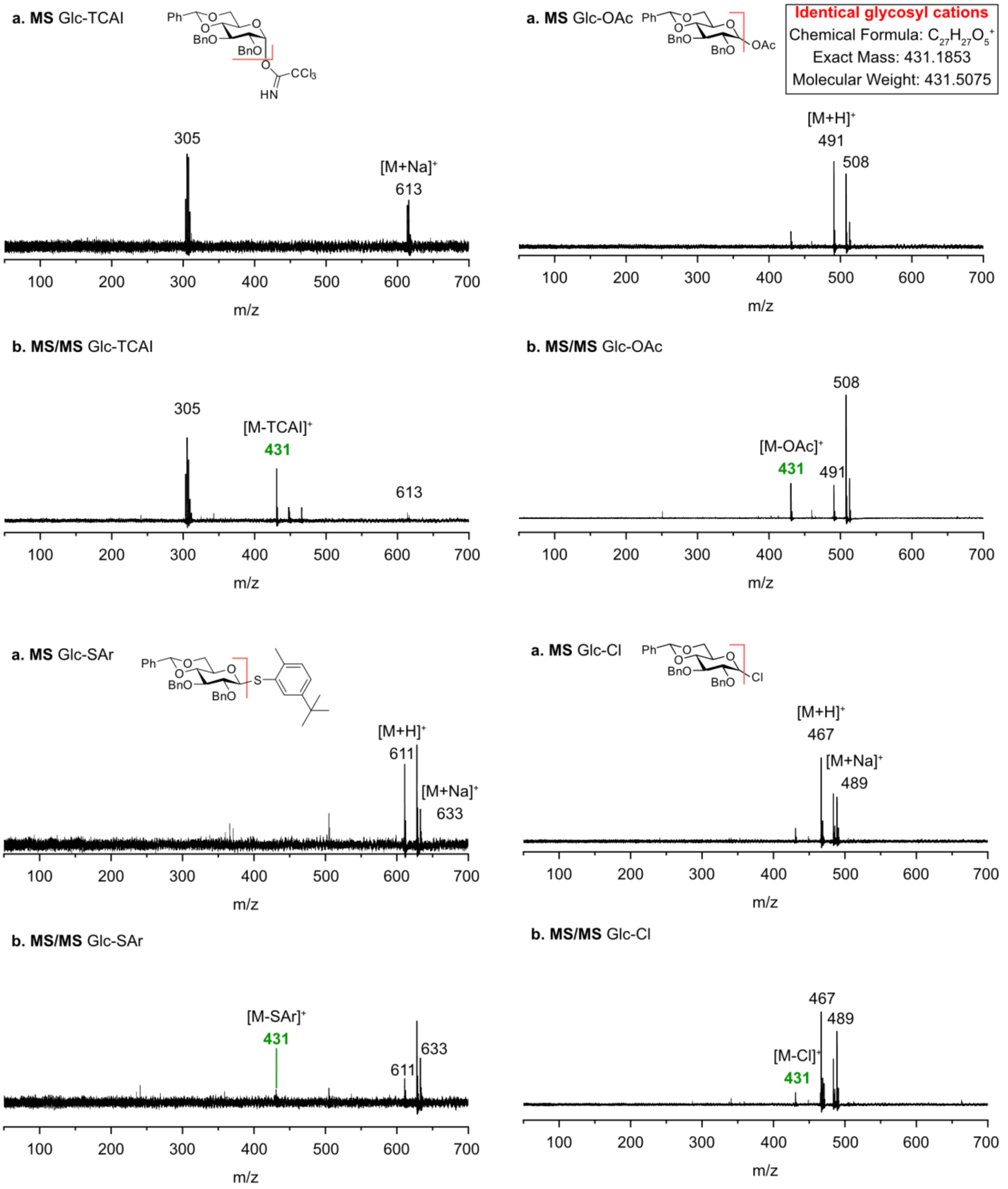

Figure S31. Mass spectra of glucosyl precursors (Glc-TCAI, Glc-OAc, Glc-SAr, and Glc-Cl). In-source fragmentation of precursor ions $[M+H]^+$ and $[M+Na]^+$ leads to identical cations (m/z = 431). The difference lies in the intensity of the TOF signal, with the ranking as follows: TCAI > OAc > Cl >> SAr. According to our reported work,[10] the formation of glycosyl cations does not exhibit an anomeric memory of fully protected thioglycoside and glycosyl imidate.

3.3 Study the Counterion Effect on the Structure of Glycosyl Cations using Computational Method

Initial geometries of glycosyl cations candidates bearing triflate anion (OTf^-) were constructed by chemical intuition using GaussView 6.[3] Conformational search were performed using CREST[4] with the semiempirical method GFN2-xTB[5] using default settings. The selected structures are reoptimized and their harmonic frequencies are computed at the PBE1PBE/6-311+G(d,p) EmpiricalDispersion=GD3BJ[6,7] level of theory using Gaussian 16.[3] Relative free energies at -78 °C were represented in the figures below. For the glucose (Figures S33) and mannose, investigations into the oxocarbenium ion do not show the corresponding ion pair structure. Instead, the findings suggest the formation of a covalent intermediate (β-triflate), which exhibits significantly lower free energy.

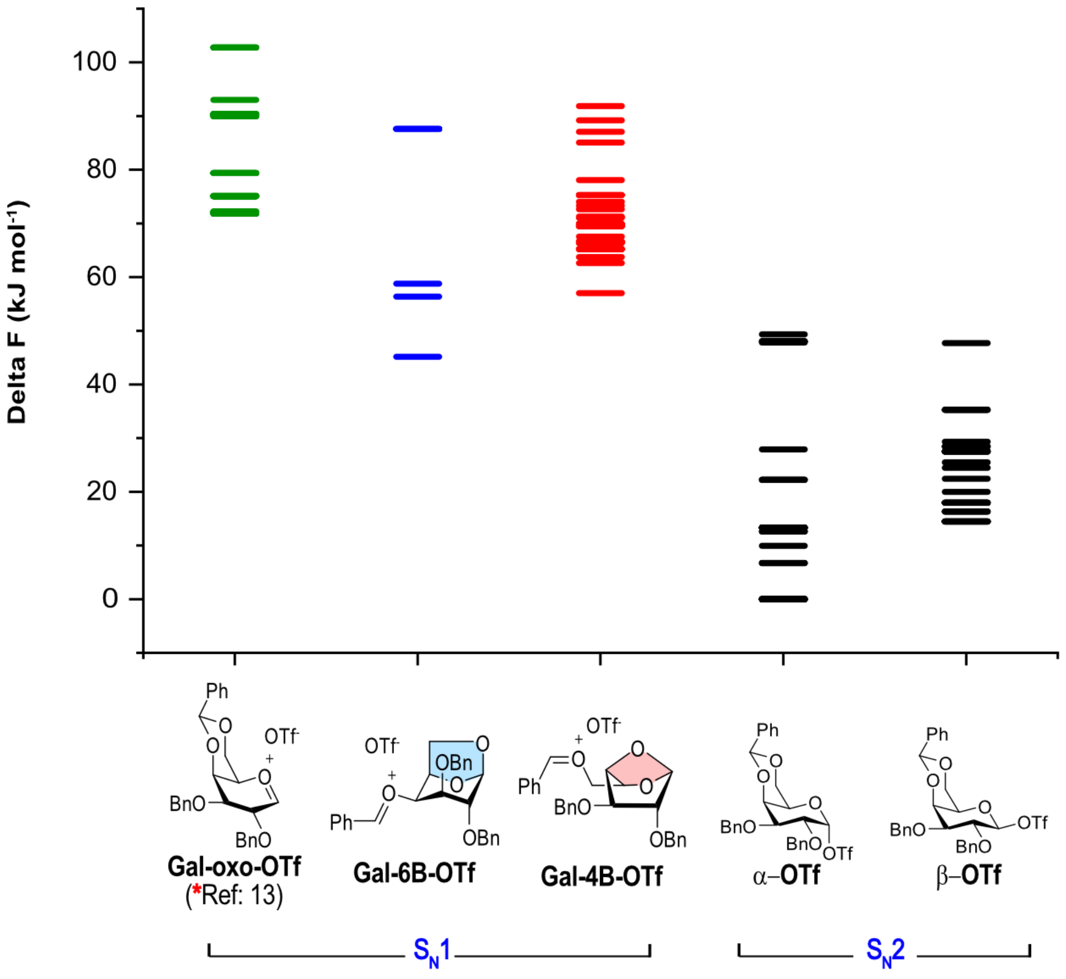

Figure S32. Energy hierarchies of the reoptimized structures for galactosyl cations with triflate anion (OTf) at -78 °C. Following the general DFT calculation procedure, the formation of anhydro cations (**Gal-6B-OTf**: +45 kJ mol^{-1}; **Gal-4B-OTf**: +57 kJ mol^{-1}) exhibit significantly greater thermodynamic stability compared to oxocarbenium ions (+72 kJ mol^{-1}). Based on DFT data, galactosyl (α-OTf and β-OTf) are covalent intermediates and their free energy is lower than that of glycosyl cations. Glycosylation reaction proceeds through a continuum mechanism spanning both S_N1 and S_N2 manifolds. Therefore, both S_N1 and S_N2 pathways need to be taken into account. It agrees that β-triflate contributes α-glycosylation via the S_N2 mechanism. However, in the S_N1-side mechanism, the stereoselectivity in the glycosylation reaction is dominated by glycosyl cations, where the anhydro cations (**Gal-6B-OTf**, **Gal-4B-OTf**) are thermodynamically stable, thereby serving as the key species for S_N1 glycosylation

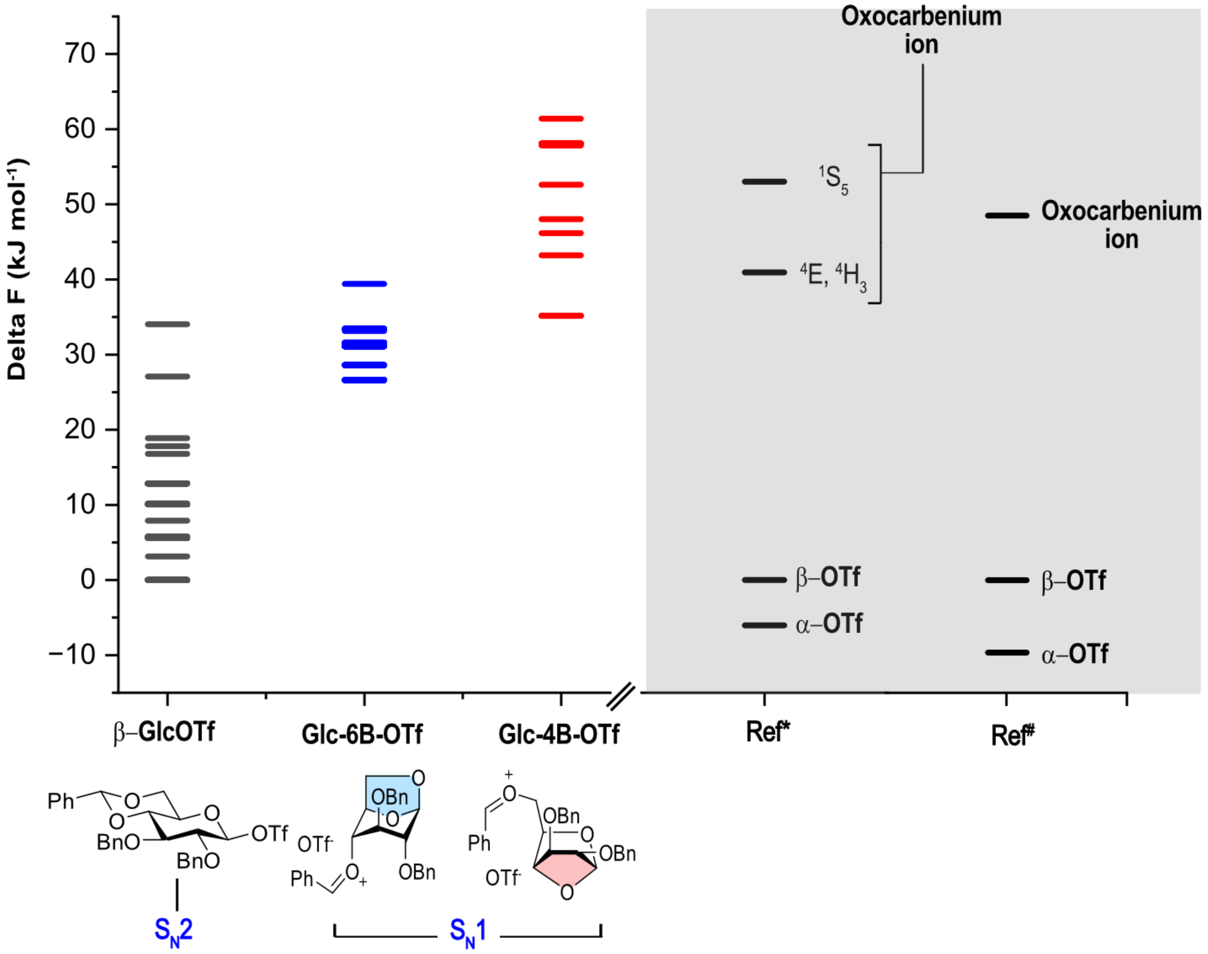

Figure S33. Energy hierarchies of the reoptimized structures for glucosyl cations with triflate anion (OTf$^-$) and covalent intermediate at -78 °C. Following the general DFT calculation procedure, the investigations into the oxocarbenium ion do not show the corresponding ion pair structure. Instead, the findings suggest the formation of a β-triflate. From an energy standpoint, the formation of β-glycosyl triflate is typically more thermodynamically stable than glycosyl cations. Consequently, it is commonly assumed that β-glycosyl triflate can effectively dictate high α-selectivity through S_N2 reactions. However, when the reaction pathway shifts to the S_N1-side, formation of anhydro cations exhibit lower free energy compared to oxocarbenium ions, of which DFT data (Ref*[11-13] and Ref#[14]) are acquired from report under DFT(M06-2X) level of theory at same temperature. Therefore, besides oxocarbenium ions, the potential impact of anhydro cations cannot be ignored in S_N1 mechanism.

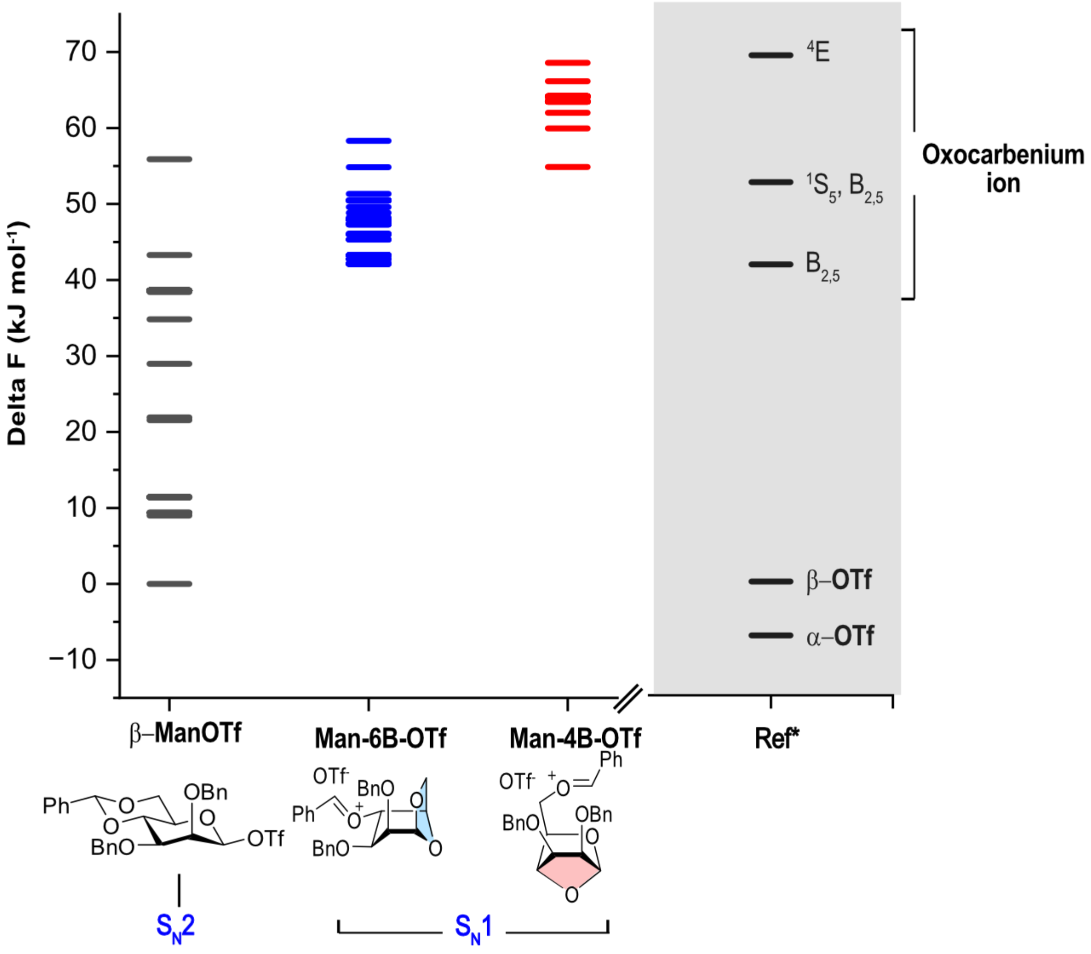

Figure S34. Energy hierarchies of the reoptimized structures for mannosyl cations with triflate anion (OTf⁻) and covalent intermediate at -78 °C. Following the general DFT calculation procedure, the investigations into the oxocarbenium ion do not show the corresponding ion pair structure. Instead, the findings suggest the formation of a β-triflate. From an energy standpoint, the formation of β-triflate is typically more thermodynamically stable than glycosyl cations. Consequently, it is commonly assumed that β-triflate can effectively dictate α-glycosylation through S_N2 reactions. However, when the reaction pathway shifts to the S_N1-side, formation of anhydro cations exhibit lower free energy compared to oxocarbenium ions, of which DFT data (Ref*[11-13]) is acquired from report under DFT(M06-2X) level of theory at same temperature. Therefore, besides oxocarbenium ions, the potential impact of anhydro cations cannot be ignored in S_N1 mechanism.

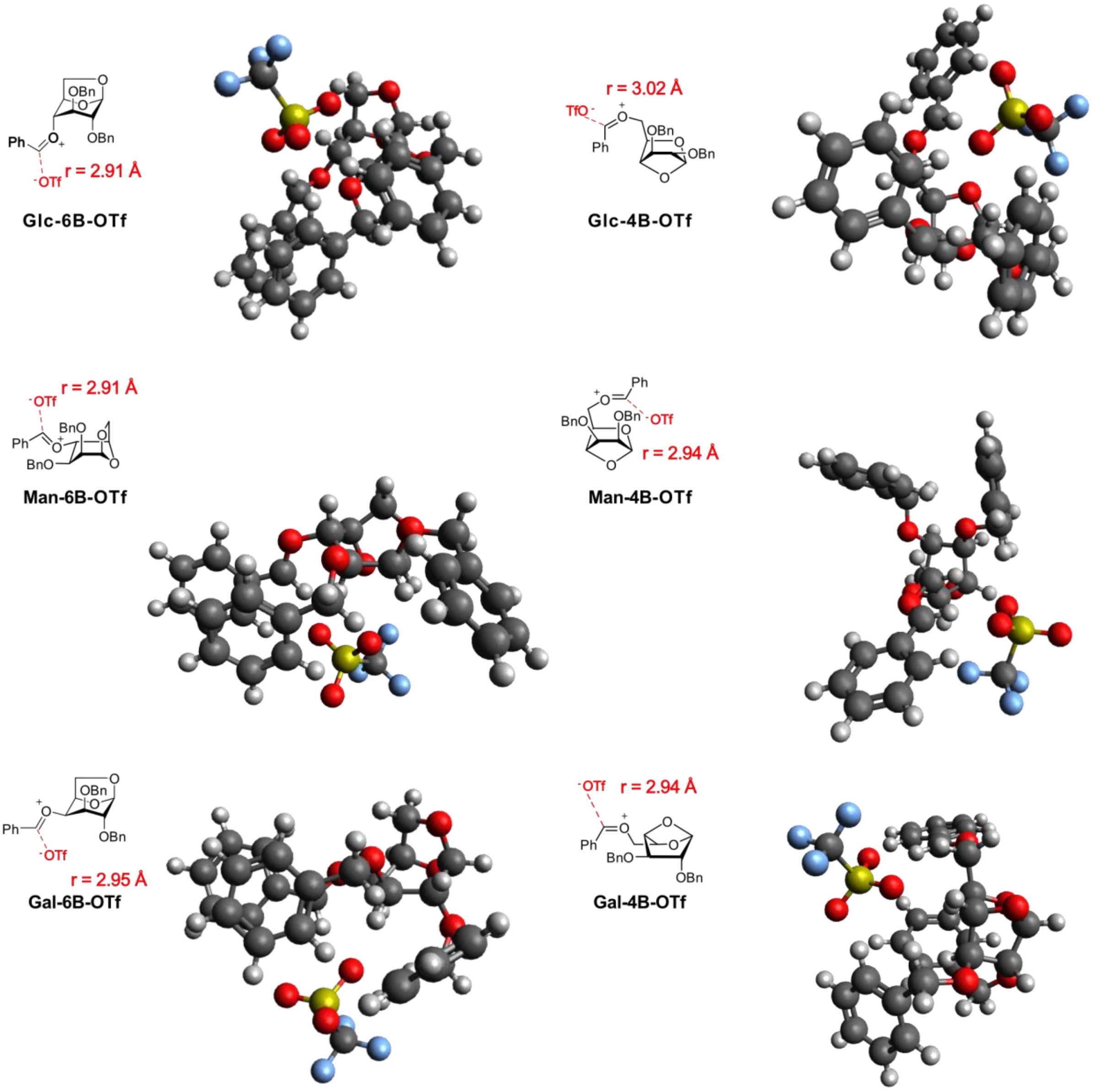

Figure S35. Reoptimized geometries of glycosyl cations in the presence of triflate anions (OTf-). The OTf- ions stabilize the glycosyl cation by coordinating with the benzylic carbocation ($PhCHO^{+}$). The optimized geometry reveals a $^{-}O(Tf) – C(=O^{+})Ph$ bond about 3.0 Å.

3.4 Analysis of the Electrophilic Site in Anhydro Cations

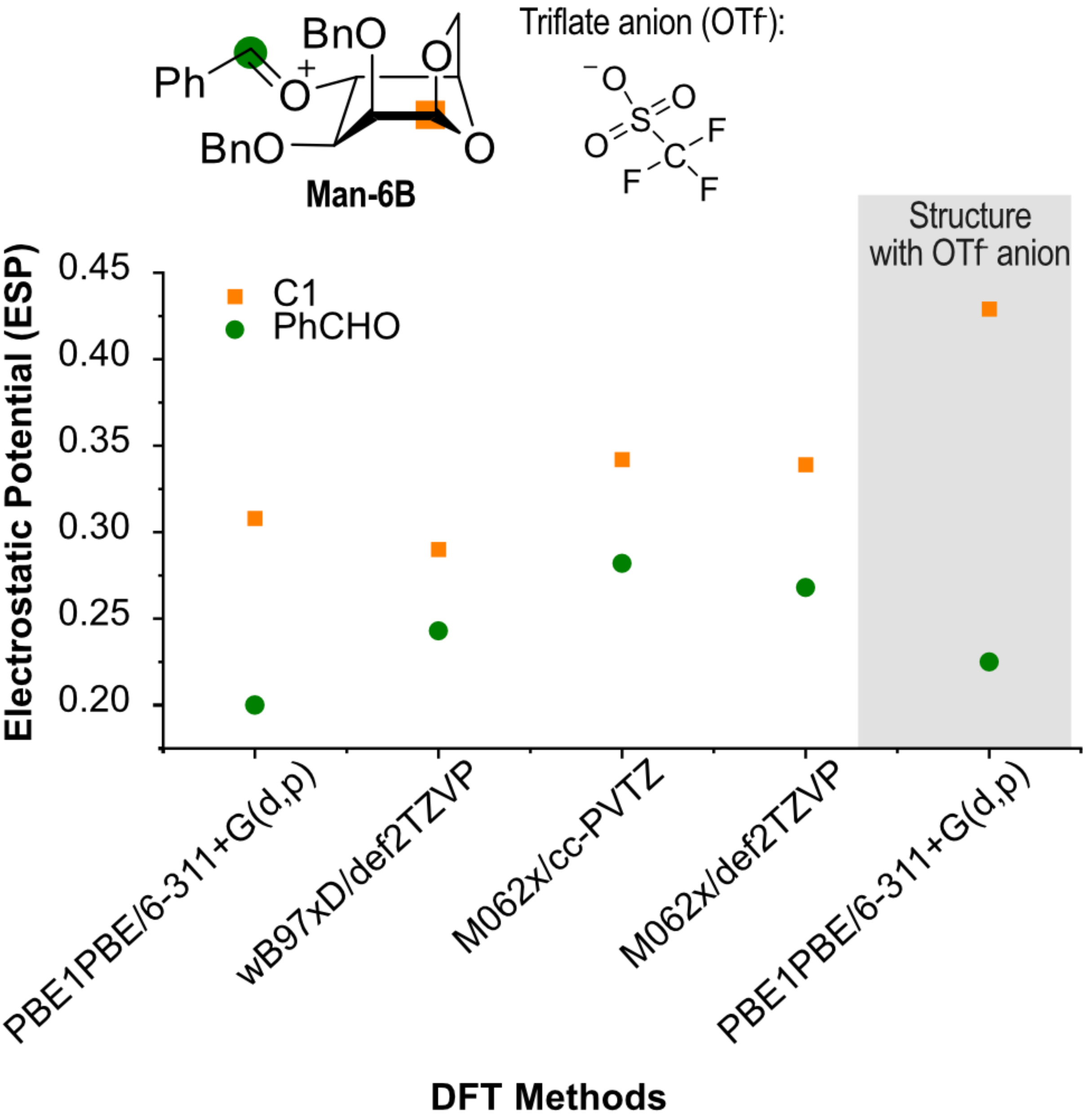

Figure S36. Electrostatic potential (ESP) mapping of the first stable conformers of the anhydro cation. The anomeric carbon (orange square) exhibits a greater positive charge compared to the benzylic carbocation (green circle). Therefore, anomeric carbon (C1) is a better electrophilic site for acceptor attacks during glycosylation reactions. From the structure point of view, the positive charge at the benzylic carbocation group is delocalized through resonance within the conjugated system of the phenyl ring, resulting in a lower ESP value. This observation remains consistent when employing DFT methods, even when the structure incorporates a triflate anion (gray box) strategy of utilizing ESP analysis to understand favored electrophilic substitution has been discussed in small molecule synthesis as well.[15,16]

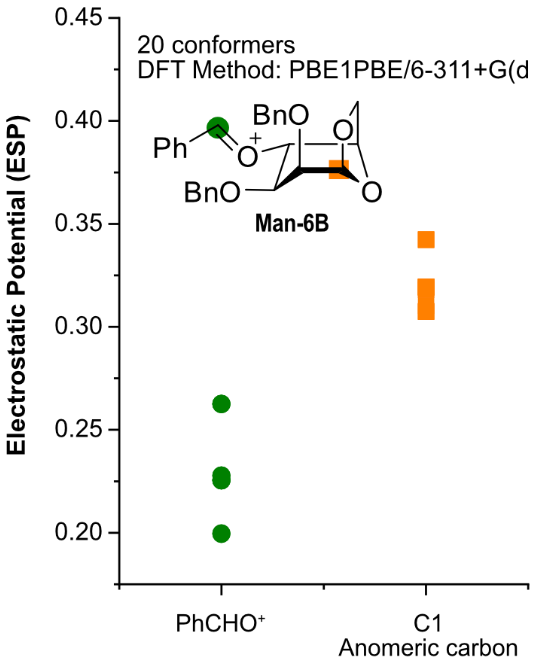

Conformers	ESP charge ($PhCHO^+$)	ESP charge (C1)
#1	0.199655	0.307581
#2	0.199444	0.307479
#3	0.199427	0.307518
#4	0.199408	0.307473
#5	0.199415	0.307467
#6	0.199433	0.310286
#7	0.199441	0.307477
#8	0.199419	0.307508
#9	0.262528	0.342348
#10	0.227726	0.318111
#11	0.225434	0.319354
#12	0.225423	0.319379
#13	0.225413	0.319326
#14	0.225557	0.319366
#15	0.262513	0.342322
#16	0.227753	0.318154
#17	0.22544	0.319295
#18	0.225517	0.319395
#19	0.225529	0.319312
#20	0.262568	0.342391

Figure S37. Electrostatic potential (ESP) mapping of the first stable conformers of the anhydro cation with 20 conformers. The ESP values for C1 ranged from 0.3 to 0.35, surpassing those of the benzylic carbocation (PhCHO), which ranged from approximately 0.2 to 0.26. Therefore, anomeric carbon (C1) is a better electrophilic site for acceptor attacks during glycosylation reactions.

4. Conformational Analysis of Glycosyl Cations

4.1 Boltzmann Analysis of the Anhydro Cations

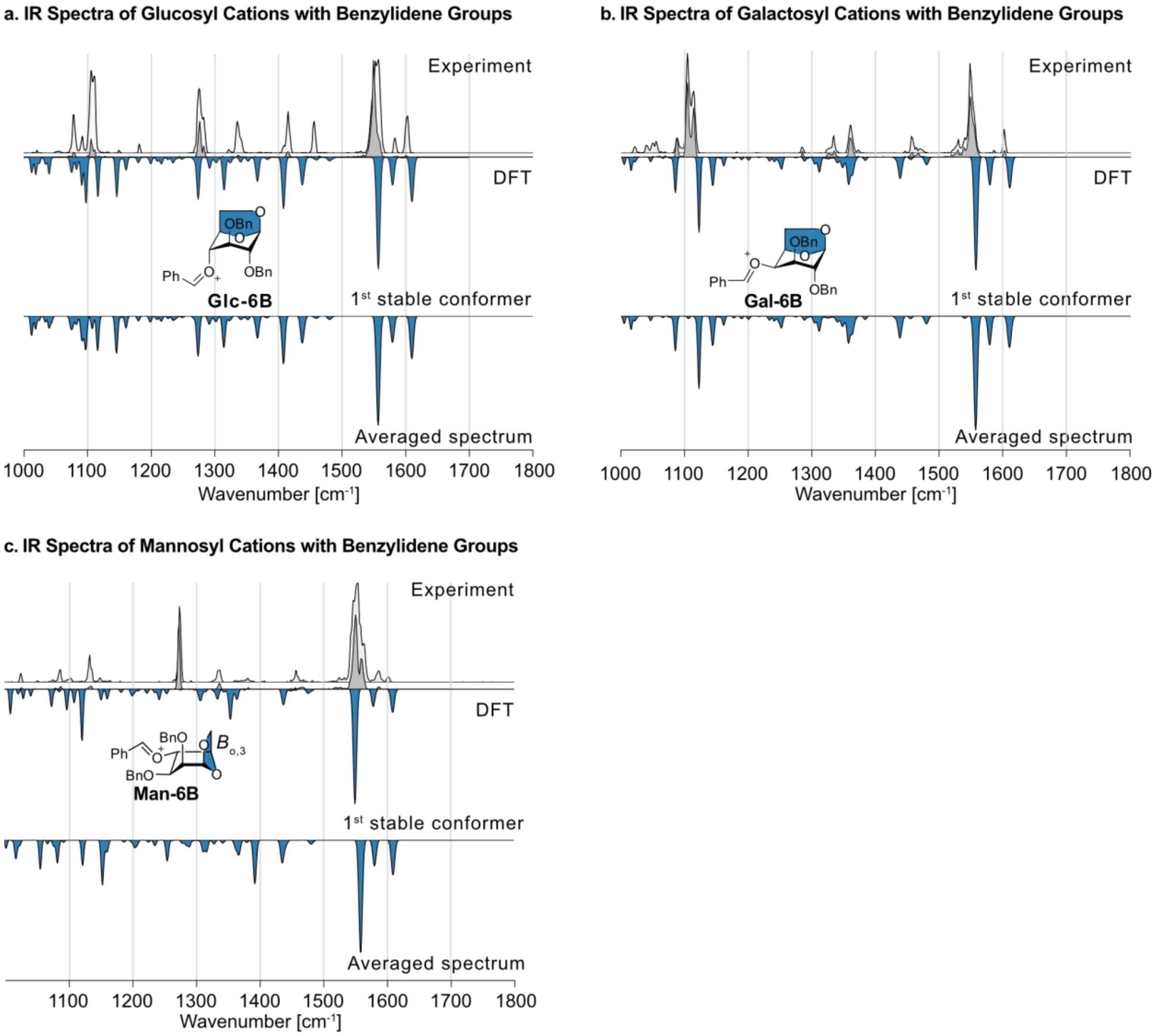

Figure S38. Boltzmann analysis of all calculated conformers and the averaged IR spectra. The resulting spectra do not differ significantly from the single-ion spectrum of the most stable species. This can be explained by Boltzmann equation according to which a difference in 3.5 kJ/mol results in a decrease in population of the respective conformer of 99% at 90 K, which is the temperature before the ions are shock-frozen in helium droplets at 0.4 K.

4.2 Solvents Impact on the Conformational Population of Glycosyl Cations.

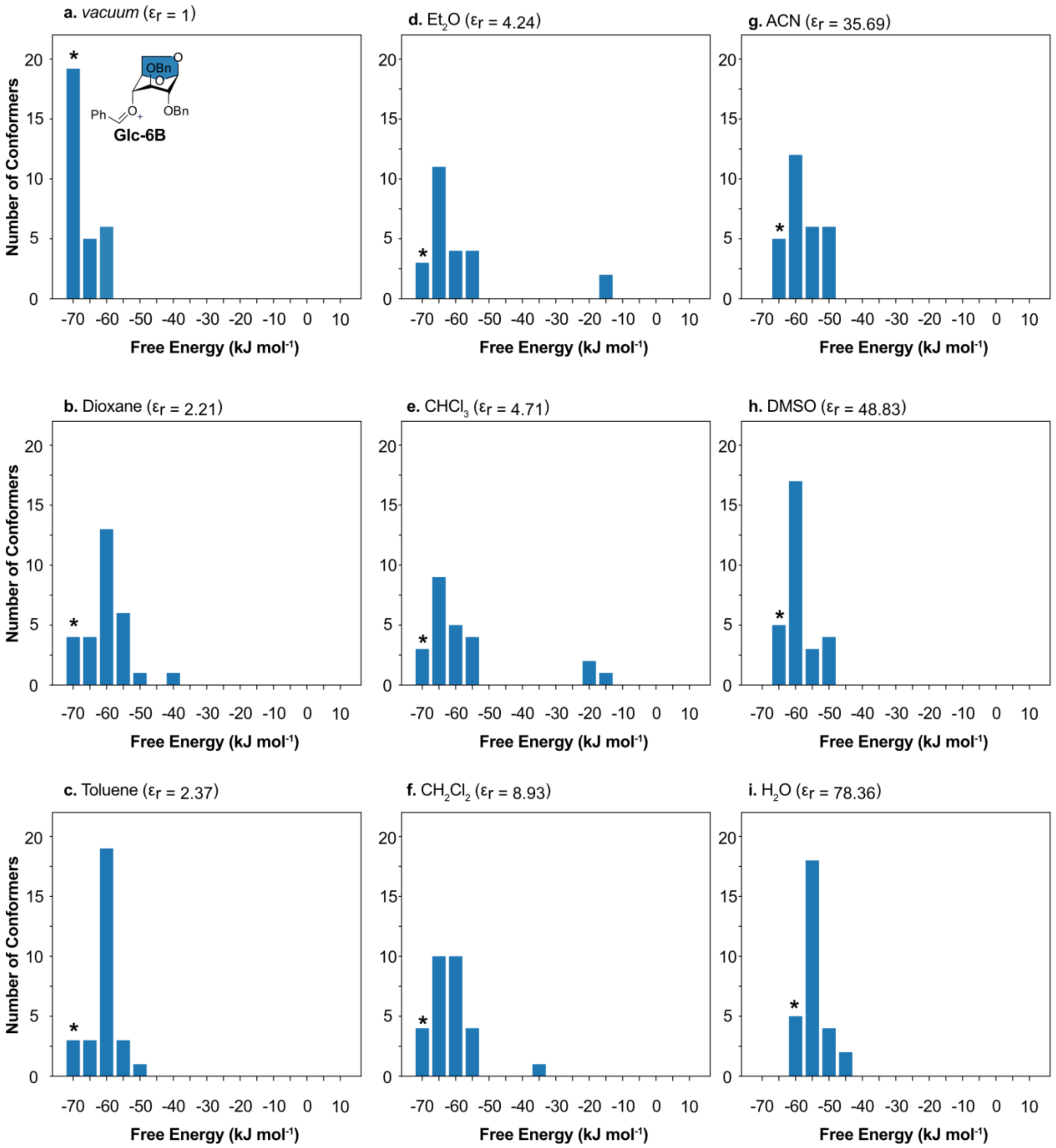

Figure S39. Conformational population of **Glc-6B** across various solvent models. Free energies were calculated relative to its oxocarbenium ion (ΔF = 0). In a vacuum, the glycosyl cations demonstrate significant stability, with a high abundance of low-energy conformers (less than -70 kJ mol-1). However, with an increase in electric permittivity, the distribution of conformers gradually right shifts to higher energies. The resulting data aligns with theory as the conformer distribution in *vacuum* remains similar to that observed in highly apolar organic solvents in comparison to aqueous solution. *The lowest free-energy conformers are selected in the discussion of the relative stability of glycosyl cations in various solvents in the manuscript or SI.

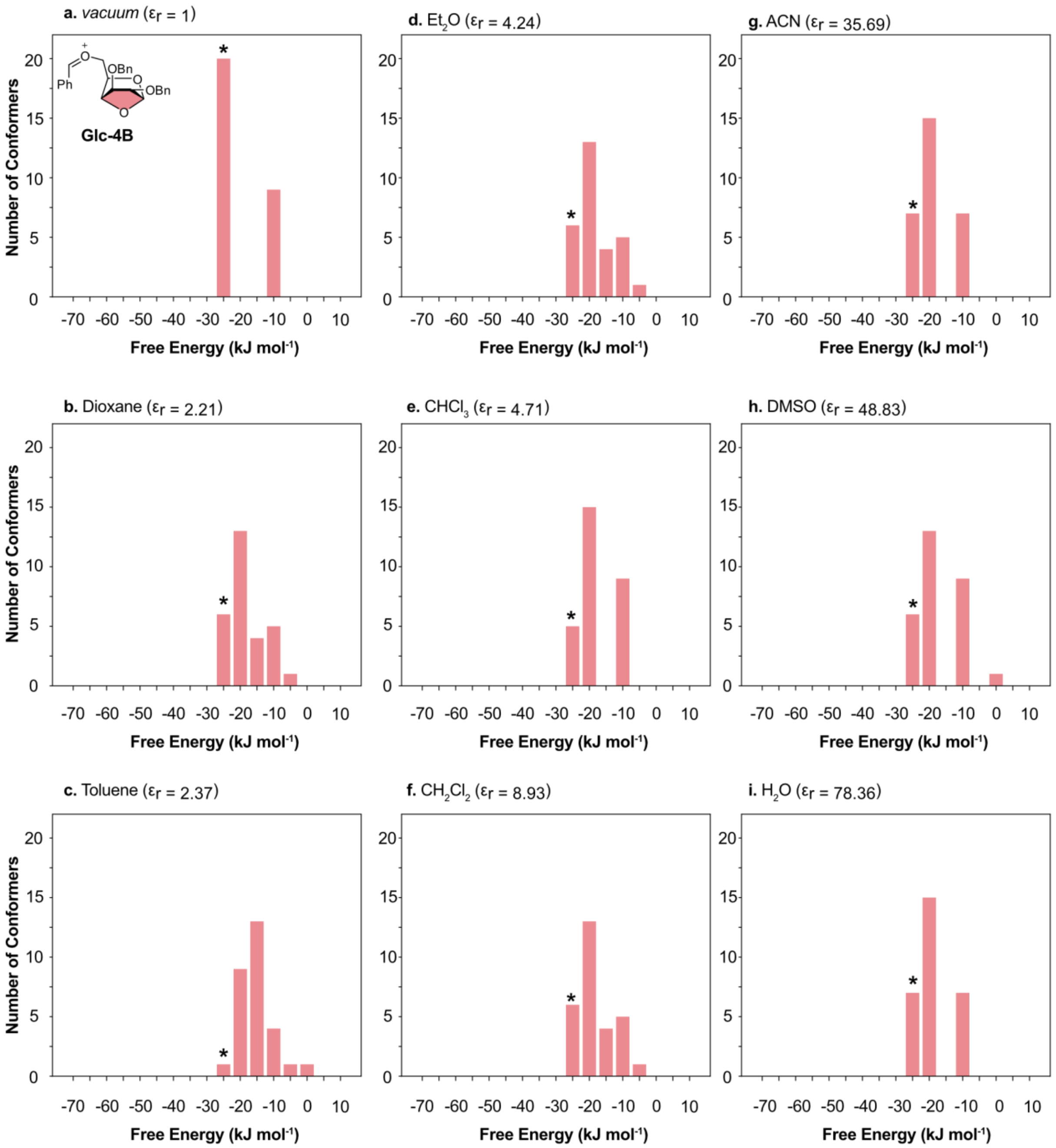

Figure S40. Conformational population of **Glc-4B** across various solvent models. Free energies were calculated relative to its oxocarbenium ion (ΔF = 0). In a vacuum, the glycosyl cations demonstrate significant stability, with a high abundance of low-energy conformers (mainly -25 kJ mol^{-1}). However, with an increase in electric permittivity, the distribution of conformers gradually right shifts to higher energy side. *The lowest free-energy conformers are selected in the discussion of the relative stability of glycosyl cations in various solvents in the manuscript or SI.

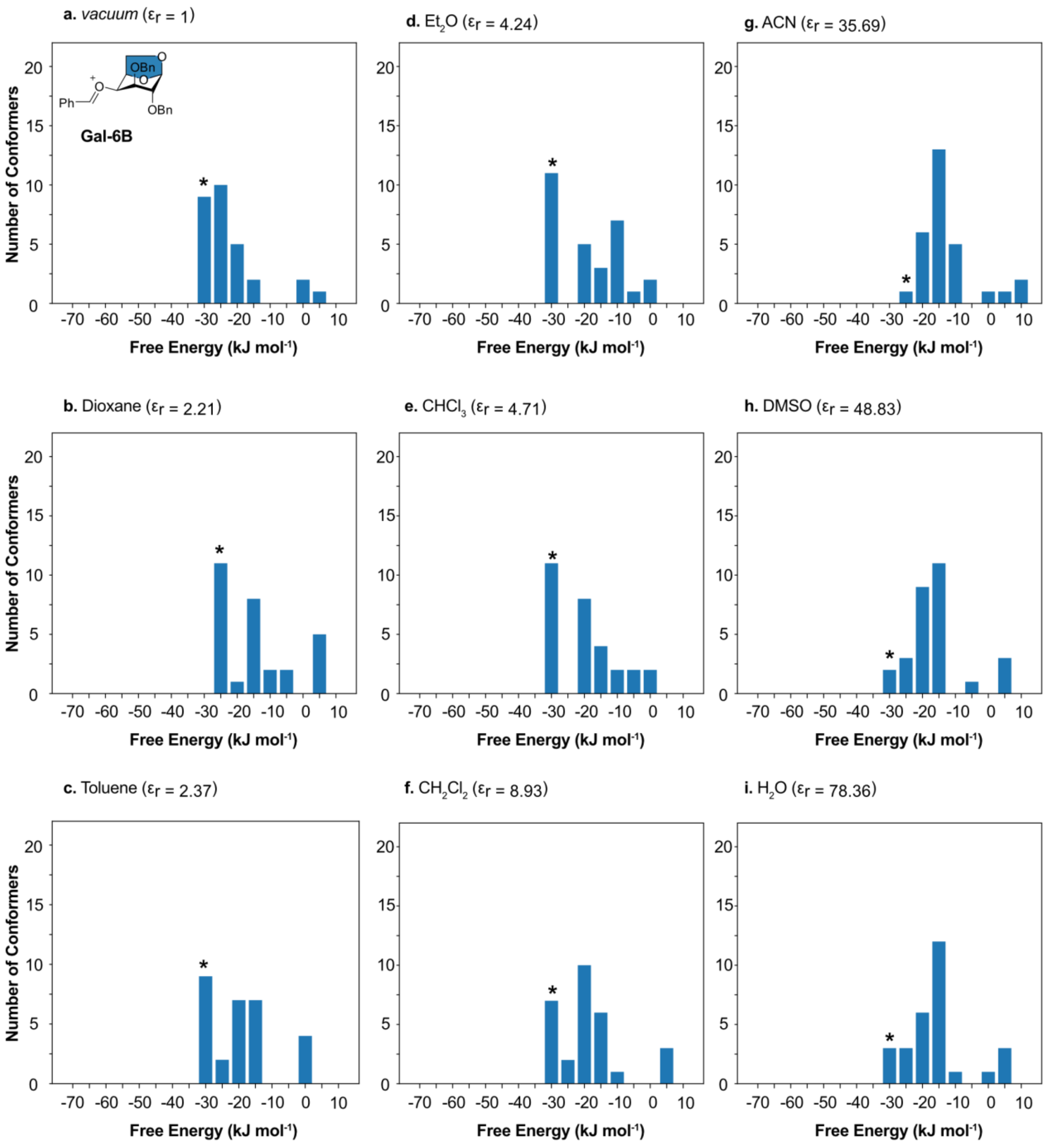

Figure S41. Conformational population of **Gal-6B** across various solvent models. Free energies were calculated relative to its oxocarbenium ion (ΔF = 0). In a vacuum, the glycosyl cations demonstrate significant stability, with a high abundance of low-energy conformers (mainly ranging -30 to -20 kJ mol^{-1}). However, with an increase in electric permittivity, the distribution of conformers gradually right shifts to higher energies. The resulting data aligns with theory as the conformer distribution in *vacuum* remains similar to that observed in highly apolar organic solvents in comparison to aqueous solution. *The lowest free-energy conformers are selected in the discussion of the relative stability of glycosyl cations in various solvents in the manuscript or SI.

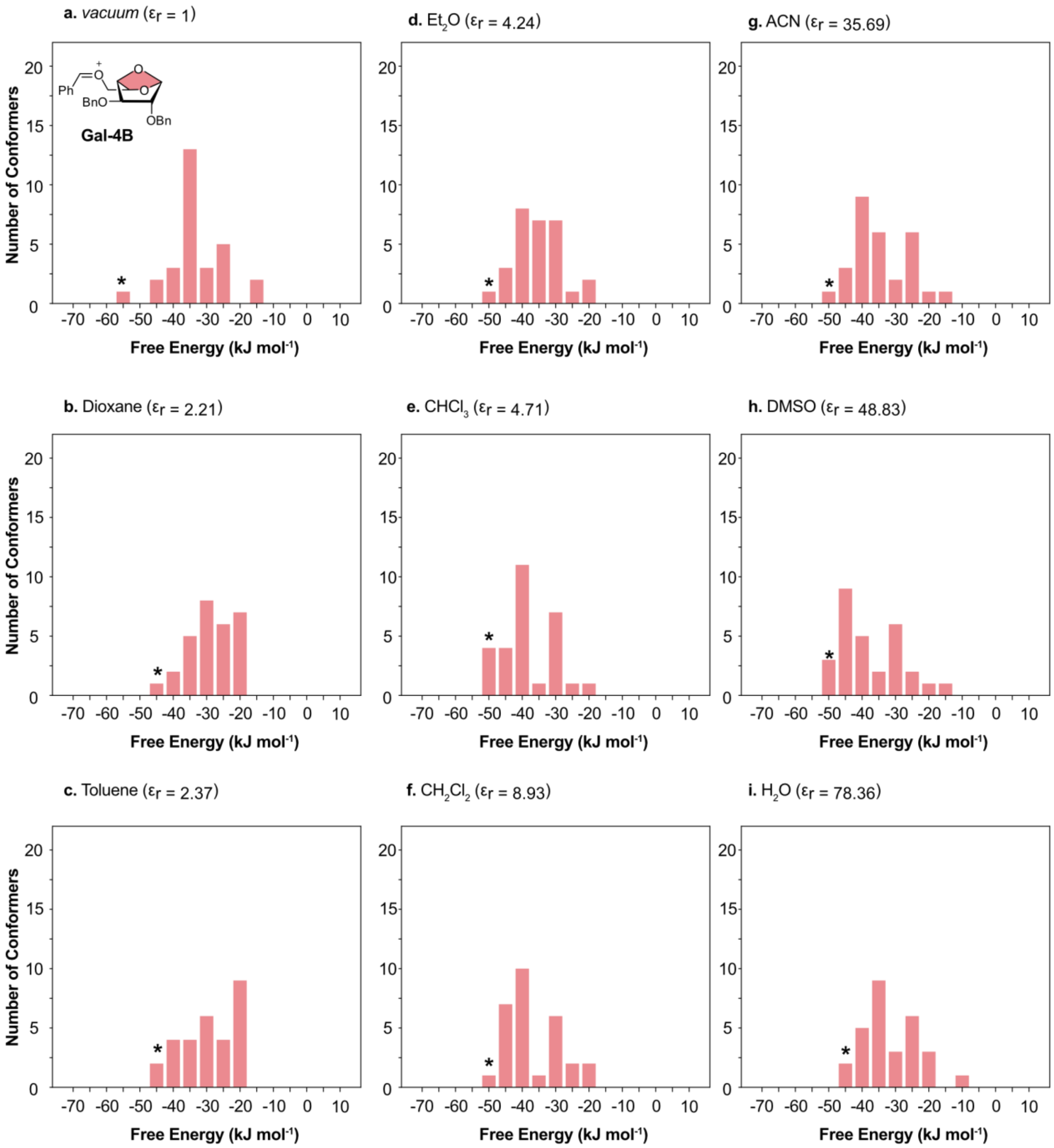

Figure S42. Conformational population of **Gal-4B** across various solvent models. Free energies were calculated relative to its oxocarbenium ion (ΔF = 0). In a vacuum, the glycosyl cations demonstrate significant stability, with a high abundance of low-energy conformers (mainly at -35 kJmol^{-1}). However, with an increase in electric permittivity, the distribution of conformers gradually right shifts to higher energies. The resulting data aligns with theory as the conformer distribution in *vacuum* remains similar to that observed in highly apolar organic solvents in comparison to aqueous solution. *The lowest free-energy conformers are selected in the discussion of the relative stability of glycosyl cations in various solvents in the manuscript or SI.

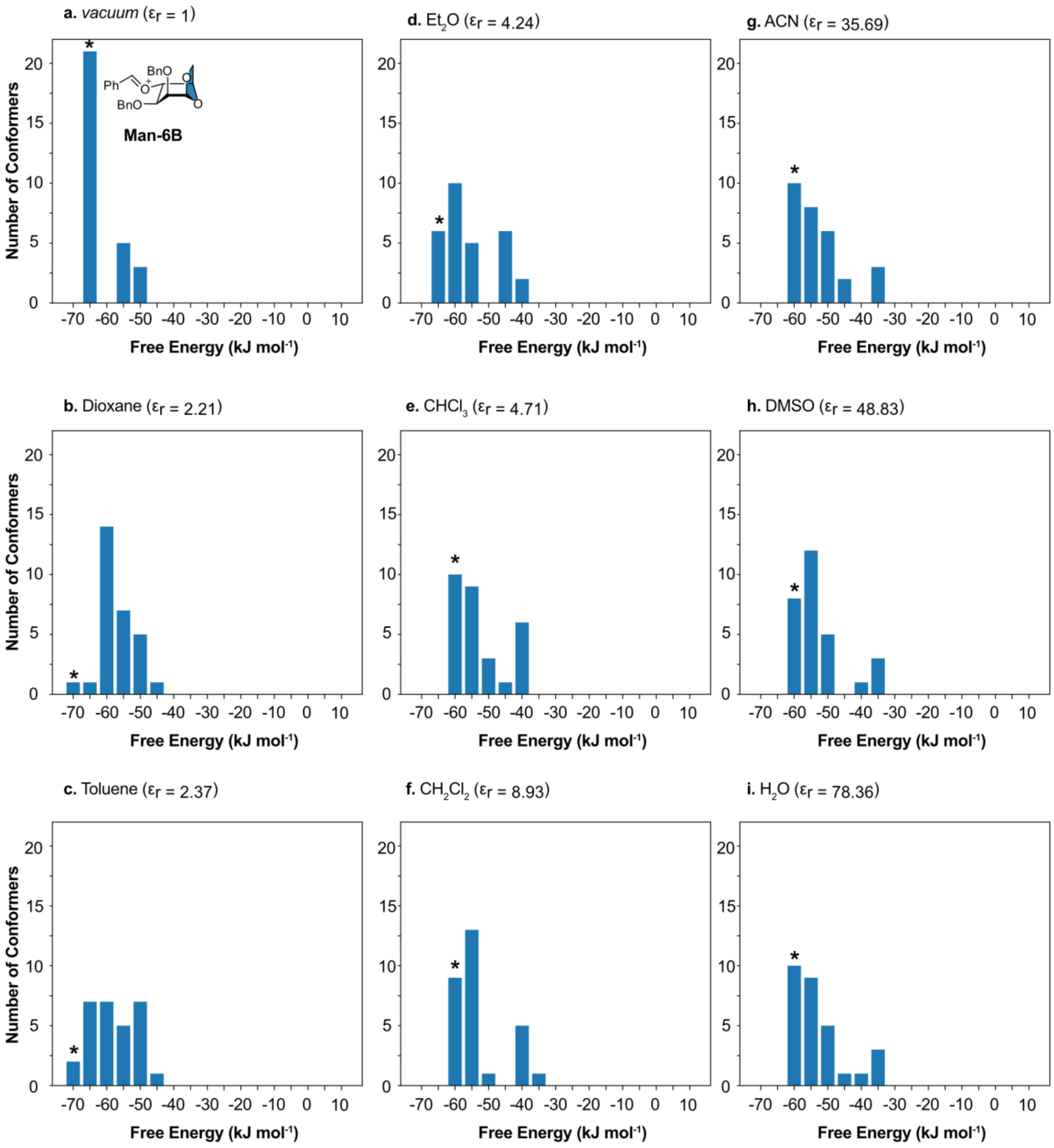

Figure S43. Conformational population of **Man-6B** across various solvent models. Free energies were calculated relative to its oxocarbenium ion (ΔF = 0). In a vacuum, the glycosyl cations demonstrate significant stability, with a high abundance of low-energy conformers (less than -65 kJ mol^{-1}). However, with an increase in electric permittivity, the distribution of conformers gradually right shifts to higher energies. The resulting data aligns with theory as the conformer distribution in *vacuum* remains similar to that observed in highly apolar organic solvents in comparison to aqueous solution. *The lowest free-energy conformers are selected in the discussion of the relative stability of glycosyl cations in various solvents in the manuscript or SI.

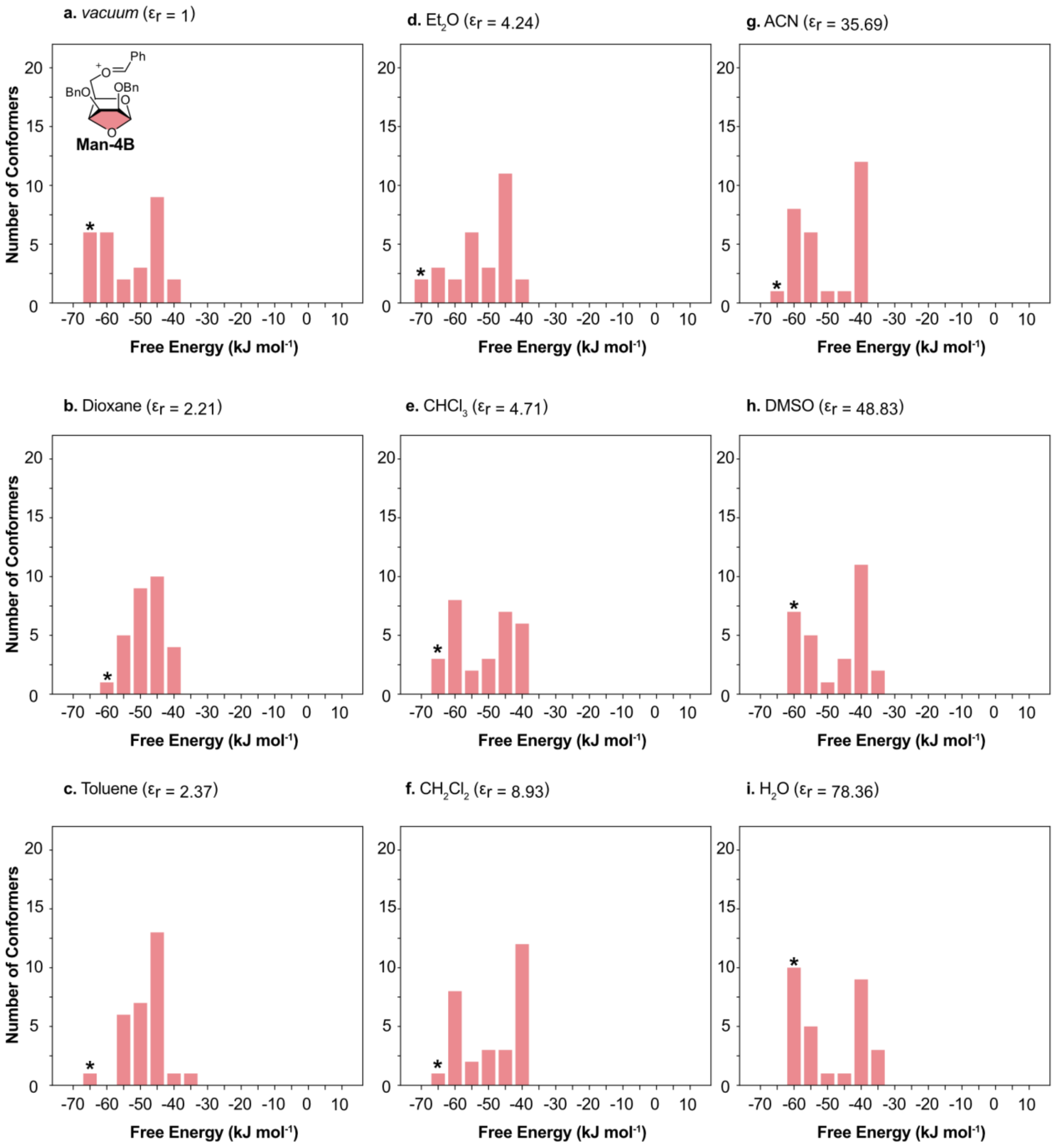

Figure S44. Conformational population of **Man-4B** across various solvent models. Free energies were calculated relative to its oxocarbenium ion (ΔF = 0). In a vacuum, the glycosyl cations demonstrate significant stability, with a high abundance of low-energy conformers (energy arranging -65 to -40 kJ mol^{-1}). However, with an increase in electric permittivity, the distribution of conformers gradually right shifts to higher energies. The resulting data aligns with theory as the conformer distribution in *vacuum* remains similar to that observed in highly apolar organic solvents in comparison to aqueous solution. *The lowest free-energy conformers are selected in the discussion of the relative stability of glycosyl cations in various solvents in the manuscript or SI.

5. xyz-Coordinates of Reoptimized Structures

xyz-Coordinates of all reoptimized geometries at the PBE0+D3/6-311+G(d,p) level of theory can be found in a separate document "SI_Coordinates_gas phase.pdf" and "SI_Coordinates_within solvents.pdf" and "SI_Coordinates_counter ion.pdf".

6. Experimental Details

6.1 Materials

All glycosyl precursors are liquid and, therefore, have no melting points. We have provided the full assignments of NMR and mass in this section. For the glycosyl cation intermediates, it could only be observed in the gas phase. Their mass, IR, and ion-mobility data are also addressed in SI.

Ethyl 2.3-di-O-benzyl-4,6-O-benzylidene-D-thio-glucopyranoside (**Glc-SEt**), ethyl 2.3-di-O-benzyl-4,6-O-benzylidene-D-thio-galactopyranoside (**Gal-SEt**), ethyl 2.3-di-O-benzyl-4,6-O-benzylidene-D-thio-mannopyranoside (**Man-SEt**) are commercially available from GlycoUniverse GmbH & Co.

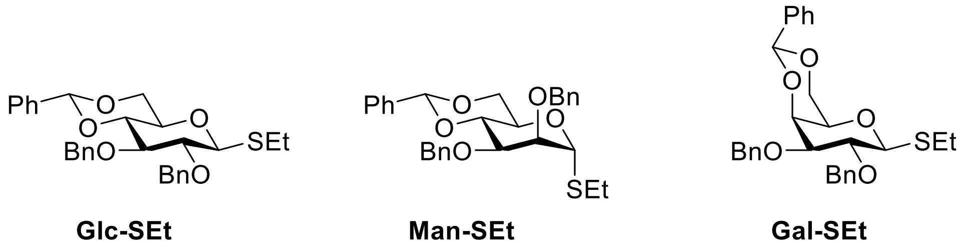

Four glycosyl precursors (**Glc-Cl**, **Glc-OAc**, **Glc-TCAI**, **Glc-SAr**) were selected for mass spectrometry. Synthesis and spectroscopic data are addressed below.

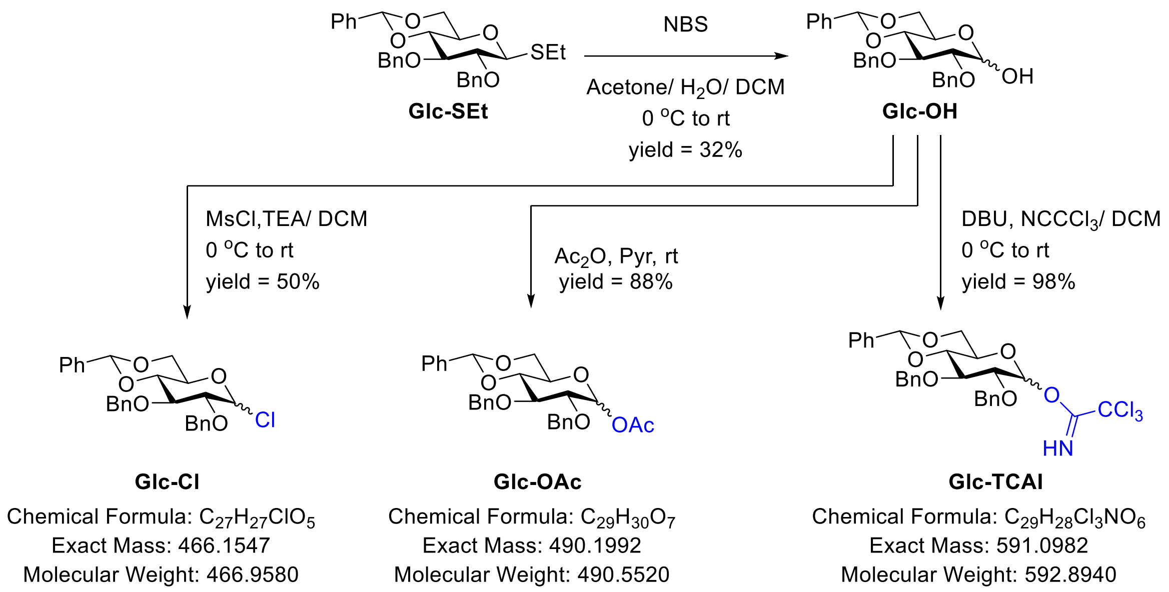

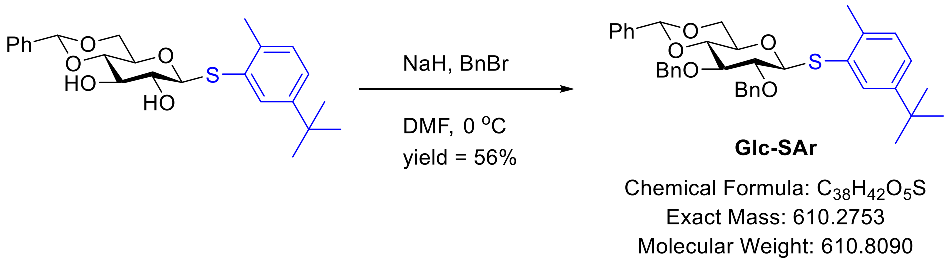

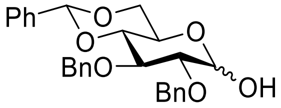

Glc-OH

(α/β = 1.8/1)

2,3-di-*O*-Benzyl-4,6-*O*-benzylidene-D-glucopyranoside

Following the procedures described in the literature,[17,18] thioglycoside **Glc-SEt**[19] (918 mg, 1.86 mmol, 1.0 equiv.) was dissolved in a mixture of acetone/water/DCM (9:1:1 v/v, 20 mL). Then NBS (995mg, 5.59 mmol, 3.0 equiv.) was added at 0 °C. The reaction was stirred at 0 °C for 1h, and then the reaction mixture was diluted by water and extracted with CH_2Cl_2 for 3 times. The combined organic phase was then washed with saturated $NaHCO_3$(aq) and dried over MgSO4. After removal of the solvent, the crude product was purified by column chromatography to get compound **Glc-OH** (267.1 mg) in 32% yield. ^{1}H NMR (600 MHz, CHLOROFORM-*D*) δ 7.57 – 7.46 (m, 6H), 7.43 – 7.27 (m, 34H), 5.58 (s, 1H), 5.57 (s, 2H), 5.20 (d, *J* = 3.8 Hz, 2H), 5.06 – 4.68 (m, 13H), 4.35 (dd, *J* = 10.5, 5.0 Hz, 1H), 4.30 (dd, *J* = 10.3, 5.0 Hz, 2H), 4.08 (td, *J* = 9.9, 5.0 Hz, 2H), 4.01 (t, *J* = 9.2 Hz, 2H), 3.71 (td, *J* = 9.8, 6.0 Hz, 3H), 3.47 (td, *J* = 9.7, 5.0 Hz, 1H), 3.42 (dd, *J* = 8.7, 7.6 Hz, 1H). ^{13}C NMR (151 MHz, CHLOROFORM-*D*) δ 137.37, 128.93, 128.54, 128.44,

128.34, 128.23, 128.14, 128.05, 128.02, 127.85, 127.67, 126.02, 125.97, 101.25, 97.81, 92.22, 82.98, 81.92, 81.57, 80.88, 79.30, 78.34, 77.21, 77.00, 76.79, 75.22, 73.85, 69.00, 68.70, 66.27, 62.52. HRMS (ESI) calcd for $C_{27}H_{28}NaO_6$ [M + Na]$^+$ 471.1784, found 471.1805.

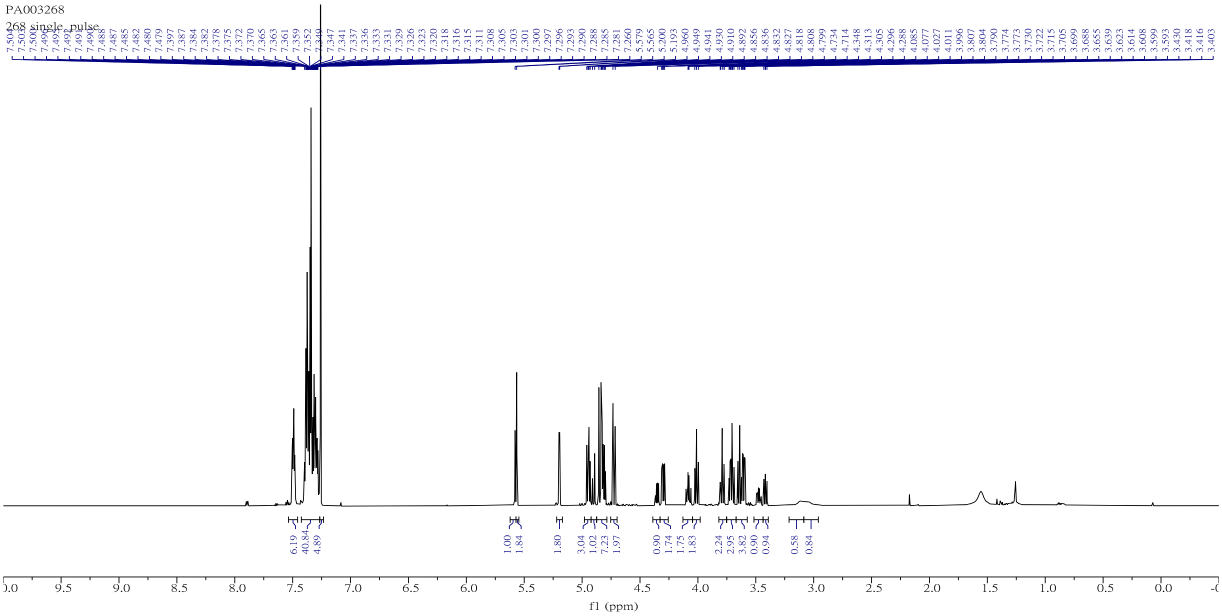

The ^{1}H Spectrum in $CDCl_3$ from 10.0 ppm to -0.5 ppm of Compound Glc-OH

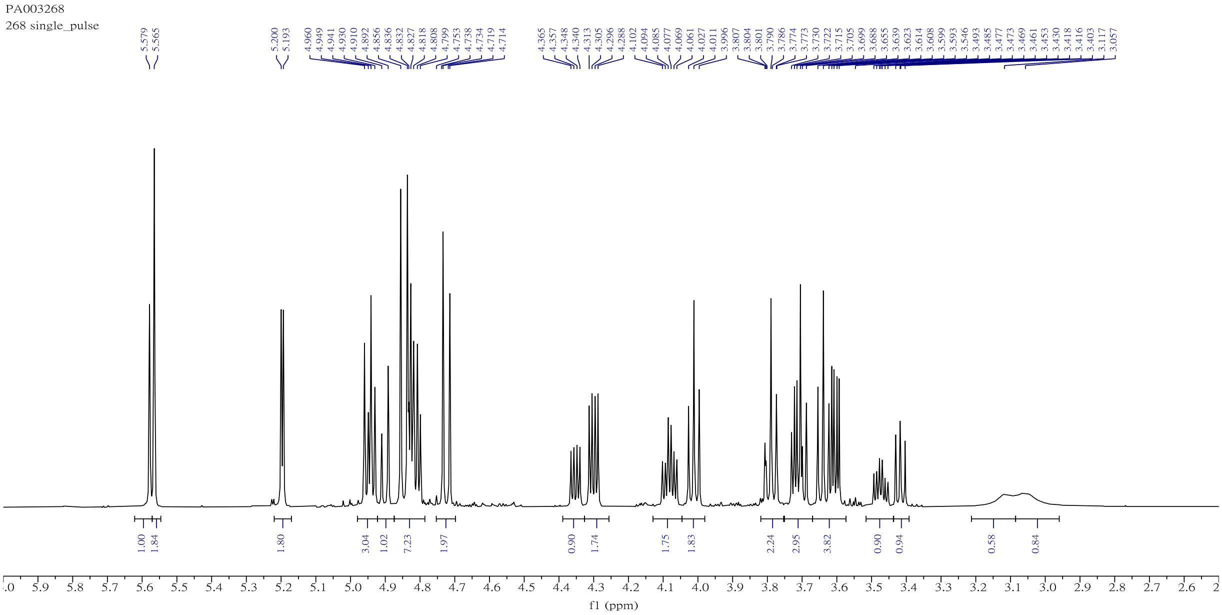

The ^{1}H Spectrum in $CDCl_3$ from 6.0 ppm to 2.0 ppm of Compound Glc-OH

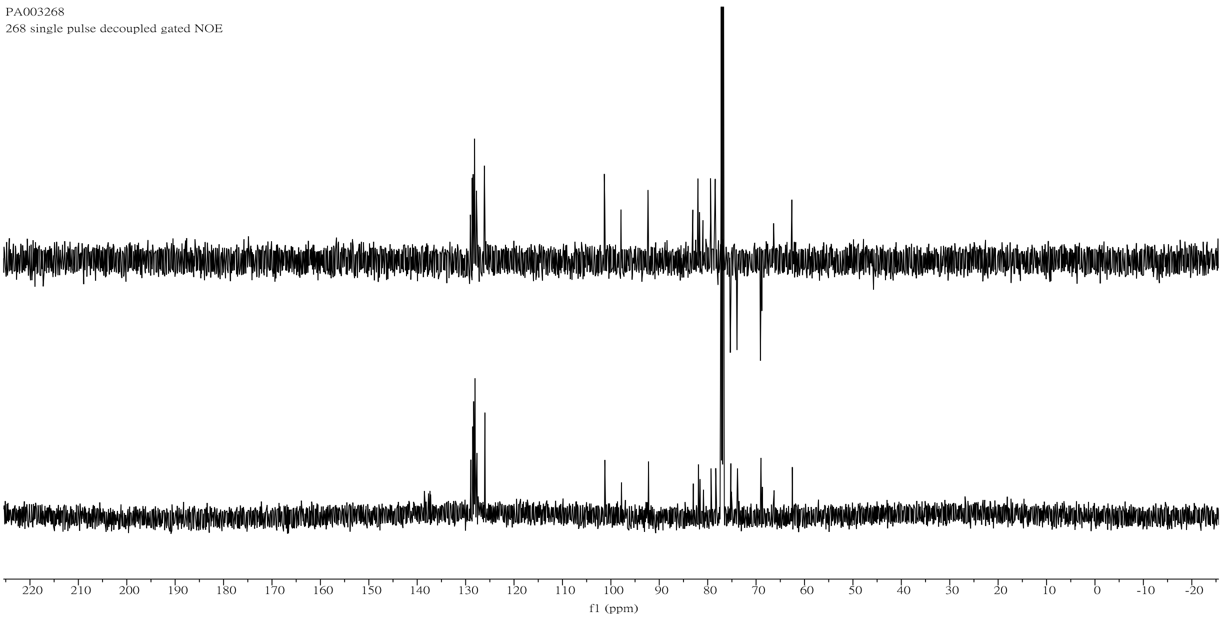

The ^{13}C Spectrum in $CDCl_3$ of Compound Glc-OH

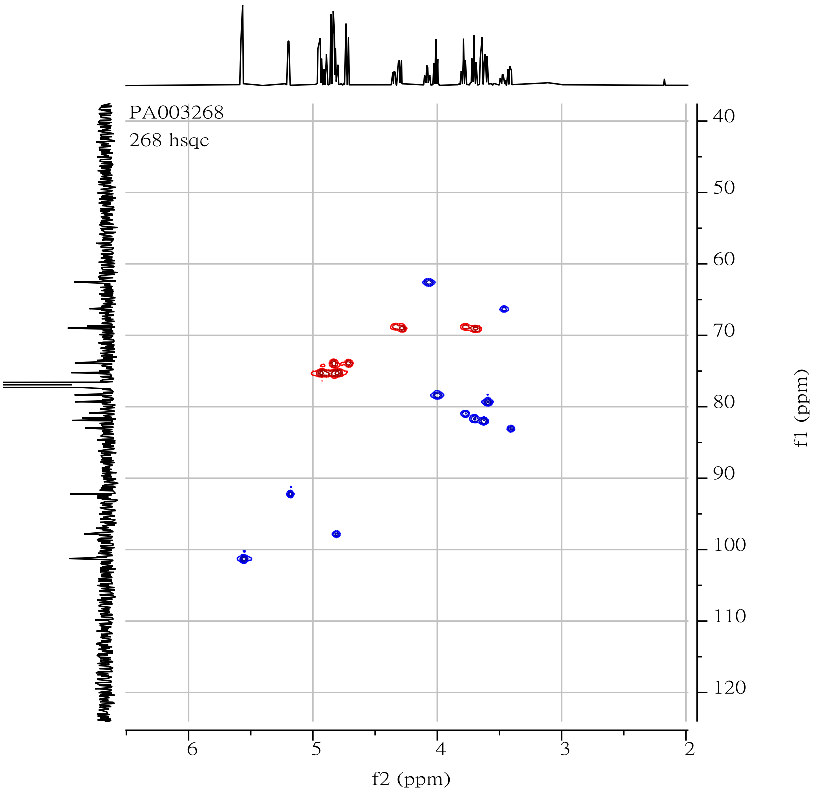

The ^{1}H-^{13}C HSQCed Spectrum in $CDCl_3$ of Compound Glc-OH

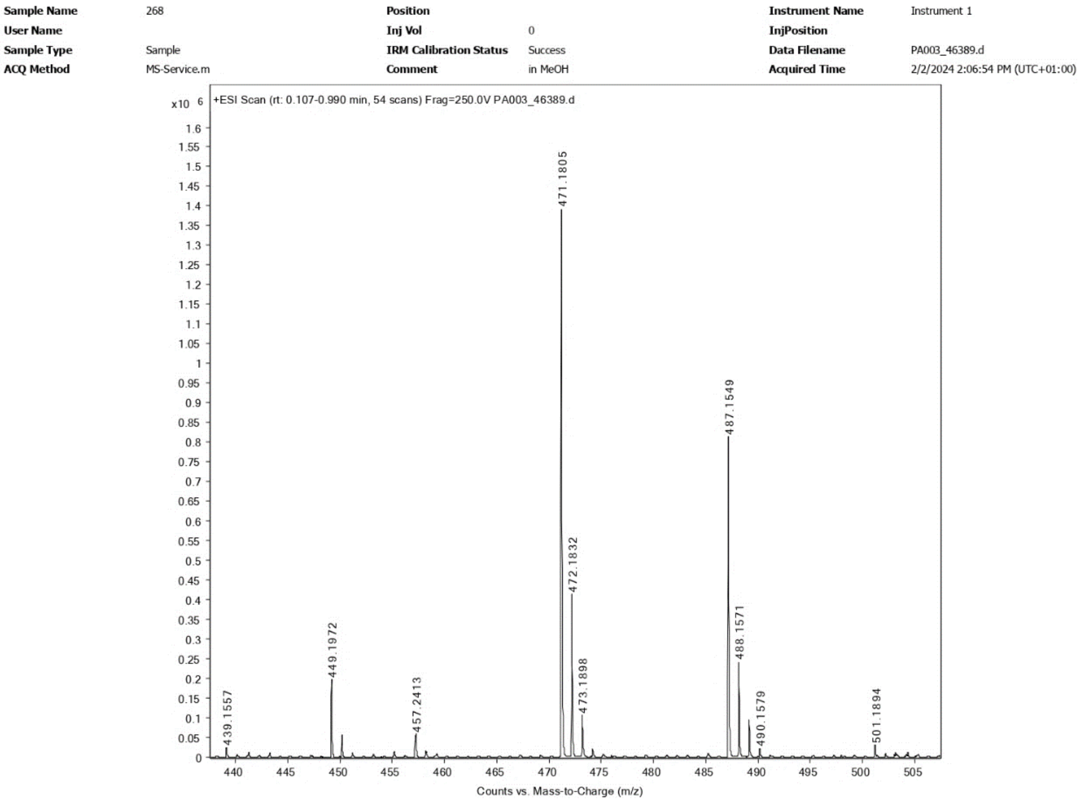

The High-Resolution Mass Spectrometry of Compound Glc-OH

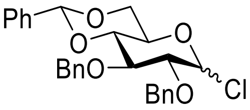

Glc-Cl
(α/β = 3/1)

2,3-di-*O*-Benzyl-4,6-*O*-benzylidene-D-glucopyranosyl chloride

Following the procedures described in the literature,[17,18] hydrolyzed glycoside **Glc-OH** (61.8 mg, 0.138 mmol, 1.0 equiv.) and Et_3N (92 uL, 0.661 mmol, 4.8 equiv.) were mixed and then stirred at 0 °C in CH_2Cl_2 (1.0 mL) for 30 min. MsCl (64 uL, 0.828 mmol, 6.0 equiv.) was injected into mixture at 0 °C. The reaction is stirred at room temperature overnight. The solvent volatilized under nitrogen flow, and the reaction mixture was purified by column chromatography on silica gel to give product glycosyl chloride intermediate **Glc-Cl** (32.4 mg) in 50 % yield. ^{1}H NMR (600 MHz, CHLOROFORM-*D*) δ 7.53 – 7.46 (m, 8H), 7.43 – 7.27 (m, 47H), 5.99 (d, *J* = 4.0 Hz, 3H), 5.59 (s, 1H), 5.56 (s, 3H), 5.30 (d, *J* = 8.2 Hz, 1H), 5.10 – 4.57 (m, 16H), 4.40 (dd, *J* = 10.5, 5.0 Hz, 1H), 4.32 (dd, *J* = 10.3, 4.9 Hz, 3H), 4.18 (td, *J* = 10.0, 4.9 Hz, 3H), 4.09 (t, *J* = 9.2 Hz, 3H), 3.89 – 3.77 (m, 3H), 3.77 – 3.71 (m, 6H), 3.71 – 3.62 (m, 4H), 3.53 (ddd, *J* = 10.0, 9.0, 5.0 Hz, 1H). ^{13}C NMR (151 MHz, CHLOROFORM-*D*) δ 138.37, 138.07, 137.54, 137.39, 137.03, 136.96, 129.07, 129.05,

128.56, 128.43, 128.39, 128.33, 128.28, 128.26, 128.23, 128.12, 128.05, 128.02, 128.00, 127.82, 127.71, 126.02, 125.96, 101.41, 101.24, 93.42, 90.43, 84.35, 81.49, 81.02, 80.93, 79.19, 77.81, 77.21, 77.00, 76.79, 76.05, 75.44, 75.10, 73.40, 69.46, 68.40, 68.30, 65.26, 29.69. HRMS (ESI) calcd for $C_{27}H_{28}O_5Cl$ [M + Na]+ 489.1445, found 489.1391.

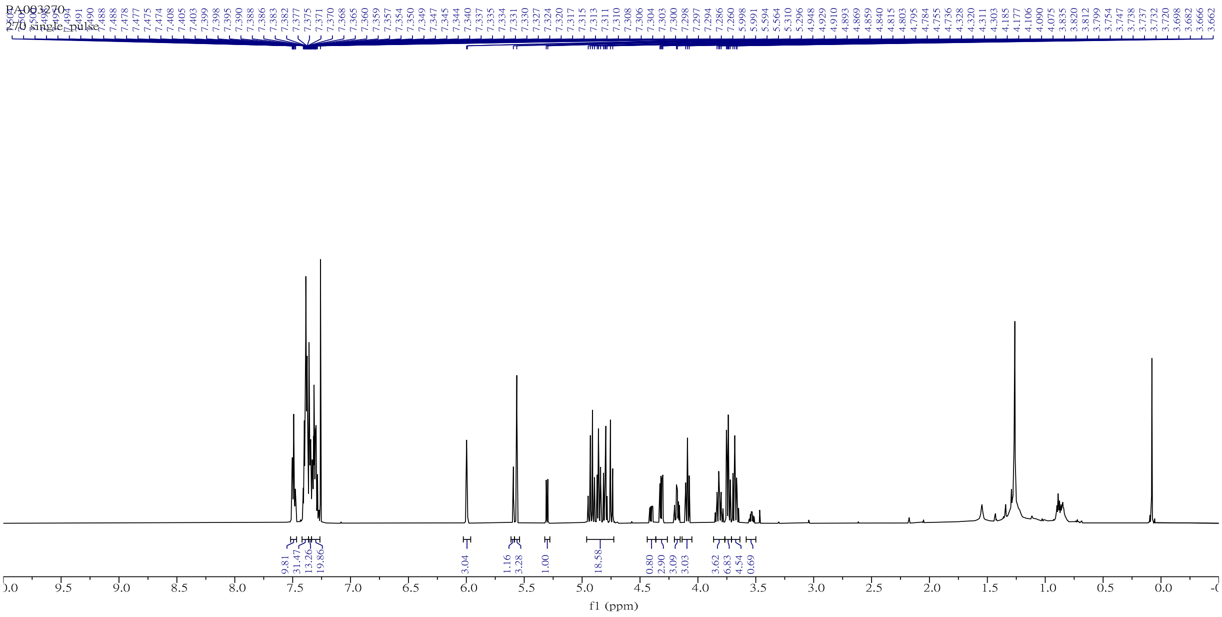

The ^{1}H Spectrum in $CDCl_3$ from 10.0 ppm to -0.5 ppm of Compound Glc-Cl

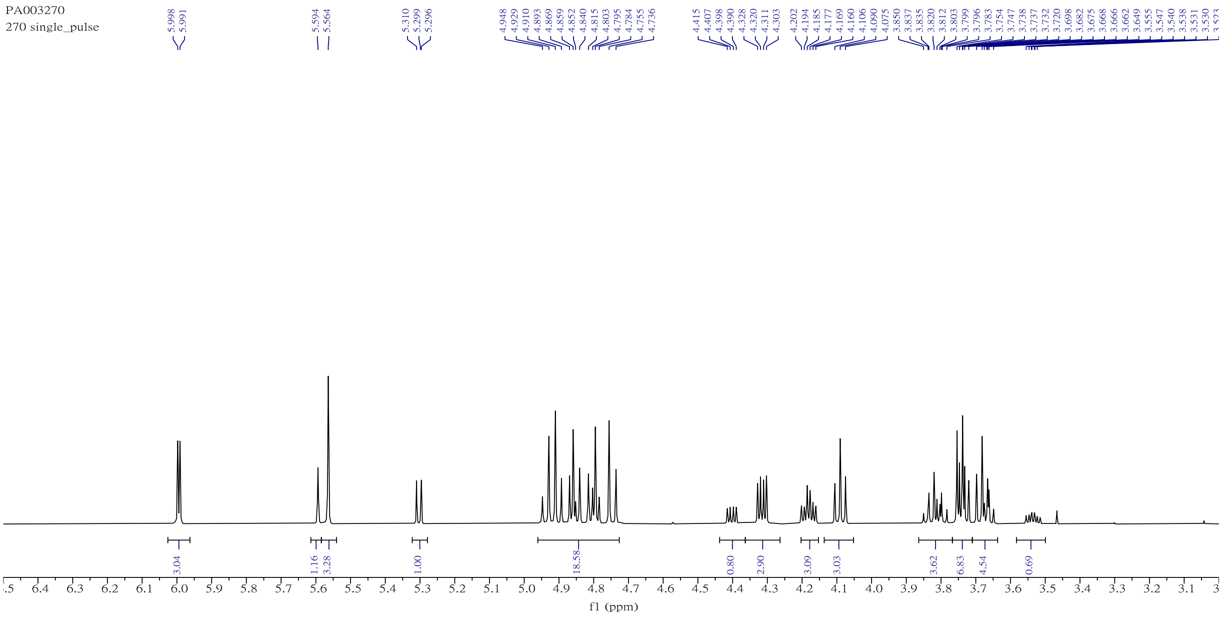

The ^{1}H Spectrum in $CDCl_3$ from 6.0 ppm to -0.5 ppm of Compound Glc-Cl

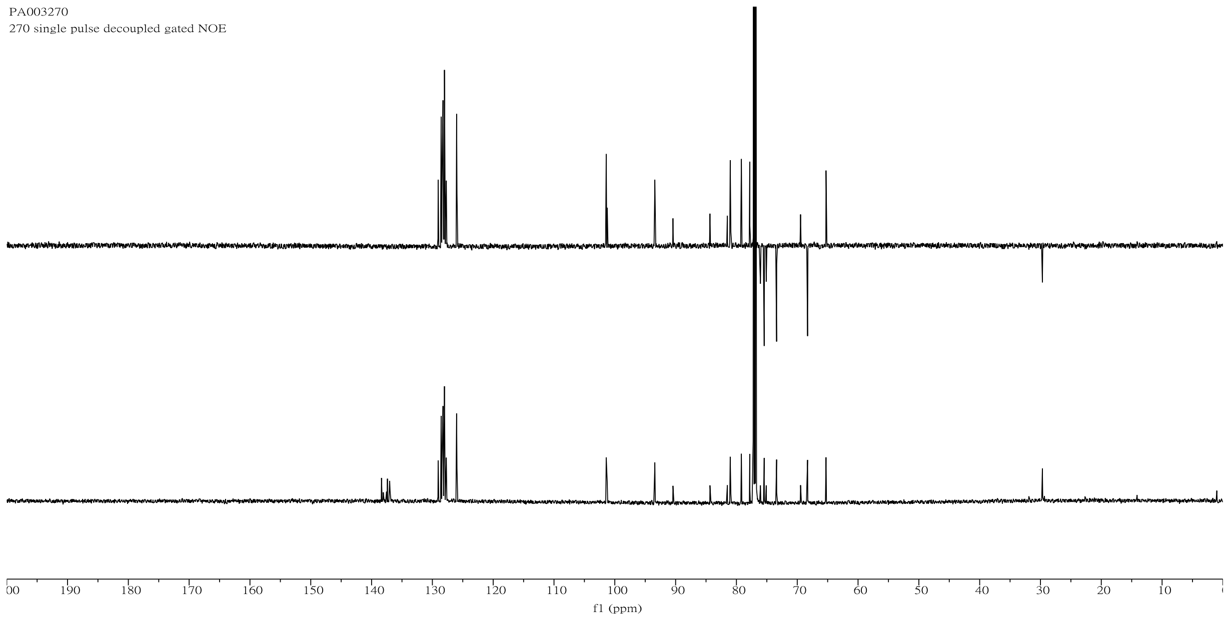

The ^{13}C and DEPT135 Spectrum in $CDCl_3$ of Compound Glc-Cl

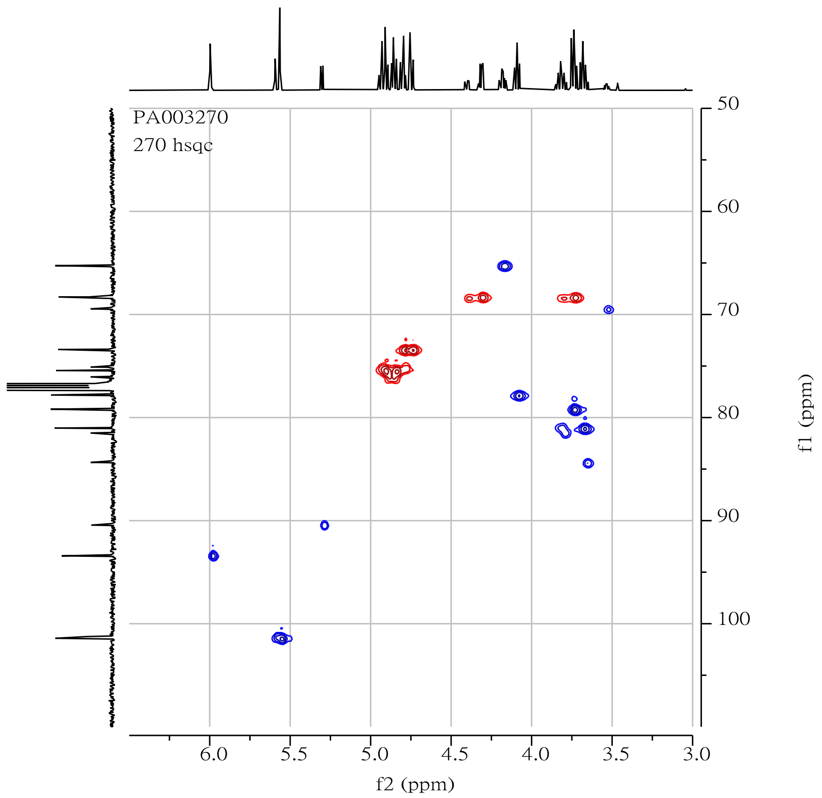

The ^{1}H-^{13}C HSQCed Spectrum in $CDCl_3$ of Compound Glc-Cl

Sample Name	27	Position		Instrument Name	Instrument 1
User Name		Inj Vol	0	InjPosition	
Sample Type	Sample	IRM Calibration Status	Success	Data Filename	PA003_270.d
ACQ Method	MS-Service.m	Comment	in MeOH	Acquired Time	2/12/2024 1:16:53 PM (UTC+01:00)

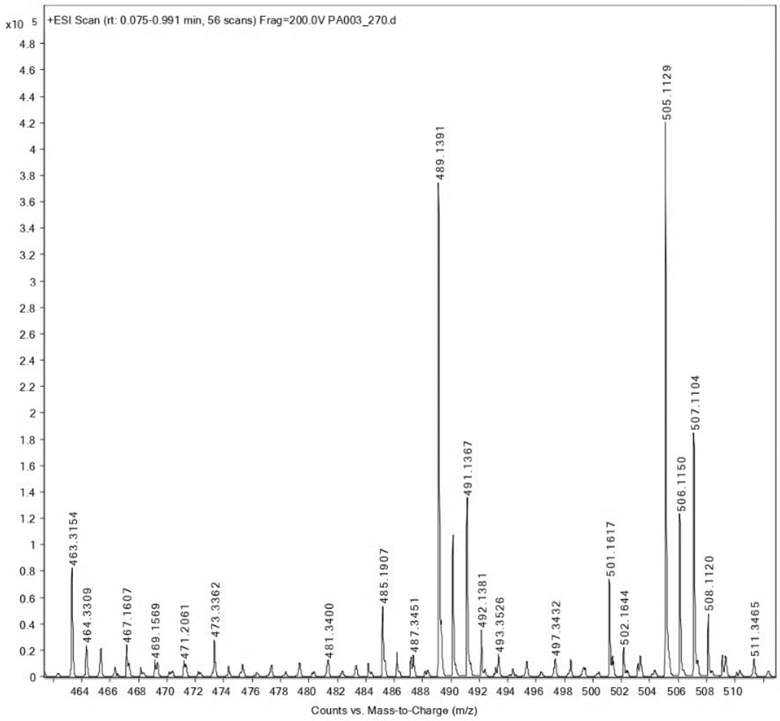

The High-Resolution Mass Spectrometry of Compound Glc-Cl

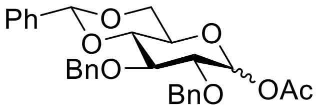

Glc-OAc

(α/β = 1/2)

1-Acetyl 2,3-di-*O*-benzyl-4,6-*O*-benzylidene-D-glucopyranoside

Following the procedures described in the literature,[17,18] hydrolyzed glycoside **Glc-OH** (32 mg) was dissolved in mixture of pyridine/Ac_2O (1:4 v/v, 1 mL) at room temperature. The reaction was concentrated for the following extraction after stirring for 10 hr. The concentrated solution was extracted by H_2O and EtOAc. The solution of extract was added MgSO4 and filtered. The desired product was purified by column chromatography **Glc-OAc** (30.8 mg) in 88% yield. ^{1}H NMR (600 MHz, CHLOROFORM-*D*) δ 7.53 – 7.47 (m, 6H), 7.43 – 7.24 (m, 32H), 6.28 (d, *J* = 3.8 Hz, 1H), 5.72 (d, *J* = 8.0 Hz, 2H), 5.58 (d, *J* = 1.5 Hz, 3H), 4.95 (dd, *J* = 11.3, 1.2 Hz, 3H), 4.88 – 4.78 (m, 5H), 4.76 – 4.69 (m, 4H), 4.38 (dd, *J* = 10.4, 5.0 Hz, 2H), 4.30 (dd, *J* = 10.3, 4.9 Hz, 1H), 4.03 (t, *J* = 9.3 Hz, 1H), 3.93 (td, *J* = 9.9, 4.9 Hz, 1H), 3.89 – 3.83 (m, 2H), 3.78 – 3.66 (m, 7H), 3.62 – 3.54 (m, 4H), 2.18 (s, 3H), 2.06 (s, 5H). ^{13}C NMR (151 MHz, CHLOROFORM-*D*) δ 169.51, 169.05, 138.53, 138.22, 137.97, 137.54, 137.15, 137.10, 129.01, 128.98, 128.47, 128.40, 128.35, 128.31, 128.25, 128.24, 128.05, 128.02, 127.99, 127.90, 127.83, 127.75, 127.64, 125.99, 125.96, 101.27,

101.24, 94.09, 90.40, 81.52, 81.29, 81.11, 80.65, 78.29, 78.07, 77.21, 77.00, 76.79, 75.39, 75.25, 75.14, 73.71, 68.75, 68.51, 66.65, 64.74, 29.68, 21.07, 20.87. HRMS (ESI) calcd for $C_{29}H_{30}NaO_7$ [M + Na]+ 513.1889, found 513.1906.

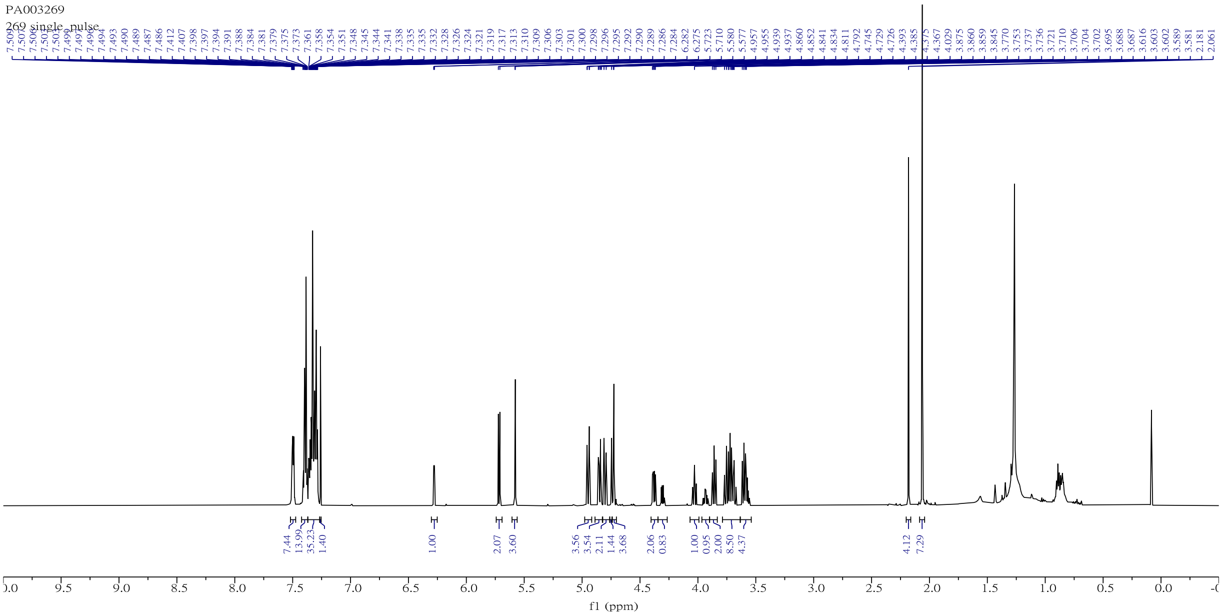

The [1]H Spectrum in $CDCl_3$ from 10.0 ppm to -0.5 ppm of Compound Glc-OAc

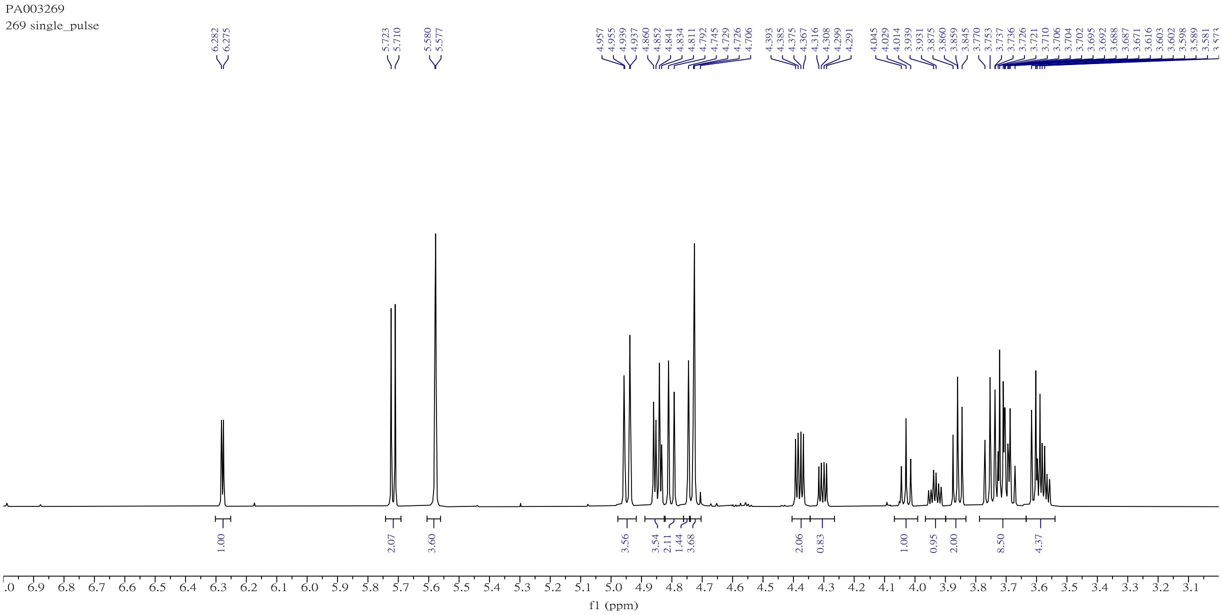

The [1]H Spectrum in $CDCl_3$ from 7.0 ppm to 3.0 ppm of Compound Glc-TCAI

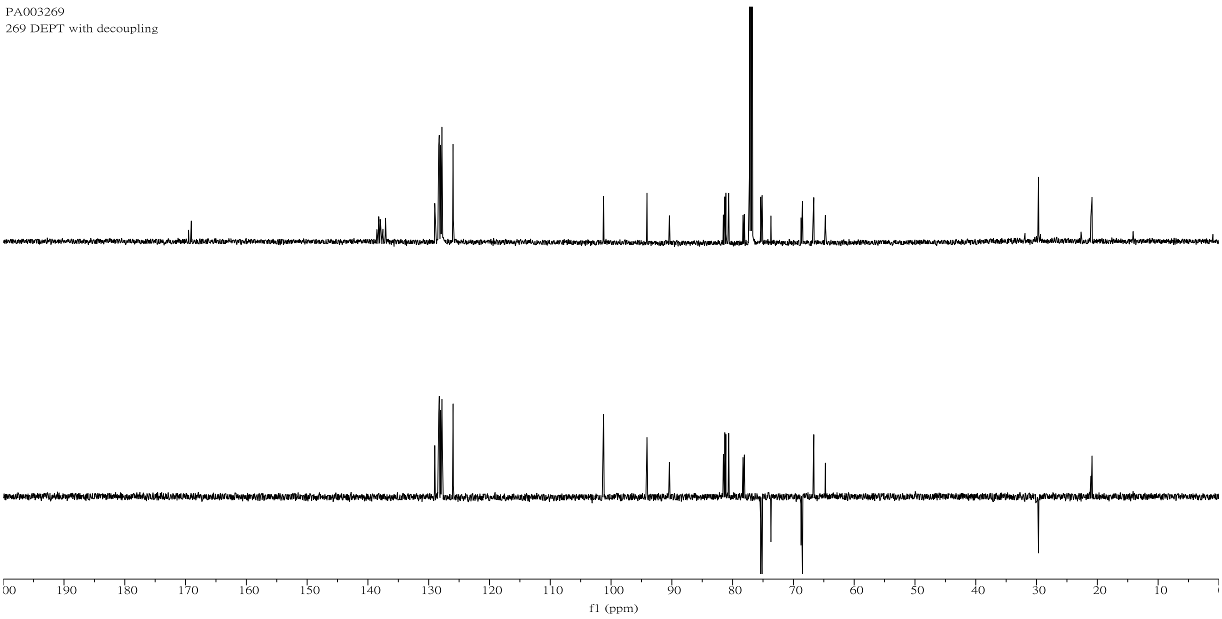

The ^{13}C and DEPT135 Spectrum in $CDCl_3$ of Compound Glc-OAc

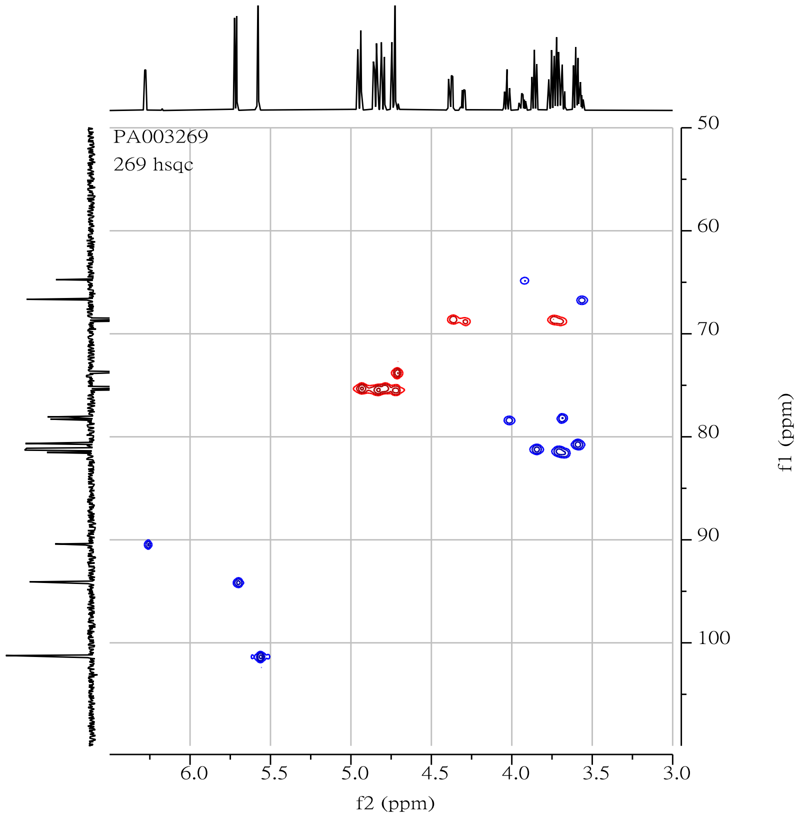

The ^{1}H-^{13}C HSQCed Spectrum in $CDCl_3$ of Compound Glc-OAc

**Sample Name**	269	**Position**		**Instrument Name**	Instrument 1
**User Name**		**Inj Vol**	0	**InjPosition**	
**Sample Type**	Sample	**IRM Calibration Status**	Success	**Data Filename**	PA003_46390.d
**ACQ Method**	MS-Service.m	**Comment**	in MeOH	**Acquired Time**	2/2/2024 2:12:07 PM (UTC+01:00)

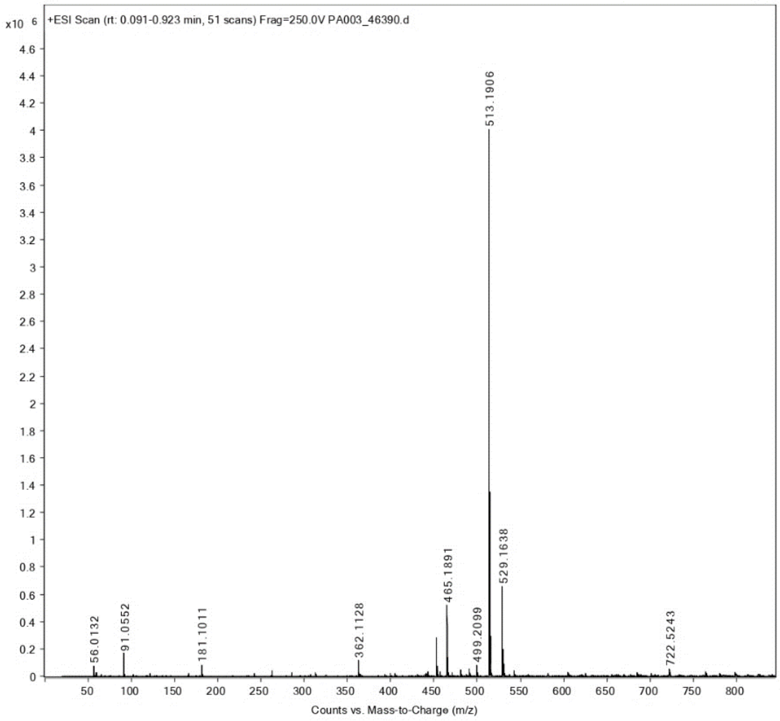

The High-Resolution Mass Spectrometry of Compound Glc-OAc

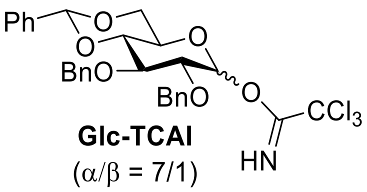

Glc-TCAI
(α/β = 7/1)

2,3-di-*O*-Benzyl-4,6-*O*-benzylidene-D-glucopyranosyl 2,2,2-trichloroacetimidate

Following the procedures described in the literature,[17,18] hydrolyzed glycoside **Glc-OH** (63.2 mg, 0.141 mmol, 1.0 equiv.) and 1,8-Diazabicyclo(5.4.0)undec-7-ene (DBU) (23 uL, 0.155 mmol, 1.1 equiv.) were mixed and then stirred at 0 °C in CH_2Cl_2 (1.0 mL) for 30 min. Trichloroacetonitrile (141 uL, 1.41 mmol, 10.0 equiv.) was injected into mixture at 0 °C. The reaction is stirred at room temperature overnight. The solvent volatilized under nitrogen flow, and the reaction mixture was purified by column chromatography on silica gel to give product glycosyl imidate **Glc-TCAI** (81.7 mg) in 98 % yield. ^{1}H NMR (600 MHz, CHLOROFORM-*D*) δ 8.74 (s, 1H), 8.63 (s, 5H), 7.51 (tt, *J* = 6.9, 1.8 Hz, 13H), 7.45 – 7.05 (m, 81H), 6.45 (d, *J* = 3.7 Hz, 5H), 5.95 (d, *J* = 7.7 Hz, 1H), 5.60 (s, 6H), 5.11 – 4.65 (m, 25H), 4.43 (dd, *J* = 10.5, 5.0 Hz, 1H), 4.34 (dd, *J* = 10.4, 5.0 Hz, 5H), 4.14 (t, *J* = 9.3 Hz, 5H), 4.05 (td, *J* = 10.0, 4.9 Hz, 5H), 3.90 (dd, *J* = 9.1, 8.3 Hz, 1H), 3.87 – 3.80 (m, 2H), 3.81 – 3.69 (m, 16H), 3.64 (td, *J* = 9.8, 5.0 Hz, 1H). ^{13}C NMR (151 MHz, CHLOROFORM-*D*) δ 164.65, 161.36, 161.04, 138.47, 138.25, 137.84, 137.78, 137.09, 129.00, 128.97, 128.36, 128.34,

128.31, 128.26, 128.24, 128.04, 127.97, 127.92, 127.81, 127.75, 127.68, 127.61, 127.57, 126.01, 125.95, 101.23, 98.17, 94.61, 91.06, 81.40, 81.07, 80.86, 80.65, 78.56, 77.95, 77.21, 77.00, 76.79, 75.27, 75.23, 74.98, 73.33, 68.69, 68.59, 66.60, 65.02. HRMS (ESI) calcd for $C_{29}H_{28}Cl_3NO_6$ [M + Na]$^+$ 614.0880, found 614.0789.

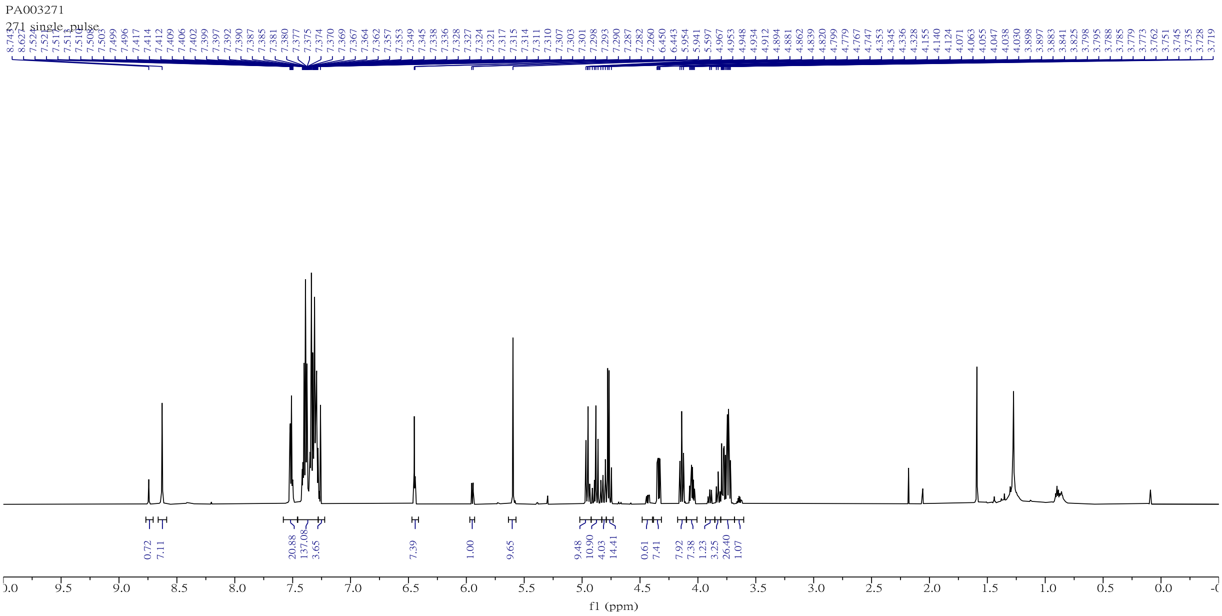

The ^{1}H Spectrum in $CDCl_3$ from 10.0 ppm to -0.5 ppm of Compound Glc-TCAI

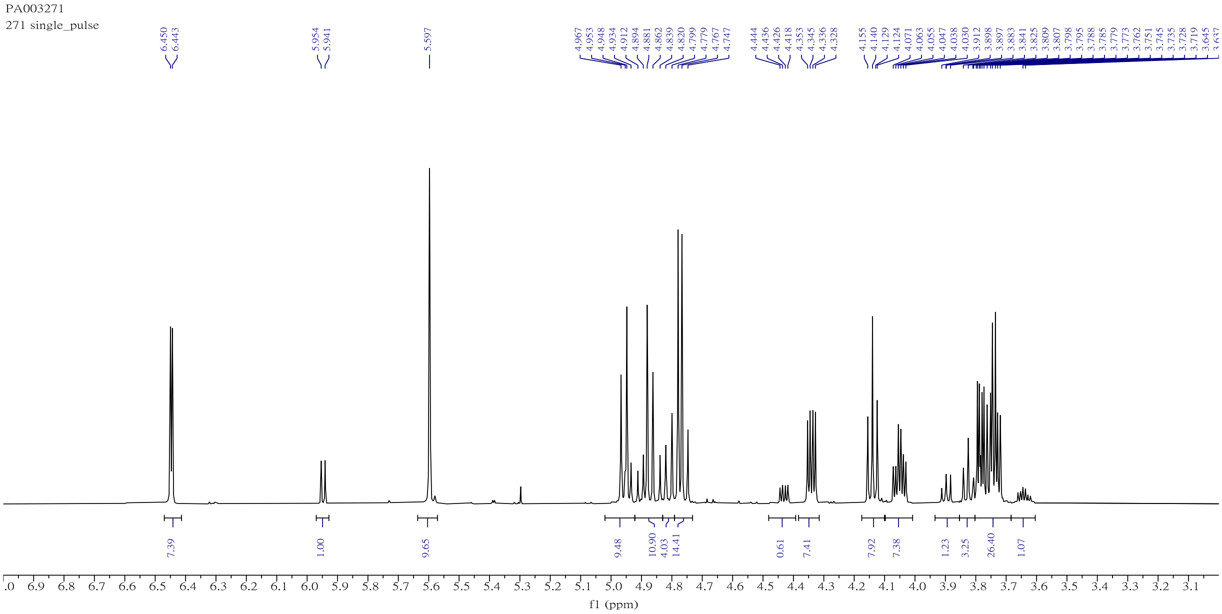

The ^{1}H Spectrum in $CDCl_3$ from 7.0 ppm to 3.0 ppm of Compound Glc-TCAI

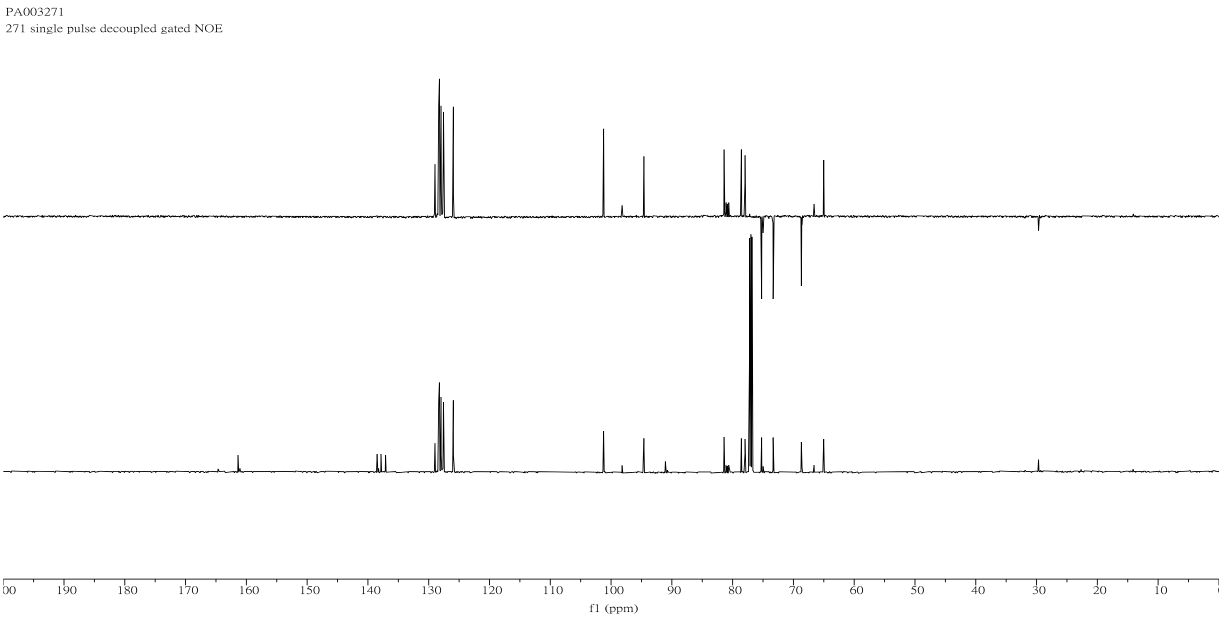

The ^{13}C and DEPT135 Spectrum in $CDCl_3$ of Compound Glc-TCAI

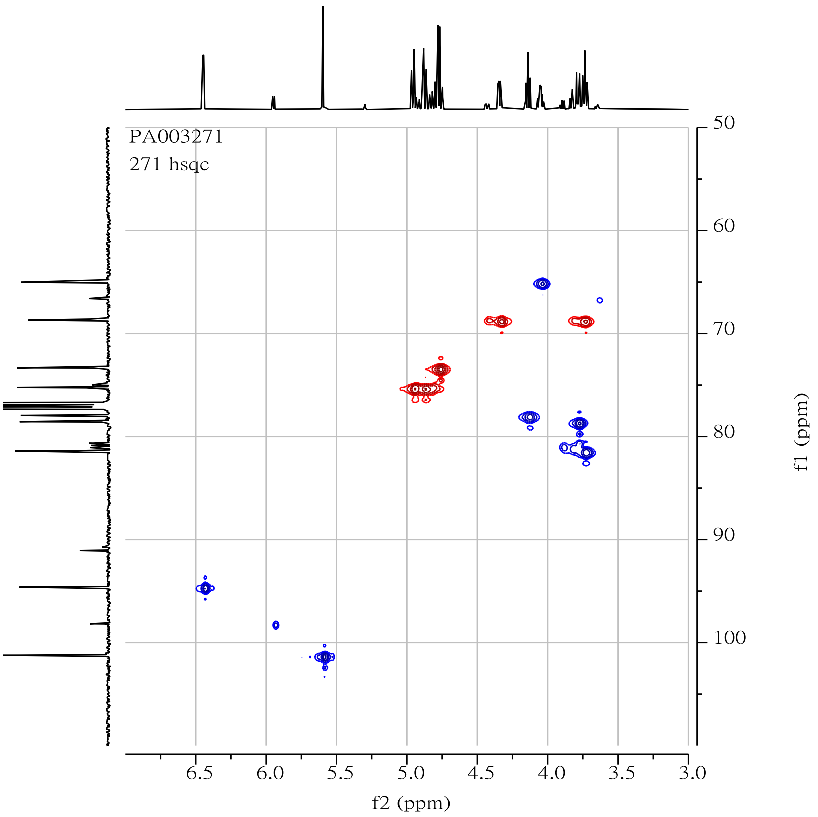

The ^{1}H-^{13}C HSQCed Spectrum in $CDCl_3$ of Compound Glc-TCAI

**Sample Name**	271	**Position**		**Instrument Name**	Instrument 1
**User Name**		**Inj Vol**	0	**InjPosition**	
**Sample Type**	Sample	**IRM Calibration Status**	Success	**Data Filename**	PA003_271.d
**ACQ Method**	MS-Service.m	**Comment**	in MeOH	**Acquired Time**	2/12/2024 1:22:04 PM (UTC+01:00)

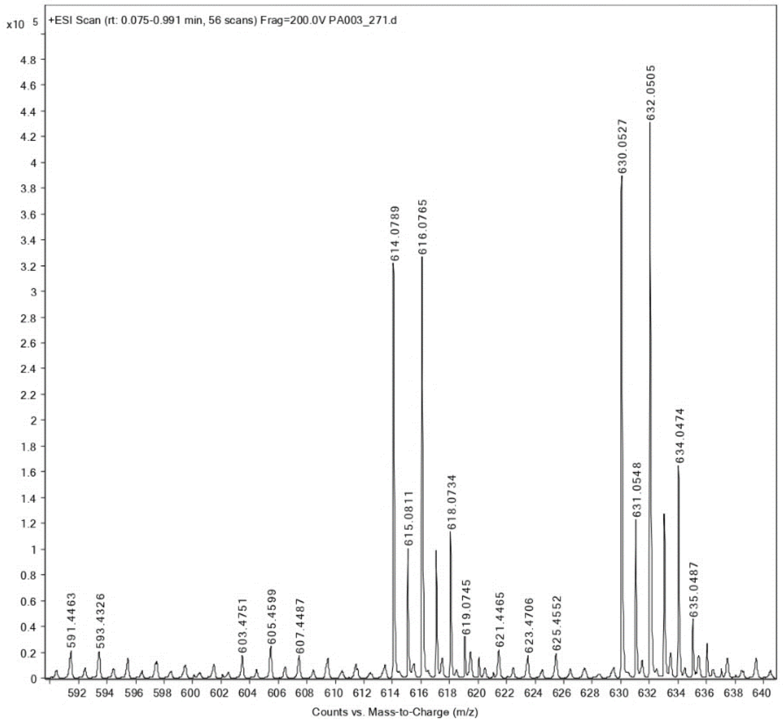

The High-Resolution Mass Spectrometry of Compound Glc-TCAI

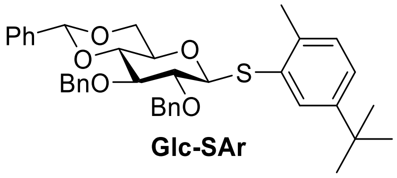

Glc-SAr

5-(1,1-Dimethylethyl)-2-methylphenyl 2,3-di-O-benzyl-4,6-O-benzylidene-D-thio-glucopyranoside

5-(1,1-Dimethylethyl)-2-methylphenyl 4,6-O-benzylidene-D-thio-glucopyranoside (40 mg, 0.093 mmol, 1.0 equiv.), commercially available from GlycoUniverse GmbH & Co., was mixed with BnBr (27 uL, 0.2232mmol, 2.4 equiv.) in DMF (1 mL) and then stirred at 0 °C for 30 mins. After that, 60% NaH within mineral oil (15 mg, 0.372 mmol, 4 equiv.) was added into the mixture slowly at 0 °C. The reaction is warmed up to room temperature and then stirred for overnight. Cold water was added into reaction to quench NaH with icebath. The mixture was washed with H_2O. The aqueous layer was extracted with EtOAc, dried with anhydrous MgSO4, filtered, and concentrated *in vacuo*. The mixture was purified by flash column chromatography (*n*-Hexane/EtOAc 6:1) on silica gel to give product **Glc-SAr** (31.8 mg) in 56% yield. ^{1}H NMR (600 MHz, CHLOROFORM-*D*) δ 7.61 (d, *J* = 2.1 Hz, 1H), 7.52 – 7.46 (m, 2H), 7.43 – 7.25 (m, 15H), 7.21 (dd, *J* = 7.9, 2.1 Hz, 1H), 7.13 (dd, *J* = 7.9, 0.8 Hz, 1H), 5.61 (s, 1H), 4.99 – 4.90 (m, 2H), 4.88 (d, *J* = 10.2 Hz, 1H),

4.81 (d, J = 11.2 Hz, 1H), 4.76 (d, J = 9.9 Hz, 1H), 4.36 (dd, J = 10.5, 5.0 Hz, 1H), 3.89 – 3.80 (m, 2H), 3.78 (t, J = 9.4 Hz, 1H), 3.59 (dd, J = 9.9, 8.3 Hz, 1H), 3.51 – 3.43 (m, 1H), 2.40 (s, 3H), 1.30 (s, 8H). ^{13}C NMR (151 MHz, CHLOROFORM-*D*) δ 149.52, 138.33, 137.98, 137.21, 136.11, 132.74, 129.92, 128.97, 128.91, 128.36, 128.25, 128.21, 128.08, 127.84, 127.77, 127.73, 125.95, 124.73, 101.12, 88.46, 83.01, 81.46, 80.84, 77.21, 77.00, 76.79, 76.03, 75.28, 70.05, 68.73, 34.45, 31.28, 29.69, 20.29, 1.00. HRMS (ESI) calcd for $C_{29}H_{28}Cl_3NO_6$ [M + Na]$^+$ 633.2651, found 633.2692.

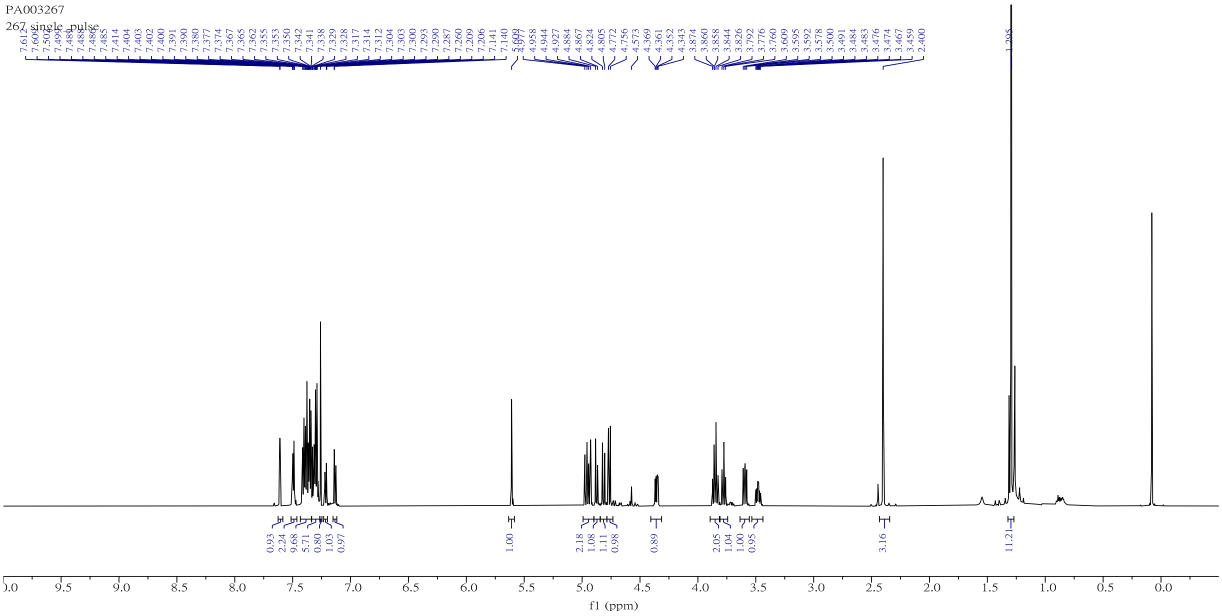

The ^{1}H Spectrum in $CDCl_3$ from 10.0 ppm to -0.5 ppm of Compound Glc-SAr

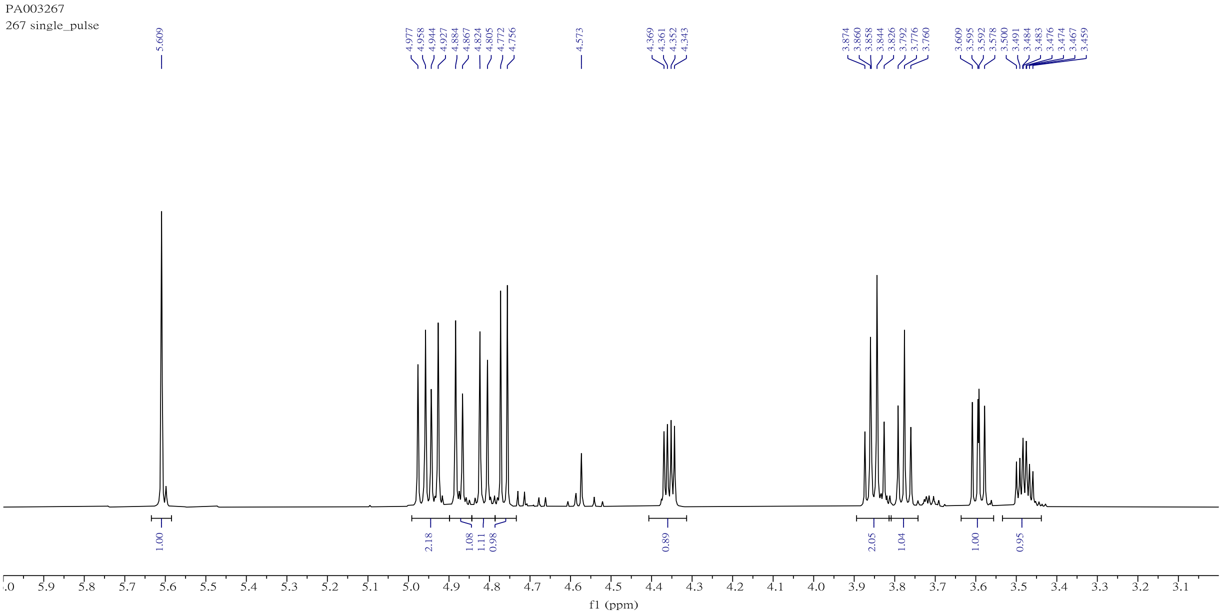

The ^{1}H Spectrum in $CDCl_3$ from 6.0 ppm to 3.0 ppm of Compound Glc-SAr

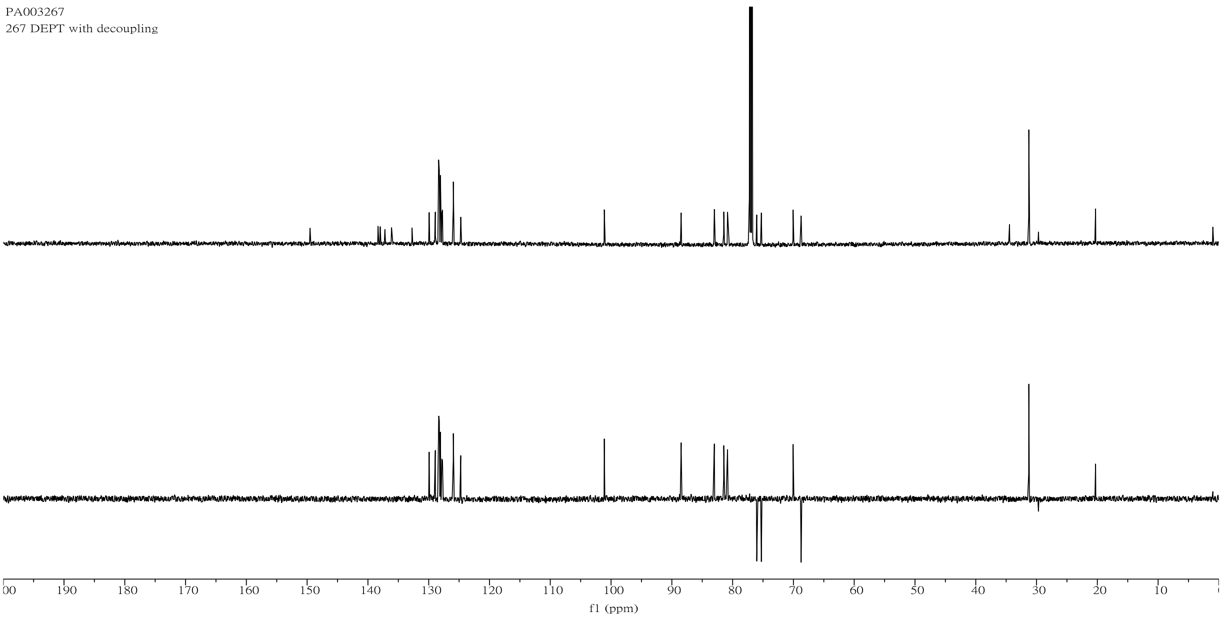

The ¹³C Spectrum in $CDCl_3$ of Compound Glc-SAr

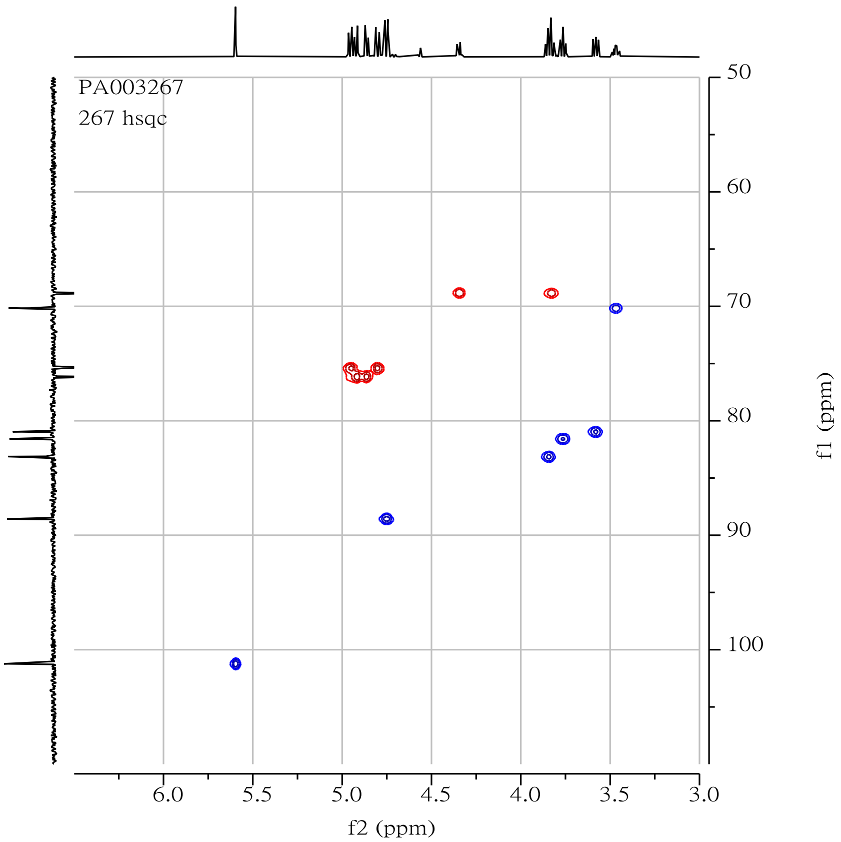

The ¹H-¹³C HSQCed Spectrum in $CDCl_3$ of Compound Glc-SAr

**Sample Name**	267	**Position**		**Instrument Name**	Instrument 1
**User Name**		**Inj Vol**	0	**InjPosition**	
**Sample Type**	Sample	**IRM Calibration Status**	Success	**Data Filename**	PA003_46388_.d
**ACQ Method**	MS-Service.m	**Comment**	in MeOH	**Acquired Time**	2/2/2024 1:37:56 PM (UTC+01:00)

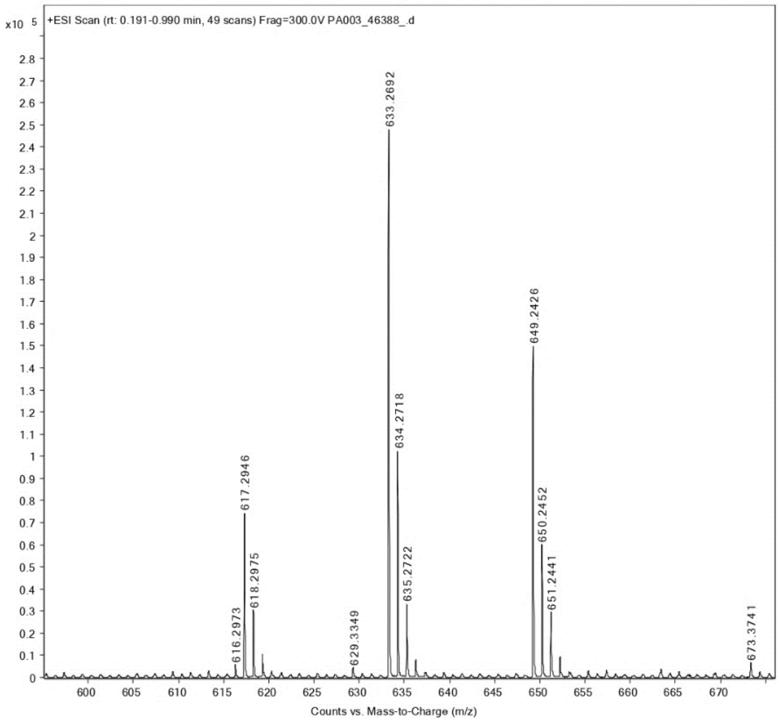

The High-Resolution Mass Spectrometry of Compound Glc-TCAI

6.2 Probing the Stereoselectivity of 4,6-*O*-Benzylidene-Directed Glycosylation Reaction

Table S7. Glycosylation Reaction in NIS/TfOH promotor System.

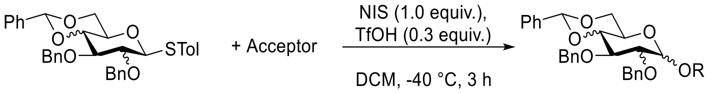

Entry	Donor	Acceptor	Product	Yield[a] (α/β)[b]	α-ratio (%)
1	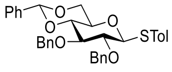 Glc	**MeOH**	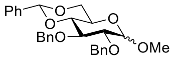	79% (1/4)	25%α
2	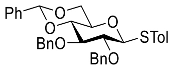 Glc	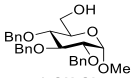 6-OH Glc	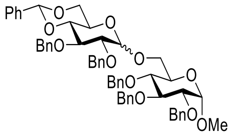	70% (1/1)	50%α
3	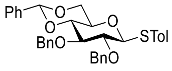 Glc	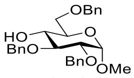 4-OH Glc	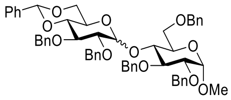	60% (1/1)	50%α
4	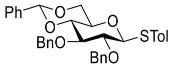 Glc	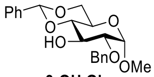 3-OH Glc	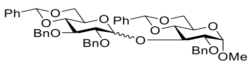	44% (1/1)	50%α
5	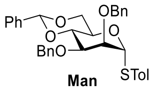 Man	**MeOH**	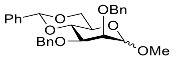	70% (1/5)	17%α
6	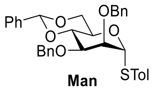 Man	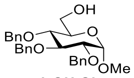 6-OH Glc	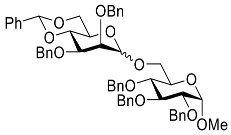	66% (1/2)	33%α
7	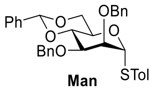 Man	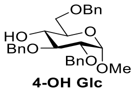 4-OH Glc	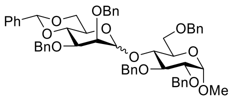	58% (1/1.4)	42%α
8	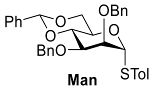 Man	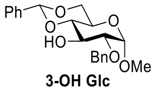 3-OH Glc	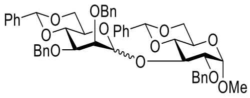	45%(1/1.7)	37%α
9	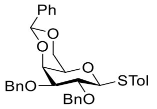 Gal	**MeOH**	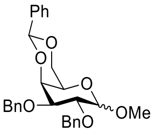	53% (1/4)	20%α

10	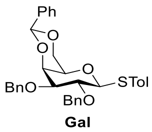<br>**Gal**	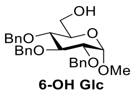<br>**6-OH Glc**	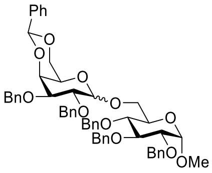	85% (1/1.5)	40%α
11	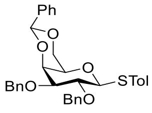<br>**Gal**	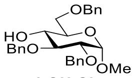<br>**4-OH Glc**	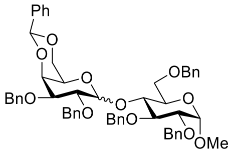	64% (4/1)	80%α
12	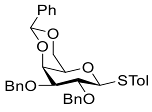<br>**Gal**	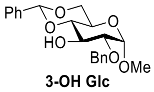<br>**3-OH Glc**	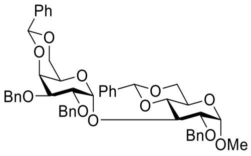	73% (1/0)	100%α

[a]Isolated yield. [b] Determined by HPLC. The examples of glycosylation reaction in condensed phase is obtained from the report by Wang et al..[17,20]

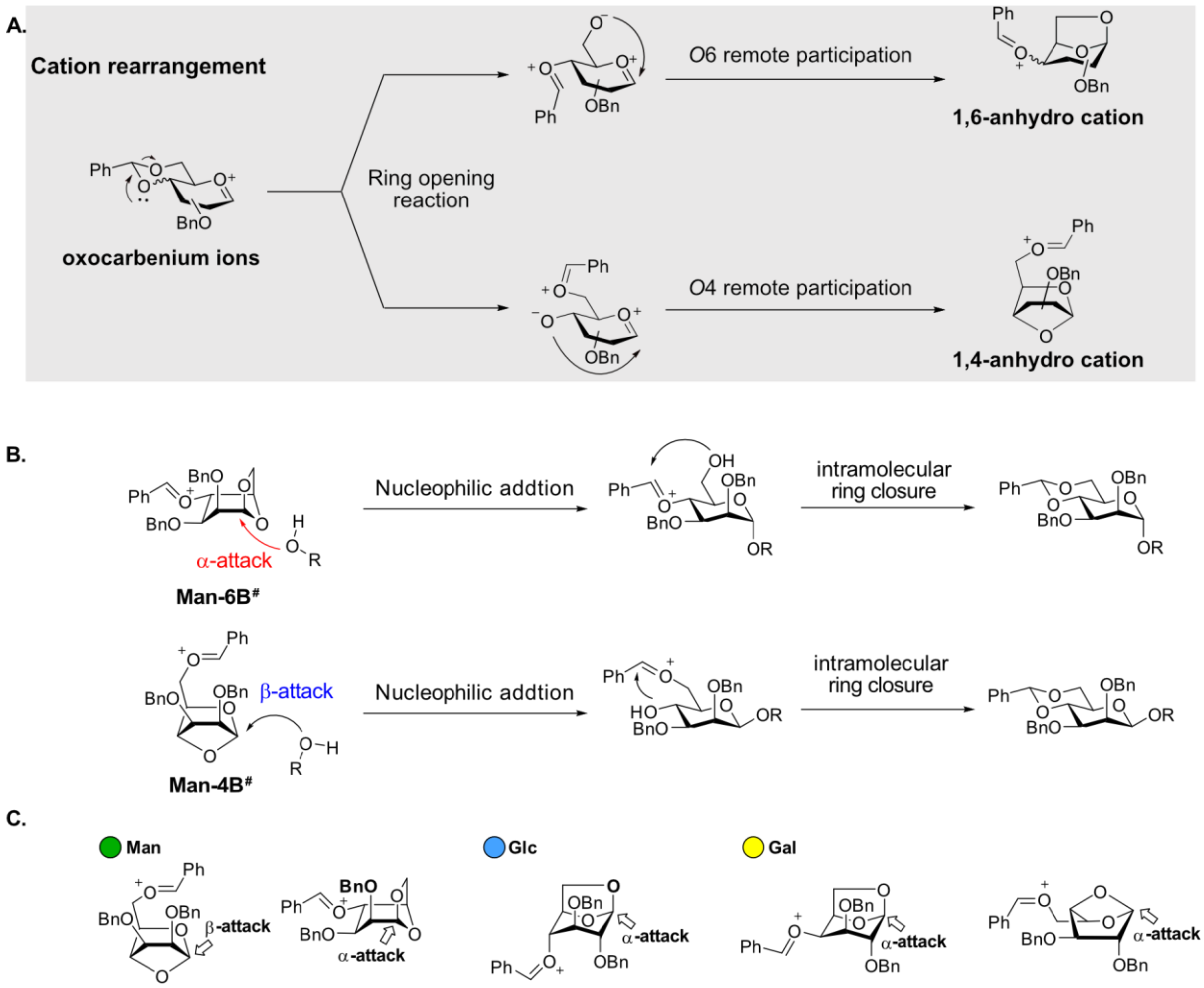

Figure S45. Hypothetical S_N1-mechanism of 4,6-benzylidene-mediated glycosylation of the gas-phase intermediates. A) Activation of glycosyl donors leads to oxocarbenium ions, which subsequently undergoes a ring opening reaction of the benzylidene acetal at O4 and O6.[21] Next, remote participation[22] of the ether at O6 or O4 occurs to give the energetically more stable 1,6-anhydro or 1,4-anhydro cations, as confirmed by cryogenic IR spectroscopy. B) For mannosylations, both **Man-6B** and **Man-4B** can be generated and have inverse selectivities, potentially explaining the mixed ratios observed in its S_N1-type glycosylations. **Man-4B** promotes the attack of the acceptor from the β-face, whereas **Man-6B** blocks the upper side, resulting in α-nucleophilic addition. C) The inverse selectivities of the anhydro cations correlates well with the mixed anomer ratios that are often observed when benzylidene-protected donors are used under conditions that favor an S_N1-type glycosylation reaction.

References

1 Schöllkopf, W., Gewinner, S., Junkes, H., Paarmann, A., von Helden, G., Bluem, H. P. & Todd, A. M. M. The new IR and THz FEL facility at the Fritz Haber Institute in Berlin. *Proc. SPIE Int. Soc. Opt. Eng.* **9512**, 95121L (2015).

2 Greis, K., Leichnitz, S., Kirschbaum, C., Chang, C.-W., Lin, M.-H., Meijer, G., von Helden, G., Seeberger, P. H. & Pagel, K. The Influence of the Electron Density in Acyl Protecting Groups on the Selectivity of Galactose Formation. *J. Am. Chem. Soc.* **144**, 20258-20266 (2022).

3 Gaussian 16, Revision C.01, M. J. Frisch, G. W. Trucks, H. B. Schlegel, G. E. Scuseria, M. A. Robb, J. R. Cheeseman, G. Scalmani, V. Barone, G. A. Petersson, H. Nakatsuji, X. Li, M. Caricato, A. V. Marenich, J. Bloino, B. G. Janesko, R. Gomperts, B. Mennucci, H. P. Hratchian, J. V. Ortiz, A. F. Izmaylov, J. L. Sonnenberg, D. Williams-Young, F. Ding, F. Lipparini, F. Egidi, J. Goings, B. Peng, A. Petrone, T. Henderson, D. Ranasinghe, V. G. Zakrzewski, J. Gao, N. Rega, G. Zheng, W. Liang, M. Hada, M. Ehara, K. Toyota, R. Fukuda, J. Hasegawa, M. Ishida, T. Nakajima, Y. Honda, O. Kitao, H. Nakai, T. Vreven, K. Throssell, J. A. Montgomery, Jr., J. E. Peralta, F. Ogliaro, M. J. Bearpark, J. J. Heyd, E. N. Brothers, K. N. Kudin, V. N. Staroverov, T. A. Keith, R. Kobayashi, J. Normand, K. Raghavachari, A. P. Rendell, J. C. Burant, S. S. Iyengar, J. Tomasi, M. Cossi, J. M. Millam, M. Klene, C. Adamo, R. Cammi, J. W. Ochterski, R. L. Martin, K. Morokuma, O. Farkas, J. B. Foresman, and D. J. Fox, Gaussian, Inc., Wallingford CT. (2016).

4 Pracht, P., Bohle, F. & Grimme, S. Automated Exploration of the Low-energy Chemical Space with Fast Quantum Chemical Methods. *Phys. Chem. Chem. Phys.* **22**, 7169-7192 (2020).

5 Bannwarth, C., Ehlert, S. & Grimme, S. GFN2-xTB—An Accurate and Broadly Parametrized Self-Consistent Tight-Binding Quantum Chemical Method with Multipole Electrostatics and Density-Dependent Dispersion Contributions. *J. Chem. Theory Comput.* **15**, 1652-1671 (2019).

6 Adamo, C. & Barone, V. Toward reliable density functional methods without adjustable parameters: The PBE0 model. *J. Chem. Phys.* **110**, 6158-6170 (1999).

7 Grimme, S., Antony, J., Ehrlich, S. & Krieg, H. A consistent and accurate ab initio parametrization of density functional dispersion correction (DFT-D) for the 94 elements H-Pu. *J. Chem. Phys.* **132**, 154104 (2010).

8 Klamt, A. & Schüürmann, G. COSMO: a new approach to dielectric screening in solvents with explicit expressions for the screening energy and its gradient. *J. Chem. Soc., Perkin Trans. 2*, 799-805 (1993).

9 Warnke, S., Seo, J., Boschmans, J., Sobott, F., Scrivens, J. H., Bleiholder, C., Bowers, M. T., Gewinner, S., Schollkopf, W., Pagel, K. & von Helden, G. Protomers of Benzocaine: Solvent and Permittivity Dependence. *J. Am. Chem. Soc.* **137**, 4236-4242 (2015).

10 Greis, K., Mucha, E., Lettow, M., Thomas, D. A., Kirschbaum, C., Moon, S., Pardo-Vargas,

A., von Helden, G., Meijer, G., Gilmore, K., Seeberger, P. H. & Pagel, K. The Impact of Leaving Group Anomericity on the Structure of Glycosyl Cations of Protected Galactosides. *ChemPhysChem* **21**, 1905-1907 (2020).

11 Hosoya, T., Takano, T., Kosma, P. & Rosenau, T. Theoretical foundation for the presence of oxacarbenium ions in chemical glycoside synthesis. *J. Org. Chem.* **79**, 7889-7894 (2014).

12 Hosoya, T., Kosma, P. & Rosenau, T. Theoretical study on the effects of a 4,6-O-diacetal protecting group on the stability of ion pairs from d-mannopyranosyl and d-glucopyranosyl triflates. *Carbohydr. Res.* **411**, 64-69 (2015).

13 Franconetti, A., Ardá, A., Asensio, J. L., Blériot, Y., Thibaudeau, S. & Jiménez-Barbero, J. Glycosyl Oxocarbenium Ions: Structure, Conformation, Reactivity, and Interactions. *Acc. Chem. Res.* **54**, 2552-2564 (2021).

14 Santana, A. G., Montalvillo-Jimenez, L., Diaz-Casado, L., Corzana, F., Merino, P., Canada, F. J., Jimenez-Oses, G., Jimenez-Barbero, J., Gomez, A. M. & Asensio, J. L. Dissecting the Essential Role of Anomeric b-Triflates in Glycosylation Reactions. *J. Am. Chem. Soc.* **142**, 12501-12514 (2020).

15 Fuster, F., Sevin, A. & Silvi, B. Topological Analysis of the Electron Localization Function (ELF) Applied to the Electrophilic Aromatic Substitution. *J. Phys. Chem. A* **104**, 852-858 (2000).

16 Li, Y., Lin, M., Tian, M., Ye, G. & Zhao, X. DFT computational and spectroscopic studies on andrographolide from different solvent effect. *J. Mol. Liq.* **390**, 123059 (2023).

17 Chang, C.-W., Wu, C.-H., Lin, M.-H., Liao, P.-H., Chang, C.-C., Chuang, H.-H., Lin, S.-C., Lam, S., Verma, V. P., Hsu, C.-P. & Wang, C.-C. Establishment of Guidelines for the Control of Glycosylation Reactions and Intermediates by Quantitative Assessment of Reactivity. *Angew. Chem. Int. Ed.* **58**, 16775-16779 (2019).

18 Chang, C.-W., Lin, M.-H., Chiang, T.-Y., Wu, C.-H., Lin, T.-C. & Wang, C.-C. Unraveling the Promoter Effect and the Roles of Counterion Exchange in Glycosylation Reaction. *Sci. Adv.* **9**, eadk0531 (2023).

19 Crich, D., de la Mora, M. & Vinod, A. U. Influence of the 4,6-O-Benzylidene, 4,6-O-Phenylboronate, and 4,6-O-Polystyrylboronate Protecting Groups on the Stereochemical Outcome of Thioglycoside-Based Glycosylations Mediated by 1-Benzenesulfinyl Piperidine/Triflic Anhydride and N-Iodosuccinimide/Trimethylsilyl Triflate. *J. Org. Chem.* **68**, 8142-8148 (2003).

20 Chang, C.-W., Lin, M.-H., Chan, C.-K., Su, K.-Y., Wu, C.-H., Lo, W.-C., Lam, S., Cheng, Y.-T., Liao, P.-H., Wong, C.-H. & Wang, C.-C. Automated Quantification of Hydroxyl Reactivities: Prediction of Glycosylation Reactions. *Angew. Chem. Int. Ed.* **60**, 12413-12423 (2021).

21 Kirschbaum, C., Greis, K., Polewski, L., Gewinner, S., Schöllkopf, W., Meijer, G., von Helden, G. & Pagel, K. Unveiling Glycerolipid Fragmentation by Cryogenic Infrared

Spectroscopy. *J. Am. Chem. Soc.* **143**, 14827-14834 (2021).

22 Marianski, M., Mucha, E., Greis, K., Moon, S., Pardo, A., Kirschbaum, C., Thomas, D. A., Meijer, G., von Helden, G., Gilmore, K., Seeberger, P. H. & Pagel, K. Remote Participation during Glycosylation Reactions of Galactose Building Blocks: Direct Evidence from Cryogenic Vibrational Spectroscopy. *Angew. Chem. Int. Ed.* **59**, 6166-6171 (2020).
