# Supplementary material for: Mechanistic insight into benzylidene-directed glycosylation reactions using cryogenic infrared spectroscopy
Source: Nat Synth. 2024 Jul 26;3(11):1377–84. doi: 10.1038/s44160-024-00619-0 (PMC11549046; doi:10.1038/s44160-024-00619-0)
Supplement: Supplementary file 2 — SI_coordinated_gas_phase.pdf. [file 44160_2024_619_MOESM2_ESM.pdf]

Glc-oxo/conf_1.out

Charge=+1, Multiplicity=+1

C	1.15699200	-1.32277700	-0.80518400
O	0.31625200	-1.82241300	0.29629400
C	-0.73143900	-1.16807800	0.55006100
H	-1.25432300	-1.48201100	1.45314500
C	-1.11008300	0.02268000	-0.28383300
C	0.11372000	0.95300100	-0.51308500
O	0.21338100	1.93843800	0.47058400
C	-0.47692200	3.15871600	0.17691700
C	-1.97138600	2.99656700	0.15019800
C	-2.66594900	2.98092500	-1.05752800
C	-4.03800200	2.74645100	-1.08183700
C	-4.72546000	2.52322100	0.10546900
C	-4.04082200	2.54866800	1.31883800
C	-2.67294600	2.78736200	1.34009300
H	-2.14041000	2.81454900	2.28653700
H	-4.57929300	2.40239700	2.24944500
H	-5.79693000	2.35307200	0.08924200
H	-4.57090700	2.75135900	-2.02691800
H	-2.13248100	3.15845300	-1.98712300
H	-0.16902400	3.84151300	0.97020500
H	-0.11474300	3.55326900	-0.77995300
H	-0.03694800	1.37759400	-1.51411600
C	1.40234800	0.15191900	-0.51047700
O	2.28940900	0.65458500	-1.46974800
C	3.51911100	-0.05454700	-1.49048200
C	4.34891800	0.18785500	-0.24716200
C	4.21296800	1.37225500	0.47443000
C	5.02220100	1.61379200	1.57791100
C	5.97639900	0.67905100	1.96225700
C	6.12468600	-0.49665200	1.23523400
C	5.31628400	-0.74102800	0.13223400
H	5.44095900	-1.65151700	-0.44481700
H	6.87679700	-1.22316000	1.52309100
H	6.61000400	0.87039300	2.82141400
H	4.91131000	2.53801200	2.13468500
H	3.47605700	2.10724200	0.16797200

O	3.29923200	-1.42281000	-1.74839800
C	2.47930900	-2.05952900	-0.79358500
H	2.37625200	-3.09943700	-1.10499900
H	2.91832000	-2.02278300	0.21231300
H	4.03254700	0.31566600	-2.37909100
H	1.83506500	0.21947800	0.50067400
O	-1.52191900	-0.45613800	-1.54645600
C	-2.88222600	-0.91604200	-1.53240900
C	-3.10405900	-1.89346000	-0.41820500
C	-2.49223000	-3.15471200	-0.45537200
C	-2.61124700	-4.02665400	0.62041900
C	-3.34485600	-3.64831800	1.73985400
C	-3.97314200	-2.40421100	1.77854700
C	-3.85863700	-1.53355900	0.70335800
H	-4.34371300	-0.56192800	0.73060100
H	-4.55627400	-2.11877400	2.64729700
H	-3.44057200	-4.32831300	2.57933400
H	-2.14206900	-5.00348800	0.58156500
H	-1.92927900	-3.44918000	-1.33668900
H	-3.54661400	-0.05127100	-1.42769300
H	-3.03199400	-1.36936400	-2.51366500
H	-1.90304100	0.57781900	0.22074500
H	0.59928500	-1.47026300	-1.73137500

Glc-oxo/struc2.out

Charge=+1, Multiplicity=+1

C	-0.23137	0.48002	1.42257
C	-1.06152	0.36042	0.14974
C	-0.58652	-0.80581	-0.71210
H	-0.93565	1.26505	-0.46754
O	-2.41110	0.17575	0.45436
O	0.79391	-0.50308	-1.10524
H	-0.57664	-1.74257	-0.15393
C	-1.45415	-0.88787	-1.95062
C	1.59036	-0.17747	-0.17509
C	1.13216	-0.25211	1.24963
H	1.87260	0.20955	1.90576
O	0.91799	-1.60906	1.57181

H	-0.74069	-0.09154	2.20370
O	-0.07786	1.76730	1.94294
H	2.57313	0.15608	-0.50265
O	-2.78322	-1.00849	-1.50849
H	-1.22801	-1.76542	-2.55742
H	-1.31680	0.01535	-2.56550
C	-3.19991	0.08778	-0.72706
C	-4.63009	-0.08617	-0.34261
H	-3.05168	1.01490	-1.30722
C	-5.55929	0.89354	-0.67142
C	-6.89239	0.73754	-0.30981
C	-7.29290	-0.39914	0.38033
C	-6.36214	-1.38070	0.70928
C	-5.03179	-1.22645	0.34944
H	-5.24449	1.78064	-1.21327
H	-7.61656	1.50204	-0.56845
H	-8.33286	-0.52315	0.66231
H	-6.67732	-2.26839	1.24672
H	-4.30277	-1.98903	0.59980
C	2.13374	-2.30241	1.89184
C	3.15642	-2.15660	0.80517
H	1.82779	-3.34066	2.02978
H	2.52259	-1.92073	2.84292
C	4.30245	-1.38155	1.00307
C	5.20266	-1.18005	-0.03474
C	4.96872	-1.75564	-1.28016
C	3.84267	-2.54835	-1.48281
C	2.94434	-2.75289	-0.44446
H	4.48680	-0.93727	1.97693
H	6.09184	-0.58078	0.12790
H	5.67430	-1.60148	-2.08935
H	3.67380	-3.01667	-2.44619
H	2.07004	-3.37966	-0.59603
C	0.24464	2.83736	1.06492
C	1.53205	2.64708	0.30402
H	0.32520	3.70201	1.72686
H	-0.58021	3.04253	0.37308
C	1.55574	2.67886	-1.09263

C	2.74267	2.50234	0.99474
C	3.93685	2.36359	0.30295
C	3.94447	2.38549	-1.09147
C	2.75434	2.55391	-1.78878
H	0.62824	2.81917	-1.64120
H	2.73932	2.51916	2.08087
H	4.86866	2.26126	0.84854
H	4.88076	2.29049	-1.63049
H	2.75767	2.59154	-2.87254

Glc-oxo/struc3.out

Charge=+1, Multiplicity=+1

C	-0.68860	0.80553	-1.18575
C	0.09542	0.18136	-0.02850
C	-0.72285	-0.83857	0.75238
H	0.35484	0.99546	0.66668
O	1.25129	-0.46529	-0.47137
O	-1.92548	-0.13495	1.22646
H	-1.05691	-1.65805	0.11563
C	0.09994	-1.32439	1.92811
C	-2.61190	0.45936	0.35124
C	-2.18430	0.44406	-1.09457
H	-2.75829	1.19283	-1.64914
O	-2.35927	-0.85198	-1.61040
H	-0.32450	0.37814	-2.13024
O	-0.65731	2.20409	-1.19009
H	-3.42970	1.05996	0.74724
O	1.28491	-1.85655	1.38557
H	-0.39407	-2.12371	2.48192
H	0.30926	-0.48748	2.61145
C	2.01888	-0.90579	0.63715
C	3.28619	-1.52819	0.15401
H	2.22637	-0.03234	1.27686
C	3.29941	-2.32437	-0.98756
C	4.48681	-2.90845	-1.40757
C	5.65928	-2.70328	-0.68755
C	5.64505	-1.91156	0.45481
C	4.45865	-1.32267	0.87361

H	2.38323	-2.47829	-1.54626
H	4.49890	-3.52704	-2.29839
H	6.58536	-3.16112	-1.01804
H	6.55789	-1.75160	1.01813
H	4.44358	-0.69973	1.76304
C	-3.73291	-1.13483	-1.90971
C	-4.61448	-0.86458	-0.72689
H	-3.74281	-2.18789	-2.19517
H	-4.04539	-0.53268	-2.77057
C	-4.52044	-1.66742	0.41931
C	-5.28676	-1.38450	1.54193
C	-6.16474	-0.30493	1.52749
C	-6.28597	0.48542	0.38616
C	-5.51960	0.20404	-0.73631
H	-3.84499	-2.51848	0.42033
H	-5.21146	-2.01267	2.42253
H	-6.76826	-0.08702	2.40188
H	-6.98501	1.31430	0.37116
H	-5.62160	0.81206	-1.63055
C	0.57126	2.79216	-1.63784
C	1.66111	2.79491	-0.60076
H	0.29422	3.81333	-1.90821
H	0.90830	2.27730	-2.54583
C	2.87882	2.16787	-0.84653
C	1.45802	3.43298	0.62513
C	2.45262	3.42715	1.59335
C	3.66857	2.79608	1.33922
C	3.88290	2.17272	0.11664
H	3.04335	1.66131	-1.79238
H	0.51543	3.93790	0.81723
H	2.28917	3.92933	2.54103
H	4.45043	2.80272	2.09136
H	4.82695	1.68014	-0.08878

Glc-oxo/struc4.out

Charge=+1, Multiplicity=+1

C	-0.09732	-0.76488	-1.59065
C	-1.01952	-0.78602	-0.38368

C	-0.87936	0.49566	0.43180
H	-0.72172	-1.59837	0.29006
O	-2.35644	-0.91567	-0.75365
O	0.48879	0.45028	0.99034
H	-0.92871	1.39269	-0.18609
C	-1.91698	0.46940	1.54569
C	1.41751	0.22483	0.16042
C	1.10923	0.18168	-1.30808
H	1.97992	-0.17671	-1.86023
O	0.72918	1.47894	-1.71511
H	-0.62083	-0.28679	-2.42358
O	0.31415	-2.01071	-2.07004
H	2.40167	0.06221	0.59630
O	-2.52976	-0.80145	1.57599
H	-2.66205	1.25453	1.37738
H	-1.45012	0.62499	2.51929
C	-3.22078	-1.08458	0.37808
C	-4.44771	-0.22705	0.20114
H	-3.48108	-2.14475	0.46139
C	-4.68629	0.49579	-0.96335
C	-5.83786	1.26842	-1.07642
C	-6.75006	1.32082	-0.03081
C	-6.51427	0.59454	1.13293
C	-5.36907	-0.17982	1.24788
H	-3.97749	0.44453	-1.78081
H	-6.02319	1.82712	-1.98746
H	-7.64803	1.92216	-0.12234
H	-7.22695	0.62911	1.94968
H	-5.18190	-0.74567	2.15521
C	1.85375	2.34038	-1.94949
C	2.76677	2.39520	-0.76159
H	1.41122	3.31288	-2.17093
H	2.39336	1.98906	-2.83652
C	2.33746	2.99765	0.42764
C	3.14156	2.97249	1.55883
C	4.38829	2.35531	1.51057
C	4.83519	1.77612	0.32673
C	4.02944	1.79818	-0.80405

H	1.36801	3.48685	0.45929
H	2.80382	3.44429	2.47496
H	5.01936	2.34129	2.39258
H	5.81544	1.31404	0.28467
H	4.37933	1.35041	-1.72971
C	0.71350	-3.00899	-1.13800
C	1.86419	-2.60319	-0.25153
H	1.00535	-3.85126	-1.76843
H	-0.13609	-3.34258	-0.53160
C	3.10542	-2.27850	-0.81445
C	1.73762	-2.60391	1.14019
C	2.81821	-2.27663	1.95427
C	4.03698	-1.93379	1.38184
C	4.17957	-1.93827	-0.00540
H	3.22478	-2.31496	-1.89358
H	0.78863	-2.87818	1.59334
H	2.70699	-2.29324	3.03285
H	4.88180	-1.68136	2.01340
H	5.13822	-1.69849	-0.45258

Glc-oxo/struc5.out

Charge=+1, Multiplicity=+1

C	-1.01996	-0.75539	0.59325
C	0.25913	-1.57254	0.63564
C	0.20458	-2.76388	-0.30493
H	0.39072	-1.94854	1.66748
O	1.34101	-0.78258	0.25399
O	-0.87687	-3.67604	0.12313
H	-0.05128	-2.46654	-1.32479
C	1.52513	-3.51032	-0.25629
C	-1.94968	-3.15779	0.52559
C	-2.22418	-1.70105	0.49043
H	-2.98012	-1.40763	1.22198
O	-2.76160	-1.84746	-0.82531
H	-1.00234	-0.13772	-0.31271
O	-1.12476	0.00628	1.75652
H	-2.72844	-3.87409	0.78634
O	2.53261	-2.58579	-0.57805

H	1.56794	-4.31594	-0.99007
H	1.67448	-3.93313	0.74929
C	2.56772	-1.49392	0.31388
C	3.66730	-0.55959	-0.06501
H	2.69880	-1.87378	1.34087
C	4.56728	-0.12520	0.90046
C	5.56477	0.78250	0.56366
C	5.66041	1.25251	-0.74022
C	4.76036	0.81425	-1.70748
C	3.76255	-0.08852	-1.37155
H	4.49083	-0.49475	1.91888
H	6.26983	1.11664	1.31698
H	6.44212	1.95604	-1.00640
H	4.83965	1.17763	-2.72643
H	3.05737	-0.43204	-2.11985
C	-4.14855	-1.42863	-0.98651
C	-4.29899	0.03113	-0.71520
H	-4.78481	-2.02900	-0.32825
H	-4.36792	-1.68862	-2.02242
C	-4.93183	0.47807	0.44455
C	-5.05570	1.83974	0.69776
C	-4.54558	2.76096	-0.20855
C	-3.91427	2.32229	-1.37034
C	-3.78983	0.96433	-1.62271
H	-5.35268	-0.23988	1.14352
H	-5.55704	2.17984	1.59700
H	-4.64677	3.82348	-0.01634
H	-3.53161	3.04374	-2.08416
H	-3.30791	0.62221	-2.53397
C	-1.28554	1.42566	1.54968
C	-0.08597	2.04143	0.89838
H	-1.44344	1.81738	2.55551
H	-2.19109	1.60878	0.96083
C	1.10851	2.16844	1.60793
C	-0.12718	2.43574	-0.43692
C	1.00980	2.93915	-1.06066
C	2.19646	3.05055	-0.34879
C	2.24318	2.66921	0.98899

H	1.14691	1.86294	2.64950
H	-1.06093	2.35868	-0.98754
H	0.96791	3.24631	-2.10037
H	3.08733	3.43650	-0.83147
H	3.16992	2.76136	1.54424

Glc-oxo/struc6.out

Charge=+1, Multiplicity=+1

C	1.68552	-0.94713	-0.59706
C	0.31226	-1.42566	-1.06969
C	-0.11475	-2.71395	-0.37392
H	0.38512	-1.63912	-2.15112
O	-0.68010	-0.47558	-0.81816
O	0.95757	-3.70519	-0.61143
H	-0.17242	-2.57096	0.70510
C	-1.43494	-3.17868	-0.94447
C	2.12530	-3.34793	-0.34304
C	2.38908	-2.01129	0.27746
H	3.46284	-1.79525	0.27356
O	1.88669	-2.19907	1.57301
H	1.55126	-0.04252	0.00325
O	2.57117	-0.73619	-1.66370
H	2.89263	-4.08665	-0.57855
O	-2.32732	-2.12256	-0.67455
H	-1.81964	-4.06803	-0.44449
H	-1.35335	-3.37413	-2.02377
C	-1.95205	-0.91340	-1.28108
C	-2.94875	0.15625	-0.94949
H	-1.87022	-1.08077	-2.37044
C	-2.64463	1.47723	-1.26919
C	-3.53571	2.49266	-0.95373
C	-4.73626	2.19200	-0.31765
C	-5.04269	0.87293	-0.00690
C	-4.15190	-0.14711	-0.32306
H	-1.70132	1.71044	-1.75042
H	-3.29614	3.52026	-1.20530
H	-5.43456	2.98482	-0.07217
H	-5.98068	0.63396	0.48245

H	-4.38649	-1.17574	-0.07869
C	1.92500	-1.04288	2.45312
C	0.64628	-0.26614	2.39929
H	2.78935	-0.41934	2.20102
H	2.09009	-1.48254	3.43869
C	0.62968	1.06864	2.00095
C	-0.57500	1.75332	1.90124
C	-1.77102	1.10801	2.18785
C	-1.76038	-0.21862	2.60545
C	-0.55697	-0.89929	2.72100
H	1.55925	1.57727	1.75675
H	-0.57860	2.78939	1.58185
H	-2.71301	1.63480	2.08052
H	-2.69237	-0.72004	2.84188
H	-0.54737	-1.92887	3.06989
C	2.38426	0.52099	-2.32603
C	2.51788	1.68502	-1.38498
H	1.40584	0.54528	-2.82236
H	3.15573	0.53699	-3.09812
C	3.66519	1.82308	-0.60008
C	1.50269	2.63072	-1.27490
C	1.63238	3.70928	-0.40618
C	2.77250	3.83654	0.37652
C	3.78954	2.88900	0.28000
H	4.46587	1.09450	-0.68951
H	0.60545	2.52527	-1.87703
H	0.84143	4.44873	-0.33985
H	2.87539	4.67543	1.05627
H	4.68681	2.99411	0.88057

Glc-oxo/struc7.out

Charge=+1, Multiplicity=+1

C	1.72027	-0.83328	-0.69547
C	0.37764	-1.39945	-1.12990
C	0.02824	-2.68901	-0.39634
H	0.44860	-1.62919	-2.20747
O	-0.65013	-0.49031	-0.87994
O	1.12914	-3.63962	-0.65956

H	0.00123	-2.53269	0.68281
C	-1.28857	-3.22034	-0.91780
C	2.29146	-3.22859	-0.45550
C	2.53323	-1.87065	0.12686
H	3.59926	-1.62293	0.06432
O	2.12608	-2.07916	1.45638
H	1.53333	0.03391	-0.05668
O	2.43375	-0.48234	-1.84948
H	3.07542	-3.94468	-0.70681
O	-2.21959	-2.19857	-0.65080
H	-1.61976	-4.11271	-0.38588
H	-1.22528	-3.44046	-1.99376
C	-1.91079	-0.98986	-1.29869
C	-2.94431	0.04316	-0.96213
H	-1.85156	-1.18334	-2.38505
C	-2.64862	1.38852	-1.16916
C	-3.58494	2.36176	-0.85025
C	-4.81989	1.99603	-0.32415
C	-5.11602	0.65312	-0.12505
C	-4.18115	-0.32524	-0.44471
H	-1.67677	1.67105	-1.55761
H	-3.35240	3.40909	-1.01158
H	-5.55185	2.75689	-0.07554
H	-6.07925	0.36331	0.28061
H	-4.40642	-1.37225	-0.28283
C	2.06892	-0.91108	2.32094
C	0.70088	-0.30186	2.34338
H	2.82907	-0.18563	2.01174
H	2.34969	-1.31047	3.29758
C	-0.39481	-1.07850	2.72945
C	-1.67854	-0.55223	2.68232
C	-1.87698	0.76174	2.27178
C	-0.78729	1.55064	1.92597
C	0.49596	1.02056	1.95616
H	-0.23851	-2.09763	3.07445
H	-2.52622	-1.16511	2.96842
H	-2.88111	1.16796	2.21616
H	-0.93679	2.57713	1.61057

H	1.33986	1.64053	1.66556
C	3.39023	0.56803	-1.67528
C	2.80898	1.80445	-1.04848
H	3.75277	0.76582	-2.68631
H	4.24623	0.21805	-1.08234
C	1.60739	2.33811	-1.51737
C	3.46931	2.43587	0.00334
C	2.94593	3.59180	0.57439
C	1.74527	4.11252	0.10737
C	1.07627	3.48156	-0.93771
H	1.08885	1.85022	-2.33677
H	4.40809	2.03028	0.37157
H	3.47389	4.08206	1.38511
H	1.33396	5.01198	0.55259
H	0.14333	3.89206	-1.30947

Glc-oxo/struc8.out

Charge=+1, Multiplicity=+1

C	-1.04703	-0.74104	0.98018
C	-0.21894	-1.39116	-0.13635
C	0.73229	-0.40892	-0.81482
H	-0.90279	-1.79118	-0.89668
O	0.56503	-2.42885	0.39465
O	0.00478	0.79731	-1.18212
H	1.49683	-0.10001	-0.10438
C	1.32936	-1.07020	-2.03885
C	-0.58493	1.38254	-0.21949
C	-0.65903	0.76980	1.14969
H	-1.43707	1.29245	1.72089
O	0.55609	0.73600	1.84528
H	-0.75918	-1.20389	1.92615
O	-2.42150	-0.90764	0.86572
H	-1.18419	2.23606	-0.52213
O	1.38462	-2.46712	-1.78933
H	2.32034	-0.66564	-2.26657
H	0.67582	-0.93655	-2.90448
C	1.65966	-2.79560	-0.45217
C	2.95569	-2.18479	0.04739

H	1.70454	-3.88594	-0.42839
C	3.06405	-1.67080	1.33775
C	4.26460	-1.11539	1.76836
C	5.36144	-1.07101	0.91520
C	5.26035	-1.59892	-0.36833
C	4.06462	-2.15961	-0.79871
H	2.20750	-1.70656	2.00028
H	4.34707	-0.72968	2.77938
H	6.29836	-0.64205	1.25402
H	6.11821	-1.58332	-1.03194
H	3.98744	-2.58196	-1.79597
C	1.11994	2.02818	2.08709
C	1.52451	2.65885	0.79174
H	1.97922	1.83615	2.73093
H	0.40192	2.65242	2.63281
C	0.78663	3.71596	0.24189
C	1.10265	4.21833	-1.01938
C	2.15302	3.66378	-1.73404
C	2.91416	2.63024	-1.17968
C	2.61228	2.13894	0.07842
H	-0.01819	4.16340	0.82020
H	0.53538	5.04495	-1.43269
H	2.40242	4.04938	-2.71659
H	3.75592	2.22412	-1.73046
H	3.21261	1.34451	0.51537
C	-3.02742	-0.33015	-0.26454
C	-4.51648	-0.36746	-0.14649
H	-2.70356	-0.81534	-1.19536
H	-2.69927	0.73387	-0.33976
C	-5.13875	-0.22646	1.09270
C	-5.29080	-0.50262	-1.29677
C	-6.67698	-0.48937	-1.21199
C	-7.29514	-0.34886	0.02488
C	-6.52426	-0.22258	1.17601
H	-4.53675	-0.14354	1.99072
H	-4.81062	-0.62893	-2.26319
H	-7.27418	-0.60053	-2.11024
H	-8.37744	-0.34588	0.09331

H	-7.00544	-0.12407	2.14294

Glc-6B/conf_1.out

Charge=+1, Multiplicity=+1

C	1.51836	-3.13260	-0.30608
O	0.85099	-3.08301	0.93988
C	1.78296	-2.38770	1.74986
C	1.70993	-0.89506	1.44063
O	0.46949	-0.44636	1.94070
C	1.34907	-1.77316	-0.97213
O	3.15037	-0.30543	-0.38321
O	-0.04950	-1.66409	-1.38024
H	1.07654	-3.92428	-0.91211
C	2.97314	-3.39612	0.09250
O	3.04316	-2.89369	1.42089
H	1.54844	-2.58507	2.79611
H	3.69499	-2.87610	-0.54123
H	3.18792	-4.46693	0.10241
C	0.40816	0.95957	2.22672
C	-0.95105	1.46966	1.85755
H	1.17295	1.49136	1.65068
H	0.61504	1.10211	3.29263
C	-2.01777	1.36714	2.74994
C	-3.28471	1.80975	2.38483
C	-3.49487	2.35559	1.12205
C	-2.43529	2.46275	0.22756
C	-1.16944	2.02321	0.59535
H	-1.85174	0.95135	3.73932
H	-4.10477	1.74253	3.09200
H	-4.48021	2.71307	0.84228
H	-2.59109	2.90467	-0.75099
H	-0.33755	2.14224	-0.09399
C	3.31645	0.56206	-1.50539
C	2.61599	1.88138	-1.33140
H	4.39719	0.69406	-1.58597
H	2.97277	0.07742	-2.43010
C	1.66908	2.31435	-2.25669
C	1.02945	3.54038	-2.09480

C	1.32276	4.33481	-0.99398
C	2.26250	3.90567	-0.05980
C	2.90883	2.68900	-0.23012
H	1.44046	1.69574	-3.12061
H	0.30500	3.87422	-2.83009
H	0.82593	5.29005	-0.86406
H	2.50070	4.52969	0.79495
H	3.65349	2.36087	0.48935
C	-0.95298	-1.35834	-0.52827
C	1.81582	-0.60701	-0.07410
H	2.54967	-0.39305	1.93196
H	1.88156	-1.73343	-1.92210
H	1.17541	0.26184	-0.28394
C	-2.31659	-1.37725	-0.89099
C	-2.75939	-1.77578	-2.16966
C	-4.10857	-1.77215	-2.44374
C	-5.01917	-1.37663	-1.45622
C	-4.58916	-0.97831	-0.19378
C	-3.23758	-0.97505	0.09519
H	-2.87740	-0.65536	1.06796
H	-6.08059	-1.38156	-1.68199
H	-5.30687	-0.66919	0.55668
H	-2.03807	-2.08311	-2.91810
H	-4.46904	-2.07805	-3.41892
H	-0.65238	-1.06546	0.48350

Glc-6B/conf_2.out

Charge=+1, Multiplicity=+1

C	0.48016	-3.11904	1.07848
O	0.04635	-2.04931	1.89801
C	1.21957	-1.26375	2.01104
C	1.40749	-0.44735	0.72937
O	0.35965	0.48790	0.59744
C	0.48557	-2.61299	-0.35832
O	2.62343	-1.82655	-0.88620
O	-0.91298	-2.43527	-0.75077
H	-0.20625	-3.95760	1.19894
C	1.89481	-3.39707	1.59648

O	2.27852	-2.15921	2.18646
H	1.11839	-0.62810	2.89233
H	2.59651	-3.66569	0.80430
H	1.88270	-4.17060	2.36727
C	0.51588	1.74684	1.25356
C	-0.76858	2.48525	1.03680
H	1.37076	2.27191	0.81066
H	0.70488	1.60374	2.32355
C	-1.74597	2.52163	2.02903
C	-2.96982	3.13910	1.79046
C	-3.22572	3.71765	0.55215
C	-2.25475	3.68294	-0.44516
C	-1.03184	3.07073	-0.20192
H	-1.54725	2.06891	2.99608
H	-3.71889	3.17522	2.57425
H	-4.17435	4.21135	0.36867
H	-2.44817	4.14439	-1.40767
H	-0.26986	3.04616	-0.97542
C	3.28762	-1.05735	-1.89641
C	3.55725	0.36229	-1.48664
H	4.21657	-1.59960	-2.07878
H	2.68800	-1.07969	-2.81597
C	4.54420	0.64458	-0.53971
C	4.75306	1.94710	-0.10874
C	3.98232	2.98562	-0.62639
C	3.00831	2.71619	-1.57974
C	2.79709	1.40803	-2.00540
H	5.14466	-0.16485	-0.13486
H	5.52480	2.15722	0.62388
H	4.15373	4.00487	-0.29708
H	2.42299	3.52630	-2.00236
H	2.04285	1.20110	-2.76009
C	-1.56204	-1.38784	-0.41227
C	1.35303	-1.34812	-0.52780
H	2.38064	0.04920	0.74892
H	0.84478	-3.36888	-1.05521
H	0.92463	-0.73620	-1.33455
C	-2.94006	-1.25668	-0.68828

C	-3.52962	-0.02706	-0.32975
C	-4.87384	0.17926	-0.57657
C	-5.62447	-0.83129	-1.17184
C	-5.04491	-2.05466	-1.52944
C	-3.70629	-2.27473	-1.29225
H	-3.23755	-3.21460	-1.56076
H	-6.68034	-0.66988	-1.36330
H	-5.65193	-2.82483	-1.99074
H	-2.92681	0.74879	0.13381
H	-5.33643	1.12115	-0.30670
H	-1.02945	-0.56590	0.07679

Glc-6B/conf_3.out

Charge=+1, Multiplicity=+1

C	-1.75936	-2.79887	-0.73358
O	-1.69492	-2.64552	0.67254
C	-0.31389	-2.83198	0.92922
C	0.44482	-1.55072	0.57118
O	0.10100	-0.53205	1.49102
C	-1.29111	-1.48639	-1.34931
O	0.96823	-1.60728	-1.81657
O	-2.33455	-0.50189	-1.07519
H	-2.78751	-3.02444	-1.01807
C	-0.77822	-3.94397	-1.00118
O	0.09720	-3.89481	0.12096
H	-0.19939	-3.09481	1.98236
H	-0.20937	-3.81328	-1.92361
H	-1.29444	-4.90628	-1.00791
C	0.92643	-0.47544	2.66393
C	2.25029	0.17955	2.40198
H	1.06592	-1.48426	3.06932
H	0.34719	0.09859	3.38972
C	3.39574	-0.58188	2.17077
C	4.61346	0.03544	1.90367
C	4.69782	1.42257	1.88242
C	3.56306	2.19075	2.12661
C	2.34656	1.57143	2.37809
H	3.34371	-1.66628	2.22372

H	5.49858	-0.56636	1.72855
H	5.64906	1.90542	1.68680
H	3.63064	3.27348	2.12819
H	1.46279	2.17337	2.56893
C	2.25209	-0.97605	-1.89627
C	2.14715	0.48955	-2.19844
H	2.81566	-1.13133	-0.96825
H	2.76189	-1.51835	-2.69446
C	1.57426	0.92272	-3.39603
C	1.44522	2.27887	-3.66033
C	1.89024	3.21619	-2.73063
C	2.46290	2.79153	-1.53883
C	2.58717	1.43170	-1.27322
H	1.23704	0.19100	-4.12463
H	1.01052	2.60922	-4.59779
H	1.79729	4.27594	-2.94299
H	2.81671	3.51635	-0.81324
H	3.03538	1.10064	-0.34199
C	-2.41196	0.07967	0.05631
C	0.10141	-1.06573	-0.85380
H	1.51959	-1.75103	0.59917
H	-1.26605	-1.52848	-2.43698
H	0.16312	0.03293	-0.85071
C	-3.50911	0.92077	0.35404
C	-4.56494	1.13763	-0.55421
C	-5.60231	1.96970	-0.19379
C	-5.59855	2.58398	1.06285
C	-4.55992	2.37399	1.96740
C	-3.51293	1.54316	1.61716
H	-2.69341	1.36169	2.30523
H	-6.42118	3.23593	1.33778
H	-4.57568	2.85735	2.93696
H	-4.54915	0.65160	-1.52288
H	-6.42194	2.14859	-0.87981
H	-1.61049	-0.06383	0.78843

Glc-6B/conf_4.out

Charge=+1, Multiplicity=+1

C	-0.74524	-2.96050	-0.52782
O	-0.88437	-2.69347	0.85568
C	0.43230	-2.31260	1.22144
C	0.66400	-0.86120	0.79649
O	-0.20000	-0.08917	1.61118
C	-0.72239	-1.62007	-1.25377
O	1.47727	-0.99079	-1.52430
O	-2.07446	-1.06987	-1.18632
H	-1.58655	-3.57184	-0.85629
C	0.60249	-3.67949	-0.59774
O	1.29249	-3.18445	0.54571
H	0.53635	-2.42831	2.30070
H	1.16917	-3.44868	-1.50133
H	0.47088	-4.75914	-0.49741
C	0.31151	1.17174	2.05206
C	0.32309	2.21270	0.96904
H	1.31765	1.03154	2.46266
H	-0.34953	1.46623	2.87092
C	1.52117	2.75142	0.51262
C	1.52579	3.68738	-0.51832
C	0.33198	4.08423	-1.10563
C	-0.87388	3.55860	-0.64649
C	-0.87594	2.63610	0.38961
H	2.45851	2.43383	0.95887
H	2.46501	4.10929	-0.86024
H	0.33575	4.81330	-1.90855
H	-1.81039	3.88545	-1.08604
H	-1.82092	2.25235	0.76503
C	2.42998	0.03903	-1.75878
C	3.58823	0.05179	-0.79458
H	2.80135	-0.14830	-2.77127
H	1.92914	1.01575	-1.76528
C	3.94444	-1.07917	-0.06418
C	5.04235	-1.04339	0.78954
C	5.80192	0.11410	0.90892
C	5.46194	1.23958	0.16562
C	4.35968	1.20740	-0.67894
H	3.35542	-1.98559	-0.15411

H	5.30857	-1.92881	1.35720
H	6.65987	0.13821	1.57187
H	6.05580	2.14400	0.24509
H	4.09969	2.08816	-1.25980
C	-2.53098	-0.55704	-0.11182
C	0.37212	-0.67336	-0.71693
H	1.70806	-0.59190	0.97769
H	-0.56108	-1.74157	-2.32350
H	0.05542	0.36610	-0.88048
C	-3.88328	-0.15019	-0.03154
C	-4.31281	0.39279	1.19504
C	-5.62304	0.80736	1.33854
C	-6.50097	0.68398	0.26354
C	-6.08246	0.14558	-0.95765
C	-4.77924	-0.27497	-1.11215
H	-4.43534	-0.69572	-2.05004
H	-7.52982	1.00995	0.37558
H	-6.78478	0.05878	-1.77842
H	-3.61230	0.47080	2.02081
H	-5.96698	1.22235	2.27847
H	-1.85296	-0.41509	0.73569

Glc-6B/conf_5.out

Charge=+1, Multiplicity=+1

C	1.15913	-3.62191	-0.29838
O	2.00410	-3.42695	0.82578
C	2.95029	-2.51066	0.31704
C	2.36437	-1.08233	0.35462
O	2.74507	-0.38222	1.50726
C	0.40837	-2.31353	-0.57901
O	0.13015	-0.08158	-0.00917
O	-1.02063	-2.46198	-0.36597
H	0.48075	-4.45058	-0.09723
C	2.20498	-3.86822	-1.37168
O	3.17326	-2.88235	-1.03487
H	3.86397	-2.59130	0.90824
H	1.86154	-3.69675	-2.39384
H	2.62267	-4.87542	-1.28340

C	3.89297	0.45767	1.32469
C	3.55175	1.68785	0.53363
H	4.70141	-0.10641	0.84301
H	4.21410	0.71397	2.33533
C	3.82779	1.76934	-0.83014
C	3.42218	2.87587	-1.57007
C	2.73998	3.91428	-0.94851
C	2.47845	3.85302	0.41750
C	2.88501	2.74763	1.15223
H	4.38032	0.96954	-1.31637
H	3.65017	2.93103	-2.62926
H	2.43003	4.78153	-1.52184
H	1.96742	4.67335	0.91020
H	2.69303	2.70644	2.22110
C	-0.17298	0.87549	1.01485
C	-1.25482	1.76714	0.49231
H	-0.49902	0.35155	1.92108
H	0.73739	1.43488	1.24570
C	-1.10011	2.39550	-0.74508
C	-2.11185	3.20116	-1.25128
C	-3.27836	3.40276	-0.51729
C	-3.42846	2.79414	0.72291
C	-2.42274	1.97254	1.22104
H	-0.17893	2.25730	-1.30280
H	-1.98395	3.69035	-2.21130
H	-4.06035	4.04624	-0.90648
H	-4.32994	2.95708	1.30410
H	-2.54776	1.48923	2.18585
C	-1.74815	-1.51153	-0.83028
C	0.84551	-1.21724	0.38295
H	2.66097	-0.55148	-0.55687
H	0.54555	-1.97866	-1.61234
H	0.56651	-1.52022	1.40074
C	-3.07658	-1.32195	-0.39487
C	-3.81590	-0.28988	-1.00441
C	-5.11941	-0.06137	-0.60964
C	-5.68203	-0.85196	0.38930
C	-4.94946	-1.87053	1.00886

C	-3.64813	-2.10969	0.62575
H	-3.06306	-2.89404	1.09214
H	-6.70645	-0.67204	0.69871
H	-5.40770	-2.46895	1.78760
H	-3.34949	0.33610	-1.75868
H	-5.69503	0.73407	-1.06719
H	-1.34054	-0.88772	-1.62266

Glc-4B/conf_1.out

Charge=+1, Multiplicity=+1

C	-0.18169	-3.12627	0.88475
O	-1.54884	-3.11116	1.28180
C	-0.28950	-2.98451	-0.64730
C	-0.78423	-1.60268	-1.08466
C	-2.18524	-1.57503	-0.43971
C	-2.27007	-3.03179	0.05041
H	0.25819	-4.10112	1.12233
C	0.50585	-2.03671	1.68093
O	-1.47167	-3.74815	-0.85853
H	0.54333	-3.39493	-1.21375
H	-3.25482	-3.47900	0.15951
O	-3.15014	-1.22524	-1.38997
H	-2.20076	-0.88728	0.41417
H	-0.91714	-1.62573	-2.17344
O	0.07542	-0.55058	-0.71540
O	1.86723	-1.88429	1.18914
H	0.59498	-2.32700	2.72868
H	-0.01283	-1.08411	1.58507
C	2.29641	-0.71730	0.92463
C	3.59536	-0.50590	0.41822
H	1.64298	0.13538	1.11470
C	3.99715	0.83140	0.23541
C	4.46911	-1.57029	0.11291
C	5.72721	-1.28705	-0.36986
C	6.12134	0.04431	-0.54787
C	5.26397	1.09992	-0.24680
H	3.31012	1.63844	0.47000
H	7.11556	0.25616	-0.92762

H	5.58749	2.12408	-0.39019
H	4.14444	-2.59393	0.26060
H	6.41332	-2.09040	-0.61115
C	-4.33469	-0.66612	-0.81946
C	-4.06834	0.67483	-0.19126
H	-4.76917	-1.35506	-0.08342
H	-5.03324	-0.57999	-1.65328
C	-4.05693	0.83453	1.19293
C	-3.74474	2.06603	1.76287
C	-3.43640	3.14849	0.94888
C	-3.45109	3.00100	-0.43566
C	-3.76770	1.77300	-1.00022
H	-4.30793	-0.00840	1.83154
H	-3.75311	2.18013	2.84187
H	-3.19888	4.11100	1.38939
H	-3.22681	3.84900	-1.07401
H	-3.78859	1.66029	-2.08064
C	-0.27447	0.67034	-1.37642
C	0.43405	1.80970	-0.71534
H	-1.35804	0.81712	-1.31049
H	-0.00235	0.59762	-2.43626
C	1.38654	2.56017	-1.39875
C	2.02339	3.63016	-0.77522
C	1.72690	3.94215	0.54641
C	0.77387	3.19610	1.23779
C	0.12184	2.14566	0.60500
H	1.62446	2.31349	-2.42915
H	2.74893	4.22139	-1.32409
H	2.22057	4.77641	1.03326
H	0.51961	3.45481	2.26030
H	-0.66374	1.60223	1.12410

Glc-4B/conf_2.out

Charge=+1, Multiplicity=+1

C	1.99096	0.34429	2.02324
O	0.69333	0.25060	2.61012
C	2.12249	-1.02026	1.33583
C	1.09808	-1.28980	0.20706

C	-0.22342	-1.27375	0.99511
C	0.32370	-1.13498	2.42062
H	2.75039	0.37418	2.81558
C	2.12135	1.66348	1.28531
O	1.54507	-1.81307	2.37853
H	3.14545	-1.32847	1.13947
H	-0.29559	-1.47204	3.24808
O	-0.90203	-2.48150	0.79348
H	-0.84422	-0.41940	0.70702
H	1.26103	-2.32498	-0.11912
O	1.05218	-0.42873	-0.89367
O	0.94229	1.93403	0.50176
H	2.95325	1.65355	0.58231
H	2.24155	2.48234	2.00004
C	-0.12313	2.24692	1.12959
C	-1.36623	2.27401	0.46918
H	-0.04552	2.53330	2.17842
C	-2.48154	2.76980	1.17477
C	-1.49913	1.80430	-0.85572
C	-2.73320	1.85690	-1.46183
C	-3.83264	2.35982	-0.76049
C	-3.71203	2.81431	0.55288
H	-2.36388	3.11706	2.19659
H	-4.80108	2.39135	-1.24757
H	-4.57874	3.19604	1.07923
H	-0.63512	1.38150	-1.35608
H	-2.86135	1.48863	-2.47145
C	-2.28261	-2.41647	1.09069
C	-3.10488	-1.77262	0.00151
H	-2.45856	-1.90810	2.04930
H	-2.59884	-3.45591	1.21751
C	-2.71344	-1.86676	-1.33356
C	-3.54455	-1.39825	-2.34432
C	-4.77518	-0.83004	-2.03222
C	-5.15989	-0.71110	-0.70081
C	-4.32417	-1.17311	0.30989
H	-1.76469	-2.33441	-1.57424
H	-3.24070	-1.49932	-3.38139

H	-5.43578	-0.49044	-2.82343
H	-6.12071	-0.27403	-0.44833
H	-4.63782	-1.09262	1.34735
C	1.96130	-0.74693	-1.94707
C	3.37041	-0.31368	-1.65747
H	1.57320	-0.22179	-2.82278
H	1.92124	-1.82455	-2.14967
C	3.72169	1.03311	-1.76827
C	5.00552	1.45805	-1.44913
C	5.95581	0.53639	-1.01796
C	5.62209	-0.80966	-0.92496
C	4.33613	-1.23082	-1.24765
H	2.98524	1.74964	-2.12305
H	5.27286	2.50454	-1.55325
H	6.96068	0.86501	-0.77602
H	6.36637	-1.53398	-0.61195
H	4.08368	-2.28646	-1.18953

Glc-4B/conf_3.out

Charge=+1, Multiplicity=+1

C	0.15901	3.74816	0.25200
O	-0.84574	3.12748	1.05093
C	1.42978	3.03209	0.73773
C	1.46154	1.52696	0.40446
C	0.27643	1.01568	1.26715
C	-0.10049	2.31186	1.98112
H	0.25389	4.80373	0.53814
C	-0.29199	3.75220	-1.20242
O	1.11440	2.98978	2.12835
H	2.35270	3.58410	0.56943
H	-0.63660	2.22551	2.92199
O	0.64400	0.08102	2.23925
H	-0.54031	0.63877	0.64307
H	2.37519	1.11035	0.84046
O	1.36337	1.22654	-0.96190
O	-0.89308	2.49785	-1.56624
H	0.53876	3.89324	-1.89086
H	-1.03684	4.53772	-1.35774

C	-2.04293	2.22015	-1.08597
C	-2.56065	0.91659	-1.19454
H	-2.62878	3.01907	-0.63144
C	-3.89785	0.69413	-0.80699
C	-1.75830	-0.14601	-1.66721
C	-2.30533	-1.40416	-1.76452
C	-3.63774	-1.61345	-1.39601
C	-4.43350	-0.57156	-0.91872
H	-4.49672	1.51877	-0.43314
H	-4.05896	-2.60913	-1.47851
H	-5.46158	-0.75893	-0.63264
H	-0.71974	0.04643	-1.91454
H	-1.70361	-2.23746	-2.10453
C	0.88491	-1.21827	1.73506
C	-0.36969	-1.99174	1.41388
H	1.43107	-1.73527	2.52924
H	1.54556	-1.18600	0.85980
C	-1.59519	-1.66119	1.98839
C	-2.71193	-2.46086	1.77298
C	-2.61341	-3.60558	0.98938
C	-1.39469	-3.93506	0.40344
C	-0.28406	-3.12586	0.60551
H	-1.66003	-0.79244	2.63520
H	-3.65679	-2.20314	2.24059
H	-3.47671	-4.24848	0.85087
H	-1.30757	-4.83147	-0.20246
H	0.66928	-3.38687	0.15517
C	2.61368	0.91816	-1.58897
C	3.10028	-0.45536	-1.23081
H	3.36386	1.67536	-1.32891
H	2.41642	0.99281	-2.66066
C	4.11441	-0.63913	-0.29254
C	4.50898	-1.91950	0.08176
C	3.89867	-3.02823	-0.49108
C	2.89844	-2.85468	-1.44416
C	2.50010	-1.57594	-1.80760
H	4.60846	0.22473	0.14377
H	5.29851	-2.05010	0.81370

H	4.21220	-4.02732	-0.20817
H	2.44076	-3.71961	-1.91335
H	1.72323	-1.44210	-2.55545

Glc-4B/conf_4.out

Charge=+1, Multiplicity=+1

C	-0.38136	-2.55197	1.23332
O	0.83123	-2.22743	1.91068
C	-1.21825	-1.29018	1.47207
C	-0.63394	-0.00236	0.84695
C	0.68122	0.14993	1.65620
C	0.51431	-1.01098	2.63249
H	-0.88743	-3.37392	1.75714
C	-0.06254	-3.09587	-0.14771
O	-0.86365	-1.08936	2.84433
H	-2.29137	-1.42727	1.36452
H	1.07262	-0.96748	3.56340
O	0.80258	1.33528	2.38546
H	1.55414	0.00842	1.00810
H	-1.29704	0.81791	1.13829
O	-0.41742	-0.00047	-0.53605
O	0.94393	-2.29810	-0.80540
H	-0.93499	-3.07129	-0.79803
H	0.32345	-4.11586	-0.06990
C	2.13234	-2.34891	-0.35535
C	3.09821	-1.40855	-0.78064
H	2.40130	-3.15108	0.33184
C	4.43241	-1.59526	-0.37162
C	2.73228	-0.29752	-1.56930
C	3.70683	0.59833	-1.95310
C	5.03317	0.39984	-1.55669
C	5.39773	-0.68941	-0.76659
H	4.69501	-2.44829	0.24629
H	5.79281	1.11078	-1.86500
H	6.42967	-0.82232	-0.46433
H	1.68851	-0.15186	-1.82460
H	3.44804	1.45854	-2.55979
C	1.31559	2.43530	1.65272

C	0.32434	3.07822	0.71754
H	2.20979	2.12559	1.09233
H	1.63129	3.15427	2.41323
C	-0.92339	3.48846	1.19050
C	-1.82469	4.11881	0.34259
C	-1.48580	4.35623	-0.98750
C	-0.24511	3.95300	-1.46488
C	0.65089	3.31163	-0.61464
H	-1.18169	3.31411	2.23086
H	-2.78857	4.44147	0.72220
H	-2.18578	4.85921	-1.64587
H	0.02556	4.13967	-2.49890
H	1.62188	2.99708	-0.98788
C	-1.50329	0.53868	-1.29630
C	-2.72873	-0.32520	-1.23273
H	-1.12432	0.60546	-2.31850
H	-1.71965	1.55286	-0.94464
C	-2.78769	-1.51869	-1.95451
C	-3.88033	-2.36766	-1.83075
C	-4.93169	-2.02730	-0.98374
C	-4.89367	-0.82952	-0.27964
C	-3.79774	0.01775	-0.40750
H	-1.97874	-1.76926	-2.63680
H	-3.92469	-3.28592	-2.40727
H	-5.78792	-2.68627	-0.88927
H	-5.72065	-0.55188	0.36503
H	-3.77124	0.95707	0.13821

Glc-4B/conf_5.out

Charge=+1, Multiplicity=+1

C	1.11088	-3.40969	0.70958
O	0.12465	-3.54323	-0.30639
C	2.09167	-2.41093	0.07465
C	1.52212	-1.01125	-0.16993
C	0.36096	-1.31235	-1.13891
C	0.65256	-2.80194	-1.40561
H	1.64966	-4.36005	0.83176
C	0.44999	-3.14462	2.04237

O	2.04750	-2.90659	-1.26234
H	3.10503	-2.44027	0.46964
H	0.31886	-3.23030	-2.34736
O	0.49276	-0.49457	-2.26978
H	-0.62121	-1.18741	-0.66731
H	2.28438	-0.47787	-0.74658
O	1.22548	-0.26812	0.99197
O	-0.58082	-2.13845	1.93070
H	1.17651	-2.84656	2.80383
H	-0.08217	-4.03810	2.36938
C	-0.42533	-1.00495	2.48769
C	-1.44472	-0.02171	2.41133
H	0.42829	-0.85843	3.14799
C	-1.32825	1.10965	3.23850
C	-2.55293	-0.17536	1.55859
C	-3.53126	0.79614	1.54772
C	-3.41104	1.91724	2.37154
C	-2.31467	2.07727	3.21634
H	-0.47274	1.21173	3.89945
H	-4.18615	2.67633	2.35471
H	-2.23894	2.95012	3.85396
H	-2.63063	-1.04923	0.92195
H	-4.38699	0.69163	0.89367
C	-0.64738	-0.50177	-3.11663
C	-1.81305	0.26857	-2.55307
H	-0.95533	-1.52888	-3.35070
H	-0.30934	-0.04310	-4.04914
C	-3.08134	-0.30234	-2.48403
C	-4.17477	0.44911	-2.06188
C	-4.00191	1.77567	-1.68746
C	-2.73202	2.34584	-1.72353
C	-1.64703	1.59747	-2.15783
H	-3.22278	-1.33434	-2.79333
H	-5.16282	0.00059	-2.04303
H	-4.85637	2.37120	-1.38200
H	-2.59417	3.38439	-1.44108
H	-0.66417	2.05454	-2.22340
C	1.11198	1.14837	0.72372

C	2.40554	1.75801	0.27945
H	0.78821	1.58567	1.66920
H	0.32185	1.30595	-0.01766
C	2.61409	2.08269	-1.06001
C	3.82330	2.63284	-1.46957
C	4.83264	2.85786	-0.54183
C	4.63291	2.53453	0.79715
C	3.42431	1.98809	1.20470
H	1.83111	1.88568	-1.78608
H	3.97803	2.88255	-2.51351
H	5.77588	3.28844	-0.85997
H	5.41837	2.71640	1.52263
H	3.26741	1.74220	2.25145

Glc-4B/conf_6.out

Charge=+1, Multiplicity=+1

C	-0.60832	2.03795	1.66735
O	-1.53442	1.12376	2.25147
C	0.61372	1.14436	1.43950
C	0.39621	-0.01556	0.44169
C	-0.68541	-0.84574	1.17405
C	-0.74730	-0.06706	2.49419
H	-0.32330	2.79655	2.40817
C	-1.28206	2.80384	0.54177
O	0.55397	0.42060	2.67596
H	1.55819	1.67549	1.34721
H	-1.11937	-0.58432	3.37524
O	-0.47002	-2.22375	1.30223
H	-1.63770	-0.76710	0.64969
H	1.33280	-0.57827	0.44127
O	0.02644	0.30343	-0.87084
O	-2.06844	1.92647	-0.28870
H	-0.55063	3.26493	-0.11934
H	-1.95321	3.56392	0.95065
C	-3.14775	1.45581	0.19563
C	-3.83006	0.40963	-0.46129
H	-3.55247	1.89990	1.10529
C	-5.09240	0.02633	0.03263

C	-3.25205	-0.25478	-1.56431
C	-3.94916	-1.27946	-2.16616
C	-5.20888	-1.64491	-1.68107
C	-5.78063	-0.99868	-0.58567
H	-5.51660	0.53879	0.89044
H	-5.74930	-2.45369	-2.16202
H	-6.75478	-1.30226	-0.22138
H	-2.25770	0.02985	-1.89129
H	-3.52106	-1.80827	-3.00965
C	0.79266	-2.66776	1.78654
C	1.82903	-2.81165	0.69920
H	0.58784	-3.64408	2.23497
H	1.16874	-2.01227	2.57947
C	1.46226	-3.23968	-0.57592
C	2.41902	-3.36783	-1.57537
C	3.75375	-3.07565	-1.31001
C	4.12695	-2.65910	-0.03732
C	3.16736	-2.52426	0.96086
H	0.41941	-3.45871	-0.78047
H	2.12512	-3.70440	-2.56431
H	4.50002	-3.17883	-2.09029
H	5.16675	-2.43703	0.17937
H	3.46114	-2.18646	1.95114
C	1.13545	0.38395	-1.77557
C	2.08648	1.48429	-1.40748
H	0.68531	0.56244	-2.75461
H	1.65166	-0.58306	-1.79764
C	3.28684	1.19546	-0.76128
C	4.11832	2.22274	-0.32649
C	3.75722	3.54798	-0.53953
C	2.57127	3.84562	-1.20590
C	1.74348	2.81760	-1.63866
H	3.56949	0.15892	-0.59661
H	5.05116	1.98826	0.17509
H	4.40711	4.34913	-0.20458
H	2.30485	4.87904	-1.40222
H	0.83016	3.04689	-2.18261

Glc-4B/conf_7.out

Charge=+1, Multiplicity=+1

C	1.22431	-2.69195	-1.76378
O	1.56454	-3.34862	-0.55018
C	-0.14338	-2.08224	-1.42129
C	-0.12430	-1.01364	-0.32375
C	0.40396	-1.82800	0.89150
C	0.39595	-3.23031	0.25875
H	1.05395	-3.43694	-2.55455
C	2.38675	-1.85605	-2.24942
O	-0.66614	-3.17911	-0.66920
H	-0.78043	-1.86681	-2.27739
H	0.31351	-4.08668	0.92389
O	-0.28945	-1.68004	2.09763
H	1.43218	-1.53924	1.11964
H	-1.16799	-0.74363	-0.14850
O	0.59213	0.16714	-0.64038
O	2.97665	-1.08421	-1.17989
H	2.09442	-1.19070	-3.06697
H	3.19156	-2.51324	-2.57925
C	2.86712	0.18300	-1.17727
C	3.45540	0.95642	-0.14386
H	2.43900	0.67437	-2.05008
C	3.47952	2.35347	-0.30237
C	3.99724	0.35573	1.00732
C	4.55326	1.15437	1.98459
C	4.57704	2.54232	1.82084
C	4.04611	3.14292	0.68162
H	3.06212	2.80476	-1.19759
H	5.01888	3.16200	2.59411
H	4.07737	4.21999	0.56734
H	3.97310	-0.72298	1.11349
H	4.97302	0.70786	2.87840
C	-1.62942	-2.17799	2.18522
C	-2.66052	-1.25121	1.59963
H	-1.79340	-2.29627	3.25863
H	-1.71192	-3.16773	1.72541
C	-3.46544	-1.65602	0.53632

C	-4.39251	-0.78024	-0.01915
C	-4.52165	0.50772	0.48522
C	-3.73340	0.91274	1.55756
C	-2.81015	0.03762	2.11289
H	-3.35311	-2.65837	0.13384
H	-5.01425	-1.10508	-0.84681
H	-5.23826	1.19479	0.04911
H	-3.84421	1.91243	1.96256
H	-2.19788	0.35140	2.95374
C	0.12816	1.28954	0.10898
C	-1.03853	1.99412	-0.52990
H	0.97643	1.97432	0.19695
H	-0.12859	0.96301	1.12371
C	-1.59738	1.57355	-1.73046
C	-2.68258	2.25221	-2.27668
C	-3.20870	3.36240	-1.63168
C	-2.64527	3.79416	-0.43447
C	-1.57015	3.11126	0.11405
H	-1.18524	0.71369	-2.24639
H	-3.11598	1.91061	-3.21063
H	-4.05236	3.89302	-2.05926
H	-3.04873	4.66329	0.07413
H	-1.14369	3.44561	1.05638

Gal-oxo/conf_1.out

Charge=+1, Multiplicity=+1

C	-0.41615	-0.69616	-0.36022
C	0.63239	-0.41926	0.71122
C	0.41893	0.95223	1.32826
O	1.90059	-0.47769	0.11433
H	0.54353	-1.15450	1.52637
O	0.34614	1.98566	0.27136
H	-0.52826	1.03180	1.86569
C	1.60397	1.33864	2.18802
C	0.01210	1.69968	-0.90709
C	-0.21635	0.33376	-1.46298
H	0.74542	0.09926	-1.95384
O	-1.16126	0.38375	-2.48209

O	2.80022	1.15948	1.47821
H	1.57643	0.71628	3.09559
H	1.55031	2.38760	2.48222
C	2.94198	-0.15908	1.02189
C	4.25917	-0.32909	0.32947
C	5.32934	0.50443	0.64105
C	6.55875	0.30921	0.02382
C	6.72273	-0.71589	-0.89994
C	5.65306	-1.54889	-1.20821
C	4.42213	-1.35932	-0.59418
H	5.19725	1.30557	1.35833
H	7.39072	0.96143	0.26582
H	7.68386	-0.86648	-1.37934
H	5.77717	-2.34947	-1.92925
H	3.58528	-2.00441	-0.83728
H	0.05634	2.54009	-1.59572
H	-1.39809	-0.55168	0.10722
O	-0.32613	-1.95597	-0.93838
C	-0.85566	-3.01696	-0.13706
C	-2.28941	-2.78218	0.24733
H	-0.23776	-3.16519	0.75780
H	-0.75039	-3.90349	-0.76357
C	-2.63727	-2.45962	1.55830
C	-3.95842	-2.17862	1.89423
C	-4.94457	-2.21785	0.91556
C	-4.60895	-2.54986	-0.39438
C	-3.28951	-2.82976	-0.72597
H	-1.87040	-2.44700	2.32914
H	-4.21910	-1.94252	2.92056
H	-5.97655	-2.00770	1.17494
H	-5.38059	-2.60217	-1.15508
H	-3.02818	-3.09363	-1.74679
C	-2.51032	0.53787	-2.02237
C	-2.64324	1.67380	-1.04915
H	-2.86422	-0.39615	-1.57581
H	-3.08407	0.72184	-2.93219
C	-3.03629	1.44457	0.27557
C	-3.12230	2.49858	1.17374

C	-2.82809	3.79702	0.76018
C	-2.47717	4.04558	-0.56151
C	-2.39180	2.99166	-1.46294
H	-3.29934	0.43735	0.59123
H	-3.43673	2.31462	2.19584
H	-2.90072	4.61866	1.46449
H	-2.28605	5.06018	-0.89315
H	-2.14460	3.18184	-2.50404
H	2.84804	-0.84087	1.88764

Gal-oxo/conf_2.out

Charge=+1, Multiplicity=+1

C	0.89728	0.93234	-0.77015
C	0.01140	0.40088	0.35645
C	0.80376	-0.42630	1.35095
O	-0.98460	-0.40347	-0.21972
H	-0.43177	1.23717	0.91303
O	1.54495	-1.50194	0.65295
H	1.57076	0.14871	1.87421
C	-0.13586	-1.12664	2.31194
C	1.87962	-1.37785	-0.55174
C	1.48367	-0.28145	-1.48381
H	0.64602	-0.73320	-2.04567
O	2.48793	-0.06041	-2.42172
O	-1.15114	-1.79694	1.61395
H	-0.54839	-0.35904	2.98447
H	0.39561	-1.86793	2.91026
C	-1.85590	-0.92823	0.76296
C	-2.97870	-1.65976	0.09566
C	-3.63485	-2.68720	0.76819
C	-4.71351	-3.32634	0.17040
C	-5.14086	-2.94182	-1.09528
C	-4.48488	-1.91604	-1.76570
C	-3.40593	-1.27310	-1.17216
H	-3.29473	-2.98997	1.75163
H	-5.22048	-4.12897	0.69475
H	-5.98347	-3.44262	-1.55950

H	-4.81234	-1.61678	-2.75551
H	-2.88616	-0.47880	-1.69466
H	2.37423	-2.25313	-0.96661
H	1.69397	1.53242	-0.30483
O	0.23212	1.66485	-1.74586
C	-0.08036	3.01548	-1.40928
C	-1.17833	3.13216	-0.38779
H	-0.37629	3.46593	-2.35891
H	0.82527	3.52969	-1.05834
C	-0.97347	3.81008	0.81182
C	-1.97238	3.84786	1.78049
C	-3.18121	3.19994	1.55524
C	-3.39867	2.53437	0.35087
C	-2.40523	2.50831	-0.61779
H	-0.02623	4.31196	0.99193
H	-1.80666	4.38365	2.70905
H	-3.96009	3.22527	2.30975
H	-4.34676	2.04039	0.16541
H	-2.57012	1.98579	-1.55425
C	3.63528	0.61772	-1.90140
C	4.15883	-0.05016	-0.66190
H	3.40037	1.67016	-1.70902
H	4.36248	0.57943	-2.71380
C	4.14832	0.60052	0.57726
C	4.60558	-0.04489	1.71744
C	5.09065	-1.34818	1.63363
C	5.14415	-1.99501	0.40383
C	4.68752	-1.34901	-0.73732
H	3.80192	1.62862	0.63941
H	4.60234	0.47114	2.67178
H	5.45160	-1.84940	2.52510
H	5.55509	-2.99594	0.33238
H	4.74580	-1.84131	-1.70439
H	-2.23148	-0.07610	1.35860

Gal-oxo/conf_3.out

Charge=+1, Multiplicity=+1

C	1.00426	1.19376	-0.33873

C	0.15322	0.47808	0.70430
C	1.02110	-0.43698	1.55113
O	-0.81006	-0.30949	0.04587
H	-0.32369	1.21652	1.36511
O	1.79599	-1.35210	0.67663
H	1.77479	0.09767	2.13296
C	0.15849	-1.32786	2.41991
C	2.04103	-1.07013	-0.52461
C	1.51856	0.10201	-1.28695
H	0.64862	-0.32549	-1.81560
O	2.41506	0.48278	-2.27786
O	-0.83838	-1.95630	1.65757
H	-0.27723	-0.69445	3.20795
H	0.75239	-2.11136	2.89262
C	-1.63127	-1.02937	0.95613
C	-2.69019	-1.74873	0.19082
C	-4.02441	-1.59557	0.54884
C	-5.01654	-2.26068	-0.16168
C	-4.67338	-3.07731	-1.23200
C	-3.33717	-3.23591	-1.58796
C	-2.34636	-2.57530	-0.87612
H	-4.28881	-0.95161	1.38172
H	-6.05684	-2.14186	0.12070
H	-5.44686	-3.59547	-1.78835
H	-3.07032	-3.88004	-2.41872
H	-1.30320	-2.70500	-1.14396
H	2.57006	-1.85360	-1.06235
H	1.85735	1.61455	0.20105
O	0.44925	2.27682	-1.00334
C	-0.85103	2.14280	-1.58129
C	-1.95951	2.39461	-0.59400
H	-0.97940	1.16109	-2.04944
H	-0.86787	2.89519	-2.37388
C	-3.16297	1.70285	-0.70142
C	-4.20446	1.96210	0.18154
C	-4.04545	2.90365	1.19197
C	-2.84347	3.59451	1.30731
C	-1.80815	3.34702	0.41342

H	-3.28560	0.94666	-1.47128
H	-5.13974	1.42166	0.08154
H	-4.85748	3.10520	1.88230
H	-2.71803	4.33973	2.08582
H	-0.87616	3.89822	0.49065
C	3.56228	1.18503	-1.78225
C	4.24604	0.41535	-0.68737
H	3.27154	2.18460	-1.44341
H	4.20738	1.29643	-2.65491
C	4.33929	0.92335	0.61372
C	4.94454	0.18288	1.61937
C	5.47861	-1.07226	1.33610
C	5.43087	-1.57420	0.04013
C	4.82423	-0.83380	-0.96589
H	3.95950	1.91866	0.82890
H	5.01976	0.58973	2.62222
H	5.95616	-1.64698	2.12215
H	5.87956	-2.53450	-0.18880
H	4.80245	-1.21130	-1.98477
H	-2.07202	-0.30726	1.66225

Gal-oxo/conf_4.out

Charge=+1, Multiplicity=+1

C	-0.93878	0.47582	-1.46100
C	-0.32011	-0.91658	-1.47185
C	-1.17964	-1.94496	-0.76340
O	0.91390	-0.82770	-0.80846
H	-0.19163	-1.23674	-2.51678
O	-1.50705	-1.47384	0.59991
H	-2.14474	-2.11214	-1.24618
C	-0.40725	-3.23423	-0.57022
C	-1.53177	-0.24567	0.87317
C	-1.10048	0.87911	0.00644
H	-0.08832	1.11082	0.39841
O	-1.87096	2.01034	0.28885
O	0.84305	-2.98385	0.01592
H	-0.30014	-3.71017	-1.55693
H	-0.94539	-3.91741	0.08818

C	1.59928	-2.06913	-0.73146
C	2.91736	-1.81475	-0.07589
C	3.01772	-1.75059	1.31079
C	4.23642	-1.44493	1.90112
C	5.35372	-1.19799	1.10955
C	5.25310	-1.26110	-0.27569
C	4.03458	-1.57078	-0.86769
H	2.14636	-1.95590	1.92220
H	4.31777	-1.40361	2.98186
H	6.30608	-0.96434	1.57330
H	6.12442	-1.07745	-0.89465
H	3.95557	-1.62574	-1.94965
H	-1.75140	-0.02928	1.91594
H	-1.93751	0.39968	-1.91516
O	-0.13041	1.29108	-2.25269
C	0.16400	2.63528	-1.85822
C	1.12999	2.72188	-0.70631
H	-0.75271	3.18912	-1.63256
H	0.60403	3.06772	-2.75958
C	0.89123	3.59278	0.35725
C	1.78527	3.66617	1.41988
C	2.91815	2.86049	1.43142
C	3.16514	1.99260	0.37127
C	2.28170	1.93197	-0.69613
H	-0.00471	4.20582	0.35779
H	1.59396	4.34922	2.24059
H	3.61136	2.91059	2.26438
H	4.04528	1.35871	0.37734
H	2.46448	1.24582	-1.51460
C	-3.21979	1.95208	-0.19433
C	-3.91664	0.69249	0.23106
H	-3.23983	2.05704	-1.28467
H	-3.69425	2.83481	0.23728
C	-4.33088	-0.26039	-0.70639
C	-4.94830	-1.43310	-0.29394
C	-5.17000	-1.66431	1.06144
C	-4.79953	-0.70921	2.00253
C	-4.18396	0.46453	1.59039

H	-4.18949	-0.06659	-1.76624
H	-5.27548	-2.16158	-1.02831
H	-5.65688	-2.57903	1.38159
H	-5.00527	-0.87315	3.05450
H	-3.91090	1.22327	2.31890
H	1.72799	-2.45278	-1.75776

Gal-oxo/conf_5.out

Charge=+1, Multiplicity=+1

C	0.40238	-1.22383	-1.41452
C	0.14545	0.18831	-1.93770
C	1.25689	1.10838	-1.46426
O	-1.08595	0.64292	-1.42747
H	0.15782	0.18368	-3.03590
O	1.33192	1.07822	0.01651
H	2.24681	0.78116	-1.78480
C	0.95783	2.54461	-1.86483
C	0.98186	0.05616	0.66420
C	0.32347	-1.16046	0.11708
H	-0.73721	-1.01195	0.39012
O	0.72704	-2.28939	0.82620
O	-0.36747	2.66564	-2.33806
H	1.59296	2.83634	-2.70178
H	1.15622	3.21249	-1.01930
C	-1.32418	2.05713	-1.52650
C	-1.41788	2.64783	-0.13660
C	-1.17426	4.00603	0.05427
C	-1.27547	4.56226	1.32407
C	-1.63545	3.76939	2.40770
C	-1.90779	2.41927	2.21520
C	-1.80229	1.86002	0.94703
H	-0.90872	4.62888	-0.79417
H	-1.07917	5.61948	1.46551
H	-1.71900	4.20633	3.39677
H	-2.21822	1.80166	3.05172
H	-2.04881	0.81499	0.79366
H	1.05486	0.17179	1.74278
H	1.43433	-1.45672	-1.69549

O	-0.33520	-2.25511	-1.98289
C	-1.76609	-2.17699	-2.01865
C	-2.43059	-2.27170	-0.67241
H	-2.04334	-3.03205	-2.63891
H	-2.08409	-1.26638	-2.53312
C	-2.14647	-3.33776	0.18371
C	-2.76341	-3.42501	1.42366
C	-3.68345	-2.45703	1.81662
C	-3.98837	-1.40362	0.96229
C	-3.36069	-1.31111	-0.27533
H	-1.42980	-4.09227	-0.12308
H	-2.53509	-4.25548	2.08292
H	-4.17179	-2.53247	2.78229
H	-4.71683	-0.65549	1.25703
H	-3.59246	-0.48694	-0.94275
C	2.05086	-2.73759	0.51866
C	3.05498	-1.62894	0.65296
H	2.07919	-3.17694	-0.48409
H	2.23896	-3.53353	1.24118
C	3.80360	-1.18651	-0.44308
C	4.70224	-0.13727	-0.30604
C	4.87167	0.47880	0.93166
C	4.16298	0.02571	2.03894
C	3.26515	-1.02478	1.90267
H	3.69888	-1.68903	-1.40119
H	5.28611	0.19181	-1.15923
H	5.57774	1.29522	1.03739
H	4.32188	0.48066	3.01040
H	2.72565	-1.39889	2.76856
H	-2.26764	2.17754	-2.06568

Gal-6B/conf_1.out

Charge=+1, Multiplicity=+1

C	-2.21760	-1.79557	-1.63515
O	-3.48929	-1.16737	-1.60720
C	-3.87365	-1.33403	-0.26016
C	-1.30625	-0.74228	-1.01613
H	-1.94138	-2.00183	-2.67000

C	-2.46915	-3.04700	-0.79864
C	-1.60533	-0.49308	0.45287
H	-1.46637	0.18235	-1.57440
O	0.10369	-1.09956	-1.18133
C	-3.13242	-0.31038	0.61327
O	-1.13092	-1.57019	1.21876
H	-1.10052	0.43289	0.75450
O	-3.59087	0.94917	0.19149
H	-3.37807	-0.49156	1.66953
O	-3.50637	-2.63985	0.09173
H	-4.95360	-1.19963	-0.19016
H	-1.60065	-3.37409	-0.22664
H	-2.83762	-3.85924	-1.43058
C	0.95399	-0.16474	-1.02452
C	2.33254	-0.37149	-1.22142
H	0.58685	0.83527	-0.77546
C	2.86515	-1.63939	-1.53411
C	4.21783	-1.76225	-1.75928
C	5.04314	-0.63465	-1.67718
C	4.52708	0.61834	-1.35818
C	3.17212	0.75414	-1.11998
H	2.20591	-2.49755	-1.59457
H	4.64523	-2.72769	-2.00379
H	6.10668	-0.74106	-1.86486
H	5.18332	1.47830	-1.29508
H	2.74189	1.71759	-0.86601
C	-0.51648	-1.19493	2.44594
C	0.92000	-0.78692	2.26258
H	-0.58326	-2.07926	3.08265
H	-1.08767	-0.39293	2.93066
C	1.31639	0.54512	2.38020
C	2.65488	0.90252	2.25162
C	3.61058	-0.07355	1.99974
C	3.22402	-1.40577	1.86718
C	1.88819	-1.75803	1.99533
H	0.57590	1.30501	2.60906
H	2.95227	1.93928	2.37056
H	4.65802	0.19753	1.92292

H	3.97086	-2.17155	1.68680
H	1.58983	-2.79909	1.91278
C	-3.07486	2.07398	0.87443
C	-1.85213	2.64976	0.19752
H	-3.87827	2.81706	0.87575
H	-2.85600	1.83168	1.92357
C	-1.77432	2.66917	-1.19698
C	-0.65303	3.18958	-1.83355
C	0.40249	3.70407	-1.08341
C	0.32320	3.70490	0.30510
C	-0.79979	3.18145	0.93952
H	-0.86182	3.19617	2.02445
H	1.26653	4.13157	-1.58196
H	1.12936	4.12638	0.89687
H	-2.60557	2.27850	-1.77613
H	-0.61143	3.21463	-2.91777

Gal-6B/conf_2.out

Charge=+1, Multiplicity=+1

C	2.13718	-1.32493	1.84247
O	3.41395	-0.79359	1.51482
C	3.70000	-1.43086	0.28311
C	1.19985	-0.50092	0.97269
H	1.94464	-1.16164	2.90358
C	2.29495	-2.79367	1.46072
C	1.40184	-0.73803	-0.51741
H	1.39561	0.54724	1.20439
O	-0.20002	-0.74898	1.31756
C	2.91325	-0.78860	-0.86816
O	0.75856	-1.94118	-0.86140
H	0.95946	0.10301	-1.06223
O	3.44420	0.42318	-1.35140
H	2.99582	-1.47315	-1.71649
O	3.28533	-2.76251	0.43679
H	4.77707	-1.38237	0.11148
H	1.37792	-3.23956	1.07298
H	2.67268	-3.37369	2.30647
C	-1.03449	0.15526	0.99379

C	-2.40989	0.03129	1.26821
H	-0.65349	1.06456	0.51769
C	-2.95897	-1.13701	1.83627
C	-4.30889	-1.18323	2.10203
C	-5.11498	-0.07629	1.81047
C	-4.58232	1.07752	1.24254
C	-3.23008	1.13319	0.96024
H	-2.31469	-1.98127	2.05286
H	-4.74951	-2.07195	2.53858
H	-6.17664	-0.12068	2.03083
H	-5.22356	1.92231	1.02068
H	-2.78990	2.01758	0.51150
C	0.14591	-1.91447	-2.14872
C	-1.17809	-1.20038	-2.12271
H	0.01222	-2.96150	-2.42523
H	0.82200	-1.45699	-2.88168
C	-2.32028	-1.86942	-1.67720
C	-3.55299	-1.23217	-1.66103
C	-3.65927	0.09107	-2.08231
C	-2.52867	0.77210	-2.51681
C	-1.29574	0.12736	-2.53862
H	-2.24127	-2.90665	-1.36461
H	-4.43703	-1.77166	-1.33801
H	-4.62671	0.58175	-2.09140
H	-2.61081	1.79548	-2.86825
H	-0.42361	0.64809	-2.92401
C	3.66452	1.51652	-0.47070
C	2.41746	2.31137	-0.16485
H	4.13928	1.19746	0.46087
H	4.37657	2.15156	-1.00547
C	2.18832	2.80561	1.11952
C	1.03373	3.52799	1.40799
C	0.09231	3.76221	0.41051
C	0.32115	3.28644	-0.87887
C	1.47905	2.57174	-1.16424
H	1.66827	2.21101	-2.17064
H	-0.79928	4.34121	0.62942
H	-0.39354	3.49369	-1.66903

H	2.91910	2.61875	1.90184
H	0.87400	3.91386	2.40936

Gal-6B/conf_3.out

Charge=+1, Multiplicity=+1

C	1.46596	2.24756	-2.14146
O	2.52266	1.30458	-2.16681
C	2.53914	0.83435	-0.83645
C	0.14096	1.46561	-2.03385
H	1.51225	2.84140	-3.05272
C	1.81927	3.00533	-0.87342
C	0.37898	-0.01878	-1.72173
H	-0.47092	1.55569	-2.93547
O	-0.64791	1.97515	-0.92390
C	1.45474	-0.24890	-0.65904
O	-0.90280	-0.49250	-1.39520
H	0.74093	-0.48928	-2.64447
O	1.92651	-1.55381	-0.87650
H	1.03206	-0.14345	0.34997
O	2.20736	1.94404	-0.01430
H	3.53641	0.45875	-0.61485
H	1.00230	3.56005	-0.41403
H	2.65960	3.67707	-1.07423
C	-1.84947	1.53944	-0.81931
C	-2.53254	1.61707	0.41673
H	-2.37494	1.22278	-1.71784
C	-1.87148	2.01411	1.59628
C	-2.58073	2.06821	2.77666
C	-3.93660	1.72451	2.79076
C	-4.59037	1.31666	1.63097
C	-3.89156	1.25721	0.44038
H	-0.81795	2.26914	1.56222
H	-2.09138	2.37544	3.69361
H	-4.48633	1.77078	3.72519
H	-5.63705	1.03906	1.66215
H	-4.37647	0.91481	-0.46820
C	-1.07143	-1.82490	-0.89570
C	-2.52263	-1.97068	-0.55732

H	-0.45164	-1.97984	-0.00928
H	-0.75037	-2.53099	-1.66934
C	-3.48624	-1.91263	-1.56563
C	-4.83629	-2.00268	-1.25277
C	-5.23485	-2.16160	0.07222
C	-4.27994	-2.22803	1.07947
C	-2.92911	-2.12630	0.76465
H	-3.17438	-1.80933	-2.60096
H	-5.57881	-1.97029	-2.04311
H	-6.28862	-2.25036	0.31448
H	-4.58552	-2.36310	2.11150
H	-2.18454	-2.17484	1.55402
C	2.41639	-2.22457	0.28523
C	3.66976	-1.62052	0.84839
H	1.62966	-2.24010	1.05418
H	2.58986	-3.24992	-0.04650
C	4.88293	-1.78044	0.17653
C	6.03312	-1.16478	0.64939
C	5.98304	-0.38534	1.80233
C	4.78259	-0.23134	2.48366
C	3.63116	-0.84855	2.00653
H	2.69263	-0.72687	2.54085
H	6.88282	0.09437	2.17201
H	4.74219	0.36875	3.38619
H	4.92035	-2.38416	-0.72547
H	6.97239	-1.29530	0.12286

Gal-6B/conf_4.out

Charge=+1, Multiplicity=+1

C	0.14391	3.62517	-1.38519
O	1.54561	3.41544	-1.34926
C	1.74851	2.96379	-0.02745
C	-0.52843	2.23862	-1.44523
H	-0.09014	4.21983	-2.26668
C	-0.06000	4.36496	-0.07223
C	0.45199	1.11283	-1.06888
H	-0.95062	2.03765	-2.43422
O	-1.61129	2.14311	-0.48892

C	1.38759	1.47191	0.07638
O	-0.33163	-0.00909	-0.74890
H	1.07719	0.92072	-1.94694
O	2.47200	0.59956	-0.06409
H	0.87791	1.31089	1.03617
O	0.85460	3.70228	0.78835
H	2.78548	3.16496	0.24580
H	-1.06457	4.29964	0.34459
H	0.22085	5.41481	-0.19933
C	-2.35937	1.10302	-0.59031
C	-3.14985	0.67396	0.49944
H	-2.47514	0.63297	-1.56519
C	-3.03067	1.26428	1.77349
C	-3.82215	0.80410	2.80325
C	-4.72111	-0.24321	2.57570
C	-4.83079	-0.84137	1.32289
C	-4.04483	-0.38863	0.28048
H	-2.32078	2.06850	1.93016
H	-3.74785	1.24845	3.78892
H	-5.33932	-0.59919	3.39337
H	-5.52200	-1.66098	1.16696
H	-4.09807	-0.85891	-0.69646
C	-0.02159	-1.24346	-1.41993
C	-1.18693	-2.16007	-1.22228
H	0.90150	-1.64148	-0.98753
H	0.14636	-1.04878	-2.48521
C	-1.39980	-2.76566	0.01592
C	-2.50650	-3.57891	0.21837
C	-3.41028	-3.79790	-0.81776
C	-3.20454	-3.20053	-2.05627
C	-2.09692	-2.38179	-2.25488
H	-0.69037	-2.60147	0.82135
H	-2.65874	-4.05480	1.18118
H	-4.26476	-4.44869	-0.66493
H	-3.89589	-3.38431	-2.87177
H	-1.92567	-1.93252	-3.22965
C	3.23882	0.39938	1.12416
C	4.12613	-0.78501	0.91067

H	3.83171	1.29487	1.34903
H	2.55452	0.22307	1.96547
C	5.40590	-0.62845	0.38462
C	6.20738	-1.73749	0.14336
C	5.73321	-3.01300	0.42719
C	4.45716	-3.17807	0.95531
C	3.65891	-2.06730	1.19474
H	2.66623	-2.19373	1.61914
H	6.36099	-3.87847	0.24454
H	4.09009	-4.17193	1.18843
H	5.77748	0.36791	0.16416
H	7.20465	-1.60636	-0.26235

Gal-6B/conf_5.out

Charge=+1, Multiplicity=+1

C	-1.70342	-1.76367	-1.49787
O	-3.06455	-1.62922	-1.11408
C	-3.06974	-2.15699	0.19753
C	-1.04969	-0.55774	-0.81791
H	-1.63423	-1.68389	-2.58413
C	-1.39299	-3.16539	-0.96835
C	-1.10854	-0.62127	0.70499
H	-1.60936	0.31266	-1.15540
O	0.30458	-0.22772	-1.23093
C	-2.48470	-1.16143	1.20379
O	-0.05676	-1.45468	1.13871
H	-0.98262	0.39211	1.09332
O	-3.37151	-0.16767	1.64344
H	-2.25590	-1.73703	2.10498
O	-2.24818	-3.29864	0.15364
H	-4.09317	-2.43743	0.45345
H	-0.36291	-3.32530	-0.64514
H	-1.65476	-3.91432	-1.72168
C	1.28612	-1.02157	-1.06047
C	2.60826	-0.58126	-1.29852
H	1.09571	-2.05335	-0.78466
C	2.89948	0.76821	-1.57637
C	4.20499	1.13704	-1.82286

C	5.21829	0.17656	-1.78938
C	4.93918	-1.15952	-1.50350
C	3.63657	-1.54165	-1.25345
H	2.09808	1.49757	-1.58643
H	4.44719	2.17123	-2.03790
H	6.24248	0.47615	-1.98636
H	5.73890	-1.89029	-1.47921
H	3.39880	-2.57639	-1.02687
C	0.52046	-1.07057	2.39923
C	1.63365	-0.08253	2.20968
H	0.89177	-1.99560	2.84286
H	-0.26550	-0.67370	3.05073
C	1.38251	1.28802	2.11883
C	2.41785	2.18006	1.86848
C	3.72032	1.71285	1.72619
C	3.98518	0.35283	1.84010
C	2.94575	-0.53748	2.07610
H	0.37629	1.66809	2.26881
H	2.21237	3.24359	1.80850
H	4.53113	2.41188	1.55051
H	5.00310	-0.01254	1.75593
H	3.15459	-1.59936	2.17401
C	-4.15691	0.56369	0.70643
C	-3.38676	1.62312	-0.03546
H	-4.65517	-0.10096	-0.00531
H	-4.93145	1.02686	1.32333
C	-2.50766	2.46422	0.64831
C	-1.77510	3.42476	-0.03727
C	-1.92711	3.56794	-1.41386
C	-2.82273	2.75370	-2.09782
C	-3.54569	1.78315	-1.41082
H	-4.23148	1.13438	-1.94792
H	-1.36398	4.32582	-1.94802
H	-2.95868	2.87269	-3.16757
H	-2.40641	2.36162	1.72491
H	-1.09881	4.07839	0.50435

Gal-6B/conf_6.out

Charge=+1, Multiplicity=+1

C	1.32224	3.03799	-0.33040
O	2.27820	2.53262	0.59014
C	1.44918	1.95967	1.57912
C	0.64263	1.84120	-1.03064
H	1.83744	3.68537	-1.03768
C	0.39310	3.75271	0.63461
C	1.03691	0.49649	-0.40719
H	0.84011	1.81970	-2.10587
O	-0.79739	1.93501	-0.85711
C	1.01918	0.55431	1.13492
O	0.14497	-0.41794	-0.99863
H	2.05903	0.29127	-0.74146
O	1.76790	-0.47282	1.74341
H	-0.00348	0.39954	1.48590
O	0.30925	2.79629	1.68279
H	1.98861	1.93818	2.52768
H	-0.60821	3.96089	0.26150
H	0.86440	4.67764	0.98049
C	-1.52609	1.15648	-1.56470
C	-2.85743	0.88253	-1.17512
H	-1.14523	0.81572	-2.52622
C	-3.30641	1.17221	0.12770
C	-4.61080	0.88382	0.46729
C	-5.46924	0.32382	-0.48185
C	-5.03007	0.03230	-1.77208
C	-3.71995	0.29704	-2.11928
H	-2.61936	1.59922	0.84990
H	-4.96970	1.08617	1.46945
H	-6.49568	0.10442	-0.20662
H	-5.71001	-0.40522	-2.49353
H	-3.35505	0.06736	-3.11558
C	0.11544	-1.78980	-0.53384
C	-1.13588	-2.07389	0.24200
H	1.00605	-1.97885	0.06264
H	0.16004	-2.39976	-1.43868
C	-1.14725	-2.01066	1.63560
C	-2.32323	-2.24130	2.34002

C	-3.49617	-2.54610	1.65864
C	-3.48848	-2.63158	0.27051
C	-2.31301	-2.39919	-0.43134
H	-0.22483	-1.79825	2.16825
H	-2.31984	-2.19965	3.42404
H	-4.41004	-2.73980	2.21035
H	-4.39497	-2.89687	-0.26333
H	-2.30302	-2.48881	-1.51435
C	3.19978	-0.40032	1.71452
C	3.81876	-0.88746	0.43265
H	3.54591	0.61549	1.92849
H	3.50800	-1.03604	2.54746
C	4.53145	-0.01593	-0.38948
C	5.08442	-0.46594	-1.58394
C	4.92648	-1.79285	-1.96616
C	4.22984	-2.67311	-1.14312
C	3.68549	-2.22329	0.05235
H	3.16211	-2.91680	0.70487
H	5.35994	-2.14640	-2.89547
H	4.12631	-3.71497	-1.42724
H	4.64809	1.02232	-0.09168
H	5.64448	0.21800	-2.21288

Gal-6B/conf_7.out

Charge=+1, Multiplicity=+1

C	-1.15315	-2.94586	-0.27823
O	-2.29679	-2.10640	-0.29315
C	-1.90471	-1.06340	0.57669
C	-0.01441	-2.22457	-1.03254
H	-1.41570	-3.89226	-0.74778
C	-0.90727	-3.01878	1.21815
C	-0.38410	-0.77955	-1.38720
H	0.29827	-2.76059	-1.93282
O	1.14922	-2.11628	-0.16782
C	-1.04636	-0.05114	-0.20207
O	0.81851	-0.22942	-1.86416
H	-1.11450	-0.84584	-2.20351
O	-1.73592	1.09847	-0.63430

H	-0.27436	0.31637	0.47697
O	-1.10890	-1.66300	1.58856
H	-2.79672	-0.60744	1.00412
H	0.09286	-3.32686	1.51927
H	-1.65481	-3.67117	1.67972
C	2.25840	-1.77364	-0.70355
C	3.30530	-1.26395	0.09603
H	2.41339	-1.98085	-1.76140
C	3.06660	-0.83348	1.41603
C	4.11540	-0.33591	2.15878
C	5.39542	-0.27645	1.60209
C	5.63757	-0.69778	0.29550
C	4.59219	-1.17977	-0.46674
H	2.06267	-0.88178	1.82332
H	3.94837	0.01302	3.17091
H	6.21581	0.11277	2.19620
H	6.63694	-0.64021	-0.11940
H	4.75709	-1.50281	-1.48994
C	0.90218	1.19096	-2.14033
C	1.71703	1.89846	-1.09837
H	-0.10412	1.60411	-2.20238
H	1.38180	1.26094	-3.11898
C	3.10873	1.89787	-1.19020
C	3.88235	2.51352	-0.21513
C	3.26840	3.14255	0.86266
C	1.88120	3.16485	0.95276
C	1.10841	2.54953	-0.02525
H	3.58903	1.42673	-2.04371
H	4.96355	2.51918	-0.30476
H	3.86974	3.63559	1.61921
H	1.39878	3.67698	1.77849
H	0.02458	2.58664	0.03074
C	-2.98155	0.91899	-1.31543
C	-4.16483	0.89624	-0.38676
H	-3.05046	1.77337	-1.99521
H	-2.97386	0.00942	-1.92706
C	-4.28656	1.85831	0.61607
C	-5.38132	1.84459	1.46914

C	-6.36895	0.87447	1.32302
C	-6.25595	-0.08208	0.32223
C	-5.15455	-0.07276	-0.52718
H	-5.05916	-0.83224	-1.29791
H	-7.22508	0.86636	1.98885
H	-7.02197	-0.84110	0.20551
H	-3.51413	2.61229	0.73026
H	-5.46957	2.59531	2.24722

Gal-4B/conf_1.out

Charge=+1, Multiplicity=+1

O	-0.54647	-1.57371	0.78024
C	-0.36277	-0.52992	-0.12841
C	0.05852	-2.58585	-0.03489
O	-1.01203	-1.00463	-1.32575
C	1.46920	-2.04479	-0.25055
C	1.16132	-0.52520	-0.37191
H	1.92079	-2.40012	-1.18700
O	2.24343	-2.41244	0.85176
O	1.56117	-0.03986	-1.61616
H	1.64325	0.00985	0.45217
H	-0.83696	0.39196	0.19665
C	-0.72381	-2.39837	-1.34978
C	-2.05838	-3.13246	-1.46060
H	-0.12074	-2.64187	-2.23045
H	-0.00146	-3.56163	0.44191
C	1.80059	1.36655	-1.63904
C	0.58041	2.16841	-1.28145
H	2.12565	1.57729	-2.65962
H	2.62535	1.60265	-0.95632
C	-0.51065	2.21875	-2.15029
C	-1.66734	2.89650	-1.78926
C	-1.74805	3.52830	-0.55063
C	-0.66401	3.48961	0.31843
C	0.49591	2.81478	-0.04972
H	-0.44705	1.72156	-3.11333
H	-2.50005	2.95300	-2.48370
H	-2.64562	4.07158	-0.27360

H	-0.71600	3.99456	1.27723
H	1.34658	2.78585	0.62601
C	3.64359	-2.21922	0.69075
C	4.08166	-0.78141	0.78995
H	3.96938	-2.64527	-0.26784
H	4.09575	-2.80868	1.49198
C	4.78414	-0.17608	-0.24703
C	5.19417	1.15072	-0.14505
C	4.88983	1.88600	0.99331
C	4.18349	1.28791	2.03491
C	3.78914	-0.03921	1.93638
H	5.01216	-0.74439	-1.14409
H	5.74949	1.60875	-0.95678
H	5.20968	2.91935	1.07548
H	3.24481	-0.50897	2.75047
H	3.95673	1.85442	2.93222
O	-2.93631	-2.55674	-0.48105
H	-1.98227	-4.19269	-1.22688
H	-2.49441	-2.99708	-2.45490
C	-3.29064	-1.34341	-0.71225
C	-3.73159	-0.50226	0.33232
H	-3.34729	-1.00810	-1.74667
C	-4.27622	0.74889	-0.01550
C	-4.70320	1.60598	0.97901
C	-4.57859	1.22363	2.31363
C	-4.02393	-0.00976	2.66673
C	-3.59647	-0.87721	1.68453
H	-4.35372	1.03150	-1.06025
H	-5.13051	2.56887	0.72579
H	-4.91284	1.89903	3.09442
H	-3.92580	-0.27963	3.71166
H	-3.14106	-1.82814	1.93159

Gal-4B/conf_2.out

Charge=+1, Multiplicity=+1

O	-1.31869	-0.01458	-0.82230
C	-1.16879	-1.29705	-0.30022
C	-0.23365	0.56983	-0.08210

O	-1.27177	-1.09838	1.12260
C	0.96823	-0.27951	-0.51765
C	0.27689	-1.66414	-0.65529
H	1.73900	-0.31941	0.25764
O	1.50471	0.11586	-1.74663
O	0.72732	-2.67752	0.18640
H	0.32147	-1.95959	-1.71260
H	-1.95268	-1.97797	-0.62273
C	-0.58961	0.13057	1.34939
C	-1.51659	1.05385	2.12365
H	0.30537	-0.03707	1.95856
H	-0.17108	1.63874	-0.26377
C	1.91650	-3.32864	-0.24453
C	3.16680	-2.52543	-0.00092
H	1.83600	-3.58878	-1.30803
H	1.94643	-4.25832	0.32922
C	3.45223	-2.05141	1.28149
C	4.60021	-1.30803	1.51885
C	5.48703	-1.04205	0.47760
C	5.21594	-1.52065	-0.79765
C	4.05550	-2.25287	-1.03553
H	2.76682	-2.27321	2.09467
H	4.81746	-0.95090	2.52038
H	6.39023	-0.47108	0.66433
H	5.90514	-1.32143	-1.61146
H	3.83891	-2.61179	-2.03744
C	2.61360	1.01006	-1.62441
C	2.27062	2.25832	-0.86048
H	2.90040	1.24014	-2.65182
H	3.44433	0.48494	-1.13853
C	1.51389	3.26898	-1.45512
C	1.09631	4.36703	-0.71399
C	1.43420	4.46866	0.63358
C	2.20552	3.47713	1.22979
C	2.62317	2.37933	0.48290
H	1.24333	3.18547	-2.50369
H	0.51546	5.15136	-1.18792
H	1.11706	5.33189	1.20935

H	3.23500	1.60767	0.94357
H	2.49651	3.56705	2.27151
O	-2.76353	1.11329	1.40478
H	-1.13788	2.07360	2.16928
H	-1.70697	0.67164	3.13074
C	-3.48849	0.06002	1.44784
C	-4.49707	-0.17125	0.48900
H	-3.35708	-0.62441	2.28524
C	-5.37835	-1.24899	0.70729
C	-6.37432	-1.51127	-0.21210
C	-6.48580	-0.71029	-1.34832
C	-5.60645	0.35175	-1.57820
C	-4.60934	0.62731	-0.66754
H	-5.27205	-1.86216	1.59682
H	-7.06280	-2.33294	-0.05470
H	-7.26668	-0.91981	-2.07207
H	-5.70568	0.95128	-2.47544
H	-3.89941	1.42815	-0.83391

Gal-4B/conf_3.out
Charge=+1, Multiplicity=+1

O	0.35446	1.78967	-1.51956
C	0.10225	0.68115	-0.70472
C	0.71637	2.67599	-0.45202
O	-0.88430	1.16125	0.22501
C	1.88233	1.96161	0.21965
C	1.43118	0.48443	0.05762
H	1.95950	2.19767	1.28993
O	3.04958	2.31509	-0.45907
O	1.32845	-0.12483	1.30641
H	2.13237	-0.04258	-0.59803
H	-0.29850	-0.16241	-1.26054
C	-0.53601	2.52824	0.43212
C	-1.69148	3.38913	-0.09074
H	-0.35669	2.69864	1.49515
H	0.93637	3.67306	-0.82881
C	1.38691	-1.54744	1.26677
C	0.20136	-2.15501	0.57046

H	1.42331	-1.85423	2.31411
H	2.32107	-1.86057	0.78500
C	0.34650	-2.80234	-0.65443
C	-0.76402	-3.30882	-1.32378
C	-2.02984	-3.17202	-0.76632
C	-2.18194	-2.53349	0.46235
C	-1.07308	-2.02790	1.12477
H	1.33761	-2.91091	-1.08657
H	-0.63870	-3.81823	-2.27335
H	-2.89440	-3.58169	-1.27882
H	-3.16598	-2.44388	0.91085
H	-1.18939	-1.52443	2.07946
C	4.25760	1.94212	0.19424
C	4.58065	0.47439	0.09665
H	4.21896	2.24892	1.24823
H	5.03066	2.53428	-0.30117
C	4.77053	-0.29222	1.24195
C	5.07796	-1.64657	1.14449
C	5.18352	-2.24645	-0.10381
C	4.99155	-1.48618	-1.25558
C	4.69954	-0.13299	-1.15535
H	4.67603	0.17145	2.21958
H	5.23309	-2.23189	2.04474
H	5.42482	-3.30120	-0.18227
H	4.55640	0.46236	-2.05242
H	5.08678	-1.94791	-2.23284
O	-2.90943	2.66944	0.17419
H	-1.60421	3.53873	-1.17012
H	-1.78115	4.34307	0.42406
C	-3.07427	1.61395	-0.53596
C	-4.04401	0.64245	-0.19781
H	-2.54935	1.55011	-1.48886
C	-4.70618	0.65536	1.04499
C	-5.66480	-0.30020	1.30628
C	-5.97332	-1.26103	0.33795
C	-5.31668	-1.28411	-0.88996
C	-4.34206	-0.34151	-1.15784
H	-4.45600	1.41216	1.77934

H	-6.18314	-0.30620	2.25801
H	-6.73563	-2.00362	0.54979
H	-5.56543	-2.03800	-1.62748
H	-3.81183	-0.35097	-2.10473

Gal-4B/conf_4.out

Charge=+1, Multiplicity=+1

O	-0.31588	-1.40848	0.80639
C	-0.49439	-0.11958	0.30544
C	0.83964	-1.71560	0.01305
O	-0.59367	-0.31553	-1.11961
C	1.82522	-0.62105	0.40533
C	0.85321	0.57334	0.60437
H	2.53647	-0.38874	-0.39718
O	2.48330	-1.01460	1.57116
O	1.19282	1.63303	-0.23558
H	0.85867	0.87429	1.66126
H	-1.40809	0.34306	0.66747
C	0.31540	-1.37671	-1.39470
C	-0.46848	-2.46698	-2.12107
H	1.11295	-1.02660	-2.05790
H	1.17695	-2.73473	0.18665
C	0.60894	2.87972	0.12141
C	-0.88520	2.91740	-0.05785
H	1.09331	3.61036	-0.52978
H	0.86841	3.12381	1.16078
C	-1.43870	2.79310	-1.33286
C	-2.81588	2.80156	-1.50629
C	-3.65762	2.93066	-0.40406
C	-3.11604	3.05409	0.86969
C	-1.73467	3.05023	1.03841
H	-0.78109	2.69265	-2.19049
H	-3.23499	2.73344	-2.50549
H	-4.73382	2.96215	-0.54179
H	-3.76593	3.17008	1.73056
H	-1.31228	3.15816	2.03378
C	3.54200	-0.13511	1.94905
C	4.64452	-0.08205	0.92922

H	3.90087	-0.53256	2.90019
H	3.15020	0.87492	2.13183
C	4.90275	1.08509	0.21494
C	5.91143	1.11980	-0.74255
C	6.66495	-0.01894	-0.99653
C	6.41021	-1.19167	-0.29035
C	5.40625	-1.22206	0.66731
H	4.30753	1.97304	0.40829
H	6.10855	2.03601	-1.28884
H	7.45509	0.00650	-1.73932
H	5.21041	-2.13437	1.22313
H	7.00466	-2.07912	-0.48020
O	-1.67512	-2.69116	-1.37472
H	0.05835	-3.41876	-2.15453
H	-0.72994	-2.14645	-3.13392
C	-2.52050	-1.72461	-1.39376
C	-3.49022	-1.58646	-0.37786
H	-2.53720	-1.06590	-2.26066
C	-4.48096	-0.59960	-0.54509
C	-5.43413	-0.41885	0.43729
C	-5.39668	-1.21171	1.58307
C	-4.40693	-2.18263	1.76264
C	-3.44895	-2.37451	0.79070
H	-4.48338	0.01557	-1.43870
H	-6.20424	0.33434	0.32100
H	-6.14613	-1.06822	2.35463
H	-4.39080	-2.77774	2.66813
H	-2.65714	-3.10260	0.91617

Gal-4B/conf_5.out

Charge=+1, Multiplicity=+1

O	0.01226	1.48351	-0.66990
C	-0.06133	0.62955	0.43550
C	0.43946	2.63985	0.06723
O	-0.96733	1.31297	1.32464
C	1.74166	2.16250	0.72127
C	1.35349	0.69383	1.04793
H	1.93720	2.68549	1.65992

O	2.89826	2.35919	-0.04116
O	1.40687	0.46904	2.42414
H	2.00116	0.00286	0.50417
H	-0.46983	-0.34366	0.17851
C	-0.64176	2.69005	1.16608
C	-1.93870	3.41830	0.81746
H	-0.25740	3.09549	2.10738
H	0.51021	3.51368	-0.57813
C	1.61472	-0.90017	2.76821
C	0.52982	-1.79746	2.24029
H	1.64667	-0.91452	3.85885
H	2.59102	-1.22490	2.38624
C	-0.74332	-1.77341	2.81148
C	-1.77134	-2.53500	2.27175
C	-1.53865	-3.32468	1.14831
C	-0.27272	-3.35770	0.57535
C	0.75723	-2.60048	1.12398
H	-0.92385	-1.15317	3.68421
H	-2.75045	-2.53151	2.74090
H	-2.33718	-3.93199	0.73469
H	-0.08194	-3.98112	-0.29189
H	1.74464	-2.62481	0.67136
C	2.94121	1.89098	-1.38710
C	3.13830	0.40262	-1.51479
H	3.79699	2.41814	-1.81569
H	2.04982	2.19803	-1.94440
C	4.22168	-0.21262	-0.88477
C	4.39705	-1.58700	-0.97065
C	3.49727	-2.36228	-1.69873
C	2.42448	-1.75498	-2.33960
C	2.24525	-0.37823	-2.24378
H	4.91930	0.39262	-0.31400
H	5.24358	-2.05571	-0.47996
H	3.64157	-3.43502	-1.77378
H	1.39805	0.09624	-2.72977
H	1.72659	-2.35341	-2.91626
O	-2.58277	2.65260	-0.21261
H	-1.77377	4.41420	0.41077

H	-2.59909	3.47332	1.68807
C	-3.01199	1.49920	0.15927
C	-3.22236	0.46332	-0.77785
H	-3.32221	1.37053	1.19500
C	-3.85905	-0.70942	-0.32952
C	-4.06765	-1.75132	-1.21146
C	-3.63584	-1.62966	-2.53076
C	-2.98926	-0.47414	-2.97885
C	-2.77649	0.57471	-2.11020
H	-4.17790	-0.78819	0.70465
H	-4.56250	-2.65687	-0.88137
H	-3.80027	-2.44916	-3.22279
H	-2.65476	-0.40615	-4.00745
H	-2.26083	1.47296	-2.42633

Gal-4B/conf_6.out

Charge=+1, Multiplicity=+1

O	-0.84229	-1.56348	-1.05965
C	-0.35187	-0.58480	-0.18345
C	-0.97242	-2.59350	-0.06660
O	0.80943	-1.20292	0.39436
C	-1.90784	-1.96973	0.97328
C	-1.43946	-0.49130	0.90803
H	-1.69923	-2.35010	1.97559
O	-3.26874	-2.22240	0.77328
O	-0.99592	-0.07484	2.16307
H	-2.24427	0.14817	0.53913
H	-0.06686	0.32703	-0.70113
C	0.46328	-2.58235	0.49300
C	1.42341	-3.36395	-0.40986
H	0.53880	-2.90244	1.53367
H	-1.30270	-3.53037	-0.51357
C	-1.05454	1.33710	2.34795
C	-0.14082	2.07379	1.40898
H	-0.76418	1.49717	3.38780
H	-2.08934	1.67941	2.21836
C	1.24193	1.98627	1.57356
C	2.09828	2.59311	0.66604

C	1.58040	3.29076	-0.42267
C	0.20375	3.38745	-0.59179
C	-0.65246	2.78321	0.32404
H	1.64317	1.43480	2.41837
H	3.17136	2.53554	0.81519
H	2.24809	3.77796	-1.12628
H	-0.20475	3.94164	-1.43030
H	-1.72839	2.85415	0.18822
C	-3.85873	-1.91687	-0.48908
C	-4.11669	-0.44808	-0.70185
H	-4.80310	-2.46616	-0.47227
H	-3.25870	-2.31359	-1.31533
C	-4.84542	0.27452	0.24464
C	-5.04638	1.63855	0.08410
C	-4.53186	2.29426	-1.03228
C	-3.82186	1.57745	-1.98772
C	-3.61323	0.21165	-1.81986
H	-5.23777	-0.23681	1.11829
H	-5.61460	2.19211	0.82426
H	-4.69744	3.35879	-1.16107
H	-3.04252	-0.34597	-2.55648
H	-3.43039	2.08091	-2.86568
O	2.70209	-2.70743	-0.33211
H	1.08319	-3.34036	-1.44861
H	1.58381	-4.38790	-0.07977
C	2.75034	-1.55556	-0.89529
C	3.80732	-0.65314	-0.63859
H	2.03184	-1.34057	-1.68573
C	3.91450	0.47751	-1.46887
C	4.95315	1.36881	-1.27808
C	5.86803	1.14560	-0.25174
C	5.75516	0.03496	0.59052
C	4.73144	-0.86873	0.40219
H	3.18538	0.64124	-2.25592
H	5.05327	2.23711	-1.91862
H	6.68155	1.84744	-0.09987
H	6.47453	-0.11471	1.38712
H	4.62802	-1.73790	1.04132

Man-oxo/conf_1.out
Charge=+1, Multiplicity=+1

C	-0.53178	-1.17253	-0.61448
C	-0.84509	0.31332	-0.43674
C	-0.46624	0.84036	0.94617
H	-1.91614	0.47741	-0.57804
O	-0.09934	1.04895	-1.37278
O	-1.00654	-0.03378	1.98526
H	0.62094	0.81346	1.07287
C	-1.00767	2.25275	1.08470
C	-0.72141	-1.26048	1.85699
C	0.07099	-1.74121	0.74155
O	0.18753	-3.10840	0.81426
H	1.06002	-1.23313	0.80332
H	-1.09322	-1.91782	2.64271
C	1.09605	-3.67292	-0.15552
H	0.26359	-1.25264	-1.35730
O	-1.55091	-1.99582	-1.06495
C	-2.67534	-2.20235	-0.23133
O	-1.10838	2.80467	-0.21859
H	-0.37022	2.86494	1.72914
H	-2.02395	2.22623	1.48494
C	-0.05175	2.45017	-1.07207
C	1.30718	2.82967	-0.52060
H	-0.25731	2.97085	-2.00931
C	1.47828	4.10445	0.01998
C	2.71001	4.48483	0.53490
C	3.78143	3.59711	0.51020
C	3.61805	2.33217	-0.04020
C	2.38603	1.94889	-0.55942
H	0.64178	4.79652	0.03749
H	2.83576	5.47697	0.95447
H	4.74336	3.89668	0.91166
H	4.45198	1.63929	-0.07411
H	2.26690	0.96894	-1.00872
C	2.39955	-2.93028	-0.20980
H	0.60748	-3.68510	-1.13488

H	1.22640	-4.70256	0.17926
C	3.19544	-2.81972	0.93443
C	4.38042	-2.09781	0.89772
C	4.78806	-1.48656	-0.28514
C	4.00856	-1.59841	-1.42976
C	2.81574	-2.31298	-1.38888
H	2.88577	-3.31110	1.85255
H	4.99679	-2.02460	1.78717
H	5.72342	-0.93790	-0.31666
H	4.32988	-1.13529	-2.35646
H	2.21388	-2.40930	-2.28877
C	-3.66284	-1.06828	-0.19712
H	-2.33601	-2.44509	0.79570
H	-3.13826	-3.11571	-0.61361
C	-4.16604	-0.59901	1.01291
C	-5.08045	0.44971	1.03780
C	-5.49080	1.03818	-0.15124
C	-4.99952	0.56530	-1.36546
C	-4.09496	-0.48631	-1.39029
H	-3.85586	-1.06072	1.94702
H	-5.47228	0.80403	1.98516
H	-6.19935	1.85892	-0.13515
H	-5.32766	1.01586	-2.29574
H	-3.70798	-0.85260	-2.33608

Man-oxo/conf_2.out

Charge=+1, Multiplicity=+1

C	0.99024	-0.49274	-0.40687
C	-0.17207	-0.24624	0.53802
C	0.28329	0.23033	1.91252
H	-0.70735	-1.19426	0.70618
O	-1.03561	0.70587	-0.00186
O	1.06873	-0.85821	2.50839
H	0.93364	1.10977	1.86074
C	-0.93982	0.47996	2.77844
C	1.95503	-1.37518	1.77144
C	2.28510	-0.85025	0.46136
O	3.18949	-1.69009	-0.14248

H	2.73637	0.15071	0.67423
H	2.48714	-2.22478	2.20182
C	3.89332	-1.09326	-1.25815
H	1.21303	0.43705	-0.93511
O	0.80027	-1.44792	-1.38607
C	0.36460	-2.73707	-0.98144
O	-1.76855	1.37776	2.08706
H	-0.68016	0.93590	3.73443
H	-1.45135	-0.47700	2.96360
C	-2.17753	0.87831	0.83301
C	-3.11615	1.83685	0.18481
H	-2.64834	-0.10877	0.97085
C	-4.38383	1.40626	-0.18954
C	-5.26575	2.28944	-0.80121
C	-4.87713	3.60197	-1.03725
C	-3.60823	4.03330	-0.66073
C	-2.72689	3.15337	-0.05017
H	-4.68283	0.37970	0.00044
H	-6.25606	1.95437	-1.08933
H	-5.56464	4.29321	-1.51253
H	-3.30893	5.05982	-0.84193
H	-1.73950	3.48547	0.25092
C	4.43560	0.25987	-0.91163
H	3.21494	-1.04300	-2.11424
H	4.68561	-1.80965	-1.47626
C	3.94030	1.40392	-1.53884
C	4.41638	2.66319	-1.18872
C	5.38611	2.78643	-0.20161
C	5.88780	1.65034	0.42953
C	5.41760	0.39410	0.07503
H	3.19436	1.30552	-2.32319
H	4.03524	3.54527	-1.69146
H	5.76204	3.76683	0.06995
H	6.65718	1.74652	1.18791
H	5.81884	-0.49345	0.55608
C	-1.12683	-2.83897	-0.78606
H	0.89631	-3.04796	-0.06586
H	0.70002	-3.40619	-1.77787

C	-1.99467	-2.08695	-1.57598
C	-3.36795	-2.18436	-1.38899
C	-3.88490	-3.03614	-0.41829
C	-3.02281	-3.79574	0.36530
C	-1.64846	-3.69478	0.18315
H	-1.59245	-1.41415	-2.32532
H	-4.03538	-1.59484	-2.00849
H	-4.95751	-3.11462	-0.27718
H	-3.42037	-4.46703	1.11880
H	-0.97859	-4.28928	0.79960

Man-oxo/conf_3.out

Charge=+1, Multiplicity=+1

C	1.28291	-0.28164	0.01619
C	-0.03345	-0.37851	0.76656
C	0.13125	-0.21140	2.27295
H	-0.46119	-1.38273	0.62030
O	-0.91946	0.59376	0.30324
O	0.95042	-1.33827	2.73670
H	0.65618	0.71097	2.54181
C	-1.23562	-0.29632	2.93031
C	2.01074	-1.56184	2.08589
C	2.47920	-0.69086	1.02147
O	3.56535	-1.25884	0.40002
H	2.70896	0.27976	1.50874
H	2.56591	-2.45186	2.38606
C	4.67052	-0.35981	0.12976
H	1.46556	0.75865	-0.26074
O	1.37958	-1.00687	-1.14916
C	1.09509	-2.39362	-1.09339
O	-2.05638	0.66366	2.31737
H	-1.19512	-0.06734	3.99578
H	-1.63697	-1.31244	2.79582
C	-2.19108	0.45182	0.92927
C	-3.13324	1.45095	0.35101
H	-2.54305	-0.57753	0.75144
C	-4.24493	1.01244	-0.35917
C	-5.12602	1.93504	-0.91093

C	-4.89271	3.29496	-0.75077
C	-3.78002	3.73375	-0.03849
C	-2.89950	2.81449	0.51237
H	-4.42385	-0.05214	-0.47783
H	-5.99547	1.59258	-1.46117
H	-5.58054	4.01646	-1.17824
H	-3.60271	4.79605	0.08908
H	-2.03430	3.15025	1.07320
C	4.22257	0.93386	-0.47318
H	5.30928	-0.93094	-0.54334
H	5.21255	-0.18651	1.06650
C	3.81397	0.98897	-1.80700
C	3.31352	2.16972	-2.33597
C	3.21435	3.30697	-1.53798
C	3.63231	3.26689	-0.21355
C	4.13846	2.08343	0.31493
H	3.87770	0.09954	-2.42500
H	3.00112	2.20710	-3.37376
H	2.82364	4.22863	-1.95524
H	3.57777	4.15744	0.40298
H	4.49660	2.06052	1.34167
C	-0.37489	-2.71630	-1.18844
H	1.52729	-2.83406	-0.17691
H	1.64429	-2.82725	-1.93298
C	-0.89436	-3.82615	-0.52391
C	-2.24864	-4.12726	-0.61150
C	-3.09355	-3.31629	-1.36153
C	-2.57750	-2.21070	-2.02980
C	-1.22323	-1.91169	-1.94746
H	-0.23854	-4.46207	0.06571
H	-2.64409	-4.99449	-0.09385
H	-4.15034	-3.55064	-1.43045
H	-3.22991	-1.57896	-2.62323
H	-0.82366	-1.04205	-2.45755

Man-oxo/conf_4.out

Charge=+1, Multiplicity=+1

C	-0.66118500	-1.55199300	-1.32204100

C	-0.06034200	-2.91000900	-1.00392700
O	1.32933000	-2.73559200	-0.88586900
C	1.66988700	-1.81727600	0.12807800
C	3.15131700	-1.67572800	0.23239900
C	3.89951700	-1.33037200	-0.88981900
C	5.26774800	-1.13642700	-0.77261200
C	5.89084500	-1.28258300	0.46368400
C	5.14446700	-1.62939700	1.58306000
C	3.77311300	-1.82564500	1.46621100
H	3.18716000	-2.09523000	2.34013700
H	5.62918700	-1.75126900	2.54552300
H	6.96151100	-1.13219200	0.55200200
H	5.85258200	-0.87105700	-1.64670300
H	3.40803700	-1.21161600	-1.84879900
O	1.13316100	-0.54067200	-0.17716500
C	-0.25925500	-0.56197900	-0.24803600
C	-0.77241200	0.83590900	-0.52640300
C	-2.15339100	0.75180100	-1.27690500
C	-2.79349700	-0.58998700	-1.23886600
O	-2.12172300	-1.66086900	-1.34765700
H	-3.86698100	-0.72168400	-1.35915700
O	-3.03168900	1.80327400	-1.10445800
C	-3.71348100	1.94315600	0.17065300
C	-4.32421100	0.64860700	0.59597300
C	-5.61149700	0.29296500	0.18540300
C	-6.10867200	-0.97261300	0.46183600
C	-5.32444100	-1.90356900	1.14667000
C	-4.05322400	-1.55574200	1.57814700
C	-3.55110200	-0.28203500	1.30451400
H	-2.57795800	0.02391700	1.67707000
H	-3.45488000	-2.26609300	2.13867900
H	-5.71947500	-2.89117200	1.35769200
H	-7.11136200	-1.24036700	0.14658100
H	-6.21869300	1.01173000	-0.35614500
H	-4.47352200	2.69640000	-0.03375100
H	-3.01456000	2.31574000	0.91697500
H	-1.89246100	0.79783000	-2.35520000
O	-0.87727300	1.53358200	0.67090900

C	-0.23752500	2.83145400	0.68639800
C	1.23098000	2.73965600	0.40662300
C	1.75935300	3.23871300	-0.78198800
C	3.11130000	3.08420300	-1.07273100
C	3.93924300	2.42153400	-0.17651200
C	3.42066700	1.92755400	1.01699700
C	2.07563600	2.09075300	1.30816900
H	1.66929800	1.69768500	2.23532400
H	4.06890900	1.40802200	1.71409500
H	4.99173500	2.29025500	-0.40278900
H	3.51503000	3.48129100	-1.99786700
H	1.11213000	3.76265700	-1.48102800
H	-0.43570300	3.20445000	1.69271900
H	-0.73586900	3.48984700	-0.03440000
H	-0.05786100	1.32343900	-1.19813500
H	-0.69247000	-0.90300100	0.70786100
H	1.23250900	-2.16008900	1.08117600
H	-0.23742700	-3.63342700	-1.80049400
H	-0.49742600	-3.29375100	-0.06902000
H	-0.35492200	-1.21578400	-2.31827500

Man-oxo/conf_5.out

Charge=+1, Multiplicity=+1

C	-0.39099	-1.12613	-0.19400
C	-0.61141	0.38416	-0.27828
C	-0.35034	1.10063	1.04485
H	-1.64645	0.58592	-0.56469
O	0.27958	0.91913	-1.22211
O	-1.10350	0.44571	2.11446
H	0.70531	1.01622	1.32255
C	-0.77917	2.55155	0.89976
C	-0.92291	-0.80291	2.21694
C	-0.03855	-1.52750	1.31906
O	-0.10740	-2.87814	1.56752
H	0.97810	-1.10519	1.43784
H	-1.47716	-1.29606	3.01551
C	1.16456	-3.56855	1.58487
H	0.49704	-1.37652	-0.77589

O	-1.39766	-1.93997	-0.67597
C	-2.64018	-1.95788	-0.00219
O	-0.71847	2.89002	-0.47566
H	-0.15338	3.21594	1.50258
H	-1.82539	2.66061	1.19479
C	0.38701	2.34646	-1.14763
C	1.71099	2.74339	-0.52881
H	0.30423	2.71404	-2.17243
C	2.73944	1.81950	-0.35622
C	3.94740	2.21957	0.20579
C	4.13588	3.54081	0.59166
C	3.11520	4.46822	0.40609
C	1.90870	4.07297	-0.15541
H	2.59838	0.79402	-0.67991
H	4.74878	1.49894	0.33224
H	5.07946	3.85198	1.02639
H	3.26198	5.50302	0.69564
H	1.11322	4.79651	-0.30595
C	2.00263	-3.25274	0.38500
H	0.88718	-4.62148	1.62824
H	1.69282	-3.30669	2.50910
C	1.66576	-3.77291	-0.86661
C	2.38848	-3.40755	-1.99311
C	3.45585	-2.51946	-1.88133
C	3.80803	-2.00912	-0.63832
C	3.08327	-2.37654	0.49138
H	0.82658	-4.45578	-0.95562
H	2.12324	-3.81700	-2.96162
H	4.02039	-2.23748	-2.76336
H	4.65460	-1.33716	-0.54575
H	3.37498	-1.99587	1.46745
C	-3.53399	-0.77777	-0.27138
H	-2.46919	-2.06594	1.08780
H	-3.11445	-2.88956	-0.32100
C	-4.16484	-0.10916	0.77386
C	-4.99181	0.98041	0.51802
C	-5.18628	1.40959	-0.78833
C	-4.56660	0.73776	-1.83894

C	-3.74924	-0.35390	-1.58394
H	-4.02432	-0.44554	1.79819
H	-5.48429	1.49096	1.33861
H	-5.82614	2.26132	-0.99092
H	-4.72601	1.06451	-2.86069
H	-3.26130	-0.87502	-2.40173

Man-6B/conf_1.out

Charge=+1, Multiplicity=+1

C	-0.41539	3.08982	0.43365
O	-0.93658	2.59529	1.65423
C	-2.21613	2.15484	1.23612
C	-2.05325	0.88214	0.32142
O	-2.61107	1.05507	-0.94665
C	0.08054	1.90354	-0.39135
O	-0.25391	-0.39026	-0.78997
O	1.52290	1.74800	-0.18532
H	0.39809	3.78275	0.64941
C	-1.64846	3.77164	-0.20519
O	-2.74348	3.22221	0.51496
H	-2.82729	1.97265	2.11885
H	-1.74636	3.55185	-1.27187
H	-1.63691	4.85063	-0.04482
C	-4.01698	0.81269	-1.01019
C	-4.35851	-0.60409	-0.64089
H	-4.28416	1.02387	-2.04781
H	-4.55254	1.52544	-0.37295
C	-3.76533	-1.66362	-1.33057
C	-4.05065	-2.97612	-0.98237
C	-4.93570	-3.24576	0.05893
C	-5.53276	-2.19795	0.74725
C	-5.24038	-0.88212	0.39977
H	-3.07684	-1.44803	-2.14226
H	-3.59467	-3.79444	-1.53028
H	-5.16369	-4.27192	0.32650
H	-6.22609	-2.40275	1.55589
H	-5.70811	-0.06303	0.93937
C	-0.21485	-1.70438	-0.21261

C	1.11661	-1.97431	0.42449
H	-1.03842	-1.82110	0.49958
H	-0.39756	-2.38661	-1.04381
C	1.34870	-1.68926	1.77136
C	2.61099	-1.86858	2.32486
C	3.65126	-2.35503	1.54017
C	3.42402	-2.66928	0.20456
C	2.16534	-2.47438	-0.34873
H	0.53147	-1.35030	2.40190
H	2.77700	-1.65040	3.37444
H	4.63155	-2.51368	1.97687
H	4.22538	-3.07852	-0.40135
H	1.98517	-2.72868	-1.38985
C	2.17299	1.02941	-1.01106
C	-0.55959	0.62812	0.11911
H	-0.08619	2.02991	-1.46385
H	-0.11366	0.42541	1.09904
C	3.52332	0.68892	-0.78122
C	4.16578	0.98451	0.43739
C	5.49510	0.65458	0.59401
C	6.18586	0.03389	-0.44953
C	5.55406	-0.27267	-1.65419
C	4.22150	0.04542	-1.82140
H	3.70958	-0.18968	-2.74925
H	7.23320	-0.21798	-0.31843
H	6.10544	-0.75789	-2.45086
H	3.60919	1.46113	1.23576
H	6.00429	0.87358	1.52523
H	1.66594	0.71017	-1.92190
H	-2.49519	0.01345	0.82567

Man-6B/conf_2.out

Charge=+1, Multiplicity=+1

C	1.01893	2.99984	0.72015
O	0.31071	2.55902	1.86539
C	-0.98889	2.40142	1.32470
C	-1.00290	1.16550	0.33644
O	-1.31982	1.54026	-0.97471

C	1.29111	1.78637	-0.17236
O	0.65991	-0.45701	-0.56487
O	2.66991	1.33169	-0.03133
H	1.94909	3.47303	1.03552
C	0.01388	3.98230	0.08082
O	-1.23393	3.59224	0.64119
H	-1.69558	2.29557	2.14624
H	-0.01805	3.90693	-1.00931
H	0.22019	5.01167	0.37871
C	-2.70916	1.75397	-1.22032
C	-3.50029	0.47874	-1.30226
H	-2.73894	2.27852	-2.17847
H	-3.12841	2.42719	-0.46610
C	-3.16465	-0.49389	-2.24534
C	-3.93251	-1.64229	-2.37566
C	-5.04818	-1.83172	-1.56332
C	-5.38319	-0.87364	-0.61540
C	-4.60779	0.27434	-0.48423
H	-2.30044	-0.33876	-2.88433
H	-3.67479	-2.38555	-3.12331
H	-5.65816	-2.72182	-1.67718
H	-6.25164	-1.01524	0.01918
H	-4.87879	1.02820	0.24979
C	0.75679	-1.73148	0.10292
C	-0.43334	-2.04679	0.95578
H	0.87063	-2.45134	-0.70929
H	1.66958	-1.74355	0.71393
C	-0.35866	-1.91962	2.34225
C	-1.47901	-2.14622	3.13359
C	-2.68248	-2.50585	2.53903
C	-2.76152	-2.64731	1.15684
C	-1.64239	-2.41997	0.36896
H	0.58470	-1.64900	2.80975
H	-1.41038	-2.04805	4.21156
H	-3.55826	-2.68528	3.15341
H	-3.69682	-2.93013	0.68894
H	-1.71398	-2.51752	-0.70905
C	3.07769	0.51318	-0.92155

C	0.42710	0.62514	0.29267
H	1.12106	1.99382	-1.23146
H	0.72526	0.37618	1.31497
C	4.24770	-0.25605	-0.73107
C	4.96163	-0.23164	0.48461
C	6.09079	-1.01042	0.61776
C	6.51125	-1.81512	-0.44579
C	5.80851	-1.84883	-1.64901
C	4.67575	-1.07217	-1.79624
H	4.11358	-1.08575	-2.72480
H	7.40074	-2.42580	-0.33100
H	6.14899	-2.47969	-2.46145
H	4.61739	0.39791	1.29722
H	6.65277	-1.00217	1.54434
H	2.51818	0.44708	-1.85383
H	-1.67914	0.38706	0.70681

Man-4B/conf_1.out

Charge=+1, Multiplicity=+1

C	1.21867500	1.44158600	0.93853000
O	0.18239400	1.11917200	1.87026600
C	0.76059000	0.13866200	2.71862800
C	0.81784100	-1.19774900	1.95632700
C	2.11788200	-0.97057000	1.11912600
O	1.95512300	-1.38064000	-0.20874900
C	3.17316800	-1.74501400	-0.86692800
C	4.09728400	-0.57798000	-1.06052000
C	3.79340200	0.40451900	-2.00431200
C	4.59409700	1.53168400	-2.13370100
C	5.71516600	1.68464800	-1.32245600
C	6.03955200	0.70037800	-0.39655000
C	5.23368900	-0.42621100	-0.26893400
H	5.49181000	-1.19531300	0.45388700
H	6.92274300	0.80704800	0.22387900
H	6.34447500	2.56189400	-1.42571800
H	4.35762200	2.28436500	-2.87860200
H	2.93251100	0.27252400	-2.65452900
H	2.85623100	-2.16105300	-1.82543000

H	3.66957800	-2.53860000	-0.29462200
C	2.36937500	0.52607200	1.41239500
O	2.10556300	0.51934800	2.81745100
H	3.36869000	0.89928100	1.20667800
H	2.94668600	-1.51558600	1.58586400
O	-0.34230500	-1.33998700	1.18441300
C	-0.64464300	-2.67329200	0.76980900
C	-1.84224400	-2.57513700	-0.12459300
C	-3.11416100	-2.91028300	0.33263800
C	-4.22460300	-2.75143100	-0.49207300
C	-4.07130400	-2.24080300	-1.77567300
C	-2.80313300	-1.90416000	-2.24116300
C	-1.69536600	-2.07739200	-1.42139500
H	-0.69983000	-1.84222600	-1.78882700
H	-2.67873200	-1.52525400	-3.25014400
H	-4.93556700	-2.11994200	-2.41983100
H	-5.20883000	-3.03310400	-0.13274100
H	-3.23592900	-3.30675100	1.33637800
H	0.21224700	-3.09498500	0.23561500
H	-0.85822800	-3.29165500	1.65040600
H	0.92556000	-2.03910700	2.64937900
H	0.26204700	0.14775200	3.68425800
C	0.70384300	1.22780900	-0.47306800
O	-0.60665200	1.85886800	-0.57827800
C	-1.59073700	1.18063400	-0.13039300
C	-2.90014400	1.70937900	-0.11532300
C	-3.92659100	0.83692900	0.29212200
C	-5.23340800	1.28713500	0.31359200
C	-5.51270200	2.59853300	-0.06224800
C	-4.49625200	3.47075400	-0.46717000
C	-3.18989300	3.03418900	-0.49814700
H	-2.38662900	3.69336300	-0.80677400
H	-4.73927900	4.48732900	-0.75347100
H	-6.53807500	2.95304700	-0.04076100
H	-6.03429000	0.62508400	0.62092000
H	-3.68298800	-0.18352200	0.57123200
H	-1.39309900	0.16726400	0.22519900
H	1.32719000	1.73084000	-1.20929600

H	0.59348500	0.17430900	-0.71726600
H	1.49131400	2.49158500	1.08235200

Man-4B/conf_2.out

Charge=+1, Multiplicity=+1

C	2.21439	-0.61184	-0.73145
C	3.26096	-1.52508	-0.06944
C	2.73935	-2.12550	1.24384
O	1.66198	-2.93520	0.78352
H	3.53214	-2.76343	1.65924
C	2.28163	-1.26944	2.39928
C	1.81926	-2.94141	-0.64531
C	1.19770	-1.66979	-1.21854
O	-0.12069	-1.48937	-0.79079
H	1.25357	-1.71356	-2.31394
O	2.71687	0.09570	-1.83119
H	1.74806	0.07941	-0.02383
O	3.17931	-2.71861	-0.86178
H	4.28358	-1.15432	-0.04366
H	1.48652	-3.89407	-1.04867
O	1.14803	-0.42291	2.09366
H	3.06361	-0.58025	2.71705
H	2.00255	-1.90749	3.24139
C	-0.02450	-0.93705	2.04712
C	-1.15225	-0.12626	1.84537
H	-0.12215	-2.00862	2.21839
C	-1.03572	1.25482	1.57236
C	-2.18000	2.00492	1.43777
C	-3.43499	1.40018	1.58297
C	-3.55860	0.04052	1.85399
C	-2.42048	-0.73162	1.96559
H	-0.05725	1.71168	1.46745
H	-2.10957	3.06369	1.21835
H	-4.33044	2.00391	1.47959
H	-4.53805	-0.41095	1.94731
H	-2.49218	-1.79730	2.15541
C	-0.79839	-0.48288	-1.52408
C	-2.28282	-0.61828	-1.36019

H	-0.53526	-0.57838	-2.58782
H	-0.46759	0.51389	-1.20111
C	-2.87014	-1.84141	-1.04328
C	-4.25382	-1.95286	-0.95335
C	-5.06231	-0.84623	-1.18492
C	-4.48006	0.37568	-1.50678
C	-3.09721	0.48836	-1.58834
H	-2.23695	-2.70532	-0.87468
H	-4.70214	-2.91178	-0.71467
H	-6.14171	-0.93712	-1.12765
H	-5.10509	1.24131	-1.70036
H	-2.64656	1.44561	-1.83655
C	3.34987	1.31537	-1.47370
C	2.39337	2.31125	-0.86834
H	3.76943	1.70013	-2.40565
H	4.18739	1.13462	-0.78537
C	2.61703	2.84911	0.39660
C	1.74197	3.78936	0.93507
C	0.62709	4.19235	0.21021
C	0.38719	3.64897	-1.04991
C	1.26520	2.71611	-1.58498
H	3.49770	2.54886	0.95840
H	1.93983	4.21643	1.91264
H	-0.04242	4.94341	0.61704
H	-0.47294	3.97390	-1.62657
H	1.08962	2.30489	-2.57458

Man-4B/conf_3.out

Charge=+1, Multiplicity=+1

C	2.42660	-0.19855	-0.62390
C	3.53982	-1.06324	-0.03541
C	3.06577	-1.82691	1.20781
O	2.07942	-2.69656	0.65498
H	3.91296	-2.41958	1.57970
C	2.51865	-1.12105	2.42496
C	2.25483	-2.55441	-0.76341
C	1.50522	-1.28809	-1.22040
O	0.19746	-1.14163	-0.74613

H	1.53338	-1.24652	-2.31797
O	2.99474	0.66862	-1.56473
H	1.87828	0.37220	0.13004
O	3.58598	-2.18064	-0.93503
H	4.51261	-0.58282	0.03662
H	2.03218	-3.49724	-1.25764
O	1.30727	-0.36871	2.17686
H	3.22851	-0.38764	2.80652
H	2.30203	-1.85136	3.20855
C	0.19404	-0.99515	2.09160
C	-1.01616	-0.30463	1.92872
H	0.21014	-2.07923	2.20401
C	-1.06001	1.09298	1.73450
C	-2.28440	1.71284	1.64444
C	-3.45966	0.95930	1.73874
C	-3.42517	-0.42183	1.91446
C	-2.20555	-1.05986	1.99866
H	-0.14186	1.66426	1.64858
H	-2.33564	2.78373	1.49069
H	-4.41922	1.46054	1.66663
H	-4.34577	-0.98924	1.96586
H	-2.15385	-2.13588	2.13009
C	-0.72129	-2.06078	-1.31614
C	-2.11393	-1.50313	-1.26402
H	-0.67202	-3.02624	-0.79426
H	-0.44188	-2.24341	-2.36476
C	-2.33798	-0.14221	-1.45864
C	-3.63437	0.35392	-1.49967
C	-4.71956	-0.50361	-1.34751
C	-4.50091	-1.86134	-1.14315
C	-3.20321	-2.35716	-1.09732
H	-1.49060	0.52547	-1.56568
H	-3.80014	1.41445	-1.66089
H	-5.73187	-0.11645	-1.39418
H	-5.34157	-2.53677	-1.02508
H	-3.03727	-3.42016	-0.94426
C	2.06392	1.54536	-2.16959
C	1.40367	2.50348	-1.20897

H	1.29237	0.98106	-2.71237
H	2.64341	2.09791	-2.91390
C	0.10816	2.95291	-1.45428
C	-0.48912	3.88741	-0.61578
C	0.19617	4.36448	0.49589
C	1.48658	3.91111	0.75500
C	2.08929	2.99355	-0.09775
H	-0.43056	2.58733	-2.32446
H	-1.48810	4.24938	-0.83729
H	-0.26315	5.09978	1.14835
H	2.03332	4.29281	1.61106
H	3.10705	2.66259	0.08526

Man-4B/conf_4.out

Charge=+1, Multiplicity=+1

C	1.68783	-0.63086	-1.07242
C	3.20738	-0.72510	-0.98132
C	3.74553	0.03903	0.23901
O	3.23304	-0.73388	1.32110
H	4.84221	-0.02705	0.22564
C	3.44588	1.50266	0.46236
C	2.67016	-1.88790	0.68177
C	1.27915	-1.50341	0.13921
O	0.49004	-0.77715	1.04346
H	0.75980	-2.40006	-0.21524
O	1.28610	-1.13846	-2.31243
H	1.30815	0.38547	-0.94384
O	3.41227	-2.05536	-0.48551
H	3.74220	-0.57337	-1.91578
H	2.75337	-2.74775	1.34216
O	2.03423	1.80566	0.61187
H	3.76598	2.10794	-0.38511
H	3.95397	1.84852	1.36574
C	1.44403	1.54417	1.71256
C	0.09224	1.90789	1.90745
H	2.03073	1.10343	2.51841
C	-0.43560	1.76788	3.20504
C	-1.74672	2.12727	3.45200

C	-2.53064	2.61327	2.40670
C	-2.01588	2.73818	1.11387
C	-0.70754	2.38841	0.85272
H	0.19316	1.39096	4.00597
H	-2.16185	2.03396	4.44855
H	-3.56019	2.89626	2.60035
H	-2.64221	3.10456	0.30972
H	-0.30596	2.46753	-0.15209
C	-0.33133	-1.58779	1.89026
C	-1.50977	-2.14407	1.14758
H	-0.66256	-0.91908	2.68851
H	0.26422	-2.38908	2.34354
C	-2.57312	-1.30468	0.81378
C	-3.65226	-1.79151	0.08986
C	-3.67497	-3.12287	-0.31707
C	-2.62201	-3.96648	0.01345
C	-1.54593	-3.47825	0.74761
H	-2.54968	-0.26407	1.12439
H	-4.48378	-1.13763	-0.15286
H	-4.51895	-3.50377	-0.88181
H	-2.64062	-5.00652	-0.29316
H	-0.73284	-4.14567	1.02032
C	-0.10883	-1.03835	-2.53964
C	-0.61656	0.38115	-2.60362
H	-0.67179	-1.59548	-1.77772
H	-0.27235	-1.54005	-3.49703
C	-1.93182	0.66203	-2.24064
C	-2.43622	1.95291	-2.35226
C	-1.61902	2.98667	-2.79650
C	-0.30003	2.71564	-3.14937
C	0.19347	1.41863	-3.06503
H	-2.56701	-0.14078	-1.87958
H	-3.47319	2.15003	-2.09810
H	-2.01031	3.99463	-2.88618
H	0.33786	3.51373	-3.51484
H	1.21168	1.19969	-3.37200

Man-4B/conf_5.out

Charge=+1, Multiplicity=+1

C	-1.68143	-0.60244	0.94250
C	-3.19333	-0.75461	0.73069
C	-3.63499	-0.17814	-0.62551
O	-3.01000	-1.06945	-1.54377
H	-4.72684	-0.27711	-0.70093
C	-3.34536	1.25156	-1.01755
C	-2.47361	-2.10548	-0.70789
C	-1.15098	-1.59662	-0.10504
O	-0.30662	-0.95881	-1.02654
H	-0.63223	-2.40569	0.41926
O	-1.27549	-1.00740	2.21956
H	-1.31095	0.40343	0.73334
O	-3.31523	-2.14524	0.40316
H	-3.82926	-0.50377	1.57699
H	-2.47184	-3.04804	-1.24941
O	-1.93257	1.57993	-1.07792
H	-3.75667	1.95454	-0.29376
H	-3.77283	1.45676	-2.00193
C	-1.23445	1.19193	-2.07376
C	0.11659	1.58856	-2.20097
H	-1.72994	0.61782	-2.85629
C	0.79449	2.25377	-1.16180
C	2.11152	2.61729	-1.34933
C	2.75458	2.32605	-2.55518
C	2.09024	1.66128	-3.58431
C	0.77198	1.28530	-3.40888
H	0.29341	2.46230	-0.22252
H	2.64620	3.12475	-0.55546
H	3.79029	2.61931	-2.69179
H	2.60348	1.44101	-4.51283
H	0.23635	0.76738	-4.19864
C	0.65611	-1.82197	-1.63755
C	1.73692	-2.22154	-0.67638
H	1.07128	-1.23873	-2.46373
H	0.16320	-2.70486	-2.06205
C	2.58697	-1.24795	-0.15025
C	3.57641	-1.59771	0.75652

C	3.72485	-2.92533	1.15008
C	2.88305	-3.89958	0.63062
C	1.89200	-3.54681	-0.28010
H	2.46338	-0.21169	-0.45081
H	4.24245	-0.83941	1.15551
H	4.49989	-3.19843	1.85789
H	2.99592	-4.93476	0.93336
H	1.23747	-4.31191	-0.68867
C	-1.25997	0.05370	3.16000
C	-0.23775	1.11045	2.82503
H	-1.02526	-0.41910	4.11646
H	-2.25568	0.51047	3.25589
C	-0.57066	2.46276	2.84241
C	0.39155	3.43426	2.57898
C	1.69673	3.05475	2.28932
C	2.03068	1.70323	2.24809
C	1.07025	0.73644	2.51161
H	-1.58685	2.76267	3.08455
H	0.12430	4.48513	2.61453
H	2.45448	3.81139	2.11165
H	3.04984	1.40189	2.02809
H	1.33154	-0.31640	2.47726

Man-4B/conf_6.out

Charge=+1, Multiplicity=+1

C	0.66619	0.31291	2.19732
C	0.78450	-0.92586	3.08012
C	-0.39650	-1.89035	2.87609
O	-0.17041	-2.37091	1.55536
H	-0.28707	-2.71525	3.59327
C	-1.83131	-1.43203	3.01845
C	1.09924	-1.80542	1.19759
C	0.87969	-0.33097	0.80016
O	-0.21989	-0.10585	-0.04394
H	1.79917	0.06253	0.36163
O	1.66473	1.21471	2.58055
H	-0.31742	0.79458	2.24841
O	1.78897	-1.69147	2.40462

H	1.06929	-0.73521	4.11207
H	1.60600	-2.45448	0.48938
O	-2.25947	-0.47559	2.01044
H	-1.99874	-0.92248	3.96681
H	-2.50244	-2.29115	2.95011
C	-2.45440	-0.87945	0.81415
C	-3.02403	-0.01952	-0.15707
H	-2.26380	-1.92794	0.58965
C	-3.20174	1.35528	0.07982
C	-3.75151	2.14409	-0.90836
C	-4.13183	1.57302	-2.12641
C	-3.96340	0.21158	-2.36616
C	-3.40260	-0.58693	-1.38601
H	-2.89883	1.77987	1.02957
H	-3.89254	3.20588	-0.74254
H	-4.56709	2.20100	-2.89671
H	-4.26649	-0.21772	-3.31377
H	-3.26158	-1.64957	-1.55666
C	0.11672	-0.02096	-1.43571
C	0.82090	-1.24390	-1.94240
H	0.72924	0.87295	-1.58823
H	-0.83808	0.13252	-1.94458
C	2.18545	-1.20182	-2.22030
C	2.86165	-2.35092	-2.61673
C	2.17523	-3.55284	-2.73904
C	0.80928	-3.60270	-2.47442
C	0.13905	-2.45320	-2.08055
H	2.71980	-0.26081	-2.12273
H	3.92425	-2.30789	-2.82978
H	2.70093	-4.45011	-3.04683
H	0.27049	-4.53824	-2.57956
H	-0.92623	-2.49593	-1.87240
C	1.45268	2.54973	2.15081
C	1.65949	2.77582	0.67504
H	2.16945	3.14214	2.72459
H	0.44237	2.87600	2.43763
C	2.88423	2.46207	0.08166
C	3.09304	2.69912	-1.27022

C	2.08238	3.26312	-2.04607
C	0.86216	3.58018	-1.46298
C	0.65280	3.33040	-0.10929
H	3.67649	2.03479	0.68956
H	4.05284	2.46327	-1.71822
H	2.25154	3.46160	-3.09897
H	0.07423	4.02685	-2.06049
H	-0.30133	3.58289	0.34560

Man-4B/conf_7.out

Charge=+1, Multiplicity=+1

C	-1.65130	-1.28785	-0.79293
C	-2.28417	-2.44961	-0.01568
C	-1.24013	-3.24975	0.77773
O	-0.81980	-2.30386	1.75663
H	-1.76356	-4.08071	1.27082
C	-0.03910	-3.88687	0.11750
C	-1.72483	-1.20539	1.58504
C	-1.26928	-0.35496	0.39913
O	0.09376	-0.03352	0.48430
H	-1.87079	0.55732	0.37526
O	-2.44844	-0.74893	-1.81078
H	-0.74349	-1.59714	-1.31324
O	-2.90122	-1.78433	1.10070
H	-3.01011	-3.05141	-0.55922
H	-1.87750	-0.70215	2.53624
O	0.91631	-2.95327	-0.44915
H	-0.33581	-4.51679	-0.72063
H	0.49916	-4.49616	0.84706
C	1.66473	-2.27154	0.33747
C	2.73036	-1.50418	-0.16505
H	1.49013	-2.35476	1.40893
C	2.98951	-1.39394	-1.54811
C	4.07912	-0.66795	-1.96961
C	4.91772	-0.05801	-1.02792
C	4.67509	-0.16768	0.33756
C	3.57384	-0.87677	0.77381
H	2.33627	-1.88587	-2.25969

H	4.29428	-0.57015	-3.02729
H	5.77588	0.50993	-1.37152
H	5.32927	0.31917	1.04955
H	3.35466	-0.96002	1.83282
C	0.46325	1.00703	-0.41019
C	1.68708	1.71985	0.08384
H	-0.37045	1.71888	-0.48988
H	0.63415	0.60197	-1.41750
C	1.95314	1.83105	1.44796
C	3.05452	2.55412	1.89191
C	3.89691	3.17773	0.97813
C	3.63270	3.07334	-0.38337
C	2.53562	2.34357	-0.82694
H	1.28828	1.35197	2.15831
H	3.24805	2.64187	2.95602
H	4.74885	3.75249	1.32533
H	4.27961	3.56586	-1.10194
H	2.33510	2.26396	-1.89186
C	-3.78157	-0.33329	-1.50651
C	-3.85045	1.01219	-0.83663
H	-4.30211	-1.08177	-0.90067
H	-4.27115	-0.29613	-2.48264
C	-4.49629	1.16999	0.38661
C	-4.52098	2.41116	1.01582
C	-3.89393	3.50192	0.42663
C	-3.25567	3.35362	-0.80250
C	-3.23922	2.11687	-1.43259
H	-4.96953	0.31257	0.85578
H	-5.02891	2.52485	1.96768
H	-3.91072	4.46938	0.91680
H	-2.78137	4.20836	-1.27349
H	-2.74298	1.99789	-2.39129

Man-4B/conf_8.out

Charge=+1, Multiplicity=+1

C	0.14889	-1.83272	0.91827
C	0.15825	-3.15691	0.14602
C	-1.08082	-3.32409	-0.74997

O	-0.87440	-2.32158	-1.74228
H	-1.01871	-4.31697	-1.21651
C	-2.49000	-3.24363	-0.20773
C	0.43321	-1.81274	-1.44846
C	0.33587	-0.84085	-0.26991
O	-0.74026	0.05912	-0.42552
H	1.27513	-0.29138	-0.17675
O	1.06929	-1.75011	1.96973
H	-0.81473	-1.64722	1.39747
O	1.11455	-2.89928	-0.89507
H	0.43276	-4.04295	0.71537
H	0.90514	-1.45644	-2.36025
O	-2.89323	-1.94009	0.29383
H	-2.62714	-3.91372	0.64033
H	-3.19973	-3.51410	-0.99259
C	-3.03776	-0.96944	-0.52814
C	-3.64029	0.24104	-0.11088
H	-2.76966	-1.13344	-1.56980
C	-4.05737	0.44816	1.21757
C	-4.62681	1.65575	1.56398
C	-4.78122	2.65552	0.59925
C	-4.37130	2.45660	-0.71775
C	-3.80062	1.25035	-1.07777
H	-3.92951	-0.33898	1.95172
H	-4.95587	1.83092	2.58162
H	-5.22901	3.60268	0.88136
H	-4.49742	3.24264	-1.45276
H	-3.46274	1.07939	-2.09479
C	-0.62308	1.18497	0.43631
C	0.42240	2.17664	-0.00132
H	-0.42279	0.85186	1.46314
H	-1.60628	1.66383	0.43061
C	0.92108	3.08584	0.92942
C	1.83351	4.05815	0.54285
C	2.27428	4.11501	-0.77508
C	1.79069	3.20116	-1.70282
C	0.85999	2.24178	-1.32039
H	0.59464	3.03318	1.96473

H	2.21005	4.76607	1.27371
H	2.99458	4.86791	-1.07622
H	2.13483	3.23753	-2.73093
H	0.47359	1.53741	-2.04967
C	2.43667	-2.10372	1.73641
C	3.21866	-1.06600	0.97803
H	2.51140	-3.07200	1.23135
H	2.84221	-2.22335	2.74363
C	3.30681	0.23772	1.46851
C	3.99851	1.20970	0.76034
C	4.61756	0.88776	-0.44337
C	4.55061	-0.41200	-0.92944
C	3.85160	-1.38433	-0.22123
H	2.81867	0.49019	2.40528
H	4.05445	2.22159	1.14445
H	5.15655	1.64943	-0.99645
H	5.04212	-0.67096	-1.86142
H	3.78388	-2.39599	-0.60845
