# Supplementary material for: Mechanistic insight into benzylidene-directed glycosylation reactions using cryogenic infrared spectroscopy
Source: Nat Synth. 2024 Jul 26;3(11):1377–84. doi: 10.1038/s44160-024-00619-0 (PMC11549046; doi:10.1038/s44160-024-00619-0)
Supplement: Supplementary file 3 — SI_coordinated_solvents.pdf. [file 44160_2024_619_MOESM3_ESM.pdf]

Glc-4B_Dioxane

Charge=+1, Multiplicity=+1

C	1.97345200	0.86231200	1.91971000
O	0.69150100	0.81726700	2.54907000
C	2.15907800	-0.59591800	1.48389500
C	1.13244500	-1.11116600	0.44770300
C	-0.17723400	-1.00512900	1.24725300
C	0.38125900	-0.58940900	2.61285800
H	2.74460000	1.06800100	2.67332300
C	2.02857800	2.03405700	0.95864100
O	1.63358600	-1.21567700	2.66556800
H	3.19202900	-0.89024200	1.32033100
H	-0.21370200	-0.80248200	3.49713500
O	-0.80788900	-2.25733900	1.28420700
H	-0.83840500	-0.24264800	0.82393400
H	1.33809100	-2.18042100	0.31425600
O	1.03945200	-0.47050600	-0.79137300
O	0.83987000	2.09645300	0.14624300
H	2.86317700	1.94592500	0.26540900
H	2.10111600	2.97112100	1.51601000
C	-0.23896300	2.48561300	0.70275000
C	-1.47602600	2.34192400	0.04473100
H	-0.18091000	2.95844300	1.68295500
C	-2.62107500	2.88520600	0.66046700
C	-1.57307300	1.65454000	-1.18427700
C	-2.80391900	1.53655500	-1.78798800
C	-3.93488000	2.08487400	-1.17632500
C	-3.84833000	2.75606900	0.04304400
H	-2.52917600	3.40317700	1.60949800
H	-4.90068000	1.98097600	-1.65838600
H	-4.73856300	3.16904700	0.50186400
H	-0.68352800	1.20672300	-1.61202700
H	-2.90289500	1.00183800	-2.72372900
C	-2.19614200	-2.19002500	1.54495100
C	-3.01827300	-1.82557100	0.33292700
H	-2.41029600	-1.49796800	2.37113400
H	-2.47546400	-3.19125600	1.88582300
C	-4.27855200	-1.25240800	0.49298700

C	-5.09899700	-1.03029200	-0.60718200
C	-4.65925100	-1.36450000	-1.88392700
C	-3.39077300	-1.90906200	-2.05285300
C	-2.57478500	-2.13904300	-0.95099500
H	-4.63162500	-0.99601500	1.48818400
H	-6.08569400	-0.60118400	-0.46552400
H	-5.30256400	-1.20184700	-2.74252100
H	-3.03940500	-2.16981300	-3.04609300
H	-1.59245000	-2.58075300	-1.07861300
C	1.90338900	-0.99246500	-1.79907100
C	3.34120000	-0.60746500	-1.59322300
H	1.52579800	-0.57596100	-2.73538900
H	1.79777600	-2.08407800	-1.84074700
C	4.26790000	-1.52189400	-1.09676800
C	5.58161400	-1.13548100	-0.85023000
C	5.98080900	0.17138300	-1.10448900
C	5.06730200	1.08770400	-1.61921200
C	3.75648600	0.69783600	-1.86336300
H	3.96126300	-2.54595500	-0.90249500
H	6.29439500	-1.85692700	-0.46531600
H	7.00600600	0.47264000	-0.91816200
H	5.38346300	2.10131500	-1.84273900
H	3.04604900	1.40728200	-2.27992400

Glc-4B_Toluene

Charge=+1, Multiplicity=+1

C	-0.29018000	-3.12956500	0.86062300
O	-1.66650600	-3.08991200	1.23731300
C	-0.37063800	-2.97018200	-0.67038800
C	-0.81836800	-1.57122100	-1.10406700
C	-2.22360500	-1.50618300	-0.47209500
C	-2.36378500	-2.96474000	-0.00113300
H	0.12370800	-4.11535000	1.09587900
C	0.41123300	-2.06323900	1.67482900
O	-1.57515500	-3.69615900	-0.91001000
H	0.45987000	-3.39788400	-1.22651100
H	-3.36584800	-3.37706400	0.08541600
O	-3.17275000	-1.11513600	-1.42610400

H	-2.22553200	-0.82718600	0.38827000
H	-0.93912500	-1.58718900	-2.19429500
O	0.06763200	-0.54713000	-0.72531100
O	1.77721000	-1.93844300	1.19493500
H	0.48344300	-2.36555900	2.71993000
H	-0.08672200	-1.09914000	1.58543300
C	2.24368900	-0.77692400	0.97541100
C	3.55138800	-0.58436700	0.48643700
H	1.61537500	0.08743800	1.19041900
C	3.99004500	0.74740200	0.35855700
C	4.39597600	-1.65966100	0.14153500
C	5.66262200	-1.39134300	-0.32742100
C	6.09281700	-0.06520200	-0.45342500
C	5.26411700	1.00081600	-0.11208700
H	3.32573800	1.56214900	0.62818300
H	7.09226000	0.13442800	-0.82521500
H	5.61339900	2.02107400	-0.21692800
H	4.04291400	-2.67887200	0.24674700
H	6.32753900	-2.20251300	-0.59925000
C	-4.35227000	-0.54880800	-0.85192700
C	-4.06528800	0.76066600	-0.16817200
H	-4.81343600	-1.25374100	-0.14839800
H	-5.03605800	-0.41328300	-1.69162700
C	-4.09402500	0.86942900	1.22066900
C	-3.75855000	2.06772300	1.84572800
C	-3.38589200	3.16738800	1.08278500
C	-3.36034600	3.07062700	-0.30627000
C	-3.70114600	1.87598300	-0.92593500
H	-4.38512300	0.00937600	1.81795300
H	-3.79184200	2.14074000	2.92778300
H	-3.12323100	4.10244100	1.56622700
H	-3.07913000	3.93035000	-0.90517200
H	-3.68470100	1.80185900	-2.00964700
C	-0.23253600	0.67722600	-1.40376800
C	0.53562900	1.79761700	-0.77836700
H	-1.30714700	0.87515800	-1.32713900
H	0.02149500	0.57163100	-2.46517900
C	1.53946600	2.45807800	-1.48179900

C	2.22839700	3.51944600	-0.90118000
C	1.93079800	3.91356500	0.39816800
C	0.93148400	3.25390000	1.11145600
C	0.22961400	2.21039900	0.52130500
H	1.77824200	2.14468700	-2.49360600
H	2.99931600	4.03548700	-1.46370500
H	2.46630400	4.74026000	0.85248200
H	0.68384100	3.57226500	2.11854400
H	-0.58286200	1.72968400	1.06002200

Glc-4B_Et2O
Charge=+1, Multiplicity=+1

C	3.21491500	1.19816300	1.19180600
O	2.94148000	-0.17110200	1.49570100
C	3.52502000	1.12999800	-0.31005300
C	2.32176600	0.73064400	-1.16563300
C	2.07293200	-0.71681600	-0.67669400
C	3.26166900	-0.87112600	0.28159000
H	4.14075500	1.50314200	1.69402800
C	2.12821500	2.09801200	1.76800200
O	4.26838700	-0.09269200	-0.31007300
H	4.12566300	1.95296700	-0.68867800
H	3.61315800	-1.87365900	0.51143700
O	2.13604800	-1.59709800	-1.76708500
H	1.11636700	-0.80421400	-0.14801900
H	2.64532000	0.67629100	-2.21367400
O	1.25886100	1.61951000	-1.01003100
O	0.80721100	1.52124500	1.66567700
H	2.06971900	3.04669800	1.23966500
H	2.31889700	2.27798700	2.82757100
C	0.51183800	0.55613900	2.43963300
C	-0.74674000	-0.07592100	2.35858500
H	1.23497200	0.24750700	3.19302100
C	-1.02236800	-1.10620600	3.27917300
C	-1.71702200	0.33304900	1.42115000
C	-2.94729200	-0.28523700	1.41712100
C	-3.21890300	-1.29983800	2.33896300
C	-2.26244800	-1.71361600	3.26515400

H	-0.26396200	-1.40956400	3.99405300
H	-4.19268300	-1.77832900	2.33130000
H	-2.49158000	-2.50431000	3.96933300
H	-1.49053700	1.12839600	0.72082300
H	-3.70035800	0.01683300	0.69907800
C	1.59237300	-2.88254700	-1.49417600
C	0.09524400	-2.85880400	-1.33259000
H	2.05882000	-3.32386300	-0.60444400
H	1.87446600	-3.49571900	-2.35308600
C	-0.51269900	-3.37155200	-0.18937100
C	-1.89905100	-3.38067800	-0.06952800
C	-2.68865900	-2.85852100	-1.08588600
C	-2.08836000	-2.33182800	-2.22665800
C	-0.70544500	-2.33700800	-2.35066300
H	0.10300400	-3.77817300	0.60837300
H	-2.36035500	-3.79477300	0.82041900
H	-3.76982900	-2.86454100	-0.99402900
H	-2.70164500	-1.93210900	-3.02791400
H	-0.23623700	-1.94145300	-3.24659500
C	0.19798800	1.35513000	-1.91198400
C	-1.01185600	2.15058500	-1.52449300
H	-0.05384400	0.28781300	-1.89947200
H	0.52189300	1.60300100	-2.93417700
C	-0.89919100	3.36335500	-0.84779400
C	-2.03959900	4.06802700	-0.47728900
C	-3.30211100	3.57317600	-0.78790800
C	-3.41952600	2.36829100	-1.47367100
C	-2.27999800	1.65917500	-1.83467700
H	0.08457000	3.74475800	-0.59892800
H	-1.94211800	5.00811500	0.05582800
H	-4.19028100	4.12373300	-0.49672200
H	-4.40057200	1.97476900	-1.71913500
H	-2.37365700	0.70553100	-2.34622600

Glc-4B_$CHCl_3$

Charge=+1, Multiplicity=+1

C	-2.56803300	-2.44411700	1.28247800
O	-3.18985000	-1.17824400	1.50219300

C	-2.59773200	-2.55242100	-0.24866900
C	-1.72263200	-1.52862600	-0.97316800
C	-2.38236300	-0.20282500	-0.53605500
C	-3.60871700	-0.76435000	0.20373700
H	-3.21460600	-3.24134600	1.67086500
C	-1.27702300	-2.55769500	2.06581600
O	-3.88360600	-1.96859100	-0.47560300
H	-2.55343500	-3.56745000	-0.63607200
H	-4.49263800	-0.13507400	0.27265900
O	-2.71511600	0.54276000	-1.67849700
H	-1.73876000	0.36929600	0.14218800
H	-1.91798300	-1.64407900	-2.04679800
O	-0.34955500	-1.67216500	-0.72648000
O	-0.54205700	-1.31245900	2.06415400
H	-0.64498800	-3.36405800	1.68639600
H	-1.50267100	-2.73159200	3.11798100
C	0.67267500	-1.28960700	1.69553000
C	1.43257000	-0.10305600	1.77583800
H	1.14147200	-2.21572800	1.36533300
C	2.80622000	-0.19400600	1.48120300
C	0.86126600	1.12063600	2.18204100
C	1.66882600	2.22928900	2.29988900
C	3.03503100	2.13058200	2.01612600
C	3.60377100	0.92707500	1.60861800
H	3.22835200	-1.13940200	1.15576200
H	3.66202800	3.01064000	2.11367300
H	4.66208600	0.86973600	1.38526000
H	-0.19896700	1.17701500	2.39921200
H	1.24880100	3.17725000	2.61117200
C	-3.00320500	1.90785200	-1.40619700
C	-1.78094200	2.69123400	-1.00496700
H	-3.77702200	1.99845200	-0.63350700
H	-3.41941300	2.29963400	-2.33736800
C	-1.77188500	3.46063400	0.15562500
C	-0.65104800	4.21143800	0.49803700
C	0.47973700	4.18001200	-0.30840700
C	0.48379400	3.40349900	-1.46424600
C	-0.64278300	2.67049200	-1.81353000

H	-2.65238700	3.48077400	0.79180200
H	-0.66269400	4.81756800	1.39817000
H	1.35667600	4.75994300	-0.04023000
H	1.36193600	3.38277000	-2.10167600
H	-0.64850600	2.07928400	-2.72439400
C	0.42932400	-0.91109800	-1.64289300
C	1.87691200	-1.27540700	-1.51982100
H	0.29152100	0.15964000	-1.45336000
H	0.07090700	-1.11400700	-2.66224000
C	2.84983900	-0.30687300	-1.75870000
C	4.19938800	-0.62567200	-1.66843300
C	4.58982800	-1.91498200	-1.32023800
C	3.62273100	-2.88660300	-1.08109800
C	2.27193700	-2.57113100	-1.19014800
H	2.54776200	0.70780100	-2.00189900
H	4.94750700	0.13745300	-1.85531100
H	5.64272100	-2.16257500	-1.23885100
H	3.92098500	-3.89602600	-0.81801500
H	1.51786700	-3.33196800	-1.01710700

Glc-4B_DCM

Charge=+1, Multiplicity=+1

C	-2.47815100	-2.56059200	1.24496600
O	-3.17228100	-1.32966600	1.45616600
C	-2.45992200	-2.65692100	-0.28754900
C	-1.61818400	-1.58532200	-0.98278100
C	-2.35194100	-0.29709800	-0.55043400
C	-3.56992700	-0.92368800	0.15022000
H	-3.09286000	-3.39328900	1.60941300
C	-1.20774000	-2.61138900	2.06627800
O	-3.76699000	-2.13397900	-0.54711200
H	-2.35449500	-3.66533300	-0.67984200
H	-4.48414700	-0.33702600	0.19641500
O	-2.69361100	0.44485400	-1.69354600
H	-1.75485400	0.29847700	0.14914000
H	-1.78264200	-1.70064900	-2.06163100
O	-0.24684400	-1.67009600	-0.70556900
O	-0.52914200	-1.33498500	2.07068000

H	-0.53047900	-3.39188600	1.71372600
H	-1.45665700	-2.78611800	3.11281700
C	0.68933800	-1.26052500	1.72198300
C	1.38548600	-0.03410900	1.78649300
H	1.20997000	-2.16947700	1.42232300
C	2.76426900	-0.05666600	1.50433000
C	0.74455700	1.16542600	2.15914100
C	1.48893000	2.31941300	2.25566400
C	2.86045100	2.28969900	1.98166500
C	3.49786200	1.10996000	1.60765300
H	3.23946300	-0.98560500	1.20634400
H	3.43704300	3.20532200	2.06024600
H	4.55934500	1.10578100	1.39191000
H	-0.31839200	1.16798100	2.37052500
H	1.01489200	3.24938200	2.54201200
C	-3.05110600	1.79167100	-1.41358000
C	-1.87194000	2.63741400	-1.00880700
H	-3.82901800	1.83891300	-0.64127500
H	-3.48662000	2.16767200	-2.34269300
C	-1.92729200	3.44774800	0.12255000
C	-0.85260600	4.26519200	0.46043400
C	0.29702400	4.26077700	-0.31996700
C	0.36646500	3.44184700	-1.44400700
C	-0.71485000	2.64204300	-1.79009600
H	-2.82157000	3.44610100	0.73943000
H	-0.91277400	4.89932000	1.33911100
H	1.13914300	4.89100600	-0.05363200
H	1.26160700	3.43652800	-2.05772400
H	-0.66938200	2.01637800	-2.67630600
C	0.52719500	-0.86154900	-1.58275800
C	1.97743400	-1.22138500	-1.47102100
H	0.38296800	0.19824800	-1.34216100
H	0.17406400	-1.01706000	-2.61202800
C	2.94453800	-0.25148100	-1.72971900
C	4.29563400	-0.56135400	-1.63702000
C	4.69407600	-1.84310100	-1.26873400
C	3.73300500	-2.81580500	-1.01270000
C	2.37990500	-2.50923600	-1.12257200

H	2.63693700	0.75795400	-1.98743400
H	5.03904400	0.20333600	-1.83593900
H	5.74852000	-2.08267600	-1.18313200
H	4.03667600	-3.81869600	-0.73132300
H	1.63009700	-3.26943200	-0.93102600

Glc-4B_ACN

Charge=+1, Multiplicity=+1

C	-2.47388500	-2.56838600	1.23679300
O	-3.16967100	-1.33930400	1.46203600
C	-2.46388400	-2.65139400	-0.29618000
C	-1.62531400	-1.57432100	-0.98703400
C	-2.35704900	-0.28956500	-0.54039600
C	-3.57121000	-0.92082400	0.16213700
H	-3.08552900	-3.40453100	1.59765200
C	-1.19876000	-2.62519600	2.04990400
O	-3.77295600	-2.12529600	-0.54557200
H	-2.36024300	-3.65634300	-0.69714300
H	-4.48406000	-0.33267200	0.21702600
O	-2.70907600	0.46070500	-1.67618500
H	-1.75626700	0.30168000	0.15931100
H	-1.79499500	-1.68174200	-2.06598500
O	-0.25356400	-1.66196500	-0.71683700
O	-0.52272000	-1.34714300	2.06098000
H	-0.52289100	-3.40123700	1.68553500
H	-1.44035900	-2.81026500	3.09628400
C	0.69763300	-1.27121800	1.72037700
C	1.39282600	-0.04425500	1.79142000
H	1.21895200	-2.17885000	1.41899400
C	2.77044700	-0.06294000	1.50441900
C	0.75086600	1.15239800	2.17083200
C	1.49277900	2.30811000	2.26759200
C	2.86294400	2.28291500	1.98622900
C	3.50152100	1.10555200	1.60665100
H	3.24667700	-0.99049500	1.20392300
H	3.43715300	3.20011500	2.06352800
H	4.56201900	1.10462900	1.38604300
H	-0.31096400	1.15159600	2.38746800

H	1.01791600	3.23607500	2.55921700
C	-3.05973500	1.80711700	-1.38434200
C	-1.87254500	2.64919700	-0.99535400
H	-3.82439200	1.85184300	-0.59897600
H	-3.50985900	2.18808700	-2.30456200
C	-1.91077200	3.45915800	0.13711100
C	-0.82779200	4.27049600	0.46331000
C	0.31279800	4.26106900	-0.33054200
C	0.36458200	3.44334700	-1.45657600
C	-0.72495600	2.64928500	-1.79060100
H	-2.79637200	3.45874500	0.76628300
H	-0.87280600	4.90124800	1.34527500
H	1.16254100	4.88389900	-0.07115900
H	1.25323700	3.43209400	-2.07946500
H	-0.69163400	2.02275300	-2.67669200
C	0.52031400	-0.86516100	-1.60428400
C	1.97056900	-1.22294200	-1.48481800
H	0.37566700	0.19800300	-1.37989500
H	0.16890500	-1.03648900	-2.63178100
C	2.93821000	-0.25298700	-1.74139700
C	4.28924900	-0.56151000	-1.64078100
C	4.68673300	-1.84196900	-1.26678600
C	3.72496800	-2.81462200	-1.01220600
C	2.37220000	-2.50958100	-1.13002000
H	2.63114600	0.75565000	-2.00249400
H	5.03291500	0.20405400	-1.83545500
H	5.74089300	-2.07981300	-1.17248700
H	4.02746800	-3.81552400	-0.72255700
H	1.62234700	-3.26912100	-0.93602200

Glc-4B_DMSO

Charge=+1, Multiplicity=+1

C	-2.47028600	-2.57285100	1.23513900
O	-3.16823000	-1.34508700	1.46163700
C	-2.46016800	-2.65426800	-0.29790900
C	-1.62305400	-1.57535700	-0.98769900
C	-2.35649300	-0.29205600	-0.53957400
C	-3.56988500	-0.92563800	0.16220900

H	-3.08051100	-3.41034000	1.59521300
C	-1.19499400	-2.62826800	2.04806700
O	-3.77000500	-2.12964700	-0.54691700
H	-2.35506100	-3.65865400	-0.69984900
H	-4.48346000	-0.33863600	0.21747400
O	-2.70985200	0.45907400	-1.67448000
H	-1.75651200	0.29923200	0.16074700
H	-1.79263400	-1.68193400	-2.06675400
O	-0.25126100	-1.66161200	-0.71760700
O	-0.52131500	-1.34890900	2.05993300
H	-0.51781000	-3.40273800	1.68288500
H	-1.43606600	-2.81464200	3.09432400
C	0.69923700	-1.27102700	1.72054500
C	1.39228400	-0.04282600	1.79156900
H	1.22223300	-2.17792100	1.41993200
C	2.76998400	-0.05912600	1.50487400
C	0.74802800	1.15283500	2.17013200
C	1.48772900	2.31003500	2.26624100
C	2.85795100	2.28727800	1.98497000
C	3.49881100	1.11084800	1.60632800
H	3.24803100	-0.98599700	1.20517800
H	3.43036700	3.20565200	2.06158300
H	4.55933900	1.11179900	1.38586500
H	-0.31381200	1.15020400	2.38668000
H	1.01105600	3.23727700	2.55718800
C	-3.06204400	1.80471000	-1.38094500
C	-1.87548100	2.64849400	-0.99362300
H	-3.82521300	1.84764600	-0.59405100
H	-3.51457300	2.18572600	-2.29998900
C	-1.91422300	3.45999100	0.13774000
C	-0.83214800	4.27320300	0.46228200
C	0.30807200	4.26420900	-0.33214500
C	0.36038700	3.44494800	-1.45703400
C	-0.72828400	2.64897900	-1.78941400
H	-2.79938100	3.45904700	0.76751900
H	-0.87740800	4.90485900	1.34358400
H	1.15723800	4.88828700	-0.07387700
H	1.24885200	3.43374800	-2.08018000

H	-0.69445400	2.02114400	-2.67455200
C	0.52224800	-0.86549700	-1.60588900
C	1.97271800	-1.22202600	-1.48511800
H	0.37680400	0.19790800	-1.38321600
H	0.17144600	-1.03863600	-2.63330500
C	2.37507600	-2.50797300	-1.12860900
C	3.72798900	-2.81174800	-1.00919600
C	4.68918600	-1.83855600	-1.26389900
C	4.29098800	-0.55881200	-1.63961400
C	2.93977700	-0.25153100	-1.74184600
H	1.62569400	-3.26786200	-0.93416800
H	4.03100100	-3.81201400	-0.71788800
H	5.74345100	-2.07533800	-1.16806900
H	5.03418500	0.20724900	-1.83413300
H	2.63206700	0.75658500	-2.00415600

Glc-4B_Water

Charge=+1, Multiplicity=+1

C	-2.46875100	-2.57506900	1.23392400
O	-3.16740200	-1.34799600	1.46306300
C	-2.46042700	-2.65417900	-0.29922200
C	-1.62400100	-1.57423100	-0.98828200
C	-2.35696900	-0.29165100	-0.53722000
C	-3.56972800	-0.92625800	0.16473300
H	-3.07791100	-3.41341500	1.59368400
C	-1.19224300	-2.63063700	2.04490500
O	-3.77061500	-2.12916900	-0.54625900
H	-2.35561100	-3.65793800	-0.70273100
H	-4.48307900	-0.33904300	0.22156300
O	-2.71205900	0.46139000	-1.67050300
H	-1.75634300	0.29849800	0.16346500
H	-1.79489500	-1.67925200	-2.06728700
O	-0.25204900	-1.66114300	-0.71983600
O	-0.51996100	-1.35051100	2.05752200
H	-0.51487100	-3.40385300	1.67742500
H	-1.43150200	-2.81888600	3.09123200
C	0.70080500	-1.27134400	1.71936900
C	1.39253300	-0.04239400	1.79125500

H	1.22486100	-2.17760600	1.41884900
C	2.77014300	-0.05691100	1.50427900
C	0.74684200	1.15233800	2.17022800
C	1.48506000	2.31049600	2.26633800
C	2.85520700	2.28960600	1.98451200
C	3.49747300	1.11404000	1.60556100
H	3.24927500	-0.98322000	1.20457100
H	3.42638600	3.20876600	2.06094100
H	4.55793100	1.11641000	1.38474900
H	-0.31491600	1.14832600	2.38714600
H	1.00728100	3.23707600	2.55759700
C	-3.06313100	1.80671200	-1.37431200
C	-1.87510000	2.65028000	-0.99090100
H	-3.82317700	1.84889100	-0.58437900
H	-3.51932400	2.18869500	-2.29117600
C	-1.91119500	3.46412200	0.13887100
C	-0.82788900	4.27713600	0.45979900
C	0.31095200	4.26564800	-0.33662300
C	0.36055100	3.44413600	-1.46000800
C	-0.72941000	2.64840600	-1.78885100
H	-2.79502600	3.46479900	0.77050200
H	-0.87089100	4.91028700	1.34013900
H	1.16124800	4.88918600	-0.08077900
H	1.24797000	3.43072400	-2.08458300
H	-0.69760200	2.01871700	-2.67273200
C	0.52161800	-0.86962800	-1.61211700
C	1.97215900	-1.22461700	-1.48752500
H	0.37524900	0.19497300	-1.39605100
H	0.17193900	-1.04944300	-2.63879400
C	2.93919900	-0.25398400	-1.74370600
C	4.29041000	-0.56024400	-1.63773600
C	4.68851100	-1.83904400	-1.25875200
C	3.72730400	-2.81240600	-1.00449200
C	2.37450900	-2.50972400	-1.12773600
H	2.63147700	0.75349300	-2.00837300
H	5.03356600	0.20604600	-1.83153200
H	5.74269000	-2.07489700	-1.15963800
H	4.03020500	-3.81182500	-0.71017700

H	1.62517100	-3.26966800	-0.93328900

Glc-6B_Dioxane

Charge=+1, Multiplicity=+1

C	1.57008800	-3.10597800	-0.32101600
O	0.91592400	-3.08021100	0.93490400
C	1.83758100	-2.36670600	1.73808800
C	1.72878700	-0.87469300	1.43771500
O	0.48065200	-0.45719100	1.94643500
C	1.37194800	-1.74658400	-0.97759500
O	3.15471100	-0.25550300	-0.38428800
O	-0.02744500	-1.65485600	-1.38164100
H	1.13850400	-3.90149000	-0.92831400
C	3.03254100	-3.34306800	0.06121800
O	3.10750300	-2.84523300	1.39384800
H	1.61932000	-2.57342600	2.78583700
H	3.73551400	-2.80537000	-0.57796500
H	3.26775700	-4.40921200	0.06351200
C	0.39159900	0.94662600	2.23821200
C	-0.97763900	1.43364800	1.87373300
H	1.14553400	1.49533300	1.66431600
H	0.59781900	1.08846500	3.30406300
C	-1.20682300	2.00003400	0.61921400
C	-2.48201300	2.41460800	0.25413200
C	-3.54079100	2.26914600	1.14409600
C	-3.32005000	1.71195800	2.40025700
C	-2.04358900	1.29532200	2.76267000
H	-0.37804400	2.14386200	-0.06879900
H	-2.64704000	2.86104200	-0.72081600
H	-4.53501700	2.60143900	0.86509900
H	-4.14153100	1.61006000	3.10165500
H	-1.86974400	0.86749000	3.74543400
C	3.30654300	0.61356100	-1.50727000
C	2.58821300	1.92240900	-1.32803200
H	4.38489400	0.76188500	-1.59012800
H	2.96628700	0.12513900	-2.43084200
C	1.62344000	2.33618600	-2.24346500
C	0.96133100	3.54942600	-2.07518000

C	1.25027700	4.34966000	-0.97710700
C	2.20679800	3.93842100	-0.05163800
C	2.87526500	2.73447800	-0.22854800
H	1.39539600	1.70833300	-3.10027600
H	0.21899400	3.86606400	-2.80018200
H	0.73276800	5.29285000	-0.83976600
H	2.43642500	4.56401000	0.80433200
H	3.62734600	2.41717300	0.48797800
C	-0.93694600	-1.37714100	-0.52789700
C	1.82387400	-0.57798000	-0.07529800
H	2.55710500	-0.35487000	1.92923700
H	1.90025000	-1.69537000	-1.92906700
H	1.17063500	0.28198900	-0.28022000
C	-2.29819500	-1.40039400	-0.89687200
C	-2.72961800	-1.75956700	-2.19083900
C	-4.07722200	-1.75262200	-2.47306700
C	-4.99604600	-1.39344000	-1.47922800
C	-4.57677100	-1.03638100	-0.20083300
C	-3.22725500	-1.03728600	0.09647700
H	-2.87502600	-0.75181500	1.08239700
H	-6.05556200	-1.39331700	-1.71289500
H	-5.30020100	-0.75323700	0.55424600
H	-2.00130900	-2.03673300	-2.94429000
H	-4.43082700	-2.02513200	-3.46037700
H	-0.64296500	-1.10304400	0.49021500

Glc-6B_Toluene

Charge=+1, Multiplicity=+1

C	1.57203000	-3.10534000	-0.31886100
O	0.91806400	-3.07911100	0.93741200
C	1.83918900	-2.36422000	1.73977600
C	1.72930300	-0.87264000	1.43810300
O	0.48084900	-0.45576300	1.94662700
C	1.37335700	-1.74672200	-0.97660900
O	3.15494200	-0.25335400	-0.38371000
O	-0.02585800	-1.65613300	-1.38138700
H	1.14068100	-3.90142200	-0.92550300
C	3.03446000	-3.34167200	0.06335400

O	3.10957400	-2.84222900	1.39566200
H	1.62145800	-2.57019200	2.78777500
H	3.73721500	-2.80476800	-0.57668500
H	3.26963700	-4.40779200	0.06691100
C	0.39127700	0.94815000	2.23776400
C	-0.97861300	1.43437400	1.87461200
H	1.14428100	1.49694200	1.66272100
H	0.59877600	1.09052800	3.30329100
C	-1.20939400	2.00145300	0.62070400
C	-2.48524000	2.41528300	0.25703100
C	-3.54314100	2.26832800	1.14778500
C	-3.32091500	1.71022700	2.40326200
C	-2.04376900	1.29451100	2.76430800
H	-0.38144000	2.14596000	-0.06817500
H	-2.65153800	2.86195900	-0.71756200
H	-4.53799700	2.59939300	0.86960700
H	-4.14178300	1.60670600	3.10510200
H	-1.86885600	0.86591400	3.74652200
C	3.30681400	0.61473500	-1.50746500
C	2.58739200	1.92320900	-1.32960000
H	4.38510100	0.76387000	-1.58985500
H	2.96753100	0.12526900	-2.43073800
C	1.62197000	2.33491800	-2.24528200
C	0.95826700	3.54741700	-2.07802200
C	1.24640300	4.34909000	-0.98076000
C	2.20375400	3.94005400	-0.05513300
C	2.87369600	2.73675200	-0.23100000
H	1.39423700	1.70557900	-3.10103700
H	0.21509900	3.86217200	-2.80297100
H	0.72747200	5.29160300	-0.84412900
H	2.43266800	4.56668600	0.80025000
H	3.62624300	2.42114300	0.48575300
C	-0.93588900	-1.37754400	-0.52858300
C	1.82412400	-0.57703200	-0.07513200
H	2.55710100	-0.35173400	1.92936900
H	1.90184500	-1.69621600	-1.92800600
H	1.17000700	0.28203300	-0.28077100
C	-2.29694700	-1.40218000	-0.89811700

C	-2.72760900	-1.76313200	-2.19182700
C	-4.07512200	-1.75721300	-2.47454500
C	-4.99452900	-1.39735300	-1.48149600
C	-4.57596800	-1.03863300	-0.20331300
C	-3.22655600	-1.03846100	0.09445300
H	-2.87481900	-0.75179000	1.08014600
H	-6.05392100	-1.39807400	-1.71562100
H	-5.29977400	-0.75503900	0.55119600
H	-1.99887800	-2.04071600	-2.94467700
H	-4.42818900	-2.03106000	-3.46166400
H	-0.64278400	-1.10238700	0.48947300

Glc-6B_Et_2O

Charge=+1, Multiplicity=+1

C	1.61153000	-3.07887600	-0.37092500
O	0.98949600	-3.08917800	0.90281400
C	1.91558500	-2.37122200	1.69484800
C	1.76867300	-0.87708800	1.42535500
O	0.52228700	-0.49731600	1.96648300
C	1.38100800	-1.71133900	-0.99808400
O	3.14648000	-0.19272900	-0.40763200
O	-0.02393600	-1.63027400	-1.38213600
H	1.17784800	-3.86918100	-0.98288400
C	3.08572700	-3.29939800	-0.02948400
O	3.18758300	-2.81668600	1.30875400
H	1.72987800	-2.60105200	2.74407000
H	3.76443300	-2.74383100	-0.67928300
H	3.33556200	-4.36195100	-0.04722300
C	0.40497300	0.90043300	2.27877000
C	-0.96234700	1.37761300	1.89321800
H	1.16466700	1.46846600	1.73260800
H	0.58450900	1.02609400	3.35110500
C	-2.03914700	1.23575600	2.76833100
C	-3.31337100	1.64321000	2.38773700
C	-3.52117500	2.19544600	1.12724100
C	-2.45133600	2.34598700	0.25128300
C	-1.17875400	1.93984200	0.63427000
H	-1.87592800	0.81063600	3.75416800

H	-4.14405400	1.53462500	3.07713500
H	-4.51463800	2.51605000	0.83219300
H	-2.60550000	2.78854000	-0.72725000
H	-0.34296900	2.08672300	-0.04431400
C	3.26154000	0.69250400	-1.52169000
C	2.51798400	1.98425000	-1.31665500
H	4.33490300	0.86638100	-1.61918400
H	2.91868200	0.20867400	-2.44599100
C	1.56391200	2.40895900	-2.23845900
C	0.88004400	3.60707100	-2.04936000
C	1.13686000	4.38185000	-0.92512600
C	2.08242000	3.95932500	0.00653900
C	2.77200900	2.77024500	-0.19030800
H	1.35843000	1.79973300	-3.11406100
H	0.14319800	3.92984900	-2.77723300
H	0.60027800	5.31167900	-0.77061100
H	2.28491500	4.56237200	0.88524300
H	3.51290500	2.44301300	0.53321500
C	-0.92982500	-1.39142900	-0.51379600
C	1.82789200	-0.55091500	-0.08284600
H	2.59453900	-0.34841400	1.91165100
H	1.89495700	-1.63778000	-1.95550800
H	1.15310700	0.29792100	-0.25902200
C	-2.29264700	-1.41621100	-0.87750300
C	-2.72285900	-1.72197100	-2.18535100
C	-4.07129400	-1.72015900	-2.46404700
C	-4.99208100	-1.41918900	-1.45300700
C	-4.57388400	-1.11573000	-0.16036500
C	-3.22348500	-1.11127300	0.13326100
H	-2.87185800	-0.86585800	1.12975200
H	-6.05208000	-1.42223600	-1.68395700
H	-5.29954800	-0.87869100	0.60827100
H	-1.99302600	-1.95424400	-2.95211600
H	-4.42317500	-1.95254200	-3.46212500
H	-0.63304700	-1.15165100	0.51201600

Glc-6B_$CHCl_3$

Charge=+1, Multiplicity=+1

C	1.61078400	-3.08007100	-0.37244900
O	0.99146300	-3.09033400	0.90285500
C	1.91862000	-2.37160400	1.69262200
C	1.77030400	-0.87767200	1.42303200
O	0.52436600	-0.49881700	1.96578200
C	1.37946200	-1.71272800	-0.99968300
O	3.14532100	-0.19316300	-0.41198100
O	-0.02572200	-1.63123400	-1.38259000
H	1.17584500	-3.87038800	-0.98340700
C	3.08546500	-3.30075300	-0.03379500
O	3.19032400	-2.81639000	1.30383000
H	1.73541500	-2.60120600	2.74234600
H	3.76309100	-2.74629800	-0.68560500
H	3.33471900	-4.36344400	-0.05087900
C	0.40635200	0.89896600	2.27773900
C	-0.96134500	1.37527200	1.89243500
H	1.16558600	1.46722300	1.73122100
H	0.58613400	1.02498500	3.34997700
C	-2.03782200	1.23311200	2.76789300
C	-3.31249600	1.63921500	2.38735100
C	-3.52110900	2.19040100	1.12652300
C	-2.45159800	2.34126700	0.25020500
C	-1.17855200	1.93655400	0.63320000
H	-1.87409000	0.80846900	3.75385000
H	-4.14298800	1.52991600	3.07685700
H	-4.51504400	2.50932800	0.83124300
H	-2.60642600	2.78241700	-0.72884800
H	-0.34310100	2.08336500	-0.04581100
C	3.25798900	0.69361400	-1.52499600
C	2.51653500	1.98598900	-1.31562100
H	4.33122200	0.86656700	-1.62556300
H	2.91175800	0.21166300	-2.44888900
C	1.56324500	2.41539500	-2.23614500
C	0.88162100	3.61419300	-2.04319300
C	1.13972600	4.38486300	-0.91638900
C	2.08439900	3.95755500	0.01400200
C	2.77188400	2.76787800	-0.18671400
H	1.35624100	1.80907800	-3.11342600

H	0.14503200	3.94039200	-2.76980300
H	0.60462700	5.31503400	-0.75876100
H	2.28770200	4.55714000	0.89489400
H	3.51180500	2.43673700	0.53602400
C	-0.93105900	-1.39331000	-0.51356100
C	1.82726100	-0.55191500	-0.08532700
H	2.59631700	-0.34822100	1.90822400
H	1.89266500	-1.63977900	-1.95751800
H	1.15186400	0.29652800	-0.26076300
C	-2.29404400	-1.41514200	-0.87685700
C	-2.72487000	-1.71285300	-2.18632300
C	-4.07341800	-1.70856000	-2.46452300
C	-4.99370200	-1.41338500	-1.45133200
C	-4.57485900	-1.11785800	-0.15704500
C	-3.22432100	-1.11545400	0.13598400
H	-2.87217200	-0.87584500	1.13368700
H	-6.05378200	-1.41453300	-1.68187800
H	-5.30014000	-0.88517300	0.61326600
H	-1.99542900	-1.94086100	-2.95470700
H	-4.42568800	-1.93463200	-3.46390800
H	-0.63388300	-1.15676800	0.51281900

Glc-6B_DCM

Charge=+1, Multiplicity=+1

C	1.81406600	-2.96612100	-0.38848000
O	1.19315300	-3.02920100	0.88524600
C	2.07451600	-2.26865800	1.68687200
C	1.83948500	-0.78302900	1.43683800
O	0.57094500	-0.48880100	1.98031900
C	1.51214200	-1.60597300	-1.00025400
O	3.16435000	0.03246500	-0.38262300
O	0.11004400	-1.59805600	-1.40113800
H	1.42587800	-3.77072200	-1.01175100
C	3.29753800	-3.10868900	-0.04753900
O	3.37195400	-2.63411500	1.29659000
H	1.90295800	-2.52166400	2.73326400
H	3.94504800	-2.51052400	-0.69116500
H	3.60593100	-4.15536000	-0.07612000

C	0.37047700	0.89322400	2.31700000
C	-1.03210500	1.28313500	1.96199800
H	1.08151400	1.51753100	1.76636400
H	0.56169200	1.01585700	3.38779300
C	-1.30701300	1.84789800	0.71598100
C	-2.60998400	2.17509400	0.36023200
C	-3.65253500	1.93982000	1.25049900
C	-3.38733500	1.38020900	2.49705400
C	-2.08232900	1.05341900	2.85059700
H	-0.49509800	2.05606700	0.02485300
H	-2.80950200	2.61887400	-0.60957100
H	-4.67034200	2.19549300	0.97567100
H	-4.19746200	1.20118300	3.19603900
H	-1.87495700	0.62210200	3.82530800
C	3.21330200	0.91133900	-1.50802100
C	2.34880800	2.13023400	-1.33301800
H	4.26675500	1.18456300	-1.59175000
H	2.93289700	0.38437800	-2.42861200
C	1.33427200	2.42083100	-2.24300400
C	0.53866700	3.55202700	-2.07871400
C	0.74425300	4.39297000	-0.99182000
C	1.74866000	4.10287700	-0.07099700
C	2.54849500	2.98086300	-0.24311500
H	1.16759000	1.75957400	-3.08881000
H	-0.24567300	3.76999500	-2.79581600
H	0.12083800	5.27034500	-0.85694700
H	1.91009600	4.75622700	0.77984200
H	3.33455200	2.75720000	0.47203500
C	-0.81613000	-1.41929300	-0.54076100
C	1.87735600	-0.43157700	-0.06604800
H	2.62978500	-0.21161000	1.93369200
H	2.03343200	-1.49362800	-1.94959600
H	1.14191500	0.36760800	-0.22975000
C	-2.17230700	-1.50767300	-0.91891400
C	-3.12740300	-1.27641900	0.08890800
C	-4.47280000	-1.35041400	-0.21808800
C	-4.86212600	-1.64755100	-1.52134900
C	-3.91722300	-1.87350700	-2.52956000

C	-2.57312400	-1.80735500	-2.23737900
H	-1.82500000	-1.98211200	-3.00164500
H	-5.91828300	-1.70413600	-1.76256400
H	-4.24636900	-2.10185800	-3.53626800
H	-2.79892800	-1.03379600	1.09408800
H	-5.21729000	-1.17159300	0.54813100
H	-0.54328300	-1.18441100	0.49262100

Glc-6B_ACN

Charge=+1, Multiplicity=+1

C	1.68208500	-3.04759200	-0.38098700
O	1.08235300	-3.07520300	0.90538200
C	2.00042700	-2.33300000	1.68124600
C	1.80951800	-0.84375700	1.41553100
O	0.55884800	-0.50143800	1.97186000
C	1.41699700	-1.68549000	-1.00483800
O	3.14338700	-0.11189000	-0.43257400
O	0.01063300	-1.63144300	-1.38524600
H	1.25607300	-3.84542700	-0.98763400
C	3.16466400	-3.23813200	-0.06274300
O	3.27956100	-2.74497500	1.27309400
H	1.83995600	-2.56686600	2.73390100
H	3.82184900	-2.67406300	-0.72661900
H	3.43388300	-4.29576600	-0.07939300
C	0.40621300	0.89224000	2.28680000
C	-0.97799000	1.33142100	1.91646800
H	1.14416400	1.48131400	1.73338300
H	0.59414800	1.02241800	3.35712900
C	-2.04136900	1.15698100	2.80215100
C	-3.33092900	1.52726700	2.43517800
C	-3.56767900	2.07533200	1.17776300
C	-2.51169200	2.25735000	0.29080400
C	-1.22394900	1.88758300	0.66032100
H	-1.85631300	0.73262400	3.78445400
H	-4.15173700	1.38835900	3.13085900
H	-4.57399700	2.36182500	0.89122200
H	-2.68886900	2.69069500	-0.68795200
H	-0.40120400	2.05287600	-0.02965300

C	3.20872100	0.77237800	-1.55186200
C	2.44095800	2.04747100	-1.32820600
H	4.27453100	0.97175800	-1.67887600
H	2.85182200	0.27789700	-2.46385000
C	2.70163500	2.83685000	-0.20558100
C	1.98951400	4.00957600	0.00996100
C	1.01446700	4.41252400	-0.90005400
C	0.75015800	3.63362300	-2.02014500
C	1.45646900	2.45147200	-2.22766500
H	3.46285700	2.52343200	0.50270000
H	2.19588400	4.61307000	0.88758500
H	0.45844500	5.32837400	-0.73031000
H	-0.01420600	3.93753000	-2.72746600
H	1.23943600	1.83541700	-3.09556000
C	-0.89954300	-1.42070000	-0.51615700
C	1.84061700	-0.51413900	-0.09205200
H	2.62420000	-0.29224900	1.89540000
H	1.92674900	-1.60234700	-1.96335500
H	1.13858000	0.31381800	-0.25815700
C	-2.26130300	-1.45123700	-0.88464100
C	-3.20036000	-1.19736300	0.13243800
C	-4.54940800	-1.20935100	-0.16757400
C	-4.95775400	-1.46665600	-1.47358200
C	-4.02842800	-1.71514600	-2.49078000
C	-2.68109000	-1.71127000	-2.20540800
H	-1.94471900	-1.90228600	-2.97707400
H	-6.01651200	-1.47297200	-1.70987400
H	-4.37164000	-1.91071700	-3.49968300
H	-2.85679200	-0.98594000	1.13942100
H	-5.28179900	-1.01193200	0.60575200
H	-0.60873500	-1.20065200	0.51521500

Glc-6B_DMSO

Charge=+1, Multiplicity=+1

C	1.69456100	-3.04143100	-0.37935200
O	1.09457600	-3.07068800	0.90696000
C	2.00966200	-2.32481600	1.68271800
C	1.81341200	-0.83643400	1.41635200

O	0.56112700	-0.49857600	1.97188000
C	1.42519800	-1.68050900	-1.00392100
O	3.14503500	-0.09948100	-0.43134800
O	0.01905300	-1.63151400	-1.38562400
H	1.27151100	-3.84093200	-0.98583300
C	3.17761100	-3.22690500	-0.06051700
O	3.29048300	-2.73228600	1.27507200
H	1.84976500	-2.55881000	2.73544300
H	3.83316400	-2.66124100	-0.72461000
H	3.45026900	-4.28366100	-0.07628100
C	0.40365800	0.89462800	2.28628100
C	-0.98348100	1.32745500	1.91958400
H	1.13742200	1.48641400	1.73016600
H	0.59429300	1.02654800	3.35593700
C	-2.04382100	1.14769600	2.80787200
C	-3.33603100	1.51200500	2.44424100
C	-3.57846100	2.05949600	1.18763300
C	-2.52558600	2.24657100	0.29801500
C	-1.23520300	1.88258000	0.66413800
H	-1.85430700	0.72380700	3.78953100
H	-4.15448100	1.36883100	3.14183900
H	-4.58685300	2.34124100	0.90365700
H	-2.70729800	2.67897000	-0.68033200
H	-0.41511100	2.05114900	-0.02818900
C	3.20713000	0.78390700	-1.55163300
C	2.43237700	2.05513500	-1.33028400
H	4.27203800	0.98880500	-1.67740700
H	2.85408600	0.28642400	-2.46340600
C	2.69078700	2.84932700	-0.21050700
C	1.97206300	4.01834900	0.00331200
C	0.99248200	4.41270700	-0.90562900
C	0.73025200	3.62888300	-2.02279800
C	1.44327600	2.45044700	-2.22857300
H	3.45546200	2.54257600	0.49698700
H	2.17676600	4.62559300	0.87875100
H	0.43125400	5.32564800	-0.73724800
H	-0.03780400	3.92590400	-2.72905400
H	1.22769100	1.83037100	-3.09396000

C	-0.89267000	-1.42322000	-0.51765500
C	1.84380100	-0.50720700	-0.09129300
H	2.62572300	-0.28170300	1.89652300
H	1.93549800	-1.59606700	-1.96201300
H	1.13853600	0.31788900	-0.25789600
C	-2.25392600	-1.45759000	-0.88762100
C	-3.19488500	-1.20669100	0.12843900
C	-4.54353100	-1.22241700	-0.17318200
C	-4.94960700	-1.48035400	-1.47979300
C	-4.01838200	-1.72583700	-2.49597500
C	-2.67138800	-1.71829400	-2.20898400
H	-1.93354400	-1.90682800	-2.97985400
H	-6.00807000	-1.48948200	-1.71734000
H	-4.35980000	-1.92187900	-3.50539900
H	-2.85315600	-0.99457800	1.13592800
H	-5.27739600	-1.02727600	0.59932700
H	-0.60362700	-1.20225100	0.51399000

Glc-6B_Water

Charge=+1, Multiplicity=+1

C	1.71845700	-3.02889700	-0.37868200
O	1.11791000	-3.06285000	0.90736900
C	2.02716800	-2.31088800	1.68390200
C	1.82069500	-0.82384700	1.41801000
O	0.56556000	-0.49514800	1.97271200
C	1.44067200	-1.66959900	-1.00300200
O	3.14781400	-0.07497300	-0.42798700
O	0.03487300	-1.62993800	-1.38696100
H	1.30118100	-3.83090400	-0.98580200
C	3.20244800	-3.20455500	-0.05891900
O	3.31120600	-2.70924600	1.27688400
H	1.86834800	-2.54634400	2.73647600
H	3.85463500	-2.63451300	-0.72256800
H	3.48217600	-4.25944900	-0.07461400
C	0.39869400	0.89663100	2.28824400
C	-0.99301300	1.31864300	1.92638600
H	1.12594300	1.49422100	1.72973400
H	0.59213500	1.02977600	3.35726800

C	-2.04916200	1.12898600	2.81761600
C	-3.34541200	1.48316500	2.45837100
C	-3.59605800	2.03053500	1.20330400
C	-2.54746600	2.22716600	0.31067500
C	-1.25307200	1.87304400	0.67234500
H	-1.85321500	0.70525500	3.79807300
H	-4.16059500	1.33210700	3.15812000
H	-4.60759700	2.30424900	0.92268500
H	-2.73569600	2.65885000	-0.66675100
H	-0.43667300	2.04817000	-0.02267700
C	3.20405400	0.80725200	-1.54972600
C	2.41506200	2.07046700	-1.33303300
H	4.26711100	1.02335500	-1.67223000
H	2.85953500	0.30427700	-2.46164700
C	2.66758500	2.87364100	-0.21829700
C	1.93564700	4.03511900	-0.00821000
C	0.94835500	4.41282300	-0.91590700
C	0.69164600	3.61983300	-2.02789700
C	1.41810200	2.44896000	-2.22993100
H	3.43820400	2.57987500	0.48826400
H	2.13598900	4.64941800	0.86331100
H	0.37675800	5.31986500	-0.75047400
H	-0.08258700	3.90359400	-2.73285700
H	1.20670800	1.82156500	-3.09105300
C	-0.87948100	-1.42728300	-0.52056900
C	1.84964700	-0.49359400	-0.08938800
H	2.62856700	-0.26351200	1.89916500
H	1.95189400	-1.58140600	-1.96024700
H	1.13806900	0.32606700	-0.25594300
C	-2.23995700	-1.46939800	-0.89268500
C	-3.18400800	-1.22436800	0.12193700
C	-4.53202700	-1.24750700	-0.18199100
C	-4.93444300	-1.50695800	-1.48945500
C	-4.00014500	-1.74662500	-2.50417200
C	-2.65367500	-1.73164200	-2.21489400
H	-1.91345900	-1.91538300	-2.98463500
H	-5.99243500	-1.52179700	-1.72880300
H	-4.33869700	-1.94389000	-3.51431900

H	-2.84525700	-1.01088300	1.13016200
H	-5.26827600	-1.05682100	0.58935400
H	-0.59354000	-1.20513000	0.51167700

Gal-4B_Dioxane

Charge=+1, Multiplicity=+1

O	-1.29392800	-0.08978900	-0.81986400
C	-1.11662100	-1.35591000	-0.26303000
C	-0.23484600	0.54092800	-0.08005600
O	-1.23200400	-1.12968100	1.15084400
C	0.99480600	-0.28598500	-0.47858700
C	0.33949300	-1.68762800	-0.61081000
H	1.74914400	-0.29772100	0.31358700
O	1.55563300	0.10957300	-1.70007800
O	0.82021600	-2.68675100	0.23479800
H	0.39210100	-1.99066100	-1.66524300
H	-1.87905300	-2.06441300	-0.57686500
C	-0.59702200	0.12948500	1.35607200
C	-1.55595800	1.04988300	2.08936700
H	0.29351000	0.01073400	1.98230200
H	-0.20054400	1.60591800	-0.28792900
C	2.01354700	-3.31841900	-0.21027500
C	3.25355900	-2.48942300	0.00033600
H	1.91892800	-3.59218700	-1.26906400
H	2.07471500	-4.24068700	0.37305100
C	4.12046200	-2.21943900	-1.05365700
C	5.27300300	-1.46541400	-0.84753300
C	5.55820400	-0.96200300	0.41534500
C	4.69276700	-1.22411700	1.47540300
C	3.55257000	-1.98893400	1.26947400
H	3.89051600	-2.59523600	-2.04627200
H	5.94216600	-1.26454500	-1.67755800
H	6.45289200	-0.37009900	0.57638800
H	4.91692900	-0.84175400	2.46600800
H	2.88221300	-2.20238900	2.09711000
C	2.63868300	1.03134600	-1.55357200
C	2.23894500	2.28475200	-0.82556200
H	2.95956700	1.25115200	-2.57316100

H	3.46222800	0.53420000	-1.02783100
C	1.47908700	3.26525600	-1.46529900
C	1.00150000	4.36136200	-0.75809800
C	1.28139200	4.49153500	0.60034500
C	2.05625900	3.53155200	1.24160300
C	2.53573300	2.43672100	0.52817900
H	1.24889600	3.15728800	-2.52115300
H	0.41361400	5.11895500	-1.26575100
H	0.91091800	5.34967500	1.15135200
H	3.14573800	1.68728000	1.02600900
H	2.29744400	3.64108900	2.29398000
O	-2.79098200	1.08283900	1.34566600
H	-1.18780300	2.07404500	2.11757500
H	-1.76157300	0.68880700	3.09988800
C	-3.53060500	0.04414100	1.41855300
C	-4.54396400	-0.19696800	0.46791300
H	-3.39357900	-0.63128000	2.26228400
C	-5.41853200	-1.27668600	0.70104900
C	-6.41720200	-1.55303500	-0.21155600
C	-6.53807000	-0.76392400	-1.35465400
C	-5.66640200	0.30183000	-1.59780600
C	-4.66676500	0.59117800	-0.69468000
H	-5.30442600	-1.87988300	1.59595800
H	-7.09957100	-2.37749500	-0.04317600
H	-7.32034900	-0.98445300	-2.07322800
H	-5.77400400	0.89318300	-2.49928300
H	-3.96624900	1.39843400	-0.87069200

Gal -4B_Toluene

Charge=+1, Multiplicity=+1

O	0.46802600	1.82450800	-1.56965200
C	0.18235500	0.68287100	-0.80969200
C	0.78635100	2.66587400	-0.45281400
O	-0.82796300	1.11944400	0.10572400
C	1.92974300	1.92187900	0.22599500
C	1.48993000	0.45296900	-0.01868900
H	1.97029700	2.11383700	1.30597500
O	3.12681200	2.30081500	-0.39213300

O	1.35871000	-0.21531600	1.19920900
H	2.21294900	-0.04309300	-0.67534700
H	-0.19895600	-0.13184000	-1.41822700
C	-0.48959100	2.47633100	0.38595700
C	-1.63290100	3.35433300	-0.11954700
H	-0.33625100	2.60097400	1.45934800
H	1.01090600	3.67993300	-0.77612500
C	1.36823000	-1.63411100	1.09257000
C	0.12411200	-2.18390400	0.45060000
H	1.45993300	-1.99086800	2.12087300
H	2.26041700	-1.95466700	0.54081800
C	0.18237100	-2.84810700	-0.77231000
C	-0.97921100	-3.31920400	-1.37855900
C	-2.20955200	-3.12647700	-0.76148100
C	-2.27506200	-2.46850800	0.46472900
C	-1.11548400	-2.00116500	1.06533700
H	1.14500600	-2.99551600	-1.25417800
H	-0.92168400	-3.83879600	-2.32928300
H	-3.11610800	-3.49707100	-1.22919600
H	-3.23270900	-2.32671900	0.95485400
H	-1.16536000	-1.48058800	2.01654500
C	4.30306600	1.91858500	0.31080300
C	4.64136000	0.45559600	0.19173400
H	4.20799500	2.19627300	1.36910400
H	5.09750100	2.52634600	-0.12882900
C	4.71902300	-0.35406000	1.32080000
C	5.03191500	-1.70541800	1.20312100
C	5.25881400	-2.25917700	-0.05060000
C	5.18144900	-1.45575700	-1.18638300
C	4.88067100	-0.10608400	-1.06426200
H	4.52748900	0.07318900	2.30065300
H	5.09230200	-2.32567600	2.09132400
H	5.50080300	-3.31254500	-0.14508500
H	4.82227100	0.52155000	-1.94869800
H	5.36649500	-1.88224800	-2.16682200
O	-2.85960800	2.66283400	0.19212000
H	-1.57345500	3.48826400	-1.20202000
H	-1.68653700	4.31498900	0.38656400

C	-3.12334600	1.64826800	-0.54122100
C	-4.10502700	0.70399000	-0.16789600
H	-2.64378800	1.57788300	-1.51637600
C	-4.69655700	0.71523900	1.11035800
C	-5.65518500	-0.22695300	1.41509700
C	-6.03060700	-1.17367000	0.45637800
C	-5.44398300	-1.19477100	-0.80666800
C	-4.47235700	-0.26361400	-1.12104400
H	-4.38938600	1.45773600	1.83741300
H	-6.11931400	-0.23599100	2.39411200
H	-6.78882000	-1.90881700	0.70443600
H	-5.74107900	-1.94092400	-1.53380500
H	-3.99189100	-0.27364100	-2.09403500

Gal -4B_Et2O

Charge=+1, Multiplicity=+1

O	0.40493500	0.40939600	-0.75038600
C	-0.45849600	-0.51556800	-0.15900600
C	1.54282100	0.09183000	0.06562700
O	-0.40976200	-0.19884900	1.24220500
C	1.73776600	-1.41144800	-0.20127200
C	0.27266400	-1.85323400	-0.39144700
H	2.16977900	-1.92718000	0.66467900
O	2.46975900	-1.69387500	-1.36350700
O	-0.05095600	-2.89405900	0.48220600
H	0.13243300	-2.14748000	-1.43969000
H	-1.47855000	-0.41514100	-0.51813100
C	0.93519300	0.22020500	1.46993100
C	0.89520800	1.61966000	2.07467800
H	1.42180400	-0.44373200	2.19097100
H	2.37953500	0.74352800	-0.16440300
C	-1.19173500	-3.65240600	0.08970500
C	-2.46115700	-2.84477600	0.07636100
H	-1.25203100	-4.46234000	0.81945500
H	-1.01497400	-4.09207200	-0.90029700
C	-3.11401700	-2.56353300	-1.12195900
C	-4.24280700	-1.74940000	-1.14053900
C	-4.72859600	-1.21154200	0.04532900

C	-4.09033300	-1.49923100	1.24955300
C	-2.96494100	-2.31196500	1.26420600
H	-2.72981700	-2.97687800	-2.05022400
H	-4.74015000	-1.53512000	-2.08060800
H	-5.60888400	-0.57737300	0.03360600
H	-4.47531100	-1.09208500	2.17908300
H	-2.46046100	-2.52708400	2.20099700
C	3.87014100	-1.85037700	-1.14190700
C	4.53094600	-0.59343600	-0.64647300
H	4.27472900	-2.14726000	-2.11107000
H	4.03577400	-2.67034200	-0.43102000
C	4.71347500	0.48807700	-1.51015100
C	5.23246200	1.68683200	-1.03884200
C	5.58051800	1.81669400	0.30395600
C	5.41762000	0.73988800	1.16793400
C	4.89443700	-0.45919800	0.69202400
H	4.43346800	0.38955500	-2.55482800
H	5.37075500	2.52137300	-1.71827300
H	5.98936900	2.75220600	0.67098100
H	4.76655800	-1.29987500	1.36830100
H	5.70128700	0.83109400	2.21126900
O	0.00512700	2.40951400	1.26566800
H	1.86344700	2.11489900	2.04723300
H	0.51298100	1.59131200	3.09827700
C	-1.23798400	2.11972600	1.36564400
C	-2.15135600	2.47212200	0.34827900
H	-1.59154600	1.65554000	2.28479400
C	-3.51980200	2.24422900	0.58735700
C	-4.44143200	2.54621700	-0.39593900
C	-4.00148800	3.06301100	-1.61305700
C	-2.64286000	3.28012800	-1.86111500
C	-1.71183400	2.98647600	-0.88816500
H	-3.83919600	1.83053400	1.53794400
H	-5.49791200	2.37884100	-0.22490400
H	-4.72621700	3.29537900	-2.38611700
H	-2.32406500	3.67263300	-2.81939600
H	-0.65210700	3.12616700	-1.06238500

Gal-4B_$CHCl_3$

Charge=+1, Multiplicity=+1

O	-0.41075500	-0.42558900	-0.75917200
C	0.45905200	0.51152800	-0.19652200
C	-1.54133200	-0.08645400	0.05855300
O	0.42123500	0.22826700	1.21168700
C	-1.73608100	1.40958300	-0.24545900
C	-0.27157200	1.84456600	-0.45588500
H	-2.16226800	1.94844200	0.60916500
O	-2.47520900	1.66120900	-1.41059500
O	0.05664800	2.90652900	0.39016900
H	-0.13563500	2.11400200	-1.51146100
H	1.47568100	0.40047500	-0.56178900
C	-0.92247200	-0.18263500	1.46035700
C	-0.87999800	-1.56831900	2.09576100
H	-1.40202000	0.49870000	2.16983000
H	-2.38088300	-0.74237300	-0.14778900
C	1.21235100	3.63691800	-0.01003900
C	2.47735200	2.82424500	0.05084400
H	1.26031000	4.48152000	0.68038500
H	1.06498700	4.02871700	-1.02474500
C	2.93702800	2.33728000	1.27544500
C	4.06438000	1.52925400	1.33346200
C	4.74928100	1.20142300	0.16574300
C	4.30567500	1.69107400	-1.05708300
C	3.17392400	2.49961700	-1.11133600
H	2.39724900	2.58612900	2.18371000
H	4.41466600	1.15698700	2.29096100
H	5.63247500	0.57281800	0.21149800
H	4.83820300	1.44319600	-1.96924500
H	2.82161200	2.87514900	-2.06794300
C	-3.87385200	1.82741800	-1.18446600
C	-4.53414700	0.58807800	-0.64587200
H	-4.28446800	2.09504100	-2.15960000
H	-4.03288900	2.66924300	-0.49819800
C	-4.72431600	-0.51962200	-1.47399700
C	-5.24221500	-1.70204200	-0.96189300
C	-5.58149800	-1.78904100	0.38663200

C	-5.41120500	-0.68589300	1.21523100
C	-4.88957200	0.49682800	0.69839800
H	-4.45057600	-0.45459400	-2.52298100
H	-5.38614100	-2.55726400	-1.61386400
H	-5.98856400	-2.71210600	0.78578200
H	-4.75524000	1.35792600	1.34718500
H	-5.68744700	-0.74374000	2.26293700
O	-0.00205200	-2.37920600	1.29412700
H	-1.85023400	-2.06026900	2.08968600
H	-0.48638400	-1.51896600	3.11413500
C	1.24300800	-2.09036200	1.37048200
C	2.14052500	-2.46606200	0.34725800
H	1.61015800	-1.60625200	2.27367400
C	3.51189300	-2.22521900	0.55540700
C	4.41647300	-2.54452100	-0.43848800
C	3.95705400	-3.09392000	-1.63389700
C	2.59545700	-3.32550800	-1.85069500
C	1.68114900	-3.01267200	-0.86799400
H	3.84672700	-1.78501500	1.48874200
H	5.47477600	-2.36483900	-0.29305300
H	4.66870600	-3.34029400	-2.41474500
H	2.26097900	-3.74331500	-2.79277400
H	0.61920300	-3.16225300	-1.01892600

Gal-4B_DCM

Charge=+1, Multiplicity=+1

O	-1.27192800	-0.23713800	-0.77225100
C	-1.02580100	-1.50918400	-0.24712900
C	-0.25617600	0.43183700	-0.00532800
O	-1.16130400	-1.33356800	1.16504900
C	1.01774800	-0.30775600	-0.43181600
C	0.44995500	-1.74351600	-0.59540800
H	1.78233500	-0.28646000	0.34972900
O	1.53790800	0.14663000	-1.65454500
O	0.99462600	-2.72717700	0.23385100
H	0.52664200	-2.02603200	-1.65347500
H	-1.74384900	-2.24873500	-0.59159900
C	-0.59759500	-0.04694700	1.41662400

C	-1.60295500	0.78723000	2.18351900
H	0.29793800	-0.13551100	2.04064900
H	-0.28275400	1.50384000	-0.17560400
C	2.20967100	-3.29356100	-0.23777900
C	3.41077800	-2.40263400	-0.05214200
H	2.10758600	-3.57011300	-1.29489100
H	2.33316800	-4.21257600	0.34117900
C	4.24681000	-2.10065500	-1.12287000
C	5.36571600	-1.29100800	-0.94232400
C	5.64826400	-0.76486200	0.31227500
C	4.81288500	-1.05790300	1.38851600
C	3.70633300	-1.87709800	1.20775200
H	4.01787200	-2.49502300	-2.10858700
H	6.00866200	-1.06273000	-1.78597100
H	6.51434200	-0.12691900	0.45342200
H	5.03052200	-0.65160500	2.37098100
H	3.05726500	-2.10833300	2.04730800
C	2.58446700	1.10721900	-1.50515800
C	2.14025800	2.34812100	-0.78187300
H	2.90062700	1.33880700	-2.52391900
H	3.42528000	0.64176200	-0.97761800
C	1.34027100	3.29423000	-1.42504300
C	0.84431100	4.38928400	-0.72894000
C	1.14585100	4.55239400	0.62141700
C	1.95482600	3.62372200	1.26669600
C	2.45055600	2.52845200	0.56515900
H	1.09589500	3.16162800	-2.47499900
H	0.22287000	5.11852300	-1.23819900
H	0.75781200	5.40729800	1.16526500
H	3.08287200	1.80193500	1.06878300
H	2.20207200	3.75258800	2.31534400
O	-2.81456900	0.87646700	1.40214500
H	-1.25931600	1.81053200	2.31948400
H	-1.83687900	0.33234700	3.14831500
C	-3.58709400	-0.13687400	1.39586500
C	-4.61694900	-0.25948200	0.43998200
H	-3.45056600	-0.89771100	2.16315900
C	-5.49121800	-1.35741200	0.55770300

C	-6.50380400	-1.52212700	-0.36756000
C	-6.64028600	-0.60228300	-1.40571000
C	-5.77108000	0.48619300	-1.53173700
C	-4.75713100	0.66372800	-0.61624800
H	-5.36454500	-2.06318200	1.37193200
H	-7.18483200	-2.36082500	-0.28915700
H	-7.43361300	-0.73511300	-2.13353500
H	-5.89300100	1.18338400	-2.35200000
H	-4.06393200	1.49188100	-0.70064600

Gal-4B_ACN

Charge=+1, Multiplicity=+1

O	-1.25377600	-0.33124500	-0.74496200
C	-0.96124500	-1.59927400	-0.23094000
C	-0.26209800	0.36700500	0.02813300
O	-1.10206300	-1.44270400	1.18059300
C	1.03649500	-0.32021700	-0.40937400
C	0.52191200	-1.77424600	-0.58549400
H	1.80259600	-0.27678200	0.36953400
O	1.53632200	0.16320700	-1.63074900
O	1.10849200	-2.74470900	0.23180300
H	0.60577900	-2.04469600	-1.64609600
H	-1.65171000	-2.36084800	-0.58357800
C	-0.58204500	-0.13940100	1.44609100
C	-1.60900300	0.65373300	2.22442800
H	0.31830300	-0.20426500	2.06600400
H	-0.32977300	1.43903800	-0.12994100
C	2.33876600	-3.26319000	-0.25475400
C	3.50840200	-2.32890400	-0.07904400
H	2.23565200	-3.53951600	-1.31175100
H	2.50314000	-4.17915000	0.31895100
C	4.32736700	-2.00291000	-1.15616600
C	5.41774600	-1.15296700	-0.98618800
C	5.68835700	-0.61047600	0.26428800
C	4.86960400	-0.92706800	1.34668900
C	3.79216700	-1.78660300	1.17645800
H	4.10685300	-2.41013100	-2.13864100
H	6.04678000	-0.90552100	-1.83494500

H	6.53092600	0.06000300	0.39697800
H	5.07581600	-0.50586300	2.32526900
H	3.15507000	-2.03370000	2.02058400
C	2.54597200	1.16174900	-1.47671300
C	2.05078500	2.38670400	-0.75944800
H	2.86010700	1.40317500	-2.49392800
H	3.39993600	0.72856000	-0.94284400
C	1.21203700	3.29463200	-1.40852300
C	0.66821600	4.37054100	-0.71835100
C	0.96034600	4.55262500	0.63178100
C	1.80742800	3.66264000	1.28262300
C	2.35099700	2.58637300	0.58707900
H	0.97432800	3.14606000	-2.45792900
H	0.01496300	5.06870600	-1.23135800
H	0.53239700	5.39061500	1.17198200
H	3.00981500	1.88820500	1.09661600
H	2.04383900	3.80417900	2.33207600
O	-2.80938300	0.76632900	1.42659800
H	-1.27721400	1.67389000	2.40557600
H	-1.85584000	0.16073200	3.16649000
C	-3.59018100	-0.23803300	1.38060100
C	-4.62302100	-0.30949100	0.42220500
H	-3.45484900	-1.03466700	2.11127200
C	-5.49706800	-1.41154800	0.48418800
C	-6.51326800	-1.52630400	-0.44508500
C	-6.65377200	-0.55201300	-1.43143800
C	-5.78493500	0.54205000	-1.50154900
C	-4.76750700	0.66980700	-0.58159400
H	-5.36693900	-2.15958300	1.25885200
H	-7.19392200	-2.36813400	-0.40904000
H	-7.45021900	-0.64507100	-2.16197500
H	-5.91078500	1.28325500	-2.28160200
H	-4.07652600	1.50319000	-0.62223900

Gal-4B_DMSO

Charge=+1, Multiplicity=+1

O	-1.25141600	-0.34044100	-0.74328300
C	-0.95422400	-1.60789800	-0.23019100

C	-0.26235000	0.36083200	0.03050200
O	-1.09567400	-1.45310200	1.18122100
C	1.03874600	-0.32116800	-0.40771700
C	0.52959300	-1.77692300	-0.58505000
H	1.80479000	-0.27547400	0.37108600
O	1.53670900	0.16501500	-1.62886400
O	1.11996900	-2.74583000	0.23140900
H	0.61448400	-2.04627700	-1.64584900
H	-1.64176200	-2.37170300	-0.58370400
C	-0.58033400	-0.14809200	1.44803800
C	-1.60961900	0.64071700	2.22737100
H	0.32029100	-0.21026400	2.06779600
H	-0.33415700	1.43274600	-0.12652800
C	2.35177500	-3.25982400	-0.25597800
C	3.51819400	-2.32130200	-0.08130500
H	2.24888300	-3.53668700	-1.31284900
H	2.51994700	-4.17507700	0.31775400
C	4.33525900	-1.99277600	-1.15912500
C	5.42286200	-1.13906300	-0.99017900
C	5.69250700	-0.59529900	0.25997300
C	4.87554900	-0.91430300	1.34303400
C	3.80095600	-1.77760400	1.17383200
H	4.11541500	-2.40104200	-2.14132600
H	6.05039300	-0.88964500	-1.83948000
H	6.53282800	0.07816300	0.39187200
H	5.08078100	-0.49186400	2.32128500
H	3.16518600	-2.02637100	2.01846600
C	2.54248000	1.16739500	-1.47428900
C	2.04225400	2.39037200	-0.75717000
H	2.85614600	1.41013200	-2.49134600
H	3.39785200	0.73740700	-0.94008300
C	1.20056000	3.29525300	-1.40670800
C	0.65183800	4.36875100	-0.71663800
C	0.94194300	4.55141700	0.63386000
C	1.79195100	3.66454200	1.28514400
C	2.34049500	2.59074800	0.58969300
H	0.96434600	3.14611500	-2.45638100
H	-0.00385300	5.06441700	-1.22993800

H	0.50987700	5.38727900	1.17408600
H	3.00128300	1.89486300	1.09977000
H	2.02649600	3.80633400	2.33497900
O	-2.80897300	0.75553700	1.42807200
H	-1.27924900	1.66051000	2.41312200
H	-1.85758100	0.14378700	3.16703200
C	-3.59035100	-0.24802600	1.37810500
C	-4.62410100	-0.31437200	0.42026400
H	-3.45464500	-1.04831700	2.10471900
C	-5.49744600	-1.41723500	0.47687200
C	-6.51458200	-1.52730300	-0.45197300
C	-6.65678100	-0.54740600	-1.43248300
C	-5.78874600	0.54766800	-1.49713100
C	-4.77038500	0.67074300	-0.57756300
H	-5.36596500	-2.16954300	1.24713700
H	-7.19466200	-2.36976200	-0.42002700
H	-7.45399000	-0.63668800	-2.16266100
H	-5.91608100	1.29341300	-2.27260400
H	-4.08024900	1.50504500	-0.61388200

Gal-4B_Water

Charge=+1, Multiplicity=+1

O	-1.24887800	-0.35271600	-0.74201300
C	-0.94555000	-1.61948800	-0.23045100
C	-0.26298700	0.35230600	0.03251800
O	-1.08756100	-1.46724400	1.18088400
C	1.04109800	-0.32275300	-0.40731300
C	0.53907900	-1.78079400	-0.58588300
H	1.80748700	-0.27393700	0.37087800
O	1.53581900	0.16710600	-1.62848400
O	1.13456800	-2.74743600	0.22970700
H	0.62519500	-2.04902600	-1.64685600
H	-1.62935700	-2.38611000	-0.58507900
C	-0.57795200	-0.16019200	1.44953600
C	-1.60978700	0.62298900	2.23075400
H	0.32330300	-0.21926400	2.06865200
H	-0.34017500	1.42408500	-0.12286900
C	2.36867500	-3.25504800	-0.25845000

C	3.53065500	-2.31095100	-0.08386800
H	2.26672200	-3.53190700	-1.31540700
H	2.54165100	-4.16977200	0.31471400
C	4.34669100	-1.97963000	-1.16164600
C	5.43031900	-1.12079800	-0.99299900
C	5.69695900	-0.57473900	0.25682200
C	4.88097600	-0.89651200	1.33981000
C	3.81040500	-1.76487200	1.17092500
H	4.12910200	-2.38974400	-2.14358900
H	6.05699000	-0.86916200	-1.84228500
H	6.53402600	0.10281900	0.38846700
H	5.08357600	-0.47201500	2.31771100
H	3.17523500	-2.01541800	2.01547300
C	2.53735500	1.17373800	-1.47364700
C	2.03207100	2.39387800	-0.75528900
H	2.84942300	1.41865600	-2.49067900
H	3.39482700	0.74701800	-0.94018400
C	1.18655100	3.29586100	-1.40391400
C	0.63295900	4.36608200	-0.71262200
C	0.92192400	4.54826100	0.63819200
C	1.77566700	3.66430300	1.28855900
C	2.32918700	2.59388100	0.59187900
H	0.95111900	3.14695000	-2.45380200
H	-0.02588900	5.05934800	-1.22512800
H	0.48571000	5.38126300	1.17950000
H	2.99248300	1.90003200	1.10144000
H	2.00889800	3.80543300	2.33876800
O	-2.80815900	0.74126400	1.43010300
H	-1.28098400	1.64216900	2.42258700
H	-1.85894300	0.12079700	3.16725700
C	-3.59036300	-0.26115800	1.37463900
C	-4.62594600	-0.31993100	0.41816600
H	-3.45362400	-1.06691200	2.09500000
C	-5.49841600	-1.42376600	0.46731000
C	-6.51754500	-1.52665700	-0.46023100
C	-6.66263700	-0.53851700	-1.43195500
C	-5.79556400	0.55777400	-1.48904400
C	-4.77524300	0.67371500	-0.57069900

H	-5.36466900	-2.18242300	1.23089600
H	-7.19694000	-2.36985800	-0.43391400
H	-7.46145300	-0.62206000	-2.16105200
H	-5.92534700	1.31020000	-2.25762600
H	-4.08618100	1.50916100	-0.60108100

Gal-6B_Dioxane

Charge=+1, Multiplicity=+1

C	-0.75659500	-2.34836600	-0.36876400
O	-2.11358600	-2.10510300	-0.70944800
C	-2.50741000	-1.16159600	0.26339600
C	-0.04203500	-1.13085100	-0.96825600
H	-0.42732100	-3.26990600	-0.85081600
C	-0.86376700	-2.46066500	1.15486400
C	-0.41158400	0.16919600	-0.26582500
H	-0.36664900	-1.07474900	-2.00623000
O	1.40291300	-1.20198300	-1.08522700
C	-1.94586400	0.22268500	-0.09522500
O	0.27713800	0.20917300	0.95883100
H	-0.11850300	1.00674100	-0.90662200
O	-2.47682400	0.67259400	-1.31635200
H	-2.16643400	0.91754100	0.72500900
O	-1.95533200	-1.61091300	1.47920400
H	-3.59528000	-1.15418300	0.31652500
H	0.01276300	-2.13442400	1.71436600
H	-1.10816700	-3.49103600	1.42833900
C	2.15161200	-1.39895900	-0.07280800
C	3.55368700	-1.27157500	-0.18029500
H	1.70314200	-1.71142000	0.86433800
C	4.16487500	-0.77480300	-1.34822400
C	5.53891600	-0.67915700	-1.39900600
C	6.30468700	-1.07283500	-0.29906700
C	5.70681400	-1.55871700	0.86311300
C	4.33139900	-1.65463600	0.92981700
H	3.55104600	-0.46714700	-2.18609100
H	6.02577400	-0.29505100	-2.28747300
H	7.38557500	-0.99467700	-0.34935000
H	6.31767900	-1.85645000	1.70686800

H	3.84743200	-2.02415600	1.82828700
C	0.63524700	1.52827000	1.40196600
C	1.95598100	1.95472300	0.83303000
H	0.68237300	1.46300900	2.48978500
H	-0.16215200	2.23108200	1.13746200
C	3.13388000	1.63990800	1.51173700
C	4.37145300	1.96377600	0.97167500
C	4.44412100	2.60282100	-0.26137000
C	3.27666400	2.93493700	-0.94043800
C	2.03905700	2.62046100	-0.39096300
H	3.07724800	1.14423000	2.47683000
H	5.27934000	1.72108600	1.51349000
H	5.40962300	2.85797700	-0.68491300
H	3.32904900	3.45479700	-1.89112800
H	1.13225700	2.91817000	-0.90968700
C	-3.53763800	1.62210400	-1.20205500
C	-4.77028100	1.05204600	-0.56025400
H	-3.18695700	2.49814000	-0.64016800
H	-3.73910700	1.93272500	-2.22897800
C	-5.53222800	0.09780700	-1.23766300
C	-6.63404900	-0.48443800	-0.62693500
C	-6.98860800	-0.11742300	0.66920700
C	-6.24189500	0.83784800	1.34706700
C	-5.13699400	1.41925700	0.73220100
H	-4.55553300	2.16762400	1.26354600
H	-7.84980600	-0.57294800	1.14622400
H	-6.51717700	1.13073600	2.35458000
H	-5.25074800	-0.19425800	-2.24498300
H	-7.22060800	-1.22451500	-1.16083400

Gal-6B_Toluene

Charge=+1, Multiplicity=+1

C	-2.04174500	-2.25030600	-1.34521700
O	-3.35430000	-1.72453300	-1.47097400
C	-3.76248300	-1.62596800	-0.12442000
C	-1.20556300	-1.04039100	-0.94520300
H	-1.72086000	-2.65131500	-2.30679800
C	-2.23159200	-3.31169000	-0.26562200

C	-1.57089300	-0.49529800	0.42917500
H	-1.37675900	-0.26597700	-1.69589600
O	0.21179800	-1.38038800	-0.98199200
C	-3.11229500	-0.39342600	0.51794000
O	-1.05520000	-1.34422700	1.42253200
H	-1.14196200	0.50906900	0.52579700
O	-3.63431000	0.71079600	-0.17784600
H	-3.38312700	-0.35752000	1.58215600
O	-3.32800600	-2.80615300	0.49875300
H	-4.85004100	-1.55108900	-0.09640200
H	-1.36133100	-3.43958100	0.37783200
H	-2.51846200	-4.26583700	-0.71420400
C	1.04227700	-0.42094300	-1.09348200
C	2.42806100	-0.64633900	-1.17348000
H	0.64923900	0.59744100	-1.16658200
C	2.98464600	-1.93694500	-1.05207400
C	4.34666200	-2.09464400	-1.17680100
C	5.15693100	-0.98056100	-1.42284700
C	4.61659500	0.29805400	-1.53545500
C	3.25252400	0.47258200	-1.40271000
H	2.33703300	-2.78527300	-0.86432200
H	4.79251500	-3.07811800	-1.08746400
H	6.22869800	-1.11596900	-1.52391600
H	5.26148900	1.14870300	-1.71998700
H	2.80762100	1.45910800	-1.48121200
C	-0.48904200	-0.67159000	2.54518300
C	0.91359600	-0.19792900	2.28058400
H	-0.49740900	-1.40765100	3.35085400
H	-1.12858000	0.16586500	2.84951200
C	1.17862500	1.14065000	1.98841600
C	2.48111400	1.57479500	1.76284600
C	3.53371300	0.67069700	1.83122000
C	3.27931000	-0.66994100	2.11327700
C	1.97788800	-1.10041800	2.33362200
H	0.36284000	1.85593400	1.95846200
H	2.67113600	2.62128000	1.54756500
H	4.55244400	1.00805700	1.67345100
H	4.10060500	-1.37580300	2.17536600

H	1.78293100	-2.14315300	2.56674400
C	-3.36545300	1.98579900	0.37449800
C	-2.11270100	2.62035500	-0.18038100
H	-4.23144200	2.60580200	0.12279000
H	-3.31122000	1.93069700	1.46943400
C	-1.74486100	2.40913000	-1.51043000
C	-0.59004300	2.99087900	-2.02255600
C	0.20254300	3.80356100	-1.21546700
C	-0.17320700	4.03875500	0.10195500
C	-1.32267400	3.44698000	0.61588600
H	-1.60704200	3.62760500	1.64937700
H	1.09644300	4.26786700	-1.61846300
H	0.43239700	4.67890900	0.73519900
H	-2.37748400	1.79304300	-2.14197700
H	-0.32112500	2.82886600	-3.06185200

Gal-6B_Et_2O

Charge=+1, Multiplicity=+1

C	0.80119800	3.26413800	-0.53396800
O	2.16474100	3.33667700	-0.91922700
C	2.81398200	2.75663100	0.19062600
C	0.40802000	1.81207000	-0.78384200
H	0.21467000	3.93942700	-1.15651600
C	0.86155000	3.67551400	0.93436700
C	1.17390500	0.82663100	0.10041700
H	0.59925000	1.59162000	-1.83656500
O	-1.02085200	1.67933700	-0.54189300
C	2.66391100	1.23324600	0.12683600
O	0.61836500	0.83889800	1.39119300
H	1.10930900	-0.17269200	-0.34561400
O	3.31819600	0.80270200	-1.04375600
H	3.10938100	0.79508400	1.02732900
O	2.17893400	3.28444700	1.32822300
H	3.86524800	3.04676400	0.16897900
H	0.12325000	3.16828200	1.55556300
H	0.76560800	4.75906300	1.03523800
C	-1.66957700	0.75810900	-1.13341100
C	-3.05317600	0.59558100	-0.94117300

H	-1.13257200	0.10969400	-1.82768900
C	-3.77400000	1.39134600	-0.02570800
C	-5.13259100	1.20723500	0.09860500
C	-5.77789800	0.23835300	-0.67771200
C	-5.07231400	-0.55757900	-1.57674400
C	-3.70738800	-0.38845500	-1.70760000
H	-3.25233700	2.13389100	0.56629000
H	-5.70277700	1.80922500	0.79580100
H	-6.84945400	0.10374100	-0.57476400
H	-5.58873900	-1.30635900	-2.16485200
H	-3.13719900	-0.99956200	-2.39884400
C	0.46956800	-0.45145700	1.98555800
C	-0.78897700	-1.14177900	1.53792300
H	0.43984100	-0.26779300	3.06096900
H	1.34797700	-1.06981600	1.76912500
C	-2.01129500	-0.81861600	2.12987300
C	-3.17822000	-1.46283700	1.74010800
C	-3.13769400	-2.44013500	0.74831500
C	-1.92810100	-2.76326800	0.14531700
C	-0.76019600	-2.11578700	0.53758000
H	-2.04244000	-0.06730200	2.91349700
H	-4.11999800	-1.21180300	2.21623900
H	-4.04739900	-2.95185600	0.45337300
H	-1.88846100	-3.53094400	-0.62063400
H	0.18709700	-2.39987100	0.08955100
C	4.21670900	-0.28708600	-0.86548200
C	3.52769400	-1.60357700	-0.62404200
H	4.79336900	-0.32755500	-1.79217000
H	4.90996900	-0.06709100	-0.04438200
C	3.63727000	-2.25786200	0.60103600
C	3.01353200	-3.48576500	0.80861600
C	2.27500100	-4.07053800	-0.21221800
C	2.15515500	-3.42130900	-1.43955400
C	2.77557500	-2.19566500	-1.64184400
H	2.69329900	-1.69610500	-2.60293400
H	1.79449000	-5.03034500	-0.05523600
H	1.58605400	-3.87934300	-2.24208300
H	4.22774900	-1.81175200	1.39637400

H	3.10988200	-3.98593000	1.76636200

Gal-6B_$CHCl_3$

Charge=+1, Multiplicity=+1

C	-0.80052900	-3.26147700	-0.53406400
O	-2.16423100	-3.33557000	-0.91928600
C	-2.81407100	-2.75625100	0.19059200
C	-0.40859000	-1.80897300	-0.78324400
H	-0.21324900	-3.93589800	-1.15676700
C	-0.86042000	-3.67334700	0.93406800
C	-1.17611800	-0.82443900	0.10066300
H	-0.59929400	-1.58838100	-1.83599400
O	1.01953200	-1.67520500	-0.53991700
C	-2.66565100	-1.23273300	0.12687800
O	-0.62192500	-0.83575700	1.39216600
H	-1.11207900	0.17476200	-0.34554300
O	-3.32108500	-0.80326600	-1.04359000
H	-3.11171700	-0.79502700	1.02727400
O	-2.17847300	-3.28392700	1.32810300
H	-3.86506800	-3.04738700	0.16905500
H	-0.12268300	-3.16537800	1.55529900
H	-0.76314200	-4.75677200	1.03471300
C	1.66965000	-0.75774100	-1.13590800
C	3.05312000	-0.59553700	-0.94269400
H	1.13388200	-0.11273300	-1.83427200
C	3.77218700	-1.38794000	-0.02296600
C	5.13078100	-1.20424000	0.10233400
C	5.77767800	-0.23897400	-0.67706000
C	5.07378600	0.55364000	-1.58042600
C	3.70898300	0.38477000	-1.71251600
H	3.24937200	-2.12765300	0.57153700
H	5.69956400	-1.80355600	0.80296600
H	6.84912400	-0.10441300	-0.57307400
H	5.59145000	1.29971300	-2.17085700
H	3.14007500	0.99330800	-2.40705600
C	-0.47136300	0.45522600	1.98500100
C	0.78921300	1.14246900	1.53842000
H	-0.44355400	0.27291200	3.06069600

H	-1.34830900	1.07491000	1.76646100
C	0.76410500	2.11445500	0.53602400
C	1.93404200	2.75837400	0.14394400
C	3.14205000	2.43382000	0.74938800
C	3.17888300	1.45861600	1.74338300
C	2.00989700	0.81784600	2.13288400
H	-0.18185100	2.39932700	0.08569000
H	1.89735300	3.52387500	-0.62432300
H	4.05345000	2.94232900	0.45412800
H	4.11944000	1.20613500	2.22115400
H	2.03835100	0.06780600	2.91783300
C	-4.21885000	0.28719000	-0.86529200
C	-3.52891900	1.60328200	-0.62430000
H	-4.79579100	0.32785700	-1.79180400
H	-4.91184900	0.06780700	-0.04384000
C	-3.63894700	2.25837400	0.60030000
C	-3.01385500	3.48564600	0.80777500
C	-2.27351200	4.06884500	-0.21266100
C	-2.15320700	3.41872600	-1.43952300
C	-2.77503400	2.19378500	-1.64173900
H	-2.69177900	1.69328200	-2.60224300
H	-1.79147000	5.02789100	-0.05564600
H	-1.58211600	3.87520900	-2.24151500
H	-4.23026400	1.81307600	1.39546900
H	-3.11025000	3.98631300	1.76526100

Gal-6B_DCM

Charge=+1, Multiplicity=+1

C	-0.75842800	-3.25439400	-0.49235000
O	-2.11684700	-3.35592200	-0.89372700
C	-2.78905800	-2.76171700	0.19475700
C	-0.38175400	-1.80184800	-0.76444400
H	-0.15349500	-3.93261300	-1.09322200
C	-0.83257900	-3.63798500	0.98243300
C	-1.17490700	-0.81043300	0.08865500
H	-0.56043300	-1.60415800	-1.82368000
O	1.03879700	-1.64513900	-0.50285200
C	-2.65877900	-1.23836200	0.10142000

O	-0.64424600	-0.78609200	1.39035100
H	-1.11629300	0.17959900	-0.37716400
O	-3.30157100	-0.84080600	-1.08820000
H	-3.12416600	-0.78713500	0.98485000
O	-2.16230600	-3.25935300	1.35139100
H	-3.83603400	-3.06657500	0.16586600
H	-0.11015700	-3.10748800	1.60267700
H	-0.72182700	-4.71755300	1.10634200
C	1.69718500	-0.76436200	-1.14404000
C	3.07768000	-0.59079000	-0.93928700
H	1.17081400	-0.16291100	-1.88716700
C	3.78318300	-1.33358600	0.03075000
C	5.14014600	-1.14235200	0.16601900
C	5.79747400	-0.21712000	-0.65147300
C	5.10631900	0.52753700	-1.60474300
C	3.74424400	0.34932800	-1.74913500
H	3.25251900	-2.04260200	0.65486600
H	5.69851300	-1.70356500	0.90553100
H	6.86690200	-0.07500500	-0.53793800
H	5.63205900	1.24383600	-2.22412700
H	3.18478700	0.92052100	-2.48200500
C	-0.49171600	0.52168600	1.94615600
C	0.78397200	1.18383200	1.50474300
H	-0.48605700	0.37200300	3.02723300
H	-1.35888100	1.14132100	1.69208000
C	0.79069900	2.11780100	0.46649900
C	1.97548100	2.73484100	0.07556900
C	3.16687700	2.42213300	0.71903900
C	3.17188100	1.48513600	1.74966600
C	1.98796300	0.87043100	2.13749700
H	-0.14241600	2.39261900	-0.01574500
H	1.96374800	3.46754200	-0.72478300
H	4.09027600	2.90758000	0.42228900
H	4.09945600	1.24024000	2.25588000
H	1.99265200	0.14850800	2.94889800
C	-4.22895800	0.22995100	-0.93833400
C	-3.57494900	1.55513800	-0.65306100
H	-4.76638900	0.26855700	-1.88820200

H	-4.95052400	-0.01218500	-0.14862900
C	-3.73052500	2.17857400	0.58310700
C	-3.12751300	3.40772400	0.83949000
C	-2.36419400	4.02448300	-0.14372700
C	-2.20125100	3.40767700	-1.38291200
C	-2.80104500	2.18068700	-1.63390400
H	-2.67996800	1.70455500	-2.60272700
H	-1.89613800	4.98344900	0.05179300
H	-1.61078300	3.88983700	-2.15530600
H	-4.33623200	1.70378500	1.34999400
H	-3.25725300	3.88226600	1.80635800

Gal-6B_ACN

Charge=+1, Multiplicity=+1

C	-0.78139600	-3.22385900	-0.54405900
O	-2.14628200	-3.32162000	-0.92713900
C	-2.80452100	-2.76293200	0.18835300
C	-0.41313700	-1.76176400	-0.77368600
H	-0.18202800	-3.87930800	-1.17482800
C	-0.83066500	-3.65277200	0.91879400
C	-1.20361400	-0.79985100	0.11546400
H	-0.59874000	-1.52983100	-1.82467200
O	1.00640100	-1.61064100	-0.51372100
C	-2.68490700	-1.23685800	0.13529700
O	-0.66437500	-0.80744700	1.41482200
H	-1.15261600	0.20198800	-0.32361500
O	-3.35334600	-0.81825800	-1.03299600
H	-3.14211800	-0.81208800	1.03615800
O	-2.15722000	-3.29192200	1.31997700
H	-3.85038900	-3.07219800	0.16732900
H	-0.10069300	-3.13769200	1.54305300
H	-0.71286500	-4.73484800	1.00864000
C	1.67609100	-0.75046100	-1.17106300
C	3.05825200	-0.59138200	-0.96444900
H	1.15800600	-0.15460500	-1.92404100
C	3.75253300	-1.33762900	0.01085500
C	5.11087000	-1.15854100	0.15129400
C	5.77951800	-0.24055100	-0.66476100

C	5.09901900	0.50740800	-1.62351900
C	3.73658600	0.33942000	-1.77529900
H	3.21291800	-2.04045700	0.63430000
H	5.66078700	-1.72289100	0.89471200
H	6.84934300	-0.10622100	-0.54590400
H	5.63369800	1.21828100	-2.24150200
H	3.18539800	0.91348300	-2.51209300
C	-0.49387800	0.48765500	1.99530300
C	0.78260600	1.14841000	1.55466600
H	-0.47918400	0.31610800	3.07304700
H	-1.35825500	1.11925400	1.76153400
C	0.78798200	2.08923900	0.52283900
C	1.97397900	2.70027200	0.12632500
C	3.16803500	2.37599700	0.75933200
C	3.17412700	1.43274100	1.78411600
C	1.98883200	0.82284700	2.17667200
H	-0.14549100	2.37069300	0.04561100
H	1.96145000	3.43424900	-0.67277300
H	4.09302500	2.85461000	0.45643200
H	4.10405700	1.17646300	2.28028800
H	1.99541900	0.09350900	2.98145600
C	-4.24220400	0.28002000	-0.85578300
C	-3.54239000	1.59330800	-0.62813600
H	-4.82481700	0.31856200	-1.77896400
H	-4.93020900	0.07066100	-0.02773700
C	-2.76823300	2.15928300	-1.64444900
C	-2.13205800	3.37832500	-1.45098200
C	-2.26027200	4.04768800	-0.23502300
C	-3.02186300	3.48910900	0.78386500
C	-3.65973900	2.26654500	0.58594200
H	-4.26114100	1.83589400	1.38155200
H	-1.76312000	5.00010000	-0.08403200
H	-3.12117900	4.00270100	1.73429500
H	-2.67203200	1.64116600	-2.59419900
H	-1.53798100	3.81152400	-2.24905300

Gal-6B_DMSO

Charge=+1, Multiplicity=+1

C	-0.76960700	-3.22297700	-0.52971600
O	-2.13256800	-3.32854200	-0.91818600
C	-2.79781500	-2.76434200	0.19026600
C	-0.40569400	-1.76100200	-0.76733000
H	-0.16491900	-3.88034100	-1.15334000
C	-0.82392200	-3.64206600	0.93570900
C	-1.20350100	-0.79613700	0.11211200
H	-0.58783400	-1.53639900	-1.82051400
O	1.01189500	-1.60313400	-0.50226300
C	-2.68326300	-1.23833200	0.12734600
O	-0.67117300	-0.79230900	1.41440500
H	-1.15437200	0.20288000	-0.33338600
O	-3.34592100	-0.82935500	-1.04779700
H	-3.14673600	-0.80888400	1.02270200
O	-2.15347900	-3.28295000	1.32860500
H	-3.84243000	-3.07766200	0.16724300
H	-0.09837600	-3.12030600	1.55959300
H	-0.70284300	-4.72310600	1.03337700
C	1.68367900	-0.75294500	-1.17043100
C	3.06536300	-0.59119400	-0.96225600
H	1.16771600	-0.16792600	-1.93325900
C	3.75732500	-1.32600600	0.02322900
C	5.11537300	-1.14502500	0.16497000
C	5.78572500	-0.23601400	-0.65948100
C	5.10736200	0.50097200	-1.62832900
C	3.74550500	0.33059500	-1.78189600
H	3.21639200	-2.02208200	0.65304200
H	5.66357500	-1.70088700	0.91599700
H	6.85519000	-0.09996000	-0.53941100
H	5.64341300	1.20506600	-2.25283700
H	3.19594900	0.89619700	-2.52639200
C	-0.49971200	0.50790700	1.98319600
C	0.78097500	1.16113900	1.54341900
H	-0.49108400	0.34668200	3.06262100
H	-1.36109600	1.13949400	1.73886300
C	0.79491000	2.09166900	0.50234400
C	1.98474600	2.69621600	0.10730000
C	3.17408500	2.37593700	0.75110300

C	3.17163800	1.44284600	1.78512800
C	1.98255300	0.83926500	2.17616100
H	-0.13495500	2.37013700	0.01638200
H	1.97886800	3.42181100	-0.69947100
H	4.10201200	2.84935700	0.44908100
H	4.09781700	1.18949800	2.28973300
H	1.98259900	0.11789700	2.98811300
C	-4.24431400	0.26289500	-0.88096600
C	-3.55514200	1.57900600	-0.63839800
H	-4.81312300	0.30057000	-1.81271000
H	-4.94316600	0.04671500	-0.06382700
C	-2.77435700	2.15620400	-1.64322400
C	-2.14449200	3.37592900	-1.43409900
C	-2.28539800	4.03450300	-0.21367800
C	-3.05433600	3.46509800	0.79364900
C	-3.68617400	2.24209000	0.57988500
H	-4.29283900	1.80268400	1.36666600
H	-1.79280700	4.98726100	-0.05046800
H	-3.16406800	3.97051900	1.74730200
H	-2.66718300	1.64601200	-2.59607800
H	-1.54480600	3.81771200	-2.22321400

Gal-6B_Water

Charge=+1, Multiplicity=+1

C	0.78662200	3.21375200	-0.55667700
O	2.15282700	3.31180200	-0.93560800
C	2.80835300	2.76297200	0.18632300
C	0.42144200	1.74906700	-0.77491600
H	0.18792100	3.86262700	-1.19479400
C	0.82977500	3.65445300	0.90275600
C	1.21226300	0.79588600	0.12343500
H	0.60846100	1.50797000	-1.82361500
O	-0.99762700	1.59855800	-0.51445900
C	2.69254700	1.23635700	0.14361300
O	0.67155100	0.81146300	1.42236000
H	1.16409900	-0.20914200	-0.30794800
O	3.36614700	0.81317300	-1.02010600
H	3.14893400	0.81828200	1.04808800

O	2.15569200	3.29904200	1.31171100
H	3.85358200	3.07449300	0.16689200
H	0.09828600	3.14315300	1.52833400
H	0.70977300	4.73697800	0.98361200
C	-1.67134300	0.74672900	-1.17851000
C	-3.05388400	0.59161100	-0.97081300
H	-1.15658000	0.15516400	-1.93704300
C	-3.74406000	1.33621200	0.00857300
C	-5.10276600	1.16071100	0.15080000
C	-5.77546200	0.24740400	-0.66701900
C	-5.09888100	-0.49898300	-1.62989700
C	-3.73642200	-0.33390400	-1.78418300
H	-3.20122800	2.03532600	0.63339500
H	-5.64962600	1.72403200	0.89724200
H	-6.84538100	0.11542800	-0.54641300
H	-5.63675600	-1.20626300	-2.24922600
H	-3.18823400	-0.90688600	-2.52404700
C	0.49519500	-0.48056300	2.00809200
C	-0.78193800	-1.13973600	1.56705800
H	0.47821300	-0.30424000	3.08503200
H	1.35835600	-1.11567100	1.77921400
C	-1.98805300	-0.81291600	2.18847800
C	-3.17410800	-1.42054200	1.79427000
C	-3.16882500	-2.36248500	0.76834200
C	-1.97476900	-2.68779800	0.13578800
C	-0.78808100	-2.07934400	0.53414800
H	-1.99426000	-0.08407700	2.99372800
H	-4.10404900	-1.16294300	2.28973600
H	-4.09448500	-2.83862500	0.46358900
H	-1.96281300	-3.41987900	-0.66504100
H	0.14498000	-2.36124000	0.05647200
C	4.24475000	-0.29230100	-0.83791800
C	3.53385600	-1.60196000	-0.62355100
H	4.83697200	-0.33138400	-1.75501600
H	4.92541400	-0.09140600	-0.00175800
C	3.64377100	-2.28694100	0.58462900
C	2.99761400	-3.50722200	0.76963100
C	2.23470900	-4.05128000	-0.25615100

C	2.11306700	-3.36945900	-1.46588900
C	2.75792900	-2.15304000	-1.64671900
H	2.66614500	-1.62495800	-2.59137500
H	1.73060000	-5.00156900	-0.11507400
H	1.51675100	-3.79049300	-2.26875700
H	4.24534200	-1.86699700	1.38581000
H	3.09105000	-4.03005600	1.71562200

Man-4B_Dioxane

Charge=+1, Multiplicity=+1

C	1.50139500	-0.95744600	-0.49989200
C	2.47806100	-1.33050000	0.62947300
C	1.95994600	-0.89052500	2.00164900
O	0.79144100	-1.69817900	2.14637600
H	2.70808600	-1.18535400	2.74948400
C	1.65178400	0.55267000	2.31385700
C	0.87840900	-2.62880300	1.05725400
C	0.36064100	-1.96431900	-0.21733200
O	-0.90875100	-1.40628400	-0.03579200
H	0.35047200	-2.70906700	-1.02309600
O	2.03309300	-1.17260700	-1.78095500
H	1.13350100	0.06893500	-0.41500700
O	2.24278800	-2.74203300	0.78370100
H	3.52819300	-1.13123200	0.43909200
H	0.43230100	-3.57687800	1.34542700
O	0.55813500	1.10699700	1.53622100
H	2.50474800	1.18937500	2.08129500
H	1.39038600	0.66031400	3.36845100
C	-0.64412400	0.79085900	1.83642800
C	-1.72943500	1.40759800	1.18741900
H	-0.80037700	0.07190200	2.63939100
C	-3.02738800	1.09027200	1.63528100
C	-4.11743100	1.72681100	1.07514200
C	-3.91871300	2.65353700	0.05671400
C	-2.63348900	2.96751500	-0.40260000
C	-1.53722100	2.35408200	0.15757000
H	-3.16421400	0.35238600	2.41848100
H	-5.11935200	1.48961000	1.40917600

H	-4.77626800	3.14430600	-0.39095800
H	-2.50650400	3.69505100	-1.19561600
H	-0.53307700	2.59264000	-0.17449800
C	-1.52834300	-1.03680500	-1.25488100
C	-3.00912900	-0.88841000	-1.06511400
H	-1.32986000	-1.81258900	-2.00857700
H	-1.09724800	-0.10080800	-1.63811000
C	-3.68559100	-1.61436300	-0.08621900
C	-5.06704000	-1.51156000	0.03492600
C	-5.78580100	-0.68683600	-0.82329300
C	-5.11494800	0.03735900	-1.80324900
C	-3.73314500	-0.06049600	-1.91920300
H	-3.12237300	-2.26054900	0.57775200
H	-5.58408800	-2.08357300	0.79850600
H	-6.86424200	-0.61197700	-0.73281200
H	-5.66868300	0.68159200	-2.47829900
H	-3.21345000	0.51332100	-2.68146600
C	2.56849900	0.00485900	-2.38005000
C	3.67874500	0.62236900	-1.57512600
H	1.76004300	0.73591000	-2.52664300
H	2.92416700	-0.31420700	-3.36099600
C	3.44891200	1.75722300	-0.79969900
C	4.44945400	2.27436800	0.01841400
C	5.69316300	1.65540500	0.06576800
C	5.93669700	0.52919400	-0.71657400
C	4.93633700	0.01840900	-1.53297100
H	2.47809200	2.24476700	-0.84122200
H	4.26252000	3.16513300	0.60957700
H	6.47709800	2.05633400	0.69931600
H	6.91038900	0.05181300	-0.69160300
H	5.12462300	-0.86511300	-2.13559400

Man-4B_Toluene

Charge=+1, Multiplicity=+1

C	-1.64864800	-0.67720700	1.08582900
C	-3.16265000	-0.82646000	0.99923300
C	-3.73310900	-0.07852200	-0.21521300
O	-3.20375300	-0.83473600	-1.30383500

H	-4.82598600	-0.18152000	-0.19373800
C	-3.48956800	1.39669500	-0.42917700
C	-2.58708300	-1.96150700	-0.67304800
C	-1.21100200	-1.52536100	-0.13188400
O	-0.45133100	-0.76454200	-1.03087400
H	-0.65971600	-2.40589500	0.21490300
O	-1.22036400	-1.18082800	2.32256200
H	-1.30945100	0.35363600	0.96411900
O	-3.31719000	-2.16553500	0.50045100
H	-3.70392100	-0.69460600	1.93263000
H	-2.63180900	-2.82260200	-1.33503400
O	-2.09082500	1.74865300	-0.59343800
H	-3.82053000	1.97864200	0.42968800
H	-4.02148400	1.73319800	-1.32096100
C	-1.52003100	1.55025400	-1.71595600
C	-0.17893900	1.94374300	-1.92345300
H	-2.11176000	1.12826100	-2.52784200
C	0.62916300	2.41436500	-0.87034800
C	1.93119400	2.77921000	-1.14002100
C	2.43203100	2.67844200	-2.44080300
C	1.63870100	2.20786900	-3.48532400
C	0.33251500	1.83484900	-3.23023400
H	0.23704400	2.47457400	0.13919500
H	2.56553200	3.13853800	-0.33902600
H	3.45804400	2.96930100	-2.64000300
H	2.04256500	2.13476900	-4.48798100
H	-0.30216900	1.46685500	-4.03028600
C	0.37995300	-1.53904400	-1.89950600
C	1.56456800	-2.10139800	-1.16891500
H	0.70318800	-0.84241800	-2.67649300
H	-0.20409900	-2.33554900	-2.37543900
C	1.61992600	-3.44795700	-0.81487000
C	2.69590400	-3.94374100	-0.08483700
C	3.72963500	-3.09436800	0.28966100
C	3.68775600	-1.74985800	-0.07063200
C	2.60966200	-1.25645900	-0.79231900
H	0.81858900	-4.11686800	-1.11636300
H	2.72664300	-4.99303200	0.18793700

H	4.57148700	-3.47919100	0.85515500
H	4.50097800	-1.08820500	0.21027300
H	2.57281400	-0.20568700	-1.06419600
C	0.17218700	-1.03332900	2.53870600
C	0.62938400	0.40245300	2.61874100
H	0.74725200	-1.55833700	1.76360000
H	0.36486400	-1.54215400	3.48696100
C	-0.21702700	1.40620400	3.08882100
C	0.22840900	2.72021700	3.17927300
C	1.53449200	3.04269400	2.82212200
C	2.38803700	2.04283000	2.36819100
C	1.93196600	0.73449500	2.25266600
H	-1.22821300	1.15036200	3.38941100
H	-0.44000800	3.49255600	3.54565000
H	1.88618500	4.06546700	2.90731300
H	3.41284400	2.28159200	2.10161500
H	2.59428000	-0.04042500	1.87975900

Man-4B_Et2O

Charge=+1, Multiplicity=+1

C	2.42857300	-0.14638800	-0.63116000
C	3.56303100	-0.98245900	-0.04766300
C	3.11108500	-1.76797400	1.18915800
O	2.16333700	-2.67784500	0.62560000
H	3.97744900	-2.32800800	1.56436200
C	2.53779000	-1.08600900	2.40715200
C	2.31767100	-2.50658900	-0.78611100
C	1.52917800	-1.25609700	-1.22236600
O	0.22729700	-1.14493200	-0.72532500
H	1.53649800	-1.20794200	-2.31958900
O	2.96859400	0.73500200	-1.58187200
H	1.87509600	0.41286200	0.12617800
O	3.64071500	-2.08936700	-0.96434000
H	4.52187500	-0.47795200	0.03770200
H	2.11542300	-3.44449800	-1.29781200
O	1.30178800	-0.37156400	2.15792900
H	3.22396800	-0.33235800	2.79036400
H	2.34123500	-1.82540700	3.18571800

C	0.20870600	-1.03057300	2.09859200
C	-1.02546400	-0.37464700	1.94956000
H	0.25634100	-2.11232800	2.21908800
C	-1.11172300	1.02341400	1.78141300
C	-2.35363300	1.60846900	1.69309400
C	-3.50579100	0.81744600	1.76231500
C	-3.42829500	-0.56404800	1.91594500
C	-2.18956300	-1.16686200	2.00230700
H	-0.20990800	1.62233100	1.71785600
H	-2.43884800	2.68006300	1.55995300
H	-4.47941700	1.28979900	1.68767300
H	-4.33084800	-1.16096400	1.95073500
H	-2.10487900	-2.24273900	2.11642900
C	-0.68664700	-2.07411500	-1.28568300
C	-2.07488900	-1.50137700	-1.28123200
H	-0.65861400	-3.02017000	-0.72955900
H	-0.38800300	-2.29373600	-2.32095000
C	-2.27864800	-0.13342300	-1.44325700
C	-3.56779300	0.38039000	-1.50777700
C	-4.66647300	-0.46775800	-1.41405000
C	-4.46819100	-1.83439500	-1.24644500
C	-3.17841400	-2.34726100	-1.17589600
H	-1.42110100	0.52683600	-1.49860500
H	-3.71507800	1.44895900	-1.62913500
H	-5.67269400	-0.06552100	-1.46658000
H	-5.31959000	-2.50200100	-1.16503300
H	-3.02768600	-3.41450400	-1.03906800
C	2.00897500	1.59446700	-2.16916700
C	1.34544700	2.53616300	-1.19450300
H	1.23996000	1.01595000	-2.69907200
H	2.56259800	2.16105300	-2.92271000
C	0.03717800	2.95895100	-1.42058100
C	-0.57640600	3.86053400	-0.55814200
C	0.10802900	4.33491400	0.55551000
C	1.41279800	3.91146500	0.79255600
C	2.03026500	3.02415200	-0.08178000
H	-0.50432500	2.58757800	-2.28636000
H	-1.59196300	4.18860000	-0.75459900

H	-0.36963000	5.03425000	1.23370400
H	1.95511400	4.28267000	1.65608400
H	3.05239700	2.70445300	0.09655100

Man-4B_$CHCl_3$

Charge=+1, Multiplicity=+1

C	2.42877400	-0.14510200	-0.63058700
C	3.56307500	-0.98178800	-0.04791500
C	3.11114100	-1.76899400	1.18779700
O	2.16419800	-2.67904900	0.62273800
H	3.97766000	-2.32892100	1.56263700
C	2.53736300	-1.08875700	2.40647400
C	2.31775700	-2.50518000	-0.78841100
C	1.52890100	-1.25408800	-1.22232200
O	0.22756500	-1.14353300	-0.72376100
H	1.53495100	-1.20465500	-2.31948200
O	2.96889400	0.73652900	-1.58134400
H	1.87585800	0.41407800	0.12715100
O	3.64090300	-2.08732700	-0.96650800
H	4.52182400	-0.47734000	0.03869100
H	2.11527200	-3.44201700	-1.30195000
O	1.30150700	-0.37384500	2.15740900
H	3.22338500	-0.33564300	2.79092100
H	2.34040900	-1.82918300	3.18389100
C	0.20814600	-1.03220000	2.09915600
C	-1.02580000	-0.37549700	1.95030500
H	0.25498400	-2.11392300	2.22017200
C	-1.11129400	1.02256600	1.78223800
C	-2.35288100	1.60829700	1.69339600
C	-3.50547200	0.81784500	1.76207200
C	-3.42866700	-0.56365500	1.91576700
C	-2.19021300	-1.16712000	2.00277100
H	-0.20911800	1.62098000	1.71927400
H	-2.43760800	2.67992500	1.56026800
H	-4.47879800	1.29073100	1.68694100
H	-4.33150400	-1.16016900	1.95024100
H	-2.10593200	-2.24301300	2.11678200
C	-0.68612300	-2.07468200	-1.28139500

C	-2.07431500	-1.50177500	-1.28025200
H	-0.65867600	-3.01838800	-0.72135400
H	-0.38661100	-2.29839900	-2.31547700
C	-2.27787500	-0.13400500	-1.44409400
C	-3.56694500	0.37982700	-1.51067500
C	-4.66576500	-0.46821300	-1.41742000
C	-4.46768500	-1.83470900	-1.24814800
C	-3.17800300	-2.34753200	-1.17537400
H	-1.42023900	0.52615900	-1.49893100
H	-3.71401500	1.44832500	-1.63289900
H	-5.67191600	-0.06593200	-1.47107200
H	-5.31919500	-2.50217900	-1.16662400
H	-3.02738700	-3.41452600	-1.03652400
C	2.00903500	1.59586900	-2.16852700
C	1.34519700	2.53719800	-1.19372900
H	1.24023000	1.01717000	-2.69845800
H	2.56243000	2.16277500	-2.92200800
C	0.03688700	2.95981700	-1.42008400
C	-0.57725700	3.86069900	-0.55729000
C	0.10671700	4.33464500	0.55686700
C	1.41149000	3.91133500	0.79417700
C	2.02947300	3.02459700	-0.08041200
H	-0.50434300	2.58852200	-2.28605800
H	-1.59309000	4.18804800	-0.75351300
H	-0.37161500	5.03296500	1.23564100
H	1.95321200	4.28160900	1.65847800
H	3.05131700	2.70442400	0.09868400

Man-4B_DCM

Charge=+1, Multiplicity=+1

C	2.43050800	-0.13502500	-0.62668800
C	3.56617700	-0.97153400	-0.04751300
C	3.11604900	-1.76749500	1.18293800
O	2.17414100	-2.67961900	0.61087700
H	3.98438300	-2.32554900	1.55557800
C	2.53931100	-1.09628200	2.40495100
C	2.32486500	-2.49463200	-0.79779400
C	1.53254800	-1.24278000	-1.22319100

O	0.23180600	-1.13778100	-0.72252200
H	1.53663800	-1.18771100	-2.32009400
O	2.96901900	0.75080500	-1.57609700
H	1.87771600	0.42112900	0.13309600
O	3.64777500	-2.07092800	-0.97456300
H	4.52315900	-0.46490600	0.04491400
H	2.12435100	-3.42765200	-1.31881100
O	1.30276600	-0.38184100	2.15680400
H	3.22338100	-0.34424800	2.79451700
H	2.34241900	-1.84166400	3.17729300
C	0.20914500	-1.03896200	2.10461200
C	-1.02445300	-0.38029900	1.95499700
H	0.25382600	-2.12021200	2.23006200
C	-1.10756100	1.01750400	1.78544200
C	-2.34811900	1.60529000	1.69363500
C	-3.50206300	0.81669200	1.76117600
C	-3.42733500	-0.56456900	1.91702200
C	-2.18972500	-1.16996200	2.00746400
H	-0.20419000	1.61424200	1.72454400
H	-2.43137600	2.67682200	1.55915600
H	-4.47439700	1.29115000	1.68340900
H	-4.33100000	-1.15988100	1.95119700
H	-2.10659700	-2.24560200	2.12282200
C	-0.67767100	-2.07576300	-1.27612600
C	-2.06789200	-1.50784100	-1.28081100
H	-0.64800000	-3.01515800	-0.70949800
H	-0.37542600	-2.30501200	-2.30809200
C	-2.27674400	-0.14156900	-1.45081900
C	-3.56773000	0.36745500	-1.51975300
C	-4.66343400	-0.48440200	-1.42339500
C	-4.46015500	-1.84951600	-1.24811200
C	-3.16851200	-2.35724300	-1.17236000
H	-1.42161800	0.52158800	-1.50763600
H	-3.71850700	1.43514100	-1.64441000
H	-5.67108200	-0.08575100	-1.47667100
H	-5.30916400	-2.51963900	-1.16173300
H	-3.01352800	-3.42253800	-1.02581000
C	2.00529100	1.60537100	-2.16431000

C	1.33585600	2.54385400	-1.19058200
H	1.23991200	1.02245200	-2.69418600
H	2.55596900	2.17492400	-2.91786100
C	0.02768200	2.96441800	-1.42262600
C	-0.59292700	3.86122000	-0.56013800
C	0.08458100	4.33363500	0.55879500
C	1.38896300	3.91225600	0.80168900
C	2.01328800	3.02902300	-0.07215000
H	-0.50893500	2.59323200	-2.29146300
H	-1.60976000	4.18405100	-0.75862600
H	-0.39993200	5.02652300	1.23876000
H	1.92454000	4.27838800	1.67155400
H	3.03349000	2.70773900	0.11387800

Man-4B_ACN

Charge=+1, Multiplicity=+1

C	2.43416000	-0.12698500	-0.61756000
C	3.56788800	-0.96695600	-0.04070100
C	3.11496400	-1.77389900	1.18118300
O	2.17725600	-2.68419900	0.59752000
H	3.98306200	-2.33310100	1.55189600
C	2.53388600	-1.11424500	2.40702500
C	2.32944900	-2.48536800	-0.80765500
C	1.53778200	-1.23015800	-1.22441900
O	0.23561100	-1.12930600	-0.72740500
H	1.54482300	-1.16710700	-2.32094500
O	2.97626100	0.76361500	-1.56232100
H	1.88072700	0.42584000	0.14391300
O	3.65359000	-2.05811500	-0.97852200
H	4.52377700	-0.46020100	0.06125000
H	2.13072100	-3.41300600	-1.33867000
O	1.29957400	-0.39617800	2.15809700
H	3.21703000	-0.36662900	2.80616700
H	2.33293000	-1.86680300	3.17097800
C	0.20307200	-1.04762000	2.11285300
C	-1.02713400	-0.38223800	1.95907500
H	0.24090300	-2.12819000	2.24598000
C	-1.10166500	1.01513700	1.78358500

C	-2.33854700	1.61001100	1.68582700
C	-3.49728900	0.82850800	1.75379100
C	-3.43077700	-0.55227000	1.91656300
C	-2.19678000	-1.16465000	2.01334400
H	-0.19455400	1.60626300	1.72388900
H	-2.41554800	2.68135600	1.54653600
H	-4.46650800	1.30842400	1.67082900
H	-4.33788300	-1.14231100	1.95170800
H	-2.11957900	-2.23991200	2.13405800
C	-0.66777700	-2.07150300	-1.28465700
C	-2.06157200	-1.51276800	-1.28701200
H	-0.63205000	-3.01235200	-0.72132600
H	-0.36388900	-2.29456600	-2.31751800
C	-2.28042400	-0.14898800	-1.46578300
C	-3.57494400	0.35170300	-1.53064800
C	-4.66470200	-0.50661200	-1.42227300
C	-4.45162300	-1.86915700	-1.23840700
C	-3.15618100	-2.36821000	-1.16594200
H	-1.43002300	0.51928700	-1.53226000
H	-3.73292900	1.41780100	-1.65972400
H	-5.67515700	-0.11431800	-1.47015200
H	-5.29577200	-2.54383300	-1.14001300
H	-2.99324000	-3.43087300	-1.00962100
C	2.01265900	1.61504200	-2.15559900
C	1.33463200	2.55112500	-1.18553900
H	1.25235000	1.02932600	-2.68929600
H	2.56492500	2.18669700	-2.90649900
C	0.02795800	2.97098000	-1.42826000
C	-0.60163300	3.86389700	-0.56814900
C	0.06540400	4.33343100	0.55848500
C	1.36803800	3.91273000	0.81183900
C	2.00136200	3.03301900	-0.05931700
H	-0.50102600	2.60070600	-2.30211400
H	-1.61835000	4.18322900	-0.77281000
H	-0.42745500	5.02075900	1.23811400
H	1.89424700	4.27351800	1.68959000
H	3.01889100	2.70971600	0.13718700

Man-4B_DMSO

Charge=+1, Multiplicity=+1

C	2.43464600	-0.12735100	-0.61631300
C	3.56742800	-0.96857800	-0.03951600
C	3.11337000	-1.77644500	1.18130600
O	2.17551600	-2.68575400	0.59617300
H	3.98095100	-2.33647200	1.55192400
C	2.53208000	-1.11771900	2.40751000
C	2.32835500	-2.48550100	-0.80860200
C	1.53777100	-1.22932900	-1.22456400
O	0.23536200	-1.12803500	-0.72832200
H	1.54548300	-1.16536900	-2.32104500
O	2.97805300	0.76338100	-1.56036600
H	1.88136700	0.42547600	0.14525200
O	3.65295100	-2.05885300	-0.97850200
H	4.52355300	-0.46251800	0.06362200
H	2.12925800	-3.41239600	-1.34075800
O	1.29852100	-0.39845700	2.15836600
H	3.21555800	-0.37107200	2.80785500
H	2.33007900	-1.87098800	3.17045800
C	0.20127900	-1.04857300	2.11384800
C	-1.02810600	-0.38175300	1.95930200
H	0.23765800	-2.12906800	2.24799900
C	-1.10085600	1.01557200	1.78275900
C	-2.33697100	1.61191200	1.68408100
C	-3.49670400	0.83189600	1.75224200
C	-3.43193000	-0.54881700	1.91617900
C	-2.19870900	-1.16266100	2.01388500
H	-0.19299400	1.60554500	1.72309200
H	-2.41263700	2.68323700	1.54394900
H	-4.46529200	1.31295400	1.66851400
H	-4.33976700	-1.13772600	1.95153900
H	-2.12280300	-2.23789700	2.13547800
C	-0.66764600	-2.07033300	-1.28612000
C	-2.06172300	-1.51236000	-1.28787400
H	-0.63134700	-3.01152400	-0.72344800
H	-0.36377200	-2.29245600	-2.31919700
C	-2.28149600	-0.14889600	-1.46811300

C	-3.57633400	0.35107200	-1.53228700
C	-4.66554500	-0.50771000	-1.42185800
C	-4.45156900	-1.86991900	-1.23656900
C	-3.15577400	-2.36820900	-1.16469500
H	-1.43153200	0.51976000	-1.53633400
H	-3.73498200	1.41695500	-1.66230200
H	-5.67625000	-0.11596000	-1.46901100
H	-5.29525900	-2.54490500	-1.13633000
H	-2.99210000	-3.43054400	-1.00696900
C	2.01530800	1.61526200	-2.15441000
C	1.33677900	2.55147300	-1.18483100
H	1.25527700	1.02987800	-2.68882900
H	2.56843100	2.18680200	-2.90477800
C	0.03047900	2.97188100	-1.42870300
C	-0.59960600	3.86477400	-0.56891500
C	0.06654900	4.33372700	0.55850400
C	1.36877800	3.91244900	0.81300000
C	2.00261600	3.03275400	-0.05781700
H	-0.49787400	2.60192200	-2.30306900
H	-1.61611400	4.18430700	-0.77430300
H	-0.42682800	5.02080100	1.23801600
H	1.89418000	4.27250000	1.69153300
H	3.01971600	2.70878200	0.13976800

Man-4B_Water

Charge=+1, Multiplicity=+1

C	2.43537800	-0.12823600	-0.61451100
C	3.56655400	-0.97142500	-0.03757900
C	3.11060100	-1.78043000	1.18173100
O	2.17239500	-2.68812500	0.59451700
H	3.97729700	-2.34179000	1.55232700
C	2.52913400	-1.12268300	2.40831400
C	2.32634400	-2.48602900	-0.80971300
C	1.53760200	-1.22837100	-1.22470500
O	0.23485600	-1.12618600	-0.72957200
H	1.54628600	-1.16318500	-2.32111900
O	2.98103900	0.76239400	-1.55759900
H	1.88233700	0.42484700	0.14701400

O	3.65170400	-2.06056100	-0.97813800
H	4.52311200	-0.46655000	0.06727500
H	2.12659300	-3.41189100	-1.34339800
O	1.29672800	-0.40154900	2.15871200
H	3.21319400	-0.37741100	2.81017600
H	2.32550100	-1.87669700	3.17004900
C	0.19838700	-1.04970000	2.11514600
C	-1.02977500	-0.38074400	1.95956600
H	0.23261300	-2.13009200	2.25071800
C	-1.09990800	1.01652000	1.78160400
C	-2.33489800	1.61500900	1.68165800
C	-3.49608000	0.83716400	1.75007200
C	-3.43386100	-0.54346400	1.91559800
C	-2.20177600	-1.15945400	2.01457800
H	-0.19094700	1.60480100	1.72199800
H	-2.40862500	2.68631400	1.54039000
H	-4.46374100	1.31990900	1.66529800
H	-4.34276600	-1.13071400	1.95122400
H	-2.12775400	-2.23466200	2.13737100
C	-0.66774200	-2.06856100	-1.28801000
C	-2.06214200	-1.51148100	-1.28910600
H	-0.63081700	-3.01013300	-0.72607900
H	-0.36386500	-2.28964100	-2.32132400
C	-2.28305000	-0.14843000	-1.47129200
C	-3.57828200	0.35065900	-1.53462600
C	-4.66682100	-0.50867200	-1.42155800
C	-4.45174100	-1.87045400	-1.23439300
C	-3.15551700	-2.36780500	-1.16321700
H	-1.43362700	0.52067700	-1.54178000
H	-3.73773200	1.41626800	-1.66588100
H	-5.67783100	-0.11758700	-1.46780900
H	-5.29486500	-2.54580100	-1.13176700
H	-2.99094400	-3.42971500	-1.00362000
C	2.01995400	1.61534600	-2.15284800
C	1.34064500	2.55169500	-1.18396400
H	1.26035900	1.03079500	-2.68874500
H	2.57476400	2.18674500	-2.90209400
C	0.03493000	2.97298800	-1.42952200

C	-0.59586200	3.86584100	-0.57017700
C	0.06898100	4.33383100	0.55843700
C	1.37059400	3.91161900	0.81462500
C	2.00516400	3.03198200	-0.05573300
H	-0.49247800	2.60360800	-2.30469900
H	-1.61202300	4.18576700	-0.77667400
H	-0.42511800	5.02055300	1.23778500
H	1.89480900	4.27054600	1.69432200
H	3.02161600	2.70696600	0.14343600

Man-6B_Dioxane

Charge=+1, Multiplicity=+1

C	-0.29613700	3.11662400	0.57198400
O	-0.79678300	2.52295300	1.75929800
C	-2.08211000	2.11456200	1.33165400
C	-1.94140900	0.91626700	0.31970300
O	-2.56336300	1.16857800	-0.90593700
C	0.18646000	2.00659200	-0.35583300
O	-0.16836300	-0.22755700	-0.97262200
O	1.62544100	1.82003900	-0.16895500
H	0.51831100	3.79331800	0.82895200
C	-1.54043700	3.84211600	0.01031300
O	-2.62372100	3.23748800	0.70766800
H	-2.67728000	1.86186700	2.20762600
H	-1.65749000	3.70402900	-1.06783200
H	-1.52625600	4.90573900	0.25064500
C	-3.96873200	0.92318100	-0.91215000
C	-4.28514700	-0.52558600	-0.66223900
H	-4.29782100	1.22889200	-1.90753300
H	-4.47077500	1.56468000	-0.17921000
C	-5.03424000	-0.91733600	0.44434500
C	-5.29185800	-2.26429100	0.68451100
C	-4.79445700	-3.23046300	-0.18093200
C	-4.04490500	-2.84703000	-1.29077000
C	-3.79319200	-1.50324000	-1.52946200
H	-5.42174800	-0.16176800	1.12252700
H	-5.87893600	-2.55751100	1.54838500
H	-4.99401700	-4.28066800	0.00376800

H	-3.66378900	-3.59940200	-1.97361500
H	-3.20706200	-1.20126400	-2.39234900
C	-0.24046200	-1.60500400	-0.57188800
C	0.91908600	-1.99089200	0.29874100
H	-1.19809800	-1.79647200	-0.07723900
H	-0.23204500	-2.16327700	-1.50917300
C	2.15006200	-2.30221700	-0.28116100
C	3.25300200	-2.59070200	0.51062500
C	3.13768700	-2.56780300	1.89726200
C	1.91290100	-2.27578300	2.48639800
C	0.80819700	-1.99422700	1.68938400
H	2.23897700	-2.32646800	-1.36367600
H	4.20214600	-2.84061100	0.04830100
H	3.99802500	-2.79439700	2.51805400
H	1.81468200	-2.27580500	3.56664400
H	-0.15344700	-1.79094000	2.15290000
C	2.26545700	1.17324000	-1.05955500
C	-0.45449900	0.69163000	0.04271600
H	0.01869800	2.23342900	-1.41100500
H	0.01446300	0.38787100	0.98622500
C	3.58776200	0.73685400	-0.83179400
C	4.20685800	0.88068100	0.42597000
C	5.48985000	0.40977000	0.60054800
C	6.15707600	-0.20293300	-0.46386700
C	5.54891500	-0.35405400	-1.70943300
C	4.26261100	0.11083500	-1.89728000
H	3.76774700	-0.00161000	-2.85623200
H	7.16622200	-0.57295900	-0.31616600
H	6.08143200	-0.83521000	-2.52086600
H	3.66627200	1.34768800	1.24062700
H	5.97916500	0.50559900	1.56242400
H	1.76910500	0.97659400	-2.01015500
H	-2.35220000	0.01192900	0.78560100

Man-6B_Toluene

Charge=+1, Multiplicity=+1

C	1.42110300	-3.44692400	-0.15732600
O	1.95138600	-3.24038500	1.14433000

C	3.01730500	-2.35088900	0.86265800
C	2.42578700	-0.94364100	0.46221400
O	2.87029900	-0.52181800	-0.79408400
C	0.57079800	-2.23491200	-0.52905800
O	0.19690300	0.01530100	-0.07839100
O	-0.84762700	-2.49370100	-0.31232800
H	0.83153400	-4.36330300	-0.16196300
C	2.70174400	-3.53309600	-1.01876500
O	3.70143600	-2.93824100	-0.19730300
H	3.66815200	-2.29980800	1.73405400
H	2.61402400	-2.98833100	-1.96208900
H	2.98468200	-4.56856300	-1.21179400
C	3.90870000	0.45767700	-0.76406000
C	3.39440200	1.78652800	-0.28650900
H	4.26356200	0.52135900	-1.79403500
H	4.73990300	0.10997600	-0.13970400
C	2.54023200	2.53270500	-1.10131800
C	1.99869300	3.72792900	-0.64967200
C	2.30263800	4.19264200	0.62792400
C	3.15797600	3.46251700	1.44366300
C	3.70235900	2.26534100	0.98534800
H	2.29957400	2.16625100	-2.09497600
H	1.33855900	4.30181700	-1.29130400
H	1.87759000	5.12595100	0.98125300
H	3.40561200	3.82485500	2.43570200
H	4.37922400	1.70175000	1.62192300
C	-0.10780900	1.03695600	0.87204600
C	-1.31149200	1.77947500	0.37640100
H	-0.31594400	0.58448300	1.84925100
H	0.75939800	1.70065500	0.97200700
C	-1.36207200	2.23132400	-0.94340600
C	-2.48408100	2.90202200	-1.41289400
C	-3.56387200	3.13648300	-0.56536100
C	-3.51731700	2.69150300	0.75025900
C	-2.39729500	2.01049900	1.21611800
H	-0.51672400	2.05420800	-1.59992100
H	-2.51341300	3.25335800	-2.43921600
H	-4.43649400	3.66799400	-0.93008000

H	-4.35621800	2.86844600	1.41498100
H	-2.36910600	1.65378700	2.24156700
C	-1.65442800	-1.63216100	-0.80743900
C	0.90550300	-1.08427000	0.40264900
H	0.69601700	-1.93883400	-1.57312200
H	0.55194800	-1.35519000	1.40496200
C	-2.98565700	-1.52812300	-0.35138000
C	-3.47363100	-2.31722900	0.71066600
C	-4.77367900	-2.14439400	1.13182600
C	-5.58611300	-1.18969500	0.51046700
C	-5.10718000	-0.39992600	-0.53197000
C	-3.80685500	-0.56460700	-0.96741300
H	-3.40458900	0.05820300	-1.75957100
H	-6.60724100	-1.05898200	0.85273800
H	-5.74680200	0.34279100	-0.99277100
H	-2.82492000	-3.04619700	1.18232300
H	-5.16805300	-2.74339500	1.94410200
H	-1.29973800	-0.99280700	-1.61380800
H	2.67575000	-0.21212600	1.23767900

Man-6B_Et_2O

Charge=+1, Multiplicity=+1

C	-0.35098200	3.09539800	0.47173800
O	-0.88592900	2.56954700	1.67808300
C	-2.16470500	2.14958000	1.24315100
C	-2.01219300	0.89776800	0.30250600
O	-2.61607600	1.08324800	-0.94549400
C	0.14299900	1.93466100	-0.38512600
O	-0.24191300	-0.32509200	-0.90457000
O	1.57312400	1.75224500	-0.15086600
H	0.46386300	3.77746100	0.71135900
C	-1.57348000	3.80076400	-0.15622900
O	-2.68016600	3.24404600	0.54715800
H	-2.78399100	1.94959600	2.11587600
H	-1.66634200	3.60627500	-1.22769300
H	-1.55292000	4.87537000	0.02723100
C	-4.02233000	0.85563700	-0.95357300
C	-4.36846900	-0.56817100	-0.61004600

H	-4.33584300	1.09695000	-1.97179800
H	-4.52655500	1.55145000	-0.27350300
C	-5.21992100	-0.86294600	0.45171800
C	-5.51990200	-2.18406500	0.77324600
C	-4.95970800	-3.22156000	0.03822300
C	-4.10347300	-2.93511600	-1.02285300
C	-3.81241500	-1.61685200	-1.34565100
H	-5.65082900	-0.05220400	1.03273300
H	-6.18608000	-2.40130800	1.60158000
H	-5.18935000	-4.25189700	0.28868500
H	-3.66771600	-3.74330400	-1.60127300
H	-3.14212900	-1.39076900	-2.16934300
C	-0.23770600	-1.67221200	-0.40905800
C	1.04593600	-1.99120500	0.29999700
H	-1.10664200	-1.83365200	0.23734400
H	-0.35938200	-2.29709000	-1.29469800
C	2.16394400	-2.38962100	-0.43511400
C	3.37868100	-2.62473900	0.19528700
C	3.49216700	-2.45311600	1.57121900
C	2.38253900	-2.06796100	2.31565700
C	1.16386700	-1.84726400	1.68323100
H	2.07706900	-2.52058300	-1.51026800
H	4.23863900	-2.94082500	-0.38540100
H	4.44185200	-2.63165400	2.06423500
H	2.46249800	-1.95000600	3.39109100
H	0.29267500	-1.57752500	2.27378100
C	2.25058500	1.10829900	-1.01515300
C	-0.52254800	0.64753800	0.06150900
H	0.00358900	2.11021500	-1.45360300
H	-0.07188500	0.38363400	1.02501500
C	3.58259200	0.72482300	-0.75529300
C	4.30432200	0.12145400	-1.80293700
C	5.61054200	-0.27164200	-1.59113800
C	6.19127100	-0.07499400	-0.33897900
C	5.47692100	0.51300100	0.70831500
C	4.17368900	0.91457800	0.50986500
H	3.59802600	1.36298600	1.31079500
H	7.21621400	-0.38899700	-0.17243700

H	5.94588300	0.64573900	1.67588300
H	3.82948900	-0.02933000	-2.76655600
H	6.17882200	-0.73339500	-2.38941600
H	1.77997700	0.87665400	-1.97110300
H	-2.43459900	0.02202700	0.80992600

Man-6B_$CHCl_3$

Charge=+1, Multiplicity=+1

C	-0.34890000	3.09570300	0.47204100
O	-0.88481600	2.57029300	1.67839600
C	-2.16368000	2.15111100	1.24312100
C	-2.01185600	0.89905700	0.30283700
O	-2.61685600	1.08420300	-0.94484300
C	0.14459400	1.93473700	-0.38466100
O	-0.24303500	-0.32457200	-0.90525100
O	1.57416800	1.75091900	-0.14940400
H	0.46630600	3.77720300	0.71183800
C	-1.57057800	3.80189300	-0.15631200
O	-2.67813600	3.24604500	0.54680200
H	-2.78342300	1.95155300	2.11559900
H	-1.66318100	3.60744700	-1.22776800
H	-1.54917700	4.87648600	0.02703600
C	-4.02300700	0.85668200	-0.95146100
C	-4.36891600	-0.56758200	-0.60935500
H	-4.33788400	1.09919000	-1.96898800
H	-4.52649200	1.55151100	-0.26984000
C	-3.81363900	-1.61536400	-1.34684400
C	-4.10442200	-2.93406000	-1.02546100
C	-4.95969100	-3.22183300	0.03606900
C	-5.51910000	-2.18521300	0.77299600
C	-5.21933400	-0.86367400	0.45289200
H	-3.14396500	-1.38836600	-2.17076800
H	-3.66891300	-3.74147500	-1.60514900
H	-5.18896100	-4.25249900	0.28557100
H	-6.18428200	-2.40347500	1.60187500
H	-5.64930400	-0.05365500	1.03559200
C	-0.23943900	-1.67168600	-0.40985500
C	1.04387300	-1.99136200	0.29956000

H	-1.10847900	-1.83288100	0.23646900
H	-0.36106500	-2.29645500	-1.29557900
C	1.16097600	-1.84921500	1.68304800
C	2.37936000	-2.07068400	2.31586500
C	3.48944300	-2.45476600	1.57152600
C	3.37679100	-2.62435100	0.19524700
C	2.16238100	-2.38846900	-0.43552000
H	0.28954600	-1.57967700	2.27333800
H	2.45883300	-1.95369100	3.39144300
H	4.43898800	-2.63342800	2.06477800
H	4.23725700	-2.93884500	-0.38554800
H	2.07629800	-2.51720500	-1.51098500
C	2.25216000	1.10927700	-1.01497000
C	-0.52244800	0.64809400	0.06123300
H	0.00618500	2.11108000	-1.45310800
H	-0.07197800	0.38325100	1.02454700
C	3.58390100	0.72488200	-0.75519000
C	4.30575200	0.12299600	-1.80358900
C	5.61153100	-0.27158400	-1.59164100
C	6.19163600	-0.07772700	-0.33877200
C	5.47715600	0.50900300	0.70917400
C	4.17436600	0.91195800	0.51066200
H	3.59860600	1.35937300	1.31207200
H	7.21616100	-0.39302000	-0.17213900
H	5.94566200	0.63952500	1.67726200
H	3.83136100	-0.02551400	-2.76775900
H	6.17988400	-0.73244000	-2.39037900
H	1.78213900	0.88025200	-1.97177700
H	-2.43417100	0.02369900	0.81091400

Man-6B_DCM

Charge=+1, Multiplicity=+1

C	-0.32292000	3.10004200	0.48818700
O	-0.85622400	2.57057200	1.69498800
C	-2.13898900	2.15974000	1.26411500
C	-1.99839700	0.91286100	0.31566700
O	-2.61252600	1.10839200	-0.92690500
C	0.16012400	1.94212300	-0.37833500

O	-0.24334200	-0.31344300	-0.90881700
O	1.58941500	1.74905300	-0.15225200
H	0.49692200	3.77584400	0.72730200
C	-1.54388000	3.81625100	-0.12923900
O	-2.65169300	3.26240700	0.57725900
H	-2.75514900	1.95702700	2.13829300
H	-1.64317600	3.62821200	-1.20101700
H	-1.51524900	4.88963000	0.05964000
C	-4.01743800	0.87771400	-0.92491700
C	-4.35677700	-0.55602200	-0.61623000
H	-4.34417100	1.14468200	-1.93261500
H	-4.51643600	1.55291900	-0.22058500
C	-3.80467800	-1.58277100	-1.38506200
C	-4.09090000	-2.91030000	-1.09725200
C	-4.93842100	-3.22808400	-0.03794100
C	-5.49403100	-2.21240500	0.73069500
C	-5.19886500	-0.88204700	0.44399900
H	-3.13999000	-1.33313600	-2.20637000
H	-3.65629100	-3.70074900	-1.70054000
H	-5.16346800	-4.26559000	0.18596500
H	-6.15187000	-2.45401500	1.55900000
H	-5.62465500	-0.08886900	1.05226800
C	-0.25442100	-1.66259300	-0.41975200
C	1.00938700	-1.99040800	0.32047700
H	-1.13918300	-1.82487600	0.20468300
H	-0.35526000	-2.28193600	-1.31182500
C	1.08443300	-1.87649900	1.70929600
C	2.28437000	-2.10779800	2.37385700
C	3.41810700	-2.47045600	1.65479000
C	3.34786300	-2.60884500	0.27192200
C	2.15140300	-2.36567400	-0.38957500
H	0.19538100	-1.61745500	2.27747500
H	2.33172400	-2.01166700	3.45338300
H	4.35396300	-2.65386700	2.17199100
H	4.22780600	-2.90293400	-0.29019900
H	2.09819500	-2.46880600	-1.46978900
C	2.25964700	1.11722900	-1.03090700
C	-0.51184200	0.65664700	0.06361300

H	0.01752800	2.12816200	-1.44447400
H	-0.05837300	0.38364100	1.02314500
C	3.59073100	0.72202700	-0.78378200
C	4.29644000	0.11263600	-1.83875700
C	5.59725800	-0.30320800	-1.63573500
C	6.18852700	-0.12102200	-0.38643300
C	5.49062300	0.47615200	0.66707700
C	4.19304600	0.89944800	0.47779700
H	3.63012200	1.35504800	1.28369300
H	7.20865000	-0.45365900	-0.22683800
H	5.96787500	0.59826400	1.63196300
H	3.81319500	-0.02648200	-2.79983700
H	6.15279000	-0.77199400	-2.43882900
H	1.78264600	0.90174300	-1.98706900
H	-2.42096500	0.03657800	0.82155400

Man-6B_ACN

Charge=+1, Multiplicity=+1

C	-1.23827500	-3.46155300	0.09319900
O	-1.79316300	-3.27903000	-1.20489500
C	-2.91763200	-2.47204900	-0.90701800
C	-2.42967600	-1.03219800	-0.49101300
O	-2.92170300	-0.65518300	0.76542500
C	-0.47718300	-2.19729400	0.48088900
O	-0.28789400	0.09171700	0.06993400
O	0.95210200	-2.35756700	0.25528100
H	-0.58261300	-4.33082100	0.08335300
C	-2.50153700	-3.65556000	0.95942700
O	-3.55176700	-3.12829500	0.14888100
H	-3.57691000	-2.45443100	-1.77286800
H	-2.44757900	-3.11751900	1.90856900
H	-2.70223100	-4.71165100	1.14194700
C	-4.00622300	0.26974200	0.73251300
C	-3.54879800	1.65063200	0.35082400
H	-4.41346600	0.26352100	1.74540300
H	-4.78704600	-0.08334400	0.04910600
C	-3.83746300	2.18518500	-0.90340200
C	-3.35598100	3.44070900	-1.26762500

C	-2.58484900	4.17413400	-0.37405200
C	-2.29994400	3.65172800	0.88628200
C	-2.77721400	2.39809100	1.24367400
H	-4.44615100	1.61725100	-1.60147000
H	-3.58357200	3.84303700	-2.24915800
H	-2.20726500	5.15168900	-0.65495300
H	-1.70487200	4.22598100	1.58883300
H	-2.54645600	1.98741200	2.22219000
C	-0.06153600	1.13969900	-0.88066400
C	1.22917700	1.81364500	-0.52820500
H	-0.00513200	0.71643700	-1.88903200
H	-0.90489500	1.83674100	-0.84088100
C	1.34809500	2.51283000	0.67389700
C	2.55875500	3.09188100	1.03329400
C	3.66074400	2.98605000	0.18869200
C	3.54784300	2.29573600	-1.01278800
C	2.33759400	1.70826300	-1.36617400
H	0.48722900	2.59779400	1.32948900
H	2.64314400	3.63028300	1.97160800
H	4.60638200	3.43768000	0.46983900
H	4.40584900	2.20259400	-1.67022200
H	2.25502500	1.15694600	-2.29826400
C	1.71246200	-1.50917500	0.83209400
C	-0.90247900	-1.05785200	-0.42588800
H	-0.61396900	-1.93850100	1.53270200
H	-0.53039700	-1.28279400	-1.43237200
C	3.06212700	-1.35314500	0.45228600
C	3.61076100	-2.04089400	-0.64905000
C	4.93043700	-1.82786400	-0.98155800
C	5.70425700	-0.93814200	-0.22808000
C	5.16567900	-0.25102700	0.85646900
C	3.84210900	-0.45081600	1.19910300
H	3.39570500	0.08853500	2.02753500
H	6.74178800	-0.77589100	-0.50010000
H	5.77568100	0.44436700	1.41983300
H	2.99113600	-2.72078500	-1.22183200
H	5.36996500	-2.34471800	-1.82631400
H	1.30174300	-0.92076900	1.65046100

H	-2.72918600	-0.31670900	-1.26316100

Man-6B_DMSO

Charge=+1, Multiplicity=+1

C	-0.32085800	3.10166000	0.46766000
O	-0.86071000	2.59110600	1.68082100
C	-2.14477400	2.18217100	1.25301400
C	-2.01030200	0.92261200	0.32120800
O	-2.62753200	1.10509000	-0.92288700
C	0.15721800	1.93039000	-0.38316900
O	-0.26685400	-0.32850200	-0.89121200
O	1.58337500	1.73149000	-0.14864800
H	0.50247100	3.77546600	0.69938700
C	-1.53569800	3.81696900	-0.16158800
O	-2.64933400	3.28037400	0.55122400
H	-2.76402700	1.99371300	2.12811500
H	-1.63378800	3.61422500	-1.23060900
H	-1.49995000	4.89272500	0.01147500
C	-4.03120200	0.87134500	-0.91394100
C	-4.36638700	-0.56371500	-0.60524800
H	-4.36476000	1.13867700	-1.91940400
H	-4.52857700	1.54350300	-0.20541600
C	-3.81136900	-1.58924800	-1.37371100
C	-4.09355700	-2.91762800	-1.08520400
C	-4.94036500	-3.23755600	-0.02576900
C	-5.49909900	-2.22295100	0.74237200
C	-5.20762600	-0.89178800	0.45512500
H	-3.14671600	-1.33894600	-2.19476000
H	-3.65484900	-3.70681400	-1.68718100
H	-5.16116100	-4.27572100	0.19957300
H	-6.15504100	-2.46592100	1.57185600
H	-5.63383100	-0.09960400	1.06426700
C	-0.26759200	-1.67056700	-0.38389300
C	1.01398800	-1.99027400	0.32944200
H	-1.13610300	-1.82327200	0.26528200
H	-0.39068100	-2.30239000	-1.26443000
C	1.12389400	-1.85730100	1.71443400
C	2.34021100	-2.08067100	2.35160200

C	3.45520000	-2.45558700	1.60960600
C	3.34997000	-2.61313100	0.23096900
C	2.13762900	-2.37667300	-0.40386600
H	0.25020000	-1.58739200	2.30110000
H	2.41532200	-1.96680300	3.42788200
H	4.40439600	-2.63008800	2.10515600
H	4.21576000	-2.91319900	-0.34961200
H	2.05873300	-2.49018700	-1.48140000
C	2.25984700	1.10743400	-1.02767800
C	-0.52538100	0.65465900	0.07108400
H	0.02016900	2.10683400	-1.45152300
H	-0.07436700	0.38783600	1.03331000
C	3.59154500	0.71666400	-0.77671300
C	4.18810600	0.89245600	0.48776800
C	5.48716000	0.47492200	0.68017700
C	6.19159900	-0.11526600	-0.37296800
C	5.60581200	-0.29554300	-1.62506200
C	4.30368000	0.11494700	-1.83174400
H	3.82446600	-0.02296500	-2.79496400
H	7.21242800	-0.44427400	-0.21060300
H	6.16617200	-0.75939000	-2.42764600
H	3.61992500	1.34241800	1.29310900
H	5.96029800	0.59551800	1.64726500
H	1.78852300	0.89429600	-1.98689400
H	-2.43646500	0.05529700	0.83884100

Man-6B_Water

Charge=+1, Multiplicity=+1

C	-0.31963000	3.10246900	0.46630800
O	-0.85948000	2.59403300	1.68046300
C	-2.14400200	2.18545800	1.25375800
C	-2.01085000	0.92463500	0.32349400
O	-2.62840300	1.10633000	-0.92068900
C	0.15702200	1.92967600	-0.38326100
O	-0.26916000	-0.32986500	-0.88754200
O	1.58313600	1.73016900	-0.14961800
H	0.50441800	3.77581500	0.69671000
C	-1.53408400	3.81805700	-0.16323800

O	-2.64800700	3.28326900	0.55076000
H	-2.76302600	1.99849800	2.12933600
H	-1.63276000	3.61412900	-1.23195700
H	-1.49728200	4.89398700	0.00849200
C	-4.03178300	0.87149800	-0.91128200
C	-4.36567900	-0.56449500	-0.60533700
H	-4.36638500	1.14060500	-1.91594000
H	-4.52928100	1.54168500	-0.20095500
C	-5.20663900	-0.89531200	0.45441400
C	-5.49713900	-2.22726600	0.73902200
C	-4.93770900	-3.23995100	-0.03117700
C	-4.09115400	-2.91727100	-1.08999500
C	-3.80993300	-1.58809900	-1.37585200
H	-5.63321600	-0.10466500	1.06527000
H	-6.15274000	-2.47234200	1.56816100
H	-5.15763500	-4.27873300	0.19218100
H	-3.65170900	-3.70489700	-1.69347400
H	-3.14542200	-1.33582100	-2.19639500
C	-0.26970000	-1.67095300	-0.37776600
C	1.01267600	-1.98986900	0.33456100
H	-1.13733000	-1.82213600	0.27295700
H	-0.39415800	-2.30437200	-1.25696700
C	2.13560500	-2.37688800	-0.39954500
C	3.34861800	-2.61260300	0.23430600
C	3.45524100	-2.45374300	1.61270500
C	2.34097700	-2.07815100	2.35546500
C	1.12398100	-1.85545400	1.71930500
H	2.05571900	-2.49107000	-1.47692800
H	4.21391200	-2.91282900	-0.34693600
H	4.40504000	-2.62740100	2.10740200
H	2.41724300	-1.96292400	3.43152200
H	0.25094900	-1.58463000	2.30652600
C	2.25886000	1.10614100	-1.02920500
C	-0.52624800	0.65512500	0.07328300
H	0.01922900	2.10493400	-1.45170000
H	-0.07510200	0.38941800	1.03573500
C	3.59087500	0.71557400	-0.77948800
C	4.18903000	0.89263800	0.48405000

C	5.48811600	0.47472100	0.67541300
C	6.19100100	-0.11711100	-0.37786800
C	5.60366800	-0.29847400	-1.62906500
C	4.30148700	0.11245900	-1.83473700
H	3.82103600	-0.02638400	-2.79720300
H	7.21181200	-0.44654700	-0.21628600
H	6.16281500	-0.76359200	-2.43175700
H	3.62203400	1.34388200	1.28950500
H	5.96246900	0.59623400	1.64178900
H	1.78668900	0.89250200	-1.98786200
H	-2.43765300	0.05829800	0.84216600
