# Supplementary material for: Mechanistic insight into benzylidene-directed glycosylation reactions using cryogenic infrared spectroscopy
Source: Nat Synth. 2024 Jul 26;3(11):1377–84. doi: 10.1038/s44160-024-00619-0 (PMC11549046; doi:10.1038/s44160-024-00619-0)
Supplement: Supplementary file 4 — SI coordinated_counterion.pdf. [file 44160_2024_619_MOESM4_ESM.pdf]

Beta Glucosyl triflate

Charge=0, Multiplicity=+1

O	-0.25486800	-1.37027000	1.47268100
C	-1.53812400	-0.74910300	1.46066100
C	-1.67514400	0.29550800	0.35904700
C	-0.71137800	1.43075200	0.64139700
O	-2.99705900	0.78306100	0.37173500
C	0.66733400	0.87224600	1.06319800
H	-1.10207200	1.95991200	1.51453300
O	-0.66962800	2.40644300	-0.37372600
C	0.77479100	-0.64897300	0.94393500
O	0.92400800	1.22551500	2.40403000
H	1.43054500	1.28624400	0.40125500
H	1.70564600	-1.01918700	1.37035500
C	-2.59295000	-1.82070600	1.25052600
H	-1.70176100	-0.25786100	2.42758900
O	-3.86451900	-1.21167400	1.18292400
H	-2.61312000	-2.52929500	2.07948200
H	-2.37332900	-2.36855000	0.32169200
H	-1.46631400	-0.17538400	-0.61329200
C	-3.91695200	-0.25550500	0.15692400
C	-5.30208300	0.31715400	0.07104300
H	-3.64499100	-0.75904200	-0.79168700
C	-6.40356500	-0.46919400	0.39697300
C	-7.68750300	0.04618300	0.26998600
C	-7.87720900	1.34635900	-0.18422400
C	-6.77674600	2.13152100	-0.50801700
C	-5.49104500	1.61971200	-0.38253000
H	-4.62750200	2.22973800	-0.62064800
H	-6.24770900	-1.47748100	0.76238600
H	-8.54238800	-0.56910700	0.53055500
H	-8.88046400	1.74795400	-0.28195500
H	-6.91842500	3.14931400	-0.85635700
C	2.21683400	1.79865200	2.61519900
C	3.33799100	0.87382300	2.23429300
H	2.24165900	2.03379900	3.68095800
H	2.29370500	2.73484800	2.04761700
C	4.01168900	1.04522300	1.02630800

C	4.98199400	0.13411800	0.62333700
C	5.28903200	-0.95431900	1.42850300
C	4.63020700	-1.12677700	2.64235600
C	3.66031600	-0.21798300	3.04250000
H	3.77349800	1.89401200	0.39173500
H	5.48724500	0.27238200	-0.32674300
H	6.03440600	-1.67456300	1.10900800
H	4.86850600	-1.97687500	3.27288500
H	3.13502700	-0.36016100	3.98281800
C	-0.02311300	2.05837700	-1.58480500
C	1.40773900	2.53665900	-1.64968700
H	-0.60161300	2.54137500	-2.37958500
H	-0.05711900	0.97983400	-1.76816200
C	2.35945400	1.81325000	-2.36578700
C	3.66604200	2.27916500	-2.46919400
C	4.03609600	3.46677800	-1.84885700
C	3.09207700	4.18580200	-1.12082400
C	1.78486100	3.72452300	-1.02330700
H	1.04486800	4.27647400	-0.45301800
H	2.08676200	0.87089900	-2.83086300
H	4.39739200	1.70383600	-3.02758100
H	5.05652200	3.82773300	-1.92430900
H	3.37566800	5.11104900	-0.62945200
S	1.75425100	-2.03424200	-1.13527500
C	0.47740100	-3.37475500	-1.37312400
F	0.10218000	-3.86628700	-0.20687500
F	-0.57997000	-2.87826100	-2.00105500
F	1.01462800	-4.33491600	-2.10644200
O	0.84579900	-0.88009900	-0.52382000
O	2.13269100	-1.60823600	-2.45533800
O	2.70326500	-2.51221300	-0.16479400

Glc-6B-OTf

Charge=0, Multiplicity=+1

C	-0.44848000	-0.25620800	1.88793900
C	-1.85671900	-0.82486200	2.02296000
C	-2.55955100	-0.09418700	3.20462000
O	-1.77602700	-2.20536100	2.28703600

H	-2.40799300	-0.61687500	1.10229000
O	-1.62903400	0.67136200	3.94635500
C	-1.40049500	1.74965900	3.05079600
C	-0.52844300	1.26351400	1.90214200
O	0.86007700	1.69506000	2.03900300
H	-0.88406700	1.62318300	0.93568700
O	0.24651800	-0.57331800	0.71722600
H	0.12074800	-0.58063500	2.77009000
C	-2.82471400	2.06026800	2.55509800
H	-0.93228500	2.57187800	3.59227400
O	-3.51223400	0.82869600	2.75001500
H	-2.85574000	2.34312400	1.49970500
H	-3.30020400	2.82920900	3.16896500
H	-3.02879300	-0.82224400	3.86676000
C	-2.61046300	-3.02717600	1.46548600
C	-1.92427200	-3.42517600	0.19036300
H	-3.55406900	-2.50852200	1.26027300
H	-2.83157000	-3.91055700	2.06913800
C	-1.93433700	-2.58758600	-0.92534400
C	-1.20734400	-2.92113400	-2.06191800
C	-0.47189700	-4.10033900	-2.09771800
C	-0.46780700	-4.95027200	-0.99640500
C	-1.19035200	-4.61125200	0.14122100
H	-2.48708000	-1.65301800	-0.91223900
H	-1.20978500	-2.23689400	-2.90247200
H	0.10109500	-4.35600900	-2.98310800
H	0.10075400	-5.87436200	-1.02234300
H	-1.17963000	-5.26761400	1.00728600
C	1.78828200	-1.07842000	-1.57635900
C	2.13870600	-1.55875600	-0.31375400
C	3.46511900	-1.89497700	-0.05400800
C	4.43368700	-1.76509500	-1.04331400
C	4.08221600	-1.28461700	-2.29961100
C	2.75932200	-0.93823300	-2.55983900
H	3.74478200	-2.25914100	0.93094400
H	5.46346800	-2.03315200	-0.82948700
H	4.83584900	-1.18265800	-3.07422600
H	2.47313300	-0.56109000	-3.53649700

C	2.93748200	1.68127200	0.94607400
C	3.56836800	1.71958100	-0.30715000
C	4.95031700	1.70878400	-0.37458100
C	5.69433200	1.65841000	0.79935800
C	5.07007000	1.61655800	2.04931200
C	3.69292500	1.62604900	2.12995700
H	2.96188700	1.73394300	-1.20645400
H	5.44649500	1.72487200	-1.33750400
H	6.77816000	1.64356200	0.74570800
H	5.66907000	1.57171500	2.95182100
H	3.18626400	1.58426100	3.08756000
C	1.09450600	-1.71690700	0.75070900
H	0.49309900	-2.61562000	0.58237000
H	1.56434900	-1.80227800	1.73956200
C	1.51661200	1.65074300	0.94031400
H	0.95311200	1.65373900	-0.00148000
H	0.76093500	-0.80560800	-1.79299800
S	-1.44964900	1.29787000	-1.73976100
O	-2.36286700	0.95291700	-0.63870800
O	-1.13377000	0.21886500	-2.67097700
O	-0.28493700	2.09967600	-1.30122400
C	-2.42439700	2.51547700	-2.74805200
F	-1.70233300	2.96446900	-3.77197800
F	-2.78169800	3.55978100	-1.99828700
F	-3.52768500	1.94763900	-3.22696300

Glc-4B-OTf

Charge=0, Multiplicity=+1

C	0.73767200	0.83747300	-2.80581000
O	0.96370900	-0.50748800	-2.39101800
C	-0.64171500	0.72774300	-3.48662400
H	1.48170400	1.10350500	-3.56803800
C	0.90655600	1.76151200	-1.62552400
C	-1.74669000	0.48570500	-2.45564100
C	-1.37668200	-0.94099000	-1.96208500
H	-2.71480000	0.41484000	-2.96938200
O	-1.74237300	1.51628300	-1.51332200
C	-0.17293100	-1.22270100	-2.87082400

H	-1.08997200	-0.94174700	-0.90593500
O	-2.41956200	-1.84183100	-2.25342400
O	-0.50377000	-0.56077300	-4.07306500
H	-0.84597600	1.48328000	-4.24199000
H	0.07998300	-2.26614400	-3.04046700
C	-2.74986900	-2.75124200	-1.20760100
C	-3.46124300	-2.06590300	-0.07468900
H	-1.85116500	-3.26036400	-0.84027800
H	-3.39388600	-3.49561800	-1.68232200
C	-4.73203900	-1.52228600	-0.27598700
C	-5.35205000	-0.79559600	0.73049500
C	-4.70642800	-0.60070400	1.95044300
C	-3.45007900	-1.15230800	2.16378400
C	-2.83482800	-1.88768900	1.15513100
H	-5.22336400	-1.65521100	-1.23577000
H	-6.33809400	-0.37293000	0.56498600
H	-5.18494600	-0.01726200	2.73028200
H	-2.92697000	-1.00702100	3.10260500
H	-1.85096100	-2.30330000	1.33789300
C	-2.81037000	1.45358200	-0.56946800
C	-2.57104800	2.49236200	0.48001100
H	-2.84022300	0.46033000	-0.11362300
H	-3.76328200	1.62186200	-1.08851900
C	-3.22332900	3.72140700	0.44029900
C	-2.97421200	4.68394100	1.41304800
C	-2.06437800	4.42177200	2.43069700
C	-1.40758600	3.19519900	2.47442300
C	-1.66071700	2.23324400	1.50525800
H	-3.93501000	3.92373700	-0.35530700
H	-3.49225500	5.63694100	1.37793200
H	-1.87212600	5.16968300	3.19333100
H	-0.70328100	2.98163900	3.27220600
H	-1.15159400	1.27445200	1.53769400
O	2.35229300	1.79113800	-1.34304000
H	0.39166800	1.41210300	-0.73529500
H	0.64481200	2.79340300	-1.85242900
C	2.78539700	1.09392100	-0.37326400
C	4.17946800	1.01961600	-0.11624000

H	2.04723200	0.56473900	0.24705400
C	5.12596400	1.63753800	-0.95774300
C	6.46578900	1.52480100	-0.65959600
C	6.86842800	0.80299700	0.47024900
C	5.93786700	0.19421000	1.30384700
C	4.58539100	0.29372900	1.01735600
H	4.79107100	2.18777800	-1.82954300
H	6.26425600	-0.36020100	2.17600400
H	3.83158400	-0.16388200	1.65697600
H	7.20850600	1.98995700	-1.29760600
H	7.92664000	0.71881700	0.69651400
S	0.91499400	-1.20177700	1.98148800
C	1.48379400	-2.66004700	0.97570400
O	-0.11429300	-1.73652600	2.85734700
O	0.42906700	-0.27795900	0.92757800
O	2.15626500	-0.74138800	2.61406000
F	1.95587500	-3.62936800	1.74968400
F	2.45710700	-2.28704800	0.13414500
F	0.48246700	-3.15962100	0.24288300

Beta Mannosyl triflate

Charge=0, Multiplicity=+1

C	0.90153700	0.69925900	-0.38816700
C	-0.06525500	1.48282800	0.47495500
C	0.46879900	0.70756600	-1.84658800
O	2.18248500	1.28308600	-0.32919100
H	0.95366300	-0.34204800	-0.04366300
O	-0.80912600	0.10505300	-2.05830400
H	0.43300900	1.74411400	-2.20431500
C	1.50600800	-0.07096700	-2.64159100
C	-1.62501600	0.00308200	-0.97475800
C	-1.52870400	1.14421400	0.02675500
H	-1.93825900	2.01545500	-0.50325800
O	-2.36284100	0.78831200	1.09009700
H	0.11408300	2.54106500	0.26028300
O	0.14427400	1.38349600	1.85761900
O	2.78442800	0.48400400	-2.42042500
H	1.30947900	-0.01807000	-3.71332600

H	1.47361600	-1.12503000	-2.32528800
C	3.10497100	0.51036300	-1.04908500
C	4.48561700	1.07222200	-0.87865800
H	3.05972900	-0.52325500	-0.65801400
C	4.76234300	2.00176200	0.11795300
C	6.05951700	2.47109200	0.29091500
C	7.08445600	2.01221000	-0.52759900
C	6.80700100	1.08494600	-1.52664100
C	5.51172600	0.61663900	-1.70260000
H	3.95639300	2.35562100	0.74895800
H	6.26878000	3.19983300	1.06730800
H	8.09668900	2.37824500	-0.39054200
H	7.60187700	0.72748800	-2.17293700
H	5.28758400	-0.09727200	-2.48759200
C	0.15063300	0.09118400	2.45773000
C	1.44904200	-0.66046000	2.30651200
H	-0.68040800	-0.51395200	2.09432500
H	-0.03452700	0.29280300	3.51738100
C	2.67502800	-0.00876600	2.42620800
C	1.43024100	-2.03188200	2.05498300
C	2.61981300	-2.73863400	1.91453200
C	3.83907500	-2.07849600	2.01913400
C	3.86380900	-0.71149600	2.27863500
H	2.68787500	1.06051600	2.60766700
H	4.76832000	-2.62557600	1.89819400
H	4.81225100	-0.18972300	2.35252600
H	0.47670400	-2.54368200	1.96170500
H	2.59292900	-3.80491700	1.71389400
C	-3.02576500	1.87897600	1.71050800
C	-4.04757100	2.50489400	0.79948100
H	-2.29665100	2.62126600	2.05635700
H	-3.50516100	1.44566800	2.59214400
C	-4.14076400	3.88709200	0.66643300
C	-5.09492900	4.45329300	-0.17353900
C	-5.86070400	2.25260300	-0.77441500
C	-5.95467700	3.63646000	-0.89643900
H	-3.46080500	4.52623100	1.22324200
H	-6.69540900	4.07528700	-1.55662900

H	-5.15925100	5.53215900	-0.26938500
C	-4.91470600	1.68852100	0.07021100
H	-4.83532400	0.60917700	0.16441800
H	-6.53027600	1.61121000	-1.33813300
S	-2.38613300	-2.28362100	0.18926500
C	-1.90163300	-3.65890700	-0.96980400
O	-1.27104300	-1.23523700	-0.24080200
O	-3.70032300	-1.84383100	-0.20761900
O	-2.08308200	-2.73301800	1.51984900
F	-2.70828500	-4.68827300	-0.77319000
F	-2.01348400	-3.24114300	-2.22063900
F	-0.65198900	-4.02509800	-0.73792400
H	-2.65249000	-0.11657000	-1.31794100

Man-6B-OTf

Charge=0, Multiplicity=+1

C	-0.63436000	-1.07797600	-0.78484900
C	-1.98756500	-0.42255700	-1.04464700
C	-1.78927600	0.79797200	-2.01289000
O	-2.84381600	-1.39585600	-1.58692500
H	-2.36472800	-0.05334200	-0.08647200
O	-0.41407700	0.97365400	-2.29732800
C	-0.14895700	-0.18169000	-3.06200300
C	0.09104400	-1.33944100	-2.09092300
O	1.53390900	-1.49603100	-1.82758500
O	-0.71793700	-2.27769200	-0.06502600
C	-1.44331900	-0.34840000	-3.89683300
H	0.73278100	-0.00299300	-3.68062700
O	-2.36162300	0.52797100	-3.26323000
H	-1.81797900	-1.37595900	-3.87932600
H	-1.30213500	-0.01548400	-4.92670100
H	-2.17828100	1.73055600	-1.61331800
C	-4.21706500	-1.12766000	-1.37230100
C	-4.59538600	-1.20200500	0.08420600
H	-4.74827500	-1.89392000	-1.94434600
H	-4.49302200	-0.15282700	-1.79406400
C	-4.13245600	-2.26037400	0.86712500
C	-4.47367100	-2.34136500	2.20975200

C	-5.28435900	-1.36699900	2.78581300
C	-5.74659700	-0.31001300	2.01279700
C	-5.39784500	-0.22706700	0.66843100
H	-3.48212000	-3.00255600	0.41496900
H	-4.10605200	-3.16581400	2.81261800
H	-5.54671000	-1.42834100	3.83676600
H	-6.36672000	0.46041200	2.45885300
H	-5.74487200	0.61065900	0.06978200
C	1.82406600	-2.87130100	1.43535600
C	0.87418500	-1.88908500	1.73061900
C	1.27717200	-0.73592900	2.39958600
C	2.60647500	-0.56278900	2.77338900
C	3.54114400	-1.54963600	2.48890900
C	3.14751000	-2.70641900	1.81748600
H	0.55186000	0.03746200	2.62709600
H	2.89703700	0.35360200	3.27580500
H	4.57740600	-1.42092400	2.78441300
H	3.87604800	-3.48024000	1.59713200
C	3.52936000	-0.70065500	-0.90710500
C	4.28116900	-1.83254100	-1.27685700
C	5.62055200	-1.88194200	-0.96088000
C	6.21330000	-0.81510300	-0.27452700
C	5.47281400	0.29746600	0.10451800
C	4.12262200	0.36279000	-0.20334000
H	3.79708000	-2.64869400	-1.80073200
H	6.21640400	-2.74410400	-1.23829700
H	7.26998000	-0.86349900	-0.03068700
H	5.94383700	1.10992000	0.64561200
H	3.50068900	1.20627700	0.08845700
C	-0.56561100	-2.09213000	1.33991200
H	-0.95913200	-3.00649800	1.79134800
H	-1.17774000	-1.25257500	1.68721100
C	2.14834400	-0.57417400	-1.19557300
H	1.51417300	-3.76855100	0.90714100
S	0.48050400	2.35908200	1.00239500
O	-0.71760300	1.51251700	0.88571200
O	1.54594500	1.99688800	0.03964300
O	0.94951900	2.61589500	2.35772600

C	-0.10201700	4.00627400	0.36996600
F	-1.04107300	4.50791400	1.16891300
F	-0.62294100	3.88059000	-0.85340600
F	0.90452300	4.87448900	0.30645700
H	-0.07487200	-0.32368800	-0.22130600
H	-0.17756600	-2.30834100	-2.50948500
H	1.62810600	0.34493500	-0.88253600

Man-4B-OTf

Charge=0, Multiplicity=+1

C	-0.07964700	-0.55567400	-2.07468500
O	0.62374900	0.02250500	-3.17831100
C	0.40159600	-2.02535200	-2.10933800
H	-1.15298400	-0.52768900	-2.28453400
C	0.19777400	0.21012300	-0.80292900
C	1.83139200	-2.15674800	-1.56493300
C	2.59163500	-1.26321700	-2.60366500
O	2.04877200	-1.71066800	-0.26065600
H	2.13639800	-3.20588400	-1.67289100
O	0.66914100	-2.17732900	-3.50534800
H	-0.33956700	-2.73836700	-1.76137600
C	1.45864200	-1.01699700	-3.62563100
O	3.03344600	-0.04486100	-2.08678200
H	3.41482700	-1.81401400	-3.07578900
H	1.75674000	-0.83511700	-4.65539900
O	-0.63442200	1.42971200	-0.83275200
C	-2.44481000	2.64455200	0.02594300
C	-3.50082900	2.59205600	0.95166400
C	-4.40448600	3.63991400	1.00547300
C	-4.25026700	4.72478400	0.14813800
C	-3.19998500	4.77738200	-0.77366100
C	-2.29521200	3.73886700	-0.84363700
H	-3.59877100	1.71695100	1.58894600
H	-5.22973500	3.61116800	1.70730100
H	-4.95973600	5.54490100	0.19151500
H	-3.10148500	5.63202600	-1.43310400
H	-1.47416300	3.75261200	-1.55174400
C	4.27921800	-0.10725600	-1.40018600

C	4.38060300	1.10132900	-0.51925800
H	5.09844700	-0.12869200	-2.13251400
H	4.32362700	-1.01645100	-0.79251600
C	3.69651300	1.12800200	0.69698900
C	5.11406700	2.21794400	-0.90969100
C	5.16844100	3.34626100	-0.09788700
C	4.48499900	3.36543000	1.11214100
C	3.74918200	2.25257800	1.50925900
H	3.12912800	0.25706700	1.00629400
H	5.64551400	2.20422000	-1.85697300
H	5.74557300	4.21037000	-0.41061600
H	4.53071500	4.24380600	1.74790000
H	3.22234200	2.25284400	2.45826800
C	1.79030800	-1.94351000	2.06833000
C	0.73373300	-1.28837100	2.69829800
C	0.94464300	-0.59242300	3.88337600
C	2.21498100	-0.54379800	4.44623700
C	3.27250200	-1.20413500	3.82828200
C	3.05822900	-1.90222700	2.64692600
H	-0.26135000	-1.33362700	2.26778000
H	0.11052600	-0.09609100	4.36857300
H	2.38028300	-0.00195100	5.37189000
H	4.26405000	-1.17443500	4.26775300
H	3.88439000	-2.41371700	2.16099600
S	-2.88622200	-1.79536300	0.95105400
C	-4.09459000	-1.33240600	-0.38637400
F	-5.25971700	-0.94315100	0.12182100
F	-3.60212800	-0.30014500	-1.10589800
F	-4.30816800	-2.33969100	-1.22155500
O	-3.49307200	-2.91432100	1.64095300
O	-1.66216400	-2.07300600	0.17617700
O	-2.78010800	-0.53373300	1.72155200
C	1.58171000	-2.61510300	0.74577300
H	0.51987700	-2.83620700	0.59470900
H	2.15557200	-3.55007400	0.67896200
H	1.22258500	0.56850000	-0.76224000
H	-0.08745500	-0.35613600	0.08002600
C	-1.56931900	1.52510200	0.01082700

H	-1.73453600	0.71762600	0.73925500

Gal-oxo-OTf

Charge=0, Multiplicity=+1

C	-0.59471500	-1.32477200	0.87236300
C	0.76916400	-1.39209200	0.20714900
C	0.67084000	-1.21095500	-1.28808400
O	1.32957000	-2.66933300	0.47954800
H	1.39185300	-0.57493800	0.58365500
O	-0.29980800	-2.19345600	-1.85326300
H	0.30548600	-0.22694100	-1.59380600
C	1.99631300	-1.49642000	-1.94114900
C	-1.23997600	-2.64380200	-1.15818300
C	-1.43904900	-2.45903300	0.31140000
H	-1.02251100	-3.39189000	0.73061800
O	-2.79825400	-2.48453600	0.62122900
H	-1.02984300	-0.35500100	0.60851700
O	-0.51654400	-1.48476300	2.25790700
H	-1.87118700	-3.36339800	-1.67461000
O	2.53859500	-2.72726400	-1.50142600
H	2.64949100	-0.64824000	-1.69967300
H	1.89666300	-1.56278400	-3.02478600
C	2.60402600	-2.83412900	-0.10090700
C	3.63522900	-1.91218400	0.52143600
H	2.86650500	-3.87733800	0.08890700
C	4.75779800	-1.52057900	-0.20283900
C	5.71989600	-0.70779500	0.38421200
C	5.56931700	-0.28760100	1.70025100
C	4.45692000	-0.68951200	2.43084000
C	3.49438200	-1.50174400	1.84580800
H	4.87167000	-1.85176500	-1.22980500
H	6.58651700	-0.39784800	-0.19004300
H	6.31623700	0.35411400	2.15521400
H	4.33454500	-0.36399900	3.45845400
H	2.62115700	-1.81409100	2.40979700
C	-0.48323400	-0.24702800	2.97916900
C	-1.98255700	1.61393500	2.19726000
C	-1.81625400	0.44541600	2.93785000

C	-2.92014600	-0.12081600	3.57969000
C	-4.17097100	0.47141500	3.47600900
C	-4.33347200	1.63222900	2.72122500
H	-0.21691300	-0.53370200	3.99862400
H	0.29852200	0.40706500	2.57801900
H	-2.79606000	-1.03840000	4.14726200
H	-5.02355200	0.02776300	3.98027600
C	-3.23931100	2.20230400	2.08262100
H	-1.13174900	2.05249700	1.68779900
H	-5.31386600	2.08984100	2.63529300
H	-3.35077000	3.10052000	1.48458100
C	-3.50990700	-1.29526800	0.24023400
C	-3.26143300	-0.93527000	-1.19536200
H	-3.24219800	-0.46668800	0.89992200
H	-4.55706900	-1.54601500	0.41715700
C	-2.58815000	0.24342600	-1.53186000
C	-2.30549100	0.54001100	-2.85875400
C	-2.71758900	-0.32793000	-3.86564700
C	-3.70588800	-1.78184600	-2.22495300
C	-3.43285200	-1.48049900	-3.55160700
H	-2.28737300	0.94590900	-0.75907400
H	-1.74469700	1.43834100	-3.08940900
H	-2.49273600	-0.09699200	-4.90165500
H	-4.26889700	-2.67600300	-1.96957100
H	-3.77978100	-2.13949000	-4.34057700
C	1.56901000	3.78172200	-0.80267100
S	0.34016500	2.39095300	-0.81168000
F	1.49961300	4.45628600	0.34279000
F	1.33243700	4.62501600	-1.80364600
F	2.80775700	3.30823200	-0.93594500
O	0.74696700	1.55981900	0.33745800
O	0.55955600	1.73417200	-2.11161400
O	-0.96280000	3.03802700	-0.65808600

Gal-6B-OTf

Charge=0, Multiplicity=+1

C	-1.13684000	-1.36569600	-0.47772800
C	-0.26913200	-1.56923400	-1.71747600

C	-0.73260300	-2.73259900	-2.58291100
O	1.11232600	-1.83921600	-1.31984400
H	-0.27538300	-0.65450100	-2.31444200
O	-2.02918500	-2.37347600	-3.01974100
H	-0.08433800	-2.85803800	-3.45152700
C	-0.99816300	-4.03069400	-1.81926500
C	-2.80920100	-2.65132100	-1.87804000
C	-2.63151700	-1.54166200	-0.83909400
H	-3.12422800	-1.85326600	0.09130000
O	-3.25862100	-0.42349400	-1.40628300
H	-0.95552400	-0.35107700	-0.10851900
O	-0.77392500	-2.32420900	0.49128400
O	-2.34919800	-3.88850100	-1.39204900
H	-3.85315200	-2.72648200	-2.18483000
H	-0.34662500	-4.15863500	-0.95338100
H	-0.92379400	-4.89546300	-2.48500800
C	1.81554600	-0.86627000	-0.89168400
C	3.15836900	-1.06873100	-0.49635300
C	3.73898500	-2.35074300	-0.43065600
C	5.05997000	-2.47123000	-0.06314800
C	5.80733200	-1.32385200	0.23231900
C	3.90254500	0.08031700	-0.17638800
C	5.23697600	-0.05914800	0.17599500
H	5.52374600	-3.44920600	-0.00220700
H	6.85029300	-1.42906600	0.51426900
H	3.42158400	1.05651800	-0.21903500
H	5.82635300	0.81878800	0.41386200
C	-0.78111300	-1.83784400	1.83263000
C	0.53267600	-1.20608900	2.20132000
H	-0.96954300	-2.71530200	2.45549400
H	-1.60801300	-1.13334500	1.97868600
C	1.62340700	-2.01051000	2.54055700
C	2.84069000	-1.44017100	2.88494000
C	2.98472600	-0.05412700	2.88628700
C	1.91148200	0.75584000	2.53742300
C	0.69064200	0.17984200	2.20099300
H	1.50744200	-3.09093400	2.54573200
H	2.01090000	1.83526700	2.51648200

H	-0.14514800	0.82404300	1.95254400
H	3.67804000	-2.07398900	3.15895200
H	3.93482200	0.39182500	3.16171500
C	-3.16638400	0.81752900	-0.71344500
C	-3.52391000	0.74699900	0.74643400
C	-2.78676400	1.48381300	1.67239500
C	-3.10244100	1.42706600	3.02638700
C	-4.14837700	0.62556600	3.46780800
C	-4.58767600	-0.03790800	1.19331300
C	-4.89362500	-0.10493800	2.54642800
H	-1.96318600	2.10817300	1.33367300
H	-2.52060500	2.00633400	3.73610800
H	-4.38751500	0.57285500	4.52494500
H	-5.17289600	-0.60106100	0.47200300
H	-5.71762000	-0.72543900	2.88399900
S	0.69429700	2.72436300	-0.38884700
C	0.30760800	4.07694300	-1.60061100
O	0.10218800	1.52833400	-1.04075100
O	0.01880800	3.12738300	0.84139200
F	0.78253400	5.24072300	-1.17092000
F	-1.01013400	4.18867000	-1.75885200
F	0.85123800	3.80968700	-2.78687200
H	-2.16709700	1.24880100	-0.83339400
H	-3.86773600	1.46731800	-1.24474900
H	3.13800600	-3.22260800	-0.66207800
O	2.15957700	2.68171300	-0.33916200
H	1.35471000	0.13179400	-0.87859600

Gal-4B-OTf

Charge=0, Multiplicity=+1

C	0.04569900	1.40490700	-1.51331300
O	0.06319600	2.82965400	-1.52761800
C	-1.31302200	1.09449700	-2.16062900
C	1.19186000	0.85060800	-2.31585100
H	0.09757400	1.02586000	-0.48849000
C	-2.37635000	1.39808500	-1.09979300
C	-2.30992800	2.94472100	-1.08415800
H	-2.10474700	0.93680800	-0.14791700

O	-3.67305900	1.01518000	-1.48266500
C	-1.18469200	3.18469000	-2.10556500
O	-2.05811900	3.63557500	0.11248700
H	-3.25550500	3.29682800	-1.51412500
O	-1.42708200	2.19572700	-3.07005600
H	-1.39427700	0.14000200	-2.67377900
H	-1.11817900	4.17800700	-2.54253900
O	2.45408600	1.19744100	-1.65379300
H	1.26280200	1.31576300	-3.29665100
H	1.12129500	-0.23664100	-2.36624400
C	2.86062200	0.43382500	-0.72685700
C	4.05177900	0.72638900	-0.01010500
H	2.31134400	-0.48799200	-0.48484100
C	4.46820100	-0.23736900	0.92488200
C	5.61701700	-0.00852200	1.66355200
C	6.33370500	1.16913900	1.47477500
C	5.91945200	2.12873000	0.54537500
C	4.78084700	1.91332400	-0.20218400
H	3.87566600	-1.14052300	1.04631000
H	5.95297600	-0.74175700	2.38750800
H	7.23019400	1.34885900	2.05942200
C	-2.21616100	2.95409000	1.34309200
C	-0.99149500	2.16526600	1.73657000
H	-3.09826100	2.30269800	1.33123600
H	-2.40287100	3.74350300	2.07789700
C	-1.10414400	0.84541400	2.16830100
C	0.02719300	0.10099000	2.48258200
C	1.28440600	0.68351700	2.38040600
C	0.27312000	2.74572800	1.64168400
C	1.40518000	2.00924200	1.96611500
H	-2.08368800	0.38135500	2.23207100
H	-0.07453500	-0.93996200	2.76820000
H	2.16723200	0.10202300	2.62343800
H	0.36290900	3.76343800	1.27803600
H	2.38639900	2.46975200	1.89554700
C	-3.93244000	-0.36589500	-1.27664600
C	-3.96357600	-0.74943800	0.17950200
H	-4.90577400	-0.53920300	-1.74294400

H	-3.19409300	-0.98580100	-1.80178900
C	-3.13500500	-1.75576100	0.66512100
C	-3.16811100	-2.11463000	2.00923500
C	-4.02978000	-1.45937300	2.87929300
C	-4.85454500	-0.44063900	2.40399500
C	-4.82213100	-0.08872200	1.06148100
H	-2.43273500	-2.24544000	0.00035700
H	-2.49293500	-2.88559600	2.36302600
H	-4.05464400	-1.73114700	3.92970000
H	-5.52504100	0.07664200	3.08296600
H	-5.46123400	0.70621000	0.68776200
H	6.49213000	3.04016800	0.41741700
H	4.43344600	2.64498200	-0.92295100
S	0.61150700	-2.53065200	-0.00321500
C	0.74631500	-3.98637400	-1.14820500
F	1.36266500	-5.00501500	-0.55916300
F	1.44081100	-3.65208000	-2.24114500
F	-0.46320900	-4.38874800	-1.53372000
O	-0.15583000	-3.02420300	1.13014500
O	-0.04923400	-1.51346700	-0.84240500
O	2.02528500	-2.20021300	0.27929700
